# Alkynyl nicotinamides show antileukemic activity in drug-resistant acute myeloid leukemia

**DOI:** 10.1172/JCI169245

**Published:** 2024-06-17

**Authors:** Baskar Ramdas, Neetu Dayal, Ruchi Pandey, Elizabeth Larocque, Rahul Kanumuri, Santhosh Kumar Pasupuleti, Sheng Liu, Chrysi Kanellopoulou, Elizabeth Fei Yin Chu, Rodrigo Mohallem, Saniya Virani, Gaurav Chopra, Uma K. Aryal, Rena Lapidus, Jun Wan, Ashkan Emadi, Laura S. Haneline, Frederick W. Holtsberg, M. Javad Aman, Herman O. Sintim, Reuben Kapur

**Affiliations:** 1 Department of Pediatrics, Herman B Wells Center for Pediatric Research, Indiana University School of Medicine, Indianapolis, Indiana, USA.; 2 Department of Chemistry, Purdue University, West Lafayette, Indiana, USA.; 3 Department of Medical and Molecular Genetics, Indiana University School of Medicine, Indianapolis, Indiana, USA.; 4 KinaRx, Inc, Rockville, Maryland, USA.; 5 Department of Comparative Pathobiology,; 6 Tyler Trent Pediatric Cancer Research Center, Purdue University Institute for Cancer Research,; 7 Department of Computer Science (by courtesy),; 8 Regenstrief Center for Healthcare Engineering,; 9 Purdue Institute for Drug Discovery, and; 10 Purdue Proteomics Facility, Bindley Bioscience Center, Purdue University, West Lafayette, Indiana, USA.; 11 University of Maryland Greenebaum Comprehensive Cancer Center, Baltimore, Maryland, USA.; 12 Department of Microbiology and Immunology, and; 13 Department of Molecular Biology and Biochemistry, Indiana University School of Medicine, Indianapolis, Indiana, USA.

**Keywords:** Hematology, Stem cells, Hematopoietic stem cells

## Abstract

Activating mutations of *FLT3* contribute to deregulated hematopoietic stem and progenitor cell (HSC/Ps) growth and survival in patients with acute myeloid leukemia (AML), leading to poor overall survival. AML patients treated with investigational drugs targeting mutant *FLT3*, including Quizartinib and Crenolanib, develop resistance to these drugs. Development of resistance is largely due to acquisition of cooccurring mutations and activation of additional survival pathways, as well as emergence of additional *FLT3* mutations. Despite the high prevalence of *FLT3* mutations and their clinical significance in AML, there are few targeted therapeutic options available. We have identified 2 novel nicotinamide-based *FLT3* inhibitors (HSN608 and HSN748) that target *FLT3* mutations at subnanomolar concentrations and are potently effective against drug-resistant secondary mutations of *FLT3*. These compounds show antileukemic activity against *FLT3^ITD^* in drug-resistant AML, relapsed/refractory AML, and in AML bearing a combination of epigenetic mutations of *TET2* along with *FLT3^ITD^*. We demonstrate that HSN748 outperformed the FDA-approved *FLT3* inhibitor Gilteritinib in terms of inhibitory activity against *FLT3^ITD^* in vivo.

## Abstract 2

Alkynyl nicotinamide compounds HSN608 and HSN748 are more effective than FDA-approved FLT3 inhibitors against FLT3^ITD^ in relapsed/refractory acute myeloid leukemia models bearing combination mutations.

## Graphical abstract

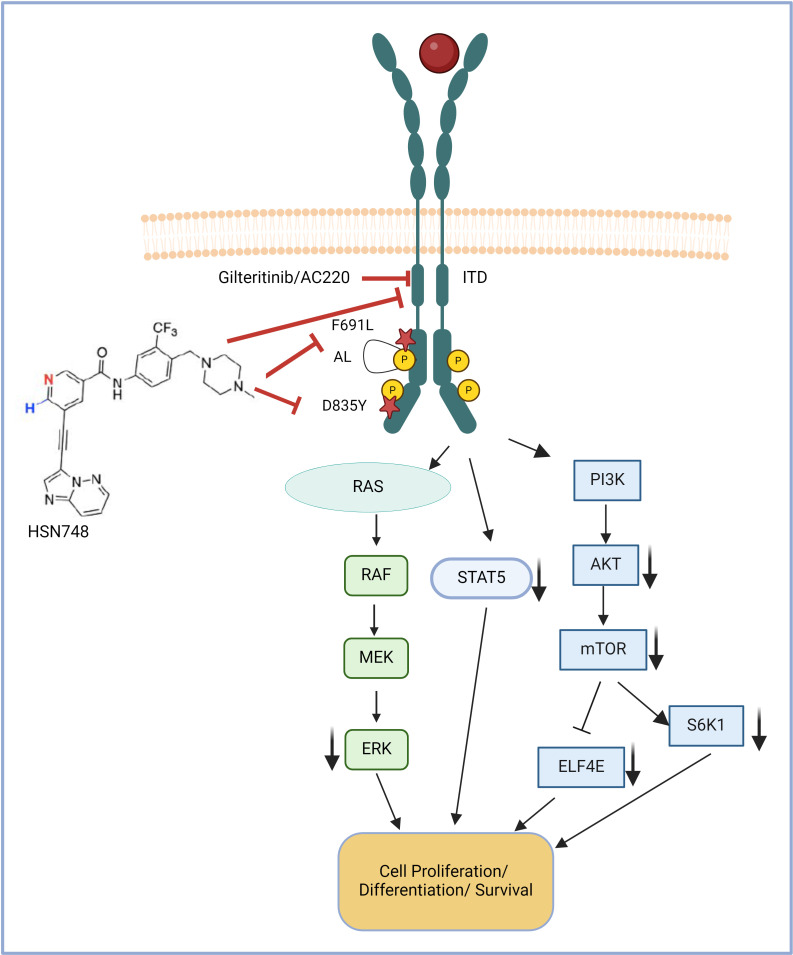

## Introduction

The prevalence of acute myeloid leukemia (AML) has increased in the past decade due to an increase in the aging population (1). Activating mutations in the fms-like tyrosine kinase 3 (FLT3) (2) are present in 25%–30% of patients with AML, approximately 10% of patients with myelodysplastic syndrome (MDS) (3), and 5–6% of patients with acute lymphoblastic leukemia (ALL). Common mutations include missense point mutations in the kinase domain, in-frame deletions and internal-tandem duplications (ITD) in the juxtamembrane domain, leading to constitutive activation of the receptor tyrosine kinase (RTK). While patients in the last decade have seen a significant improvement in survival rates with other forms of leukemia; there has been only a dismal increase in the overall survival of patients with AML. The 5-year survival rate remains around 29% for patients with AML, and *FLT3^ITD^* is considered an independent prognostic factor in these patients. Patients with *FLT3^ITD^* mutation are at a higher risk of disease relapse and reduced overall 5-year survival. These patients also demonstrate poor overall survival rate compared with patients with AML with other mutations (2, 4, 5).

Activating mutations of *FLT3* contribute to dysregulated proliferation of hematopoietic progenitor cells (6). Recently, 2 kinase inhibitors, Midostaurin (RYDAPT) and Gilteritinib (XOSPATA) have been granted FDA approval for treatment of AML. Midostaurin (7) is a multikinase inhibitor and is effective primarily during the induction and consolidation phase of AML treatment in combination with chemotherapy, while Gilteritinib, a *FLT3^ITD^* inhibitor, with activity against other kinases such as AXL, was approved for refractory and relapsed patients with AML (8). Given the poor survival rates of patients diagnosed with these mutations and lack of treatment options, the FDA fast tracked Gilteritinib and Quizartinib; ultimately, Gilteritinib was approved, but not Quizartinib. Additional experimental drugs specific for mutant *FLT3,* which are in various stages of clinical trials, including Quizartinib and Crenolanib, have also been described in the literature, although some of these are associated with the development of drug resistance (2, 3, 5, 9). While the intrinsic resistance in AML *FLT3*–directed inhibitors depends on the presence of cooccurring mutations (5), acquired resistance is due to activation of parallel survival pathways and/or acquisition of secondary mutations in *FLT3^ITD^* (2, 3, 9). We (10) and others (11) have demonstrated that the cooccurrence of *FLT3^ITD^* mutations with epigenetic mutations can promote the self-renewal of hematopoietic stem cells (HSCs) and transform these cells into AML (1, 10–14). Characterization of the mutational landscape in patients with AML has shown that mutations in epigenetic regulators are among the most frequent cooccurring mutations with *FLT3^ITD^*. These include mutations in epigenetic regulators such as *DNMT3A*, *TET2*, and *IDH1*. When mutations in these regulators in mice are combined with *FLT3^ITD^*, a fully penetrant, rapid onset, and fatal AML develops (11, 15, 16). Consistently, in patients with AML, the combination of *FLT3^ITD^* with mutations in DNA methylation regulators results in reduced overall survival and increased risk of relapse (11). Importantly, presence of *TET2* mutations is frequently associated with primary resistance to Crenolanib (5). Similar resistance to Quizartinib (AC220) has been noted in mouse models of AML carrying heterozygous combinations of either *Tet2* or *Idh1* mutation along with *FLT3^ITD^* (15). More recently, emergence of RAS mutations has been reported in patients with AML who have been treated with Gilteritinib (2). However, despite the high prevalence rate and the clinical significance of *FLT3* mutations in the pathogenesis of AML, targeted therapeutic options are still limited. Thus, there is a critical unmet need to further identify and develop potent and selective inhibitors for mutant *FLT3* to provide additional therapeutic options for treating patients with AML with these mutations.

In the current study, we report what we believe to be a novel class of nicotinamide-based *FLT3* inhibitors that not only selectively target *FLT3^ITD^* at subnanomolar concentrations but are also effective against the drug resistance, conferring secondary mutations acquired in response to targeted therapy in the *FLT3^ITD^*. We studied the biological effects of what are, to our knowledge, 2 novel *FLT3* inhibitors, HSN608 and HSN748, on primary de novo AML, drug resistant AML, and relapsed/refractory AML with *FLT3* mutations. Compared with Gilteritinib, an FDA-approved *FLT3* inhibitor, our compounds inhibit both *FLT3^ITD^* and *FLT3*-gatekeeper mutations in AML with increased potency, resulting in robust and deeper responses compared with currently available FLT3 inhibitors.

## Results

### HSN748, a type II inhibitor of FLT3 mutants.

**Figure 1:**
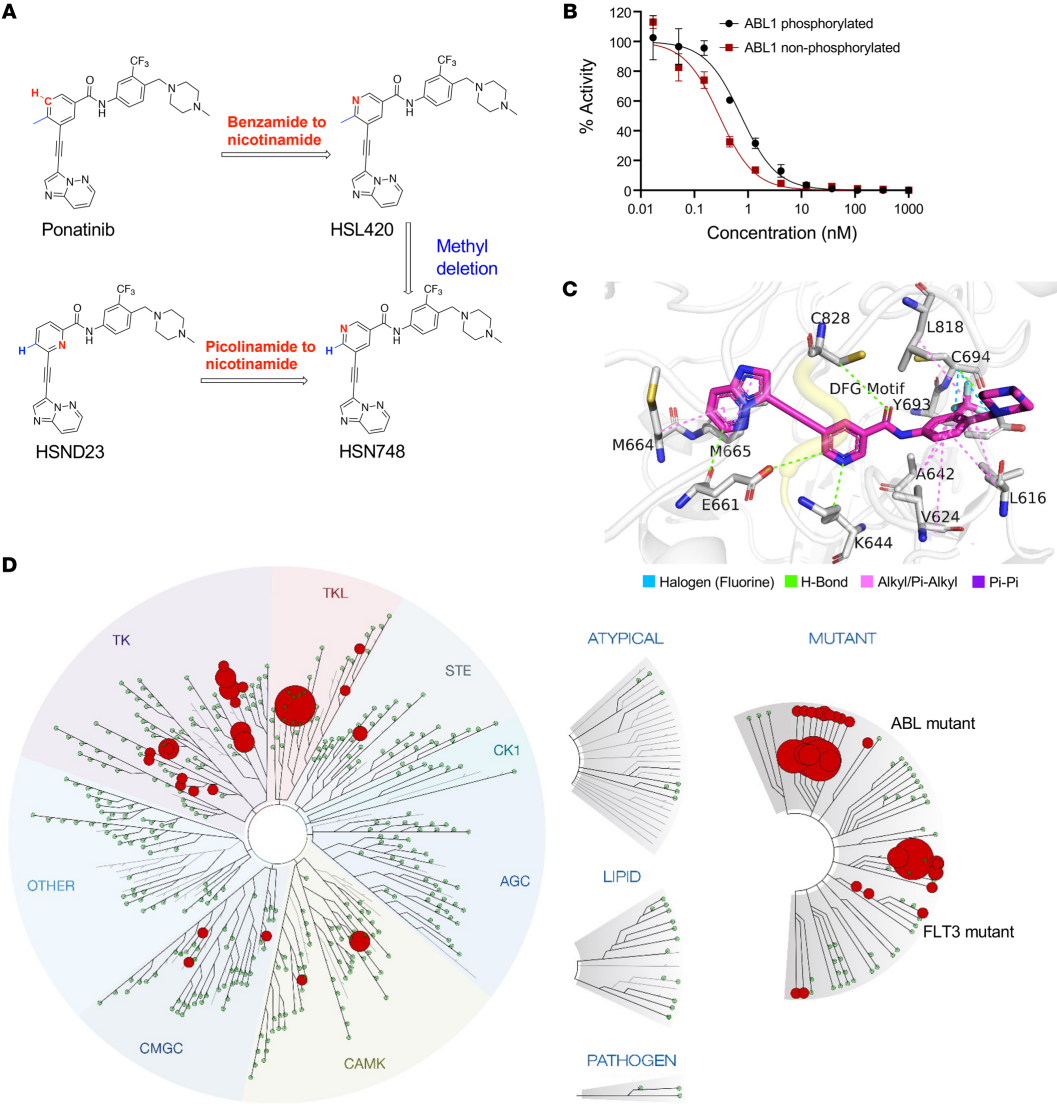
HSN748, A type II inhibitor of FLT3 and FLT3 mutants. (**A**) Nicotinamide conversion of the benzamide moiety of the ponatinib-derived HSN748 compound structure. (**B**) In vitro inhibition of phosphorylated ABL. HSN748 preferentially binds the nonphosphorylated form of ABL over the phosphorylated. (**C**) Docked pose of HSN748 binding to FLT3; showing all interactions in the binding pocket. DFG motif shown in yellow. (**D**) Binding assays using the KINOMEScan platform. Binding of HSN748 at a single concentration (10 nM) is depicted as dots in the kinome tree schematic.

We have previously reported that the conversion of the benzamide moiety of ponatinib into a nicotinamide yields compounds with improved selectivity and potency against *FLT3* (17). To further enhance the selectivity and potency of these compounds, we modified the piperazine moiety of HSN748 with various cyclic and acyclic rings (Figure 1A and Supplemental Table 1A supplemental material available online with this article; https://doi.org/10.1172/JCI169245DS1). We carried out structure-activity–relationship studies of the synthesized analogs by comparing the inhibition of *FLT3^ITD–F691L^*, *FLT3^ITD–D835Y^*, MOLM14, and MV4-11 AML cell lines. In general, modifications to the piperazine ring found in HSN748 were tolerated for the inhibition of the *FLT3^ITD^*-mutated cell lines (MOLM14 and MV4-11) but were detrimental for the inhibition of the secondary mutated cell lines (MOLM14, *FLT3^ITD–F691L^,* and *FLT3^ITD–D835Y^*). For example, replacement of piperazine with a bicyclic ring (Supplemental Table 1) maintained inhibition of MV4-11 and MOLM14 but did not inhibit the secondary mutated cell lines MOLM14 *FLT3^ITD–F691L^*, and *FLT3^ITD–D835Y^* (Supplemental Table 2). Changing the piperazine moiety in HSN748 to dimethylaminopyrrolidine (Compounds HSND08 and HSND09) adversely affected the inhibition of MOLM14 *FLT3^ITD–D835Y^* cell line (IC_50_ for HSN748 = 5 nM versus greater than 50 nM for compounds HSND08 and HSND09). Likewise, changing the piperazine (6-membered ring) in HSN748 to 1, 4-diazepane (7-membered ring, HSND10) also reduced activity against MOLM14 *FLT3^ITD–D835Y^* (Supplemental Table 1). Modification of the piperazine group in HSN748 with 4-membered aziridine ring (Supplemental Tables 1 and 2, HSND02) drastically reduced the activity toward all 4 *FLT3*-mutated cell lines (Supplemental Table 2).

Sticking with the 6-membered piperazine moiety, we investigated the effects of other modifications. The incorporation of a methyl group at the 2-position of the piperazine ring (Compounds HSND05 and HSND06) slightly increased the IC_50_ against the *FLT3* mutation–bearing cell lines (Supplemental Table 2). Also, modifying the piperazine with an isopropyl group (instead of the methyl group found in HSN748) to afford compound HSND07 led to a poorer inhibitor of the *FLT3*-mutated cell lines (Supplemental Table 2). Modifying the piperazine with hydroxyethyl group (compound HSND12, Supplemental Tables 1 and 2) adversely affected the inhibition of MOLM14 *FLT3^ITD–D835Y^* cell line (Supplemental Table 2).

To investigate the importance of the trifluoromethyl group, we replaced the trifluoromethyl with chloro, which led to reduced activity against MOLM14 *FLT3^ITD–F691L^* and *FLT3^ITD–D835Y^*. Previously, we reported that changing the methyl benzamide moiety in ponatinib to a nicotinamide moiety led to abrogation of c-Src inhibition and enhancement of activities against the clinically relevant secondary mutations *FLT3^ITD–F691L^* and *FLT3^ITD–D835Y^* (17). We wondered if the analogous picolinamide regioisomer analog (compound HSND23) would also display a potent *FLT3^ITD^* inhibition profile like HSN748. To our surprise, compound HSND23 was orders of magnitude worse than both HSN748 and ponatinib, highlighting that, while the incorporation of nitrogen into drugs is a common tactic to improve activity, the position of the nitrogen is an important factor to consider.

To determine if HSN748 is a type I or a type II inhibitor, we determined the degree of inhibition against phosphorylated and nonphosphorylated ABL1. In vitro inhibition of phosphorylated ABL1 is often used as a surrogate to determine if a kinase inhibitor is Type I or Type II without the need for crystal structures (18). We determined that HSN748 preferentially (albeit marginally) binds the nonphosphorylated form of ABL (dissociation constant, or Kd = 0.29 nM) compared with the phosphorylated (active) enzyme (Kd = 0.74 nM) suggesting that it is a type II inhibitor (Figure 1B and Table 1). This assertion of Type II inhibition agrees with that of ponatinib (the parent compound), which is known to bind as Type II (PDBs 4VO4, 3ZOS, 3OXZ, and 4VOI). Next, to gain a deeper insight into the mode of binding of HSN748 to *FLT3,* we performed molecular dynamics simulations of inhibitor binding on *FLT3* (Figure 1C). We sought to determine whether HSN748 could inhibit other clinically relevant *FLT3* mutations, including *FLT3^ITD–F691L^* and *FLT3^ITD–D835Y^*. In this regard, we performed binding assays using the KINOMEScan platform (DiscoveRx) and commercially available *FLT3* mutants; the Kd for most of the tested proteins was in the sub-to-low nanomolar range with the exception of *FLT3^ITD–D835Y^* (Table 2). Importantly, there was no loss of binding affinity for the *FLT3^ITD–F691L^* mutant, which confers resistance to all approved *FLT3* inhibitors (19). To determine the selectivity of HSN748, we performed KINOMEScan analysis to assess binding of HSN748 to more than 450 human kinases. Binding of HSN748 at a single concentration (10 nM) is depicted as dots in the kinome tree schematic (Figure 1D).

### HSN748 docking with FLT3 reveals favorable structural interactions.

**Figure 2:**
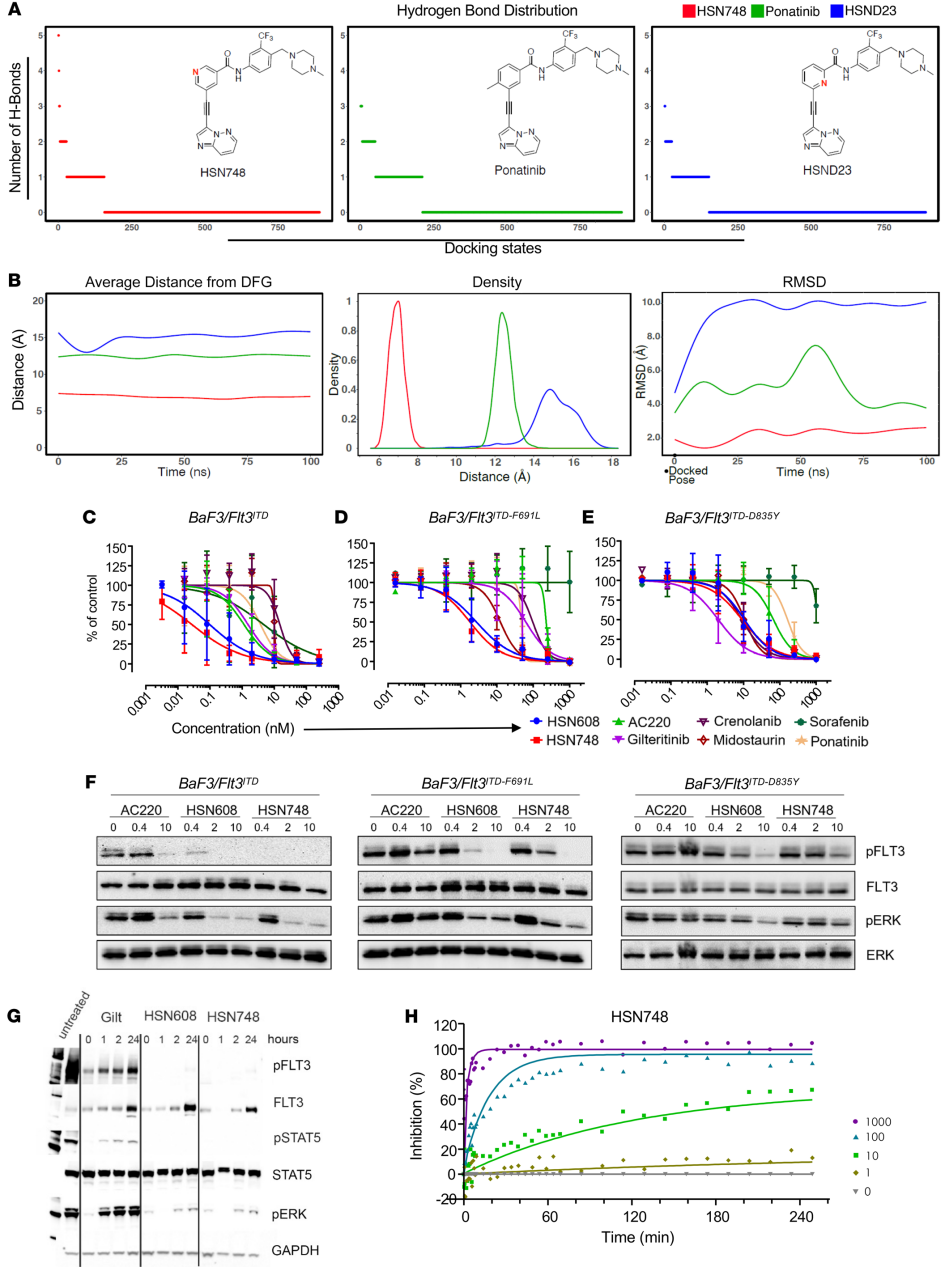
Superior growth inhibitory effect of HSN608 and HSN748 compared with FDA-approved FLT3 inhibitors on the proliferation of leukemic cells. (**A**) Hydrogen bond distribution plots of all docking states of HSN748 (red), Ponatinib (green), and HSND23 (blue) with FLT3. (**B**) Distance distribution plots using MD simulations. (Left) Average distance plots from DFG Motif for HSN748 (red), Ponatinib (green), and HSND23 (blue); (middle) Probability density plot over entire MD simulation for finding HSN748 (red), Ponatinib (green), and HSND23 (blue) at a specified distance from DFG Motif (x-axis); (right) RMSD plot of HSN748 (red), Ponatinib (green), and HSND23 from starting (*t* = 0) docked conformation over time. Murine BaF3 cells expressing (**C**) *Flt3^ITD/ITD^* (**D**) *Flt3^ITD/F69L^* and (**E**) *Flt3^ITD/D835Y^* receptors were cultured with the indicated concentrations of the inhibitors for 48 hours and proliferation was estimated by colorimetric assay. Data presented were pooled from 3 independent experiments. (**F**) Murine BaF3 cells expressing *Flt3^ITD/ITD^, Flt3^ITD/F69L^,* and *Flt3^ITD/D835Y^* receptors were cultured in cytokine-free media with the indicated nanomolar concentrations of HSN608 and HSN748 for 1 hour and cell lysates were subjected to Western blotting to assess for FLT3 and ERK activation (*n* = 3). (**G**) Western blot data showing the length of time HSN748 is engaged and inhibits the FLT3 target. We treated MV411 cells bearing *FLT3^ITD^* for 1 hour with Gilteritinib, HSN608, and HSN748 before removing the drug from culture media with sequential washes and allowing the cells to recover for 0, 1, 2, and 20 hours in culture media without inhibitor. This was followed by Western blotting. (**H**) FRET data showing prolonged occupancy of HSN748 duration on *FLT3^ITD^* receptor.

FLT3 crystal structure (PDB ID: 4XUF) with DFG-out motif — when the aspartate is away from the binding site — an inactive kinase, was selected for docking calculations using CANDOCK (a docking software developed in-house) to evaluate molecular interactions of small molecules with the kinase binding site (20). The binding site, shown as a purple mesh in Supplemental Figure 1A, was used for docking HSN748, Ponatinib, HSND23 and HSN420. Figure 1C shows docked interactions of HSN748 in the binding pocket of FLT3 and the DFG motif. A fluorine atom on HSN748 forms a hydrogen bond interaction and the other 2 fluorine atoms form a halogen bond with Cys694. An oxygen atom and a nitrogen atom on the small molecule also form hydrogen bonds with Cys828 and Lys644, respectively, and other alkyl interactions/pi-pi interactions, shown in pink, suggest several stabilizing interactions of HSN748 in the binding pocket. In comparison, the docking interactions of Ponatinib, HSND23, and HSL420 with FLT3 were less favorable than HSN748 (Supplemental Figure 1B). Specifically, while evaluating the number of hydrogen bonds (H-bonds) over all the docking poses predicted by CANDOCK for each compound (Figure 2A and Supplemental Figure 2A), we found that HSN748 had favorable poses with a maximum 5 H-bonds, compared with Ponatinib and HSND23 with 3 H-bonds. A larger number of favorable interactions for HSN748, including more hydrogen bonds, indicates stable binding interactions toward stronger binding affinity when compared with Ponatinib and HSND23.

### Molecular dynamics simulations show strong association of HSN748 with FLT3 compared with Ponatinib.

In order to validate that favorable interactions were stable and also translated to strong association over time, we performed 100 ns–long molecular dynamic (MD) simulations on docked poses of small molecules in FLT3 in explicit water as solvent. We evaluated the change in distance of the small molecule conformations to the DFG motif (D829, F830 and G831 residues) using the equilibrated MD simulations (Supplemental Figure 2B). Over the 100 ns simulation time, the average distance between HSN748 and the DFG motif is about 5.5–8 Å (Figure 2B), which is substantially closer than the distance between Ponatinib at 11–15 Å and HSND23 at 13 Å, suggesting higher association of HSN748 with FLT3. Further, over the entire MD simulation, HSN748 conformations show the highest probability to be closer to the DFG motif compared with Ponatinib and HSND23 (Figure 2B). We also computed the average association time of these molecules within 10 Å from the DFG motif over MD simulations normalized to Ponatinib; HSN748 was 27.5 times more associated with the FLT3 DFG motif compared with HSND23, which was only 2.6 times higher than the Ponatinib association time. All of these data suggest that HSN748 is strongly associated with FLT3, resulting in potent binding and inhibition of its function. In addition to association, we also calculated the root-mean-square deviation (RMSD) to show any changes in the protein structure from the docked starting conformations with the small molecule. HSN748 shows the least change in protein structure, compared with HSND23, with the most change over the 100 ns simulation. These results indicate that the HSN748 prefers to stay closer to the DFG motif in the binding site compared with the HSND23, which suggests that the more exposed nitrogen atom in the HSN748 is important for potent binding and stability of the protein–small molecule complex rather than the more hindered position in the HSND23.

### Superior growth inhibitory effect of HSN608 and HSN748 compared with FDA-approved FLT3 inhibitors on the proliferation of leukemic cells.

Next, to further evaluate these inhibitors, we compared the effect of HSN748 and analogous alkynyl nicotinamides — HSN608 (a naphthyridine) and HSN431 (an isoquinoline) — which we previously showed to be potent FLT3 inhibitors (21), with the FDA approved FLT3 inhibitors, including AC220, Gilteritinib, Crenolanib, Midostaurin, Sorafenib, and Ponatinib on the growth of murine BaF3 cells expressing *FLT3^ITD^*, *FLT3^ITD–F691L^* (gatekeeper mutation), and *FLT3^ITD–D835Y^* (activation loop mutation). As shown in Figure 2C, HSN748 and HSN608 showed a greater growth inhibition at subnanomolar concentrations against *FLT3^ITD^* with IC_50_ values of 0.04 nM (HSN748) and 0.09 nM (HSN608). While FDA approved inhibitors AC220 (IC_50_, 0.83 nM) and Sorafenib (IC_50_, 0.9 nM) showed almost equal efficacy but weaker than HSN608 and HSN748 compounds. Likewise, Gilteritinib (IC_50_: 1.25 nM), Ponatinib (IC_50_, 2.54 nM), Midostaurin (IC_50_, 9.63 nM), and Crenolanib (IC_50_, 10.3 nM) showed weaker efficacy against *FLT3^ITD^* compared with HSN748 and HSN608. As demonstrated in Figure 2D, HSN748 and HSN608 showed greater growth inhibition at low nanomolar concentrations against *FLT3^ITD–F691L^*-expressing cells with IC_50_ values of 1.52 nM (HSN748) and 1.89 nM (HSN608). While FDA-approved inhibitors Midostaurin (IC_50_, 10.51 nM), Gilteritinib (IC_50_, 26.43 nM), Ponatinib (IC_50_, 45.97 nM), Crenolanib (IC_50_, 74.43 nM), AC220 (IC_50_, 223.7 nM), and Sorafenib (IC_50_, 1201 nM) showed significantly weaker efficacy than HSN608 and HSN748 against *FLT3^ITD–F691L^* gatekeeper mutants. As seen in Figure 2E, HSN748 and HSN608 showed a greater growth inhibition at nanomolar concentrations against *FLT3^ITD–D835Y^*-expressing cells with IC_50_ values of 6.62 nM (HSN748) and 7.45 nM (HSN608). While other FDA-approved inhibitors Crenolanib (IC_50_, 8.95 nM), Midostaurin (IC_50_, 10.7 nM), AC220 (IC_50_, 50.47 nM), Ponatinib (IC_50_, 162.5 nM), and Sorafenib (IC_50_, 1095 nM) showed weaker efficacy than HSN608 and HSN748 against *FLT3^ITD–D835Y^* activation loop mutant expressing cells. Of the 2 AC220 resistance-conferring mutations, proliferation driven by *FLT3^ITD^ ^F691L^* was more sensitive compared with *FLT3^ITD–D835Y^* to inhibition by both HSN608 and HSN748. These data confirmed that HSN608 and HSN748 are more potent than AC220 against not only *FLT3^ITD^* but also against acquired drug resistance-conferring mutations including FLT3^ITD-F691L^ FLT3^ITD-D835Y^ in cellular systems.

Next, we performed Western blot analysis to evaluate the effect of HSN608 and HSN748 on autophosphorylation of FLT3 and downstream ERK signaling compared with AC220. Biochemical analysis of cells treated with the inhibitors showed that HSN608 and HSN748 inhibited the phosphorylation of FLT3 and activation of ERK to a greater extent than AC220. (Figure 2F). Importantly, HSN608 and HSN748 at low nanomolar concentrations were also effective against the *FLT3^ITD–F691L^* and *FLT3^ITD–D835Y^* mutants, which are relatively resistant to AC220. Consistent with inhibition in growth, as shown in Figure 2, C–E, HSN608 and HSN748 inhibited the phosphorylation of FLT3 and ERK activation of all 3 BaF3 cell lines at much lower concentrations than AC220 (Figure 2F). Further, inhibition of cell proliferation correlated with inhibition of FLT3 signaling, as evidenced by reduction of FLT3 autophosphorylation and phosphorylation of ERK. Importantly, growth inhibition depends on the presence of FLT3 mutant alleles, as growth of BaF3 cells in the presence of IL3, which bypasses the requirement for oncogenic *FLT3^ITD^* signaling, rendered the cells insensitive to HSN748 (Supplemental Figure 3). Additionally, we performed comparative analysis of these inhibitor in *FLT3^ITD^-*positive MV411 and *FLT3^ITD^-*negative HL-60 AML cell lines of human origin (Supplemental Figure 3). A very high degree of specificity was observed with MV411 cell line being extremely sensitive to HSN608 and HSN748, while HL-60 was relatively resistant. The efficacy of these kinase inhibitors was determined not only by their potency but also by the length of time they engaged and inhibited their target (residence time). We treated MV411 cells for 1 hour with Gilteritinib, HSN608, and HSN748 before removing them from culture media with sequential washes and allowing cells to recover for 0, 1, 2, and 20 hours in culture media without inhibitor. As seen in Figure 2G, cells harvested at different times after recovery showed profound and prolonged target inhibition of pFLT3, pSTAT5, and pERK in cells treated with HSN608 and HSN748. We observed partial restoration of phosphorylation of FLT3 and downstream targets when the cells were treated with Gilteritinib starting at 1 hour after recovery. To determine the duration of drug target occupancy of HSN748, we measured the residence time of HSN748 on FLT3 in a fluorescence resonance energy transfer–based (FRET) assay. In vitro measurements showed that the residence time of HSN748 on FLT3 was 385 minutes, compared with Gilteritinib, which had a residence time of approximately 60 minutes, indicating 6.4-fold greater prolonged drug occupancy of HSN748 compared with Gilteritinib (Figure 2H). These findings suggest that HSN748 is not only effective, but also has a long duration of drug target occupancy, which is important for lead optimization of novel compounds.

### In vivo efficacy of HSN748.

**Figure 3:**
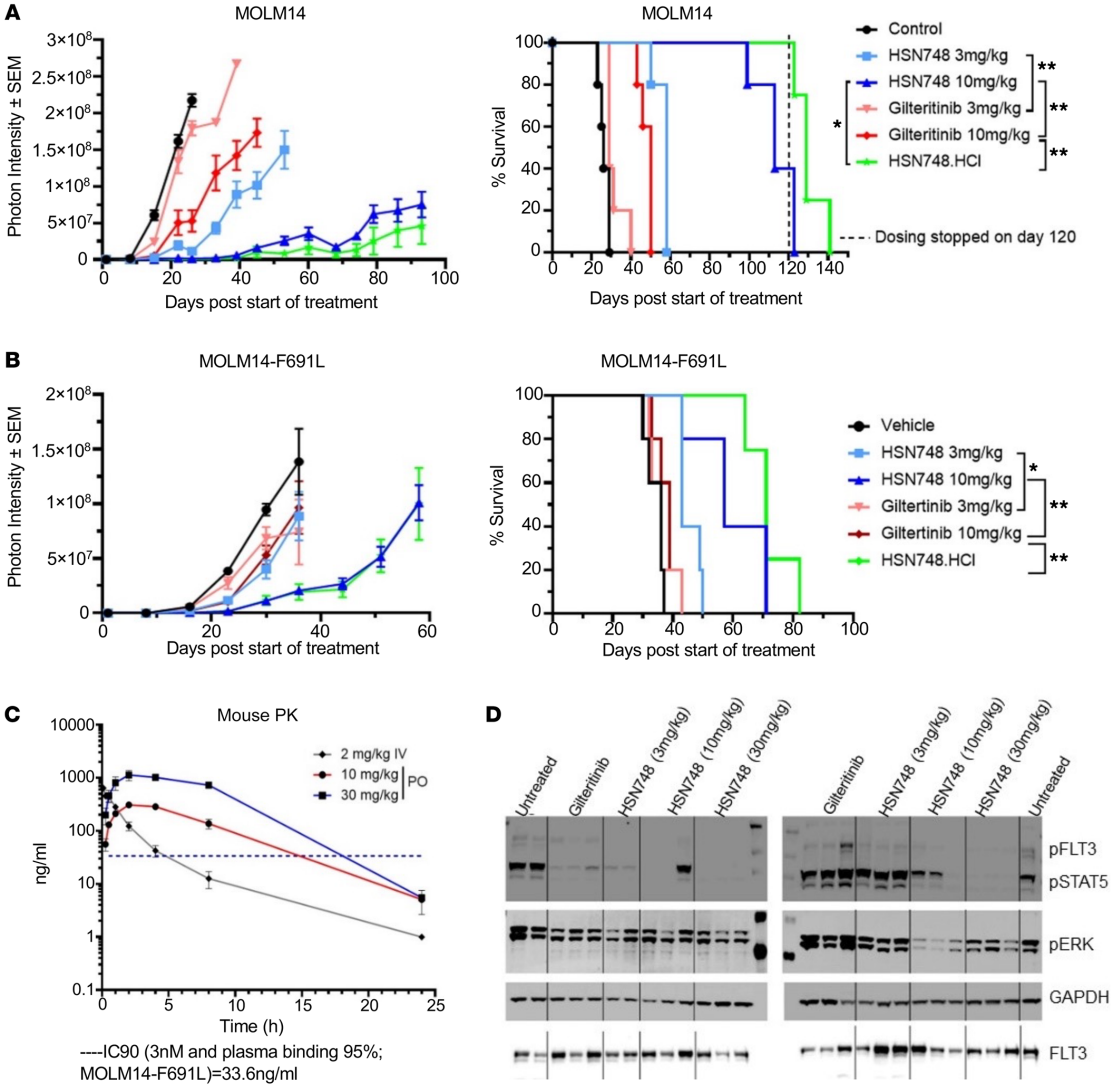
Prolonged survival of mice bearing FLT3 mutant cells treated with HSN748 compared with FDA-approved FLT3 inhibitor Gilteritinib. MOLM14 and MOLM14-*F691L* cells transduced with a luciferase reporter were implanted in NSG mice intravenously and treated with HSN748, Gilteritinib or an HCl salt form of HSN748 once daily and tumor cell growth was monitored by imaging for bioluminescence as a measure of disease burden. (**A** and **B**) Lowered photon intensity in MOLM14 and MOLM14-*F691L* recipients treated with HSN748 compared with Gilteritinib and controls. Right side panel shows the greater survival of mice treated with HSN748 (*n* = 5/group). (**C**) Pharmacokinetics of HSN748 after single dose of HSN748 to CD1 mice followed by both oral and intravenous mode of drug delivery. (**D**) Based on pharmacokinetic C_max_ values, lysates from tumors at 6 (left panel) and 24 hours (right panel) after drug administration were assessed for phosphorylation of FLT3, STAT5 and ERK.

Given that HSN748 was superior in inhibiting FLT3 mutant–driven cell lines compared with all other FLT3 inhibitors tested in vitro (Supplemental Figure 3), next we wanted to evaluate the efficacy of HSN748 in vivo. MOLM14 and MOLM14-*FLT3^ITD–F691L^* cells transduced with a luciferase reporter were implanted in NOD-*scid* IL2Rg^null^ (NSG) mice intravenously and treated with HSN748, Gilteritinib or an HCl salt form of HSN748 once daily. Tumor cell growth was monitored by imaging for bioluminescence as a measure of disease burden. As shown in Figure 3, A and B, HSN748 has a superior inhibitory effect on the tumor growth and survival advantage of MOLM14 and MOLM14-*FLT3^ITD–F691L^*-recipient mice compared with the Gilteritinib treated group. Next, we conducted pharmacokinetic and pharmacodynamic studies to confirm that the inhibition of FLT3 controlled tumor growth in vivo. In a separate experiment, we assessed the plasma concentration of HSN748.HCl in CD1 mice following intravenous and oral drug administration. As shown in Figure 3C, the plasma concentration of HSN748 peaked 3 hours after oral administration and dropped to around 5 ng/mL (9.6 nM) after 24 hours. Next, we assessed the effect of HSN748 on activation of FLT3, STAT5, and ERK under in vivo conditions. MOLM14 cells were implanted subcutaneously into NSG mice, and when tumors reached 300mm^3^ in size, the mice were given a single dose of HSN748 or Gilteritinib. Based on our pharmacokinetic Cmax values, we harvested tumors from 2–3 mice per group at 6 and 24 hours after drug administration, and lysates were assessed for phosphorylation of FLT3, STAT5, and ERK. As seen in Figure 3D, all phosphorylation events were reduced at 24 hours, with a more profound reduction observed with the 10 mg/kg and 30 mg/kg doses of HSN748. Interestingly, suppression of phosphorylation was maintained at 24 hours, with the higher doses of HSN748, while in mice treated with Gilteritinib phosphorylation of all proteins tested was comparable to untreated controls (Figure 3D; right panel). Overall, our findings indicate that HSN748 outperforms all rigorous therapeutic validation standards, from the in vitro BaF3 cell line system to the preclinical xenograft models.

### Effect of HSN748 on the proteome and phosphoproteome.

**Figure 4:**
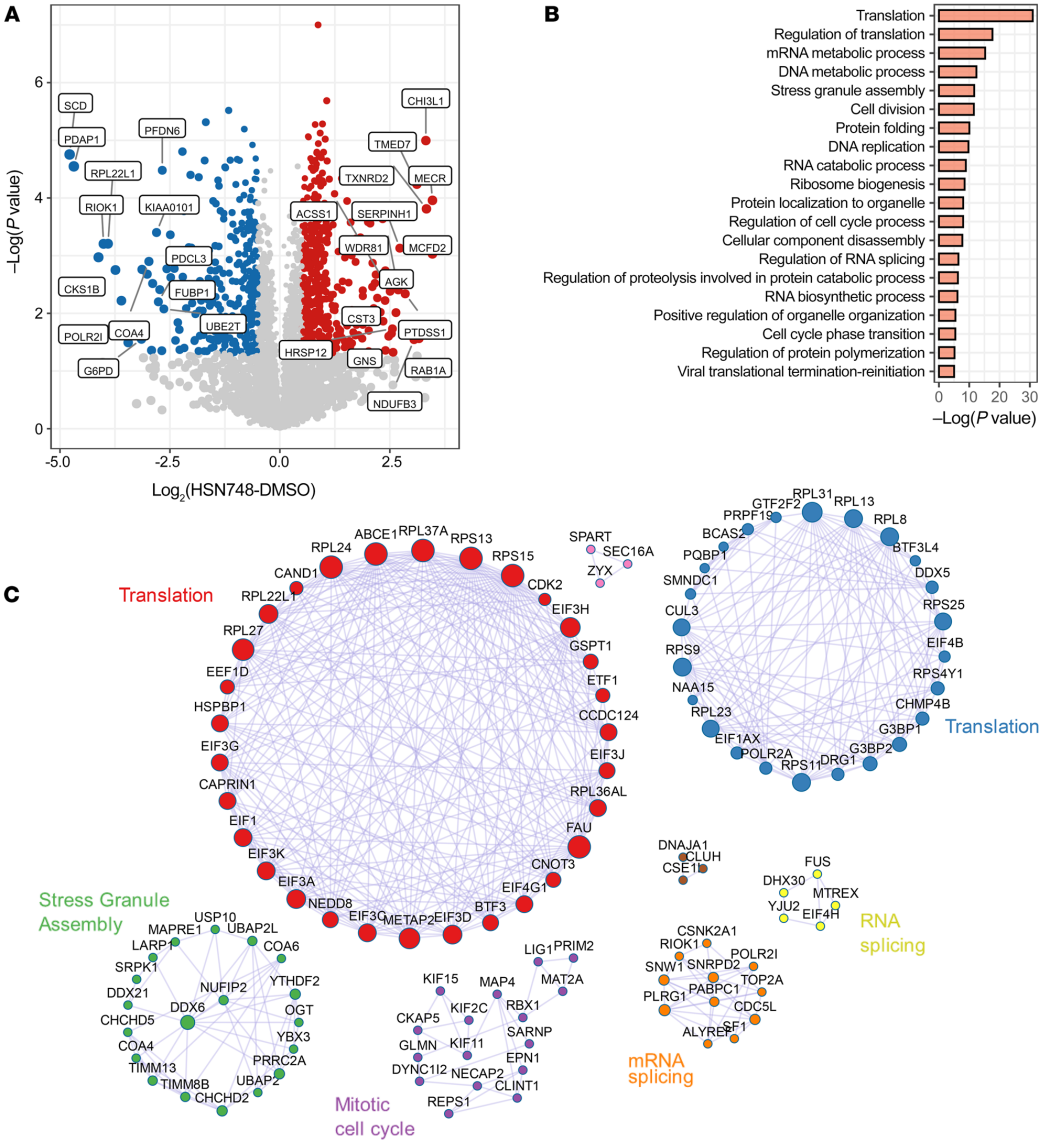
Effect of HSN748 on the proteome and phosphoproteomics. (**A**) Volcano plot analysis of global proteomics. Proteins with a *P* value and log_2_ (fold change) > 2.5 are labeled. Red hue represents significantly upregulated proteins (*P* < 0.05 and log_2_ (fold change) ≥ 0.5), and blue hue represents significantly downregulated proteins (*P* < 0.05 and log_2_ (fold change) ≤ 0.5). (**B**) Gene Ontology Biological Processes (GOBP) enrichment of downregulated proteins with log_2_ (fold change) ≤ 0.5 (**C**) Protein-protein interactions of downregulated proteins with log_2_ (fold change) ≤ –0.5.

To determine the effect of HSN748 on *FLT3*-mutated AML cells with regard to changes in global proteome, we treated MOLM14 cells with 100 nM HSN748 for 2 hours and 24 hours and performed MS-based bottom-up proteomics on both global-protein and phosphoprotein levels. Three biological replicates were collected per treatment condition at each time point. Principal component analysis of significant proteins indicated that the 2 hour treatment did not lead to distinct clustering between treatment and vehicle (DMSO) control, whereas the 24 hour treatment led to distinct clustering between HSN748 treatment and the DMSO vehicle group, showcasing the impact of the treatment in the cellular proteome (Supplemental Figure 4). Thus, we proceeded to analyze the 24 hour treatment samples. We quantified 3,597 proteins in the global-protein study and 6,599 phosphoproteins in the phosphoprotein study. HSN748 downregulated several proteins involved in translation, mRNA processing, and cell division (Figure 4). Phosphoproteomic analysis indicated that HSN748 affected several phosphorylation networks (Supplemental Figure 4). Treatment with HSN748 for 24 hours significantly altered the phosphorylation of several key cell cycle checkpoint proteins, including *INCENP*, *MCM2*, *MDC1*, *TP53BPI*, *RANBP2*, *BUB1*, and *RB1* (Supplemental Figure 5). Regulation of differential phosphorylation of essential proteins involved in the PI3K/AKT/mTOR and MAPK signaling pathways including MAPK1, AKT1, IRS2, EIF4EBP1, RPS6, RPTOR, and EIF4B was also observed upon HSN748 treatment (Supplemental Figure 6).

### Transcriptional effects of HSN748 on FLT3-mutated AML cells.

**Figure 5:**
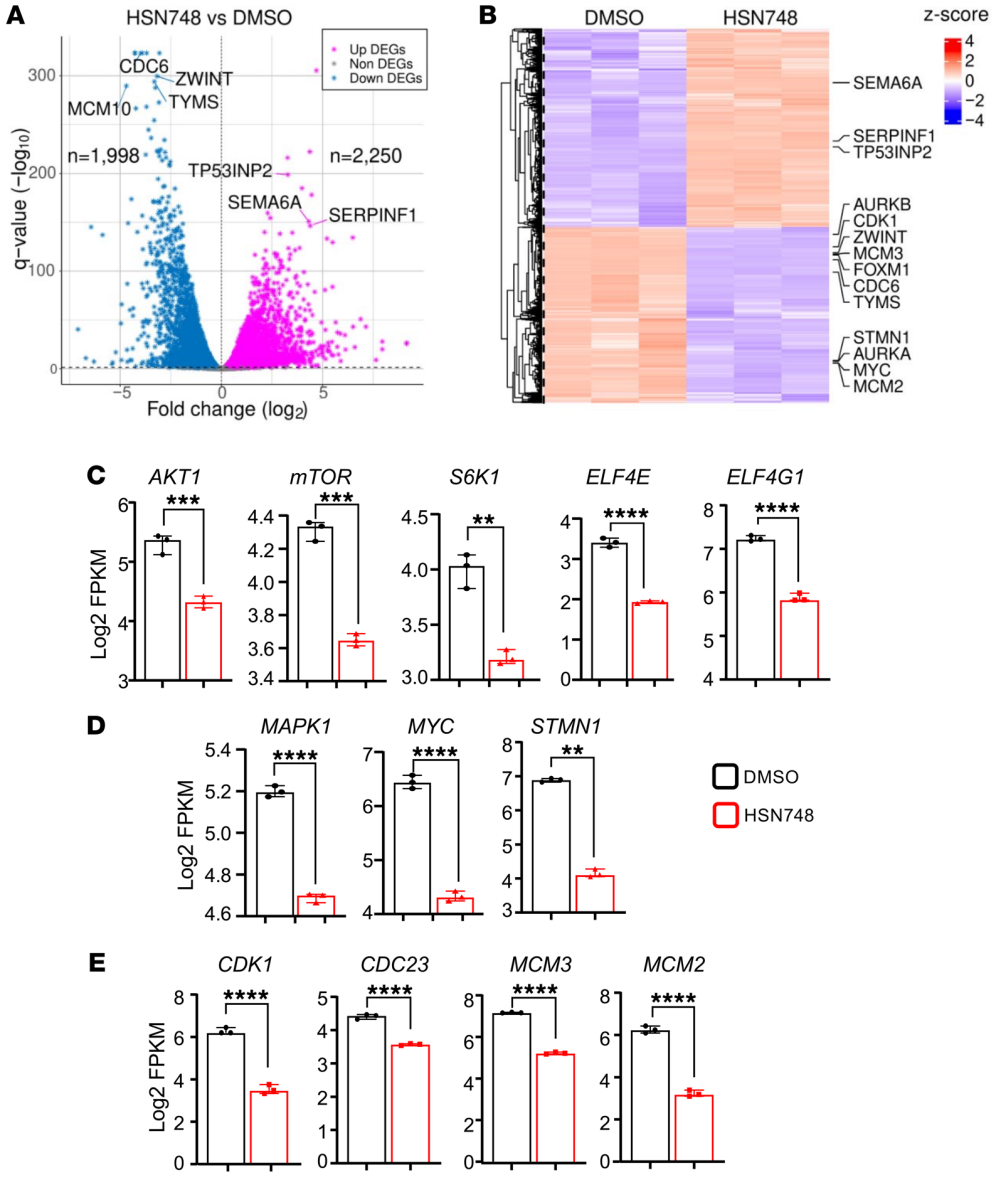
HSN748-regulated genes in *FLT3^ITD^*-bearing cells. MOLM14 cells were treated with 2 nM of Gilteritinib and HSN748 for 24 hours. RNA was extracted by Trizol reagent and subjected to RNA-Seq analysis. (**A**) Volcano plot analysis of differentially expressed genes (HSN748 versus DMSO) in MOLM14 cells. (**B**) Heat map showing differential expression of tumor suppressor, apoptosis, angiogenesis, and Gilteritinib resistance genes in response to HSN748 and DMSO treatment. Effect of HSN748 on differentially expressed genes involved in the PI3K/AKT/MTOR pathway (**C**) RAS/MAPK pathway (**D**), and the cell cycle (**E**) pathways correlating with phosphoproteomics in response to HSN748 treatment in MOLM14 cells. Y-axis showing the log_2_ FPKM values. Data represents median with interquartile range by 2-tailed Student’s *t* test (*n* = 3 in each group ****P < 0.0001, ***P < 0.001, **P < 0.01, *P < 0.05).

To determine the effect of HSN748 on FLT3-mutated AML cells with regard to gene transcription, we performed RNA-Seq analysis. We compared transcriptome data from MOLM14 cells exposed to HSN748 with those treated with DMSO after 24 hours of drug exposure, like the phosphoproteome studies. Significant changes in differential expression of genes were defined at a FDR of less than 0.05 and log FC greater than 1 between the HSN748-treated cells and DMSO controls. Volcanic plot analysis revealed 2,250 genes were upregulated and 1,998 genes were downregulated in HSN748-treated cells compared with vehicle controls (Figure 5A). Genes involved in tumor suppression, angiogenesis, proliferation, apoptosis, autophagy, and metabolic pathways were significantly altered upon treatment with HSN748 compared with vehicle controls. Some of these genes, including p53 expression, which is involved in solid tumors, were found to be altered in drug treated cells (22). *SEMA6A,* a tumor suppressor gene, was also found to be upregulated in cells treated with HSN748 compared with controls (Figure 5B). Lower expression of *SEMA6A* has been linked to lung cancer (23) and higher expression with higher overall survival in Glioblastoma (24). We also found *FOXM1* to be downregulated in HSN748-treated cells. *FOXM1* regulates survival, quiescence, and self renewal of MLL-AF9 AML cells (25), and its inactivation overcomes Venetoclax resistance in AML (26).

Thymidylate synthase (TYMS) catalyzes the reductive methylation of deoxyuridylate (dUMP) to thymidylate (dTMP), a nucleotide for DNA replication that maintains metabolic needs for cell growth (27, 28). TYMS was found to be downregulated in HNS748 treated cells compared with controls, suggesting that actively proliferating cells were inhibited by blocking the generation of nucleotides (Figure 5B). Minichromosome maintenance complex component 2 (MCM2) and MCM10 are essential for initiation of genome replication forks and form prereplication complexes with CDC6. MCM4, 2, 6, and 7 proteins with their DNA helicase activity unwind DNA (29–32). In HSN748-treated cells, significant downregulation of *CDC6* and *MCM4* and *MCM10* was seen, suggesting that the prereplication complex is disturbed, resulting in decreased replication and enhanced cell death (Figure 5B and Supplemental Figure 7). *ZWINT* plays a crucial role in the mitotic checkpoint, which ensures high fidelity of chromosome segregation, spindle checkpoint activation during mitosis, abnormalities of which contribute to hematopoietic malignancies and cell death (33, 34). The analysis in Edward et al. (35) of the TCGA pan-cancer data set revealed a greater frequency of the *ZWINT* mutation in Fanconi anemia mutant-associated cancers than in Fanconi anemia WT cancers. Recent studies by Mou et al. (36) showed *ZWINT* knockdown inhibits the proliferation and migration of A375 melanoma cells. Mechanistically, ZWINT knockdown decreases the protein expression levels of c-MYC, MTOR, phosphorylated MTOR, pp38, and fibronectin, while c-MYC overexpression reversed the effects on melanoma cell proliferation and migration. *ZWINT* was found to be downregulated in response to HSN748 treatment compared with DMSO, as was the expression of MYC; consistent with Mou et al.’s (36) studies. Importantly, genes that contribute to Gilteritinib resistance, including *AURKA* and *AURKB* were found to be downregulated in HSN748-treated cells compared with DMSO (Figure 5B). Overall, these results show that HSN748 affects the expression of genes involved in regulation of tumor suppression, proliferation, apoptosis, and angiogenesis.

### The effect of HSN748 on transcriptomics correlates with proteome expression.

To gain a deeper molecular insight into the effect of HSN748 on AML cells, we interrogated genes related to signaling pathways that are dysregulated as result of *FLT3^ITD^* mutation. Given that the PI3K/AKT, RAS/MAPK signaling pathway is activated downstream of FLT3 and plays a key role in regulation of survival, growth, and metabolism, we hypothesized that HSN748 might impact these pathways. As shown in Figure 5C, the genes related to the PI3K/AKT/mTOR (*AKT1*, *MTOR*, *S6K1*, *ELF4E*, and *ELF4G1*); RAS/MAPK (*MAPK1*, *MYC*, and *STMN1* (Figure 5D)) pathway and cell cycle genes (*CDC23* (37), *MCM3*, (38) and *MCM2* (29, 30) (Figure 5E) were found to be downregulated in response to HSN748 compared with DMSO-treated control MOLM14 cells. Consistent with gene expression data, our phosphoproteomic data also showed downregulation of phosphorylation of proteins of the above-described genes, indicating that HSN748 robustly acts both transcriptionally and translationally to reprogram the hyperproliferation and survival process resulting from dysregulated *FLT3^ITD^* signaling (Supplemental Figures 5 and 6).

Among several genes modulated by HSN748, the expression of protein arginine methyltransferases (PRMT) was substantially downregulated in response to HSN748-treated *FLT3^ITD^*-bearing AML cells as opposed to controls. *PRMT* genes, including PRMT1, 3, 5, 6, and 7 were all significantly downregulated in cells treated with HSN748. PRMTs methylate various arginine residues in critical AML genes to regulate cell proliferation and survival (39, 40). We show that HSN748 significantly inhibited the expression of PRMT 1, 3, 5, and 7 genes (Supplemental Figure 7). Metabolic reprogramming has long been recognized as one of the key characteristic feature of AML (41, 42). Creatine has been shown to be significant player in tumor cell proliferation (43). To this end, hyperactivation of STAT5 by *FLT3^ITD^* causes *FLT3^ITD^*-mutant cells to express high levels of glycine amidinotransferase, the first rate-limiting enzyme in de novo creatine synthesis. *FLT3^ITD^* inhibitors, such as AC220, Crenolanib, and Gilteritinib, reduce the viability and proliferation of *FLT3^ITD^*-positive AML cell lines by suppressing the expression of genes involved in de novo creatine biosynthesis (44). The downregulation of *PIM1*, *GATM*, *SLC7A1*, and *SLC7A2* expression in response to HSN748 compared with DMSO controls is consistent with the findings of Zhang et al. (44), suggesting that HSN748 inhibits the de novo creatine biosynthesis and contributes to the reduction in growth and survival of leukemic cells (Supplemental Figure 7).

To determine the survival probability resulting from the effect of HSN748 on gene expression, we utilized Survival Genei, a web-based platform, applying TARGET AML data sets to compare the low and high expression of genes on overall survival in AML patients (45). HSN748-treated cells showed reduced expression of key survival genes, including *AKT1*, *mTOR*, *S6K1*, *ELF4G1*, *STMN1*, *CDK1*, and *CDC23*, correlating with greater overall survival of patients with AML compared with higher expression of these genes, correlating with the survival advantage of HSN748 treatment (Supplemental Figure 8). Overall, our data suggest that the HSN748 compound is effective for the treatment of patients with AML with *FLT3^ITD^* and *FLT3^F691L^* mutations and may result in improved clinical outcomes.

### Therapeutic efficacy of HSN748 in a genetic mouse model of AML bearing a combination of epigenetic (Tet2^–/–^) and genetic (Flt3^ITD/ITD^) mutations.

**Figure 6:**
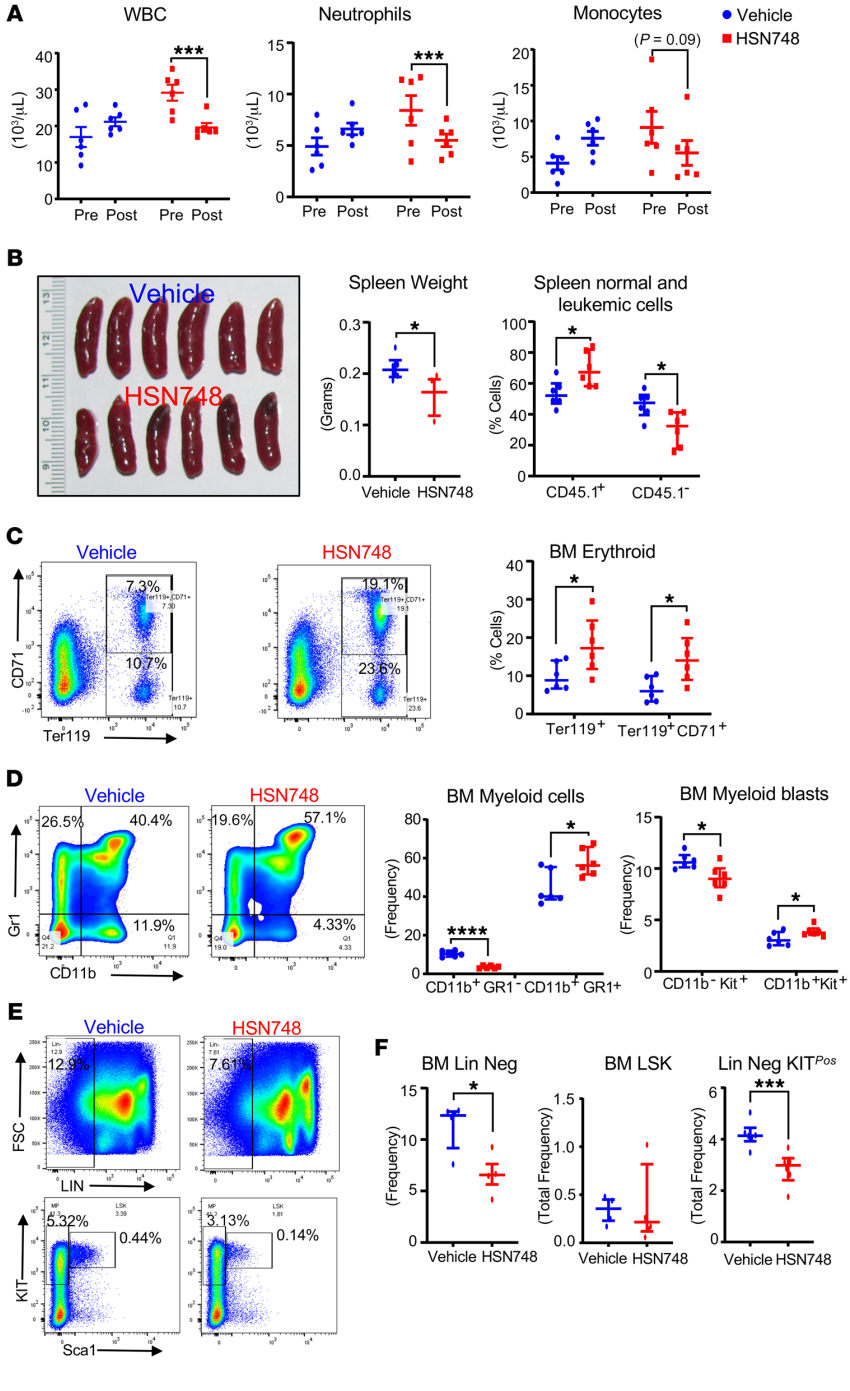
Therapeutic efficacy of HSN748 in a genetic mouse model of AML bearing a combination of epigenetic (*Tet2*) and genetic (*Flt3^ITD/ITD^*) mutation. Equal number of bone marrow mononuclear cells from CD45.1-expressing BoyJ mice and CD45.2 expressing *Tet2^–/–^:Flt3^ITD/ITD^* AML mice were mixed and transplanted to lethally irradiated F1 recipients that expresses both CD45.1 and CD45.2. 6 weeks after transplant peripheral blood engraftment was analyzed. Mice were treated orally with either vehicle or HSN748 at 20 mg/kg 5 times a week. HSN748 treatment of these mice reduces peripheral leukemic burden. (**A**) WBC, neutrophil,and monocyte counts. HSN748 treatment reduces splenomegaly. (**B**) Spleen pictures, quantification data for spleen weight in the middle panel and reduction of leukemic cells (CD45.1) with significant increase in normal cells (CD45.1^+^) in the right-side panel. (**C**) the representative flow profiles of erythroid differentiation in bone marrow from vehicle and HSN748-treated groups. HSN748 treatment induces differentiation of erythroid cells in bone marrow as demonstrated by increase in mature Ter119^+^ single-positive cells in the bone marrow of HSN748-treated mice compared with vehicle-treated group (right side panel). (**D**) shows the representative flow profiles of CD11b/Gr1 cells. HSN748 treatment induces differentiation of myeloid cells in the bone marrow as shown by increase in the frequency of mature CD11b/Gr1 double-positive cells and a decrease in the frequency of immature CD11b^–^/Gr1^+^ single-positive population compared with vehicle-treated group (middle panel) and a reduction in the frequency of immature CD11b^–^/Kit^+^ leukemic cells (right side panel). (**E**) The representative flow profiles of Lin^–^ Sca1^+^Kit^+^ (LSK) cells. (**F**) shows the quantification data of frequency of lineage negative cells (left side panel) and total frequency of lin^–^ Kit^+^ myeloid progenitors (right side panel) with insignificant effect on LSK total frequency (middle panel) on HSN748 treatment. Data represents median with interquartile range by 2-tailed Welch’s *t* test (*n* = 6 in each group ***P < 0.001, *P < 0.05).

Next, we sought to validate the antileukemic activity of HSN748 in a relevant AML mouse model carrying a combination of epigenetic mutation and *Flt3^ITD^*. We have previously generated mouse models combining mutations in *Tet2* with *Flt3^ITD^* and used them to identify novel targetable pathways (11, 46). To study the antileukemic effect of HSN748 driven by a combination of *Tet2^–/–^/Flt3^ITD^* mutation, an equal number of bone marrow mononuclear cells from CD45.2-expressing *Tet2^–/–^:Flt3^ITD/ITD^* mice (mutant/leukemic cells) were mixed with CD45.1-expressing BoyJ mice (WT cells) and transplanted into lethally irradiated F1 recipient mice, which expresses both CD45.1 and CD45.2 surface antigens. This model system specifically tracks the progression of both normal cells and leukemic cells utilizing flow cytometry to detect the presence of cell surface antigens CD45.1 or CD45.2, which are uniquely expressed only on either WT or mutant cells, respectively. Six weeks after transplant, peripheral blood engraftment was analyzed to confirm the manifestation of leukemogenesis and to establish a baseline reading before initiating drug treatment. Mice were treated orally with either vehicle or HSN748 at 20 mg/kg 5 times a week for 6 weeks and euthanized for detailed hematopoietic analysis. This strategy allowed us to assess the effect of HSN748 in parallel on both normal WT (CD45.1*^Pos^*) and leukemic (CD45.2*^Pos^*) cells. As seen in Figure 6A, peripheral blood counts before treatment show similar leukemic burden in mice as reflected by increased WBCs; HSN748 treatment significantly reduced peripheral leukemic burden, as demonstrated by decreased WBC, neutrophil, and monocyte counts 6 weeks after drug treatment. During this same period, the counts in mice treated with vehicle only kept going up. Consistent with a decrease in total WBC counts, we observed significant normalization of splenomegaly after HSN748 therapy (Figure 6B left and middle panel). The frequency of leukemic cells (CD45.1*^Neg^*) was significantly decreased upon HSN748 treatment, while the frequency of normal cells (CD45.1*^Pos^*) increased concurrently in the spleen of drug treated mice as shown in Figure 6B (right panel), suggesting the specificity of HSN748-therapeutic activity on leukemic cells as opposed to normal cells. Anemia is frequently observed in patients with AML. We and others have shown an erythroid maturation defect in the bone marrow of *Tet2^–/–^/Flt3^ITD^* AML mice leading to anemia (10, 11). In the current study, an erythroid defect observed in the bone marrow of vehicle-treated mice is in line with the published literature (Figure 6C) (11). HSN748 treatment partially restored the erythroid differentiation defect in drug treated leukemic mice as demonstrated by a significant increase in the frequency of Ter119/CD71 double–positive proerythroblasts and Ter119 single–positive mature erythroid cells compared with vehicle treated mice (Figure 6C).

Next, we assessed the effect of HSN748 treatment on terminally differentiated myeloid cells. Monocytic skewing as a result of a block in differentiation is one of the cardinal features of AML, which our transplant model system phenocopies; the frequency of myeloid cells (CD11b^+^Gr1^–^) in the bone marrow of the vehicle-treated group was increased (Figure 6D), while this was normalized by HSN748 treatment. HSN748 treatment seems to induce differentiation of myeloid cells in bone marrow, as shown by an increase in the frequency of mature CD11b/Gr1 double–positive cells compared with vehicle treated mice (Figure 6D middle panel). Importantly, HSN748 treatment induces differentiation of immature leukemic myeloid blast (CD11b^–^Kit^+^) to more differentiated mature CD11b^+^Kit^+^ double–positive cells (right panel). Figure 6D shows the representative flow profiles of CD11b/Gr1 cells. These data suggest that HSN748 exerts its antileukemic activity in part by inducing differentiation of immature myeloid lineage in addition to partly restoring erythroid lineage skewing. It is of note that normal BM cells are spared by this drug.

To gain deeper insight into the effect of HSN748 on primitive leukemic cells of the bone marrow, we gated on lineage-negative leukemic fraction and found a significant decrease in the frequency of lineage-negative leukemic cells in HSN748-treated group compared with the vehicle-treated group (Figure 6E), indicating that HSN748 treatment normalizes primitive leukemic population in addition to affecting abnormal monocytic skewing. Consistent with the effect on myeloid lineage skewing, lin*^Neg^* KIT*^Pos^* myeloid progenitors were profoundly decreased in HSN748-treated mice compared with vehicle-treated mice (Figure 6, E and F) without impacting the Lin*^Neg^* Kit*^Pos^* Sca1*^Pos^* cells (Figure 6E), suggesting HSN748 treatment partly restores differentiation of AML cells.

### HSN748 outperforms Gilteritinib in inhibiting the progression of leukemogenesis in AML patient–derived xenograft.

**Figure 7:**
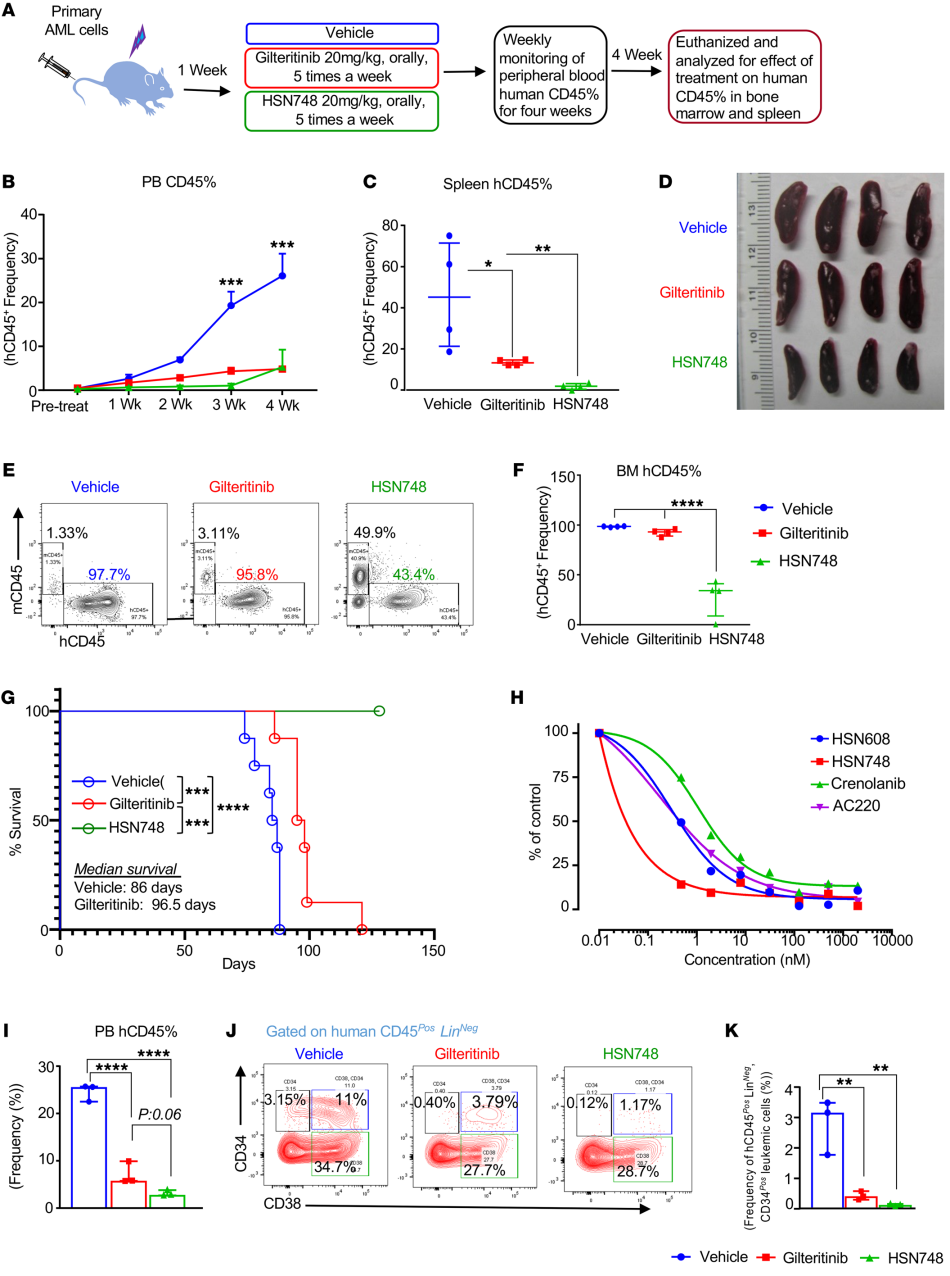
Superior growth-inhibitory effect of HSN748 compared with FDA-approved FLT3 inhibitor Gilteritinib on the development of AML in NSGS mice. Figure shows PDX data from 2 different samples from patients with AML. Data from Panel **A**–**G** originated from the first sample from a patient with AML and Panels **H**-**J** from different patient sample. (**A**) Experimental design. Briefly, multimutational (*FLT3* *^ITD^^(ins46)^*, *DNMT3A^R882H^*, *NPM1^288FS12^*, and *CHEK2*) cells from patients with AML were transplanted to sublethally irradiated (200 rads) NSGS mice. A week after transplanting peripheral blood hCD45-positive cells engraftment was assessed, and, based on the engraftment, mice were divided into vehicle, Gilteritinib, and HSN748 groups randomly and followed up on treatment for 4 weeks. After treatment, mice were euthanized and assessed for hCD45 percentage in peripheral blood, spleen,and bone marrow. **B** shows the robust inhibitory effect of HSN748 on hCD45-positive cells in peripheral blood at indicated time points. Panel **C** shows the inhibitory effect of HSN748 on hCD45% in spleen. **D** shows spleen pictures and **E** shows the representative flow profiles of human and murine CD45-positive cells in bone marrow of vehicle, Gilteritinib, and HSN748-treated mice. X-axis showing hCD45-positive cells and y-axis showing mCD45-positive cells. (**F**) Quantification data on the frequency of hCD45 cells in the bone marrow in mice treated with various drugs. (**G**) Kaplan-Meier plot showing the effect of Gilteritinib and HSN748 treatment on the survival of same-patient sample-derived xenografts in a separate experiment (*n* = 8 in each group). (**H**) Dose dependent superior proliferation inhibitory effect of HSN748 and HSN608 compared with FDA-approved FLT3 inhibitor AC220 on different samples from patients with AML with a multimutation of *DNMT3A^R882C^*, *FLT3^ITD^* *^(E598–Y599^^ins12),^^N676K,^^Y842H^, NRAS^G12D^,* and *KMT2A^MLL^ MLL^PTD^* ^(exons^ ^2–8)^ ending up relapsing profile. This patient had previously been on Gilteritinib and other kinase-inhibitor therapies. Panel **I** shows the same-patient sample-derived xenografts effect of treatment with HSN748 compared with Gilteritinib and vehicle on peripheral blood hCD45 frequency quantification. (**J**) Representative flow profiles of leukemic stem and progenitors (**K**) quantification data on the frequency of human leukemic stem and progenitors under drug treatment. Data represent median with interquartile range by ordinary 1-way ANOVA analysis. (*n* = 3–4 in each group ****P < 0.0001, ***P < 0.001, **P < 0.01, *P < 0.05). Panel **I** shows a 5.5-week time point quantitative data on the effect of HSN748 on hCD45 frequency. A representative flow profile of hCD45 frequency on biweekly peripheral blood assessment for the impact of HSN748 treatment on engraftment and propagation of leukemic cells was presented in Supplemental Figure 11.

Based on the observed antiproliferative effect of HSN748 in vitro (Figure 2C) and in vivo model systems (Figures 3 and 6), we extended our studies to further validate the effect of HSN748 on patient-derived xenografts. We compared the effect of HSN748 with a FDA approved drug Gilteritinib. As outlined in Figure 7A, we transplanted multimutational (*FLT3^ITD^* (*ins46*), *DNMT3A*, *R882H*, *NPM1*, *288FS12*, and *CHEK2*) AML patient cells into sublethally irradiated (200 rads) NOD-*scid* IL2Rgnull-3/GM/SF(NSGS) mice through tail vein injection. A week after transplantation, mice were randomly divided into vehicle, Gilteritinib, and HSN748 treatment groups. Gilteritinib and HSN748 were given orally for 5 days a week at 20 mg/kg. This dose was well tolerated, and body weight was not affected (data not shown). Weekly presence of peripheral blood human CD45 (hCD45 positivity) and murine CD45 (mCD45 positivity) cells was assessed to monitor the effect of treatment. As shown in Figure 7B, peripheral blood human CD45% in the vehicle-treated group increased every week to nearly 30% by week 4, indicating aggressive progression of disease (AML), while treatment with HSN748 and Gilteritinib showed a decline in the presence of peripheral blood human CD45-positive cells. Next, we assessed the effect of HSN748 on the presence of human CD45-positive cells in the spleen. As shown in Figure 7C, robust inhibitory effect of HSN748 was observed in the spleens of HSN748-treated mice compared with Gilteritinib and vehicle-treated mice, demonstrating that HSN748 has a broader and deeper antileukemic effect than Gilteritinib with respect to inhibition of human CD45 positive cells, which correlates with the spleen size reduction (Figure 7D). Further, to gain deeper insight into the impact of HSN748 on the bone marrow, the organ responsible for propagation of leukemic stem cells, relapse, and drug resistance, we assessed the presence of human CD45-positive cells in bone marrow of various drug-treated groups. As seen in Figure 7E, a remarkable reduction in human CD45-positive cells in bone marrow was observed in HSN748 treatment group relative to the Gilteritinib treated group. Figure 7F shows the quantification data of human CD45-positive cells from vehicle, Gilteritinib, and HSN748 treatment groups. Again, we observed a deeper and more robust response in the bone marrow of leukemic mice treated with HSN748 compared with Gilternitinib. Next, we hypothesized that the inhibitory effect of HSN748 might have a survival advantage in PDX-bearing complex multimutational AML patient-derived cells. To test this, we generated PDX from the above-described patient sample in a separate set of transplant experiments and treated with Gilteritinib and HSN748, followed by assessing survival. As shown in Figure 7G, HSN748 compound exhibited a greater survival advantage compared with the vehicle group (median survival duration of 86 days) and Gilteritinib group (96.5 days). The mice in the HSN748-treated group neither displayed moribund conditions nor succumbed to mortality upon our follow up until 128 days, when we terminated the study. Likewise, our data related to enhanced survival in AML-bearing PDXs was also noted using a completely different multimutational AML sample. Briefly, in a separate PDX experiment, utilizing multimutational (*FLT3^ITD^*, *DNMT3A*, *ASXL1*, and *NPM1)* cells from patients with AML, transplanted and drug treated in a manner described in Figure 7A, we observed similar findings of enhanced survival in the HSN-treated group relative to controls. At different time points, peripheral blood human CD45 frequency was assessed and followed up for 196 days for survival. Our data in this second PDX experiment also demonstrated a robust inhibitory effect of HSN748 on human CD45-positive cells in peripheral blood at indicated time points (Supplemental Figure 9). As shown in Supplemental Figure 9, the HSN748 compound exhibited a significant survival advantage compared with the vehicle group (median survival duration of 157 days for vehicle) and the mice in the HSN748-treated group neither displayed moribund conditions nor succumbed to mortality at this time point of the study, while all the vehicle-treated mice were found to be moribund or dead. Later, we stopped this study. Additionally, we also generated a third set of multimutational AML sample-derived PDX (# 3263 *FLT3^ITD^*, *DNMT3A*, *MLL^PTD^*) and subjected them to treatment with HSN748 to compare its effect on hCD45 with Gilteritinib and vehicle groups, as described above for the previous 2 experiments. Again, our data is consistent in terms of robust inhibition of human hCD45, as shown in Supplemental Figure 10, along with remarkable reduction in splenomegaly.

Next, in a separate experiment, we validated the antiproliferative efficacy of HSN608 and HSN748 on a different AML patient-derived bone marrow sample (Figure 7H). We compared the growth inhibitory effect of HSN608 and HSN748 with AC220 and Crenolanib on AML patient-derived PDX. This patient was treated with Gilteritinib but relapsed and presented with a multimutational complex genotype including *DNMT3A* (R882C), *FLT3* (ITD (E598-Y599 ins12), *N676K*, *Y842H*, *NRAS* (*G12D*), and *KMT2A* (*MLL*) *MLL-PTD* (exons 2–8). Recently, emergence of *RAS* mutations in patients with AML who were treated with Gilteritinib has been reported (2). As shown in Figure 7H, dose dependent superior inhibitory effect was observed in these cells in the presence of HSN748 followed by HSN608 and AC220, suggesting HSN748 demonstrates a more potent antiproliferative effect not only in the BaF3 cell line system but also in a complex relapsed multimutational AML patient-derived bone marrow cells in vivo as well as in vitro. To assess the effect of HSN748 on the growth and survival of leukemic stem cells, we transplanted the above sample from the patient with AML (bearing the *NRAS* (*G12D*) mutation) in NSGS mice, as described in Figure 7A. To expand these cells further, we retransplanted them into secondary NSGS mice and drug treatment was initiated after 8 weeks of transplantation. Biweekly peripheral blood human CD45-positive cells and murine CD45-positive cells were assessed by flow cytometry to monitor the impact of drug treatment on engraftment and propagation of these leukemic cells (Supplemental Figure 11), and quantitative data at a 5.5-week time point in Figure 7I shows greater inhibition of human CD45 leukemic cells upon HSN748 treatment compared with vehicle and Gilteritinib treatment. We also observed a remarkable reduction in primitive leukemic cells (CD45*^Pos^*, Lin*^Neg^*, CD38*^Neg^*, and CD34*^Pos^*) by further gating on human CD45 cells to get a deeper insight upon HSN748 treatment (Figure 7J). Figure 7K shows quantitative data for CD45*^Pos^*, Lin*^Neg^*, CD38*^Neg^*, and CD34*^Pos^* leukemic cells. Overall, our PDX data from 5 different PDX experiments shows efficacy of HSN748 for AML treatment.

## Discussion

In the current study, we present what is, to our knowledge, a novel class of nicotinamide-based FLT3 inhibitors that target *FLT3^ITD^* at subnanomolar dosages and are effective against drug-resistant secondary mutations of *FLT3^ITD^*. We evaluated HSN608 and HSN748 on primary de novo AMLs, drug-resistant AMLs, and relapsed/refractory AMLs with *FLT3^ITD^* mutations, as well as in vivo genetic models including mice bearing *Tet2 and FLT3^ITD^* and cells from AML patient-derived PDXs. We found that the growth inhibitory impacts of HSN748 and HSN608 were more potent than those of FDA-approved FLT3 inhibitors including AC220, Gilteritinib, Crenolanib, Midostaurin, Sorafenib, and Ponatinib on murine BaF3 cells expressing *FLT3^ITD^* and *FLT3^ITD/F691L^* (gatekeeper mutation) (Figure 2). HSN748 showed a superior inhibitory effect on tumor growth and survival of MOLM14 and MOLM14-*FLT3^ITD/F691L^-*recipient mice compared with Gilteritinib-treated mice (Figure 3). In an AML-relevant mouse model carrying a combination of epigenetic mutation *Tet2* and *Flt3^ITD^*, the in vivo efficacy of HSN748 was found to be quite potent. Oral administration of HSN748 significantly reduced peripheral leukemic burden, normalized splenomegaly, decreased the frequency of leukemic cells, corrected the erythroid defect, and induced differentiation of immature leukemic myeloid blast (CD11b-Kit^+^) cells into more differentiated mature CD11b^+^Kit^+^ double positive cells (Figure 6).

Hematopoietic cells rely on the PI3K-AKT-MTOR pathway for fundamental processes including proliferation, differentiation, and survival. Sixty percent of patients with AML demonstrate constitutive activation of PI3K-AKT-MTOR pathway and this is linked to a shorter overall survival (47, 48). Given that *FLT3^ITD^* dysregulates PI3K-AKT-MTOR and RAS/MAPK pathway, affecting growth and survival functions (49), we hypothesized that inhibiting these pathways with HSN748 might improve the overall survival. In the current study, our RNA-Seq data show that genes involved in the PI3K/AKT/MTOR (*AKT1*, *MTOR*, *S6K1*, *ELF4E*, and *ELF4G1*), RAS/MAPK (*RAF1*, *STK3*, *MAPK1*, *MYC*, and *STMN1*) and Cell cycle pathway (*CDK1*, *CDC23*, *MCM2*, and *MCM3*) are downregulated upon HSN748 treatment. This is in consistent with the downregulation of phosphorylation of these kinases as seen in our phosphoproteomic data indicating that HSN748 acts both transcriptionally and translationally to reprogram hyperproliferation and survival of cells bearing *FLT3^ITD^* (Figure 5).

Emerging evidence on the pattern of drug resistance shows that FLT3-mutated AMLs treated with type I versus type II FLT3 inhibitors manifest different resistance mechanisms (2, 50). Patients treated with type I FLT3 inhibitors are more likely to develop RAS/MAPK pathway mutations than those treated with type II FLT3 inhibitors (2, 50). Interestingly, 37% of patients with AML who failed Gilteritinib therapy exhibited RAS/MAPK mutations as well as the emergence of additional new mutations (including *BRAF*, *CBL*, *KRAS*, *PTPN11*, *RUNX1*, *WT1*, and *CEBPA*), which were undetected prior to Gilteritinib treatment (2). Crenolanib (Type 1, FLT3 inhibitor) monotherapy has shown therapeutic improvement in patients with relapsed/refractory AML, but responses to this drug have been transient, and relapse is frequent (5). Whole exome sequencing of samples from patients with AML before and after crenolanib treatment revealed emergence of *TET2* and *IDH1* mutations to cooccur with FLT3-mutant clones (5). In the current study, we found that HSN748 treatment of *RAS* mutant–bearing AML mice increased the survival of these mice, possibly by inhibiting the RAS/MAPK pathway and downregulating *PTPN11*, *IDH2*, and *WT1* genes, indicating the potential of HSN748 compound to eliminate the development of resistant subclones (Figure 3 and Supplemental Figure 7).

Patients with high leukemic stem cell (LSC) burden have substantially poorer relapse-free survival rates than those with lower LSC proportions (51). Patients with AML who relapse shortly after reaching complete remission and patients who respond initially and later relapse may be attributable to the presence of dormant, chemotherapy-resistant leukemic stem cells (52, 53). In the current study, we demonstrate that HSN748 has a profound inhibitory effect on the progression of disease and the content of leukemic cells (Lin Neg CD34^+^, CD38^–^), suggesting the potential of this compound in preventing relapse (Figure 7). As a potential mechanism of FLT3 inhibitor resistance, Traer et al. (54) proposed that drug-induced stromal stress via release of fibroblast growth factor 2 (FGF2) and RAS-MAPK pathway activation results in increased FGF2 production. Recent comprehensive analysis of microenvironment-mediated resistance to Gilteritinib in FLT3 mutant cell lines were found to express greater upregulation of *KRAS*, *NRAS*, *PTPN11*, *STAG2*, *SRSF2*, *AURKA*, *AURKB*, *CDK2*, *CDK4*, *ATR*, and *CDC7* genes (55). In the current study, in response to treatment with HSN748, most of the Gilteritinib-resistant genes described in Joshi et al. (55), including *PTPN11*, *AURKA*, *AURKB*, *CDK2*, *CDK4*, *CDC7*, and *ATR* were found to be significantly downregulated, suggesting that the HSN748 (Figure 5 and Supplemental Figure 7) compound will be likely more effective than gilteritinib in terms of not only halting the expansion of leukemic cells but also preventing the development of resistance/relapse.

Overall, our data suggest that HSN748 has superior efficacy by our rigorous therapeutic validation using an in vitro BaF3 cell line system, preclinical transplant model system, and patient-derived xenografts. We believe that clinical development of HSN748 would provide additional treatment options for patients with relapsed refractory AML harboring FLT3 mutations. The process of clinical development of this drug is currently ongoing.

## Methods

### Sex as a biological variable.

We utilized both male and female mice in our study and observed comparable findings.

### Cell culture and in vitro drug studies.

MOLM14 and MV411 cells were purchased from ATCC. MOLM14-*FLT3^F691L^* cells were a gift from Neil Shah (UCSF School of Medicine, San Francisco, California, USA). MV411-luc cells were a gift from Sharyn Baker (The Ohio State University College of Pharmacy, Columbus, Ohio, USA). All cells were grown in RPMI supplemented with 10% FBS. One day prior to drug addition, 5 × 10^3^ cells/well were plated in a 96-well plate. The next morning, HSN748 was serially diluted in DMSO, added to RPMI media and then added to cells. The assay was terminated 72 hours later with water soluble tertrazolium (WST-1; Clontech) and plates were read after an additional 4-hour incubation at 37°C using BioTeK HT plate reader (BioTek). Data were analyzed and graphed using GraphPad Prism Software (GraphPad). IC_50_s were calculated on GraphPad.

BaF3-*FLT3^ITD^*, BaF3-ITD-*FLT3^F691L^*, and BaF3-ITD-*FLT3^D835Y^* cells were washed 3 times with plain RPMI1640 media and resuspended in 10% FBS and 2% pen strep containing RPMI-1640 media. Cells with greater than 95% viability were used in the assay. Cells were plated into 96-well tissue culture plates at a density of 2 × 10^5^ cells/mL, with 50 μL/well of cell suspension and 50 μL/well of either media or compound of interest in quadruplets. Stock solution of compounds were prepared in 10 mM DMSO,except for Gilteritinib, which was at a 2 mM concentration. Compounds were serially diluted in media before plating in cell culture. The assay was terminated 52 to 56 hours later with the addition of water-soluble tertrazolium reagent WST-1 (Roche diagnostics, Cat 11644807001) to cultures and incubated for further 4 hours and the absorbance was measured following manufactures recommendations. The absorbance data was fitted to a nonlinear regression equation to derive IC_50_ values.

### In vitro kinase binding assays.

In vitro kinase assays were performed at DiscoverX. Briefly, compounds that bind the kinase active site prevent kinase binding to an immobilized ligand and will reduce the amount of kinase captured on solid support. Conversely, test molecules that do not bind the kinase have no effect on the amount of kinase captured on the solid support. Kinases used in the panel are tagged with DNA barcodes. Screening “hits” are identified by measuring the amount of kinase still bound to the immobilized ligand after compound treatment by using a quantitative, precise, and ultra-sensitive qPCR method that detects the associated DNA label. In a similar manner, KDs for test compound–kinase interactions were calculated by measuring the amount of kinase captured on the solid support as a function of the test compound concentration. Residence time determination assays were conducted by Enzymlogic.

### Western blotting.

Whole cell lysates were prepared in RIPA buffer with protease and phosphatase inhibitors. Proteins were resolved on 4%–12% BisTris gels and transferred on PVDF membranes, blocked with SuperBlock (TBS) Blocking Buffer and probed with pFLT3 (Cell Signaling Technology, 3461), pSTAT5 (Cell Signaling Technology, 9359), pERK (Cell Signaling Technology, 4370), ERK 1/2 (Santa Cruz, sc514302). Membranes were reprobed with total FLT3 antibody 3462 (Cell Signaling Technology) and GAPDH-HRP (Cell Signaling Technology 884).

### Experimental mice.

Mice were housed in pathogen-free settings at the Indiana University Laboratory Animal Research Center, Indianapolis, Indiana. *Flt3^ITD^* mice were crossed with *Tet2^–/–^* mice to produce double mutants of *Tet2^–/–^*: *Flt3^ITD^* mice. WT (C57BL/6) and F1 mice expressing both CD45.1 and CD45.2 were procured from Indiana University’s in vivo Core facility.

### In vivo drug studies.

Female NRG (NOD-*Rag1^null^* *IL2rg^null^*) mice were injected with 5 × 10^5^ MOLM14 or MOLM14-F691L cells that had been previously transduced with a luciferase reporter. Mice were imaged 3 days later and sorted into 5 mice per group so that luminescence (as a surrogate marker for disease burden) was equal. Dosing started the same day and continued once daily, 5 days/week. Mice were imaged for efficacy once per week and body weight recorded daily. Mice were euthanized when 20% body weight loss, hind limb paralysis, or lack of ability to eat/drink (moribund) were noted. For pharmacokinetic measurements, CD1 mice (3 per group) were treated with 1 dose of HSN748.HCl administered either intravenously (in PBS) or by oral gavage (methylcellulose, 0.1M citrate buffer). Blood samples were collected at indicated time points, and levels of HNS748 in plasma were quantified by LC/MS. Western blots of tumors resected from animals after dosing was performed as described above.

### Drug treatment for proteomics experiment.

For MOLM14 proteomics, T-75 flasks (Corning) were seeded with 10 × 10^6^ cells in 30 mL of media and incubated overnight. Biologically independent triplicates (*n* = 3) were treated with HSN748 dissolved in DMSO for a final concentration of 100 nM (10 μL of 0.3mM HSN748) or with 10 μL of DMSO control (Thermo Fisher Scientific). All conditions contained the same amount of DMSO. Cells were collected following 2 hours and 24 hours of treatment. Cells were washed 3 times with PBS via centrifugation at 4°C, and the cell pellet was frozen at –80°C. All LC-MS raw data files were deposited to MassIVE data repository (massive.ucd.edu) under submission ID MSV000090855.

### RNA-Seq analysis.

MOLM14 cells were seeded with 0.2 × 10^6^ cells/mL for 24 hours in the presence of 2 nM HSN748 and DMSO in 24-well plate. Biologically independent triplicates were treated with HSN748 and DMSO controls. Cells were harvested and washed 2 times with PBS and resuspended in trizol reagent and the cell pellet was frozen at –80° until use. RNA was extracted and sequenced as described earlier (46). The raw data file for RNA-Seq has been deposited in GEO with accession code GSE221459.

### Flow cytometry.

Bone marrow and spleen single-cell suspension was prepared for flow cytometry analysis as described earlier (46, 56). Flow cytometric analysis was performed on a multiparameter analyzer 5 laser LSRII with Diva software (BD Biosciences) and the data were analyzed using FlowJo software. The data were analyzed using FlowJo software. Detailed information related to docking calculation, molecular dynamics simulations, proteomics sample preparation, and mass spectrometry and their analyses are described in Supplemental Methods.

### Statistics.

Statistical analyses were performed using GraphPad Prism software (Version 7 and 10). Results are expressed as mean ± SEM for Figure 3. The significance of the differences was analyzed by a 2-tailed Student’s *t* test (Figure 5), Welch’s *t* test (Figure 6), and by ordinary 1-way ANOVA analysis (Figure 7). Results are expressed as median with interquartile range (Figures 6 and 7). A comparison of survival curves between the control and drug-treated groups was performed by applying the Log-rank (Mentel-Cox) test for Figures 2 and 7. *P* ≤0.05 was considered significant.

### Study approval.

Animal studies presented were approved by Indiana University Laboratory Animal Resource Center and these animals were maintained in pathogen free facility at Indiana University School of Medicine, Indianapolis. The Indiana University School of Medicine’s IACUC approved all animal procedures, which were carried out in accordance with the National Academies Press 2011 publication, Guide for the Care and Use of Laboratory Animals. Patients provided written informed consent prior to sample collection. Indiana University has granted ethical approval for the collection of patient samples (protocol no. 1011003088).

### Data availability.

Raw data file for RNA-Seq has been deposited in GEO with accession code GSE221459. All LC-MS raw data files were deposited to MassIVE data repository (massive.ucd.edu) under submission ID MSV000090855. All the data used to generate graphs are provided in the Supporting Data Values file.

## Author contributions

HOS, R Kapur, GC, MJA, AE, LSH, RL, and FWH designed and supervised the project. ND and EL synthesized compounds. BR and ND, designed and executed the experiments and analyzed and wrote the manuscript. RP initiated the project, designed and executed the experiments, and analyzed the data. CK, SKP and R Kanumuri assisted with experiments. SL and JW contributed to RNA-Seq analysis. GC designed and supervised the computational work on molecular docking and molecular dynamics simulations that were performed and analyzed by SV, ERFYC, RM, and UKA contributed to proteomics. All authors reviewed and revised the manuscript.

## Supplementary Material

Supplemental data

Unedited blot and gel images

Supporting data values

## Figures and Tables

**Table 1:**
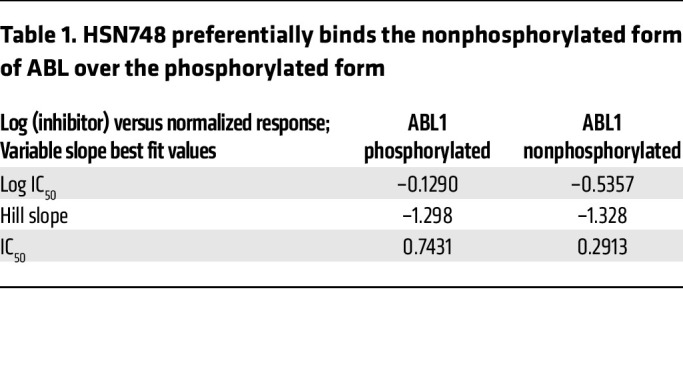
HSN748 preferentially binds the nonphosphorylated form of ABL over the phosphorylated form

**Table 2:**
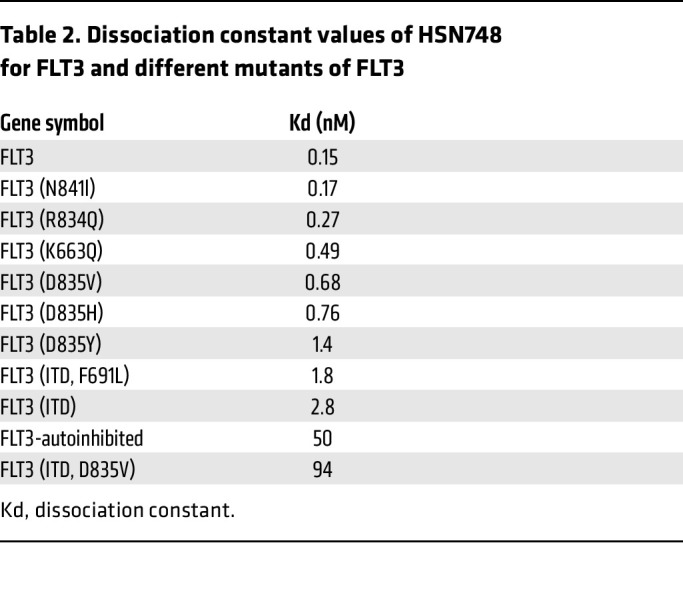
Dissociation constant values of HSN748 for FLT3 and different mutants of FLT3

## Version 1 06/17/2024

Electronic publication
