# Supplementary material for: Alkynyl nicotinamides show antileukemic activity in drug-resistant acute myeloid leukemia
Source: J Clin Invest. 2024 Jun 17;134(12):e169245. doi: 10.1172/JCI169245 (PMC11178545; doi:10.1172/JCI169245)
Supplement: Supplemental data [file jci-134-169245-s276.pdf]

Supplemental Information

Alkynyl nicotinamides show antileukemic activity in drug-resistant acute myeloid leukemia

Baskar Ramdas[1§], Neetu Dayal[5§], Ruchi Pandey[1§], Elizabeth Larocque[2], Rahul Kanumuri[1], Santhosh Kumar Pasupuleti[1], Sheng Liu[3], Chrysi Kanellopoulou[4], Elizabeth Fei Yin. Chu[2], Rodrigo Mohallem[5], Saniya Virani[2], Gaurav Chopra[2,6], Uma K. Aryal[5,7], Rena Lapidus[4,8], Jun Wan[3], Ashkan Emadi[4,8], Laura S. Haneline[1*], Frederick W Holtsberg[4*], M Javad Aman[4], Herman O Sintim[*2,4,6], Reuben Kapur[*1,3,9,10]

Supplemental Table 1

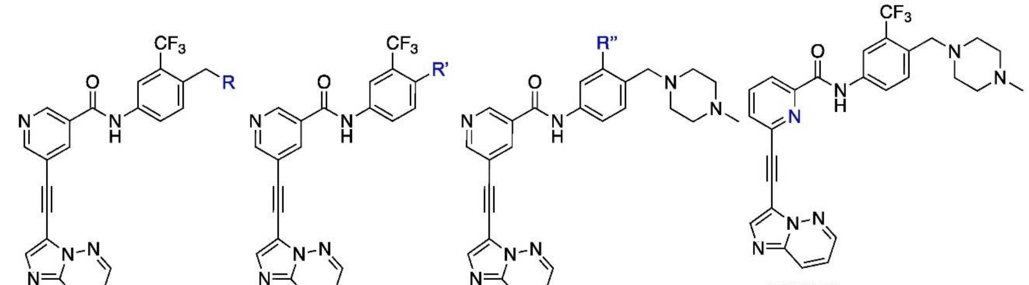

HSND23

Compound	R	R'	R''
HSND01	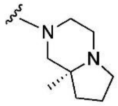		
HSND02	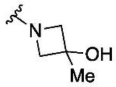		
HSND05	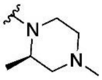		
HSND06	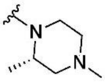		
HSND07	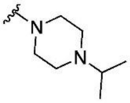		
HSND08	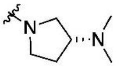		
HSND09	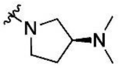		
HSND10	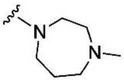		
HSND12	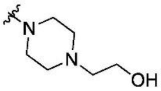		
HSND11		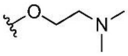	
HSND13			Cl

Supplemental Table 2

Compound	MOLM14 IC_{50} (nM)	D835Y IC_{50} (nM)	F691L IC_{50} (nM)	MV4-11 IC_{50} (nM)
HSND01	1.17	64.05	32.6	0.306
HSND02	56.7	>100	>100	42.35
HSND05	0.012	19.73	2.57	0.15
HSND06	0.097	11.45	1.65	0.18
HSND07	0.283	89.4	3.03	0.42
HSND08	0.129	>50	3.15	0.145
HSND09	0.65	280.5	23.95	0.7
HSND10	0.165	111.25	3.2	0.23
HSND11	3.1	ND	3.88	0.175
HSND12	0.175	>100	8.75	0.675
HSND13	0.9	180.5	38.8	0.165
HSND23	27.35	>200	>100	>50
HSN748	0.125	5.066	1.95	0.065
Ponatinib	0.9	93.5	9.96	0.382
Gilteritinib	1.22	3	3.65	0.29

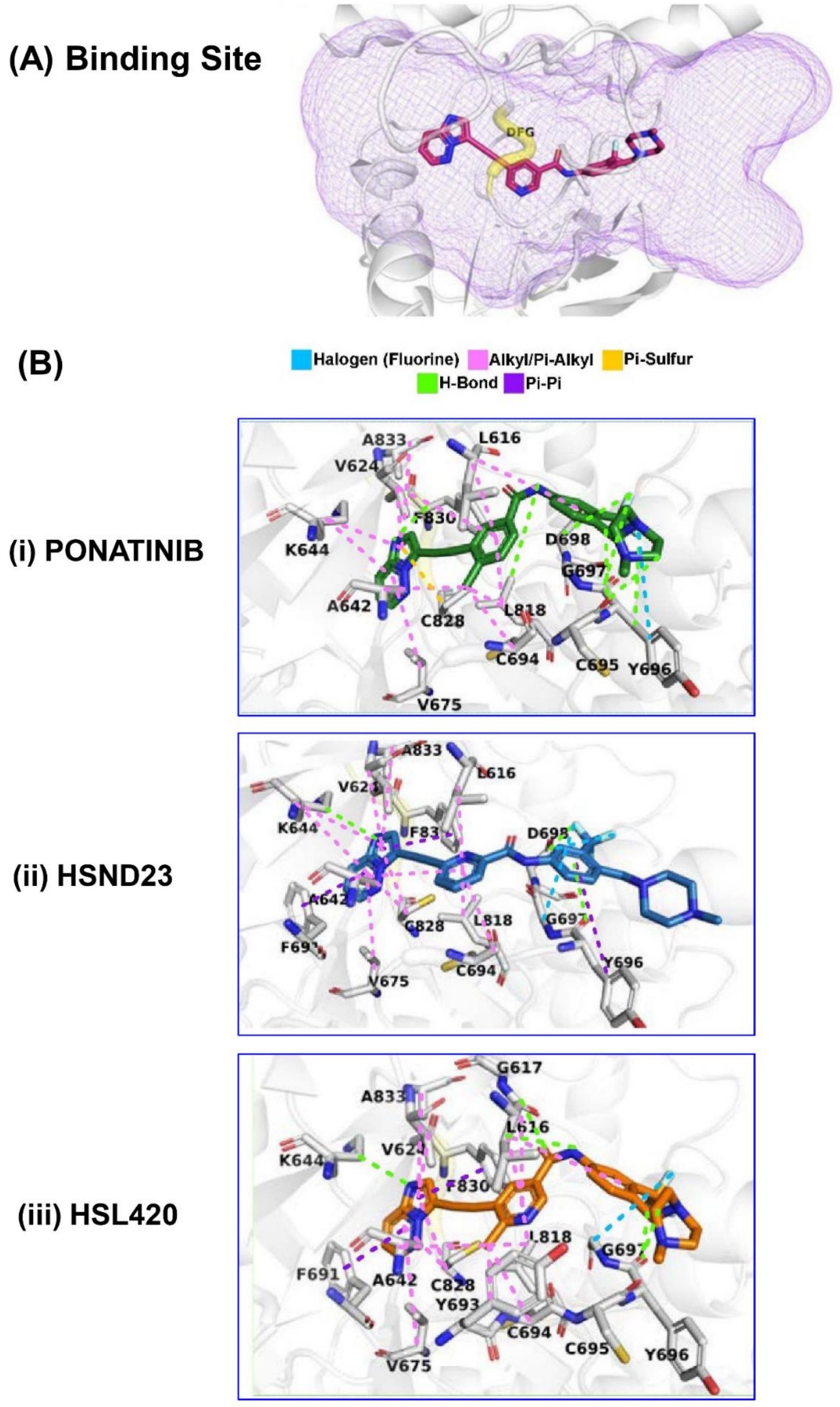

Supplementary Figure 1 (A) Binding site grid (purple) with HSN748 and DFG motif shown in yellow (B) Top docking poses for (i) Ponatinib, (ii) HSND23, and (iii) HSL420 showing Florine interactions (blue), hydrogen bonds (green), Alkyl/Pi-Alkyl interactions (pink), pi-Sulfur (yellow) and pi-pi interactions (purple) interactions in the binding pocket.

A

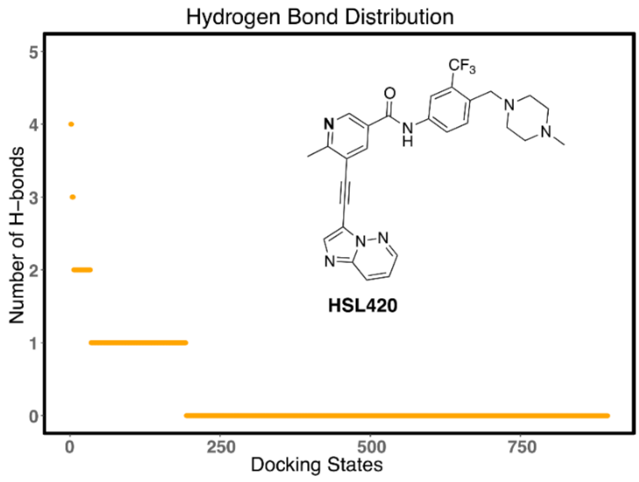

B

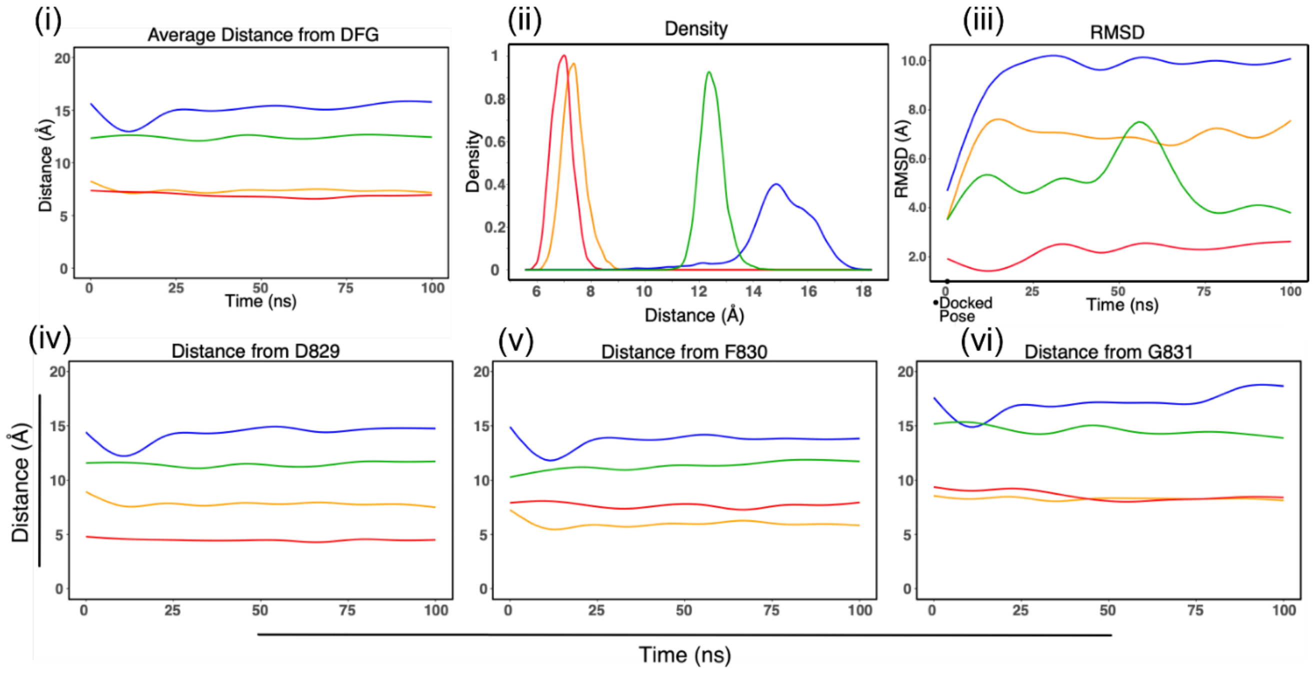

Supplementary Figure 2. Plots with HSN420 (orange), HSN748 (red), HSND23 (blue), and Ponatinib (green). (A) Hydrogen bond distribution for HSL420. (B) (i) Average Distance Plot from DFG motif. (ii) Probability density distance plot, (iii) RMSD plot of protein backbone and ligand, and (iv-vi); Distance Plot for all 4 compounds from residue D829, F830 and G831, respectively.

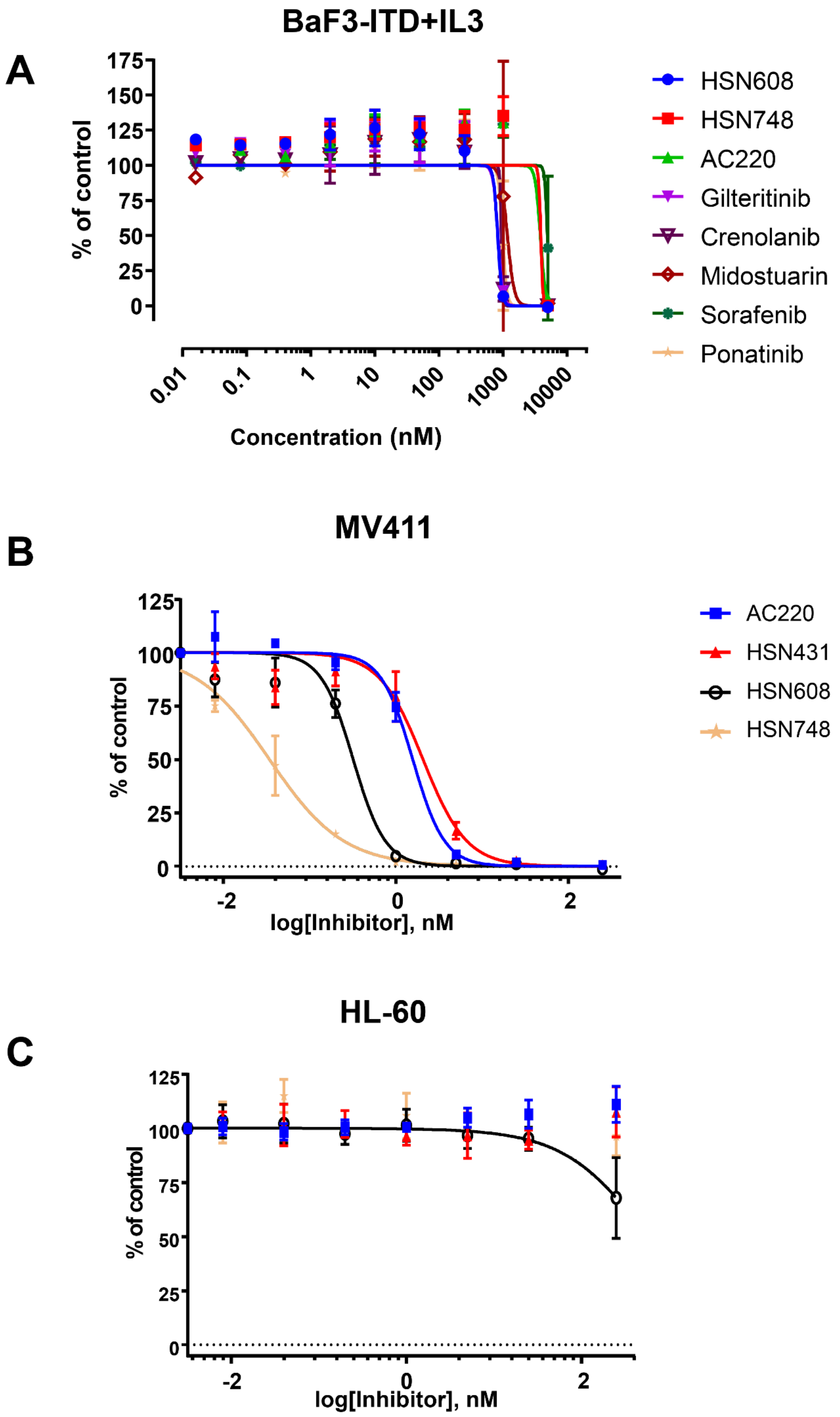

Supplementary Figure 3. Efficacy of HSN608 and HSN748 compared to the FDA approved FLT3 inhibitors in the presence of growth factor IL3. Murine BaF3 cells transduced with *Flt3*$^{ITD/ITD}$ expressing retroviral vector were cultured in the presence of IL3 growth factor along with the indicated concentrations of the inhibitors for 48 hours and proliferation was estimated by colorimetric assay. Human AML cell lines (B) MV411 with *Flt3*$^{ITD/ITD}$ or (C) HL60 without *Flt3*$^{ITD/ITD}$ were cultured with serial dilutions of the indicated inhibitors for 48h and proliferation was estimated by colorimetric assay.

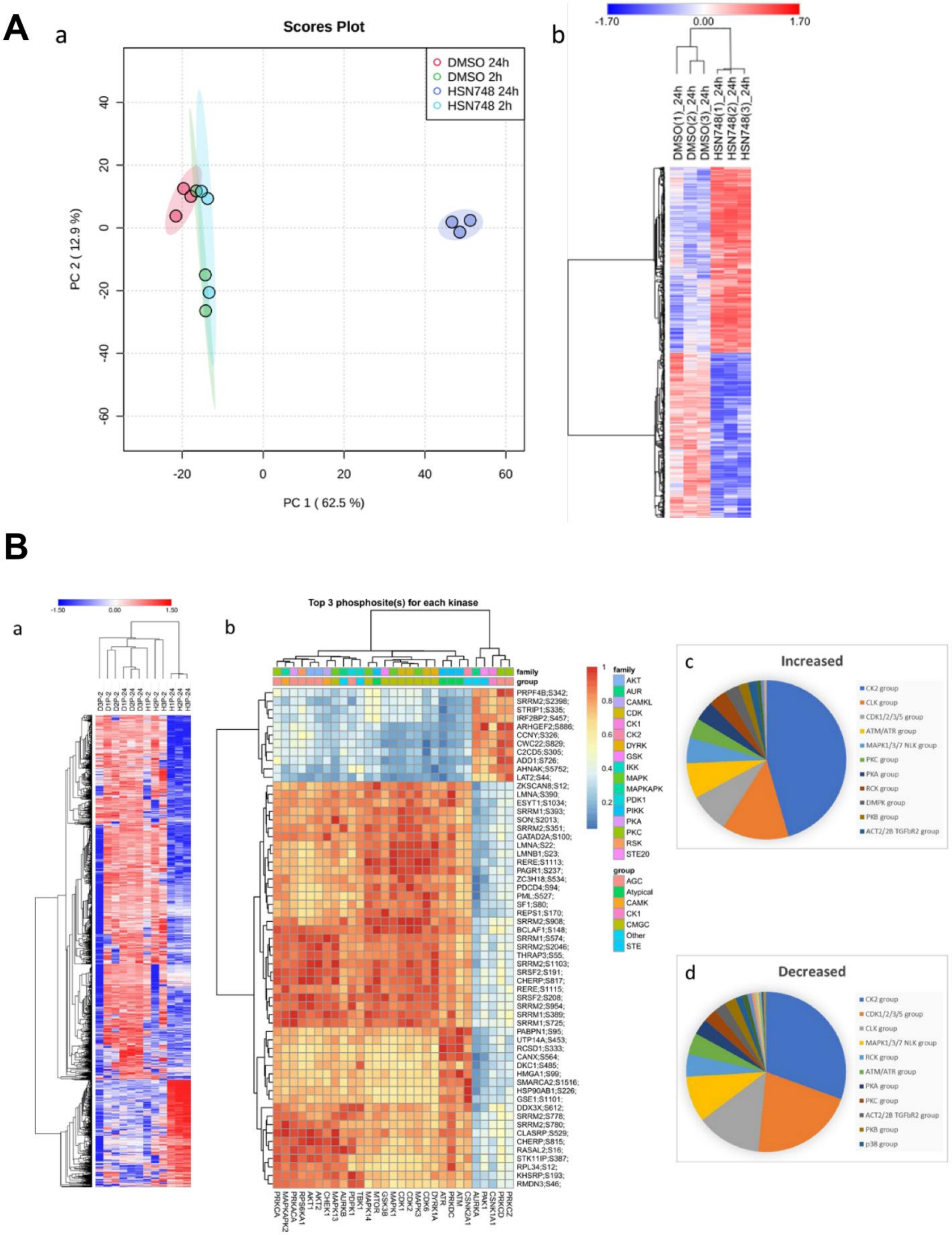

Supplementary Figure 4: Effect of HSN748 on the proteome and phosphoproteomic A (a). Principal component analysis of significant proteins. The explained variances are shown in brackets. (b) Hierarchical clustering of z-score values of significant proteins from 24h time point. B (a) Shows the hierarchical clustering of z-score values of significantly detected phosphoproteins generated with Morpheus. (b). PhosR phosphosite clustered heatmap indicating combined kinase-substrate score for top three phosphosite of x-axis kinases. Enriched kinase groups in phosphopeptides generated using NetPhorest: (c) Increased: $\log_2$ fold change > 1. (d) Decreased: $\log_2$ fold change < -1.

A

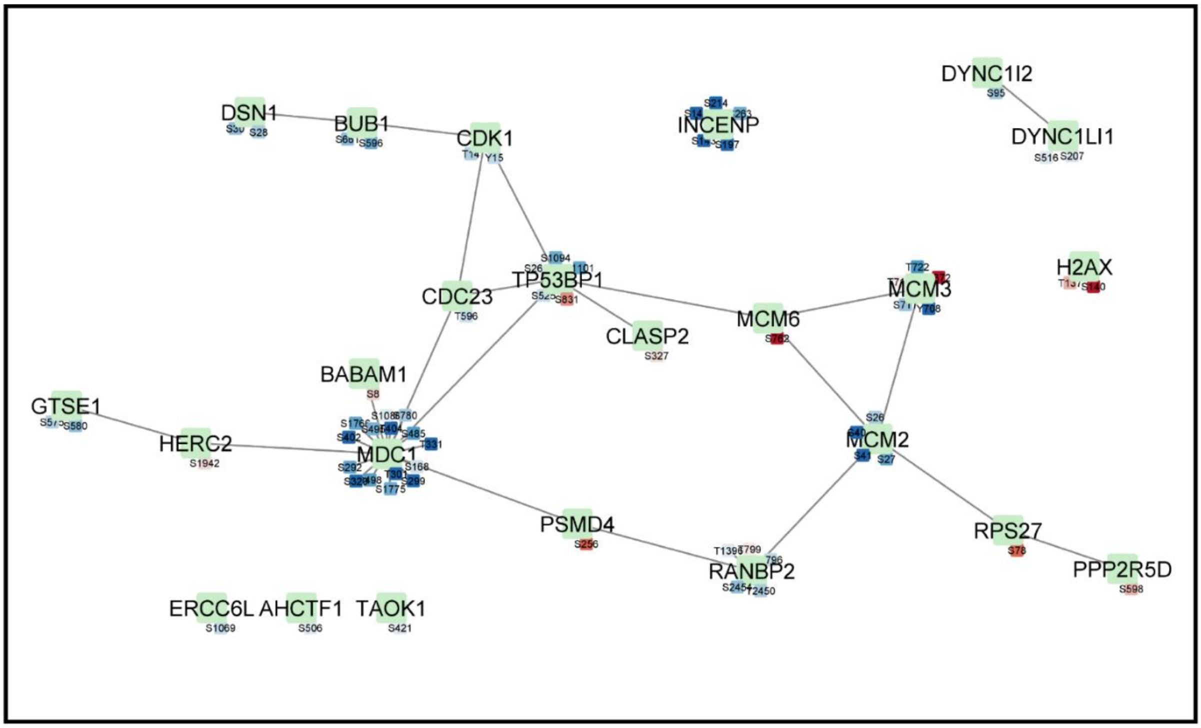

B

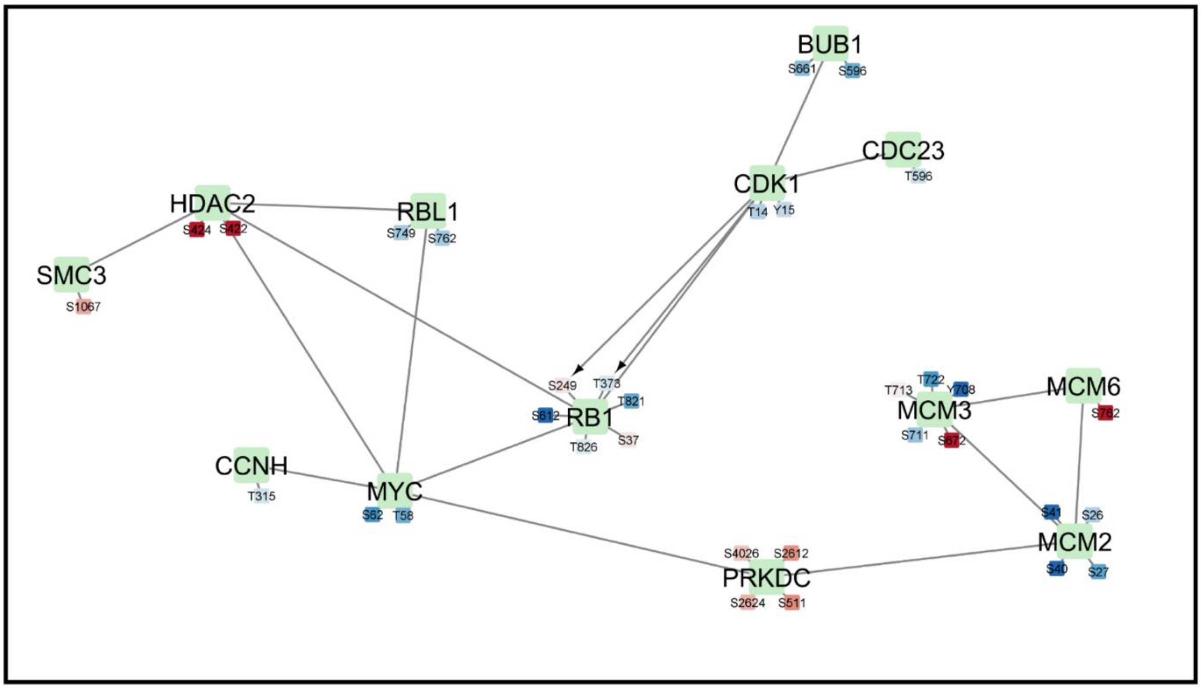

Supplementary Figure 5: The effect of HSN748 on the cell cycle related phosphorylation networks A. Cell cycle checkpoint pathway map B. Cell cycle pathway map. Red indicates upregulation and blue indicates downregulation.

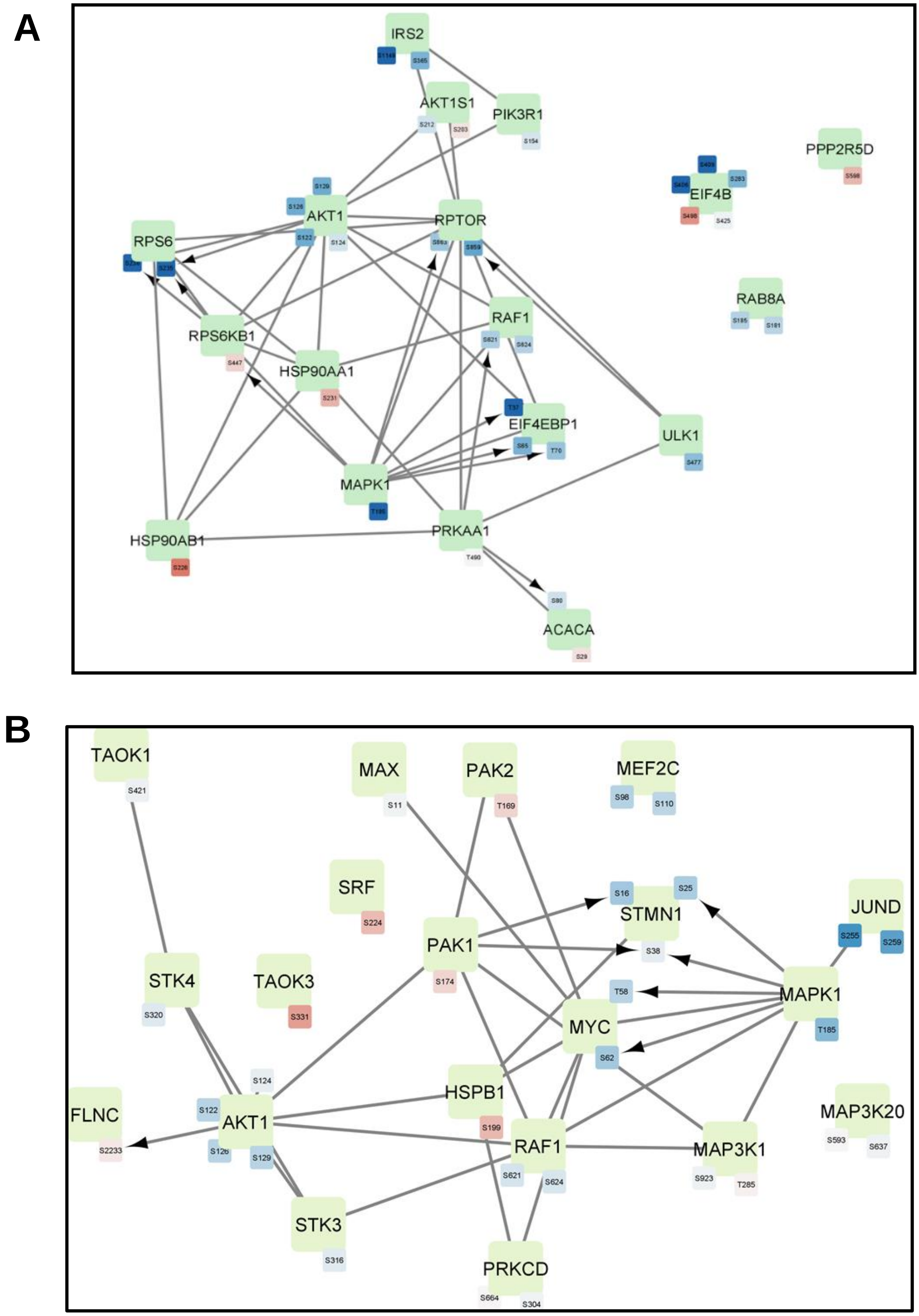

Supplementary Figure 6: The effect of HSN748 on phosphorylation pathways**.**

A. PI3K/Akt/mTOR signaling pathway B. MAPK signaling pathway. Red indicates upregulation and blue indicates downregulation.

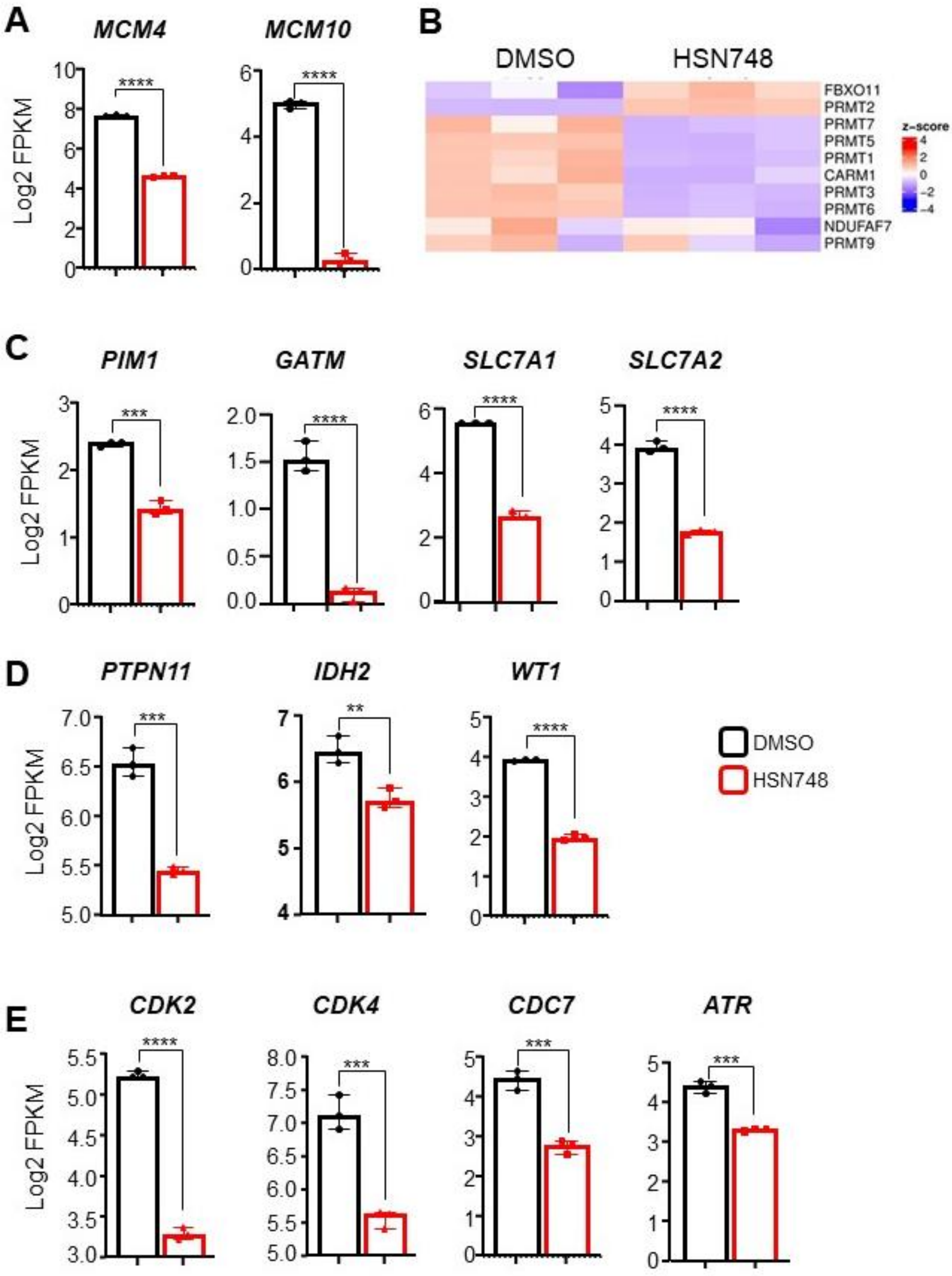

Supplementary Figure 7 The effect of HSN748 on key regulatory genes involved in different functional pathways of *FLT3*[ITD] signaling. (A) Cell cycle; (B) Arginine methylation; (C) Creatine synthesis; (D) Effect of HSN748 on Gilteritinib treatment relapsing genes (E) Effect of HSN748 on the Gilteritinib resistant genes .Data represents median with interquartile range by 2 tailed student`s t test. (N=3 in each group ****p<0.0001, ***p<0.001, **p<0.01)

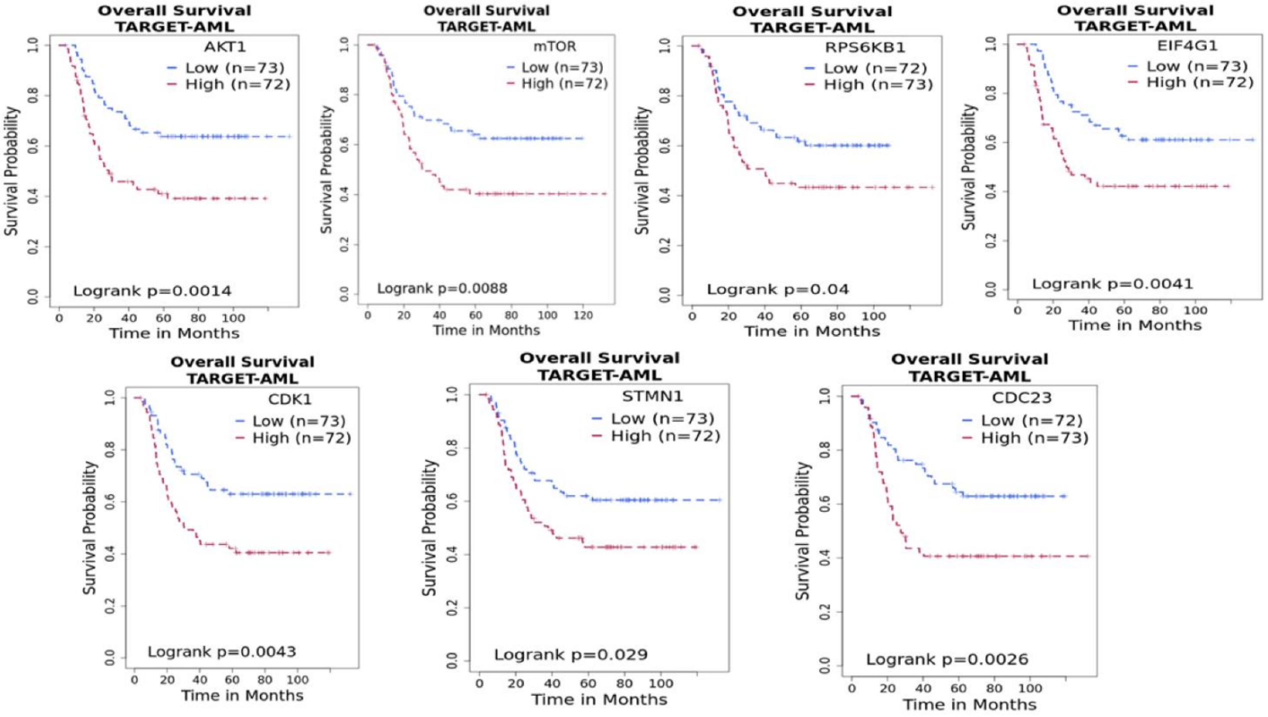

Supplementary Figure 8. The effect of HSN748 on the expression of key genes involved in the overall survival of AML patients. Reduced expression of key survival genes as a result of HSN748 correlated with the greater overall survival probability of AML patients.

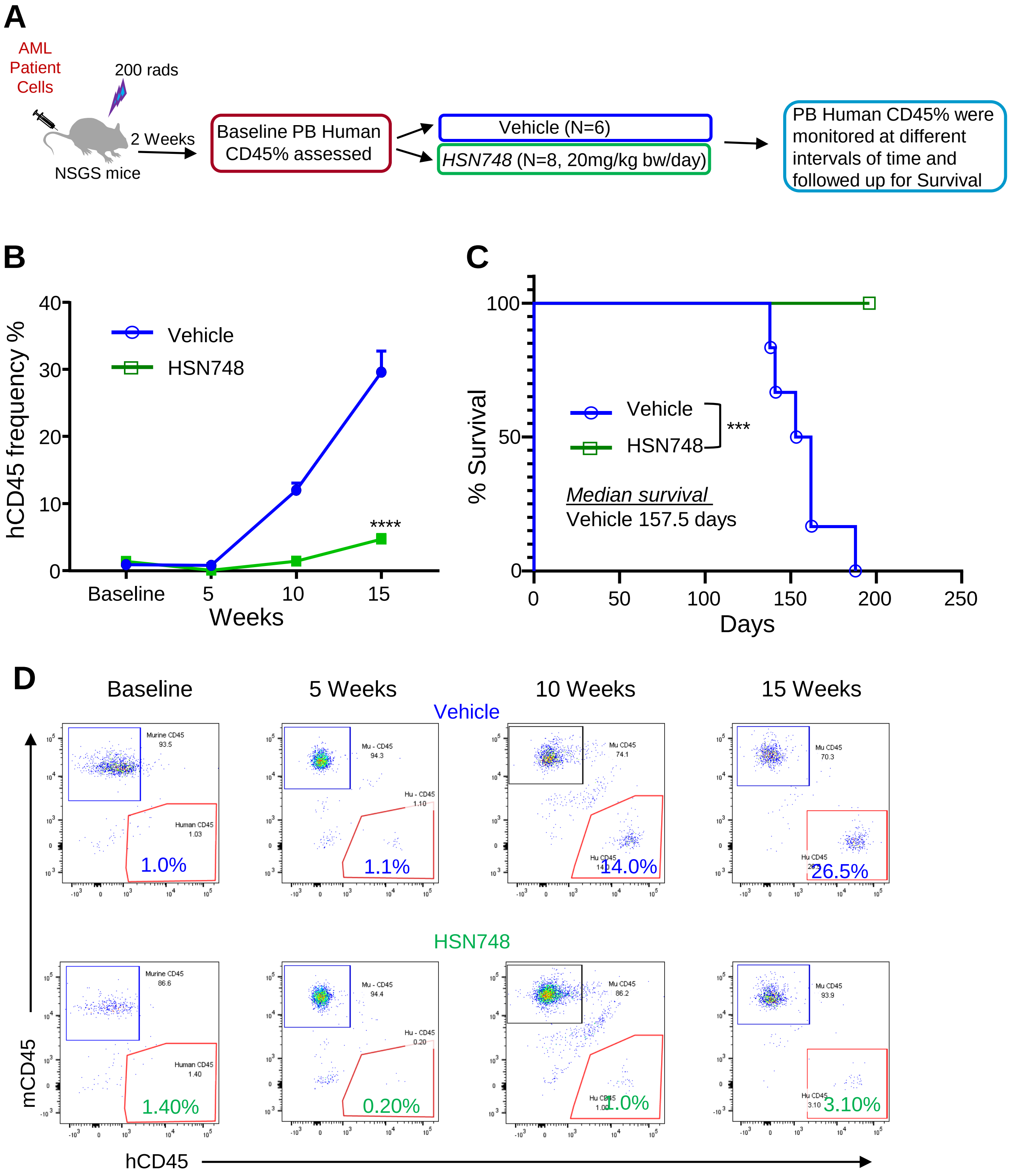

Supplementary Figure 9. Prolonged survival of AML patient derived xenografts treated with HSN748. (A) Experimental design. Briefly, multi-mutational (*FLT3^{ITD}*, *DNMT3A*, *ASXL1, NPM1)* AML patient cells were transplanted to sub lethally irradiated (200 rads) NSGS mice. Two weeks after transplanting peripheral blood hCD45 positive cells engraftment was assessed and based on the engraftment, mice were divided into vehicle and HSN748 groups randomly and followed 196 days for the effect of HSN748 treatment on hCD45 frequency and survival. (B) Shows the robust inhibitory effect of HSN748 on peripheral blood hCD45 frequency at different time points (C) Shows the Kaplan-Meier prolonged survival plot of PDXs treated with HSN748 compared to vehicle treated group. (D) Shows the representative flow prolife of hCD45 frequency of peripheral blood at different intervals of time. (N=6-7 in each group ****p<0.0001, ***p<0.001).

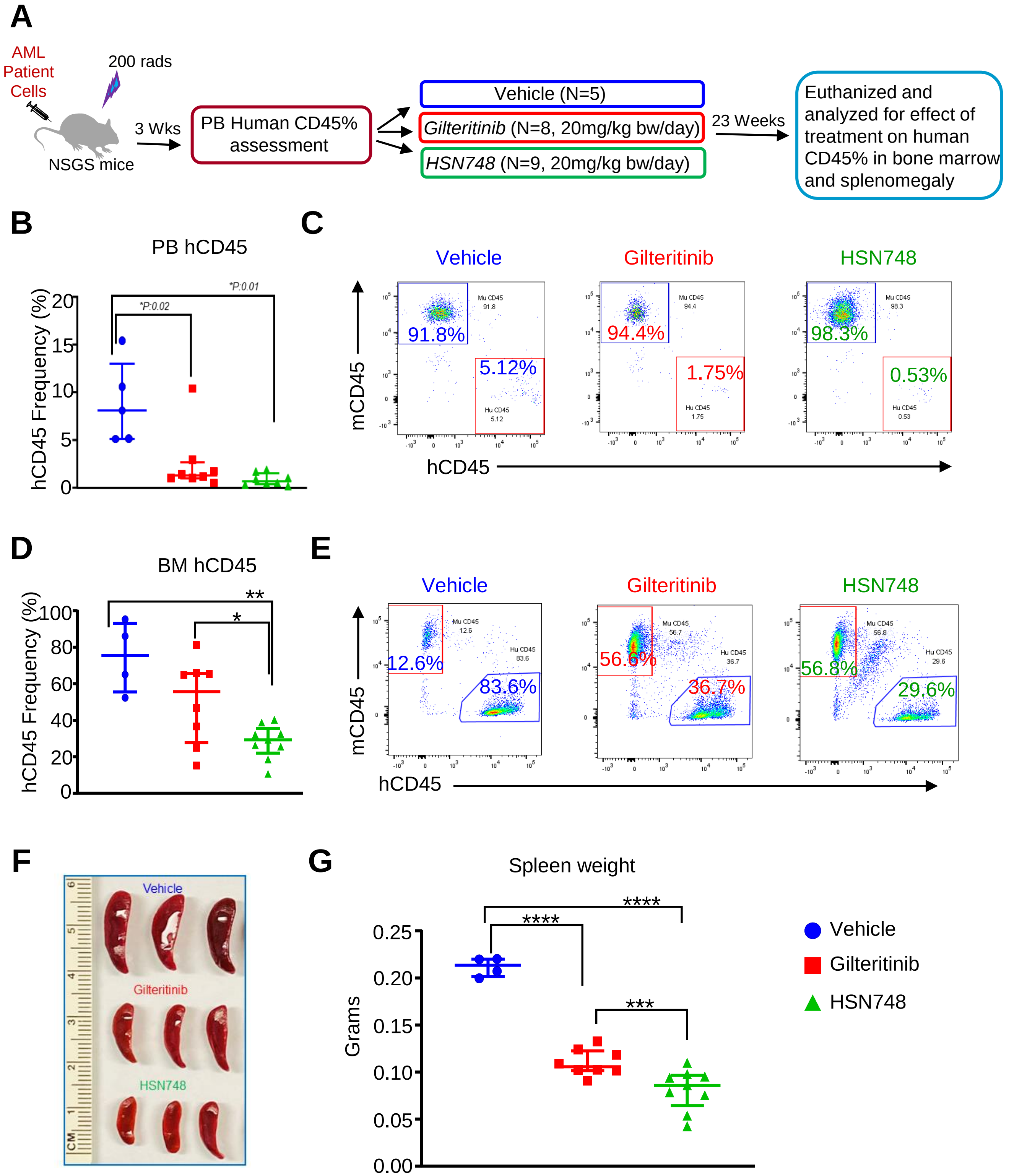

Supplementary Figure 10. Effect of HSN748 on AML patient derived xenografts (A) Experimental design. Briefly, multi-mutational (# 3263 *FLT3*^{ITD}, *DNMT3A*, *MLL*^{PTD}) AML patient cells were transplanted to sub lethally irradiated (200 rads) NSGS mice. Three weeks after transplanting peripheral blood hCD45 positive cells engraftment was assessed and based on the engraftment, mice were divided into vehicle, Gilteritinib and HSN748 groups randomly and followed 23 weeks for the effect of Gilteritinib and HSN748 treatment on hCD45 frequency and splenomegaly. (B) Shows the robust inhibitory effect of HSN748 on peripheral blood hCD45 compared to vehicle. (C) Shows the representative flow profile of peripheral blood hCD45 frequency. (D) Shows the robust inhibitory effect of HSN748 on bone marrow hCD45 frequency compared to vehicle and Gilteritinib. As one out of 5 mice from vehicle group was found dead, 4 mice data from the vehicle group was presented. (E) Shows the representative flow profile of hCD45 frequency in bone marrow. (F) Spleen pictures and spleen weight (G). Data represents ± mean by ordinary one-way ANOVA analysis. **p<0.01, *p<0.05.

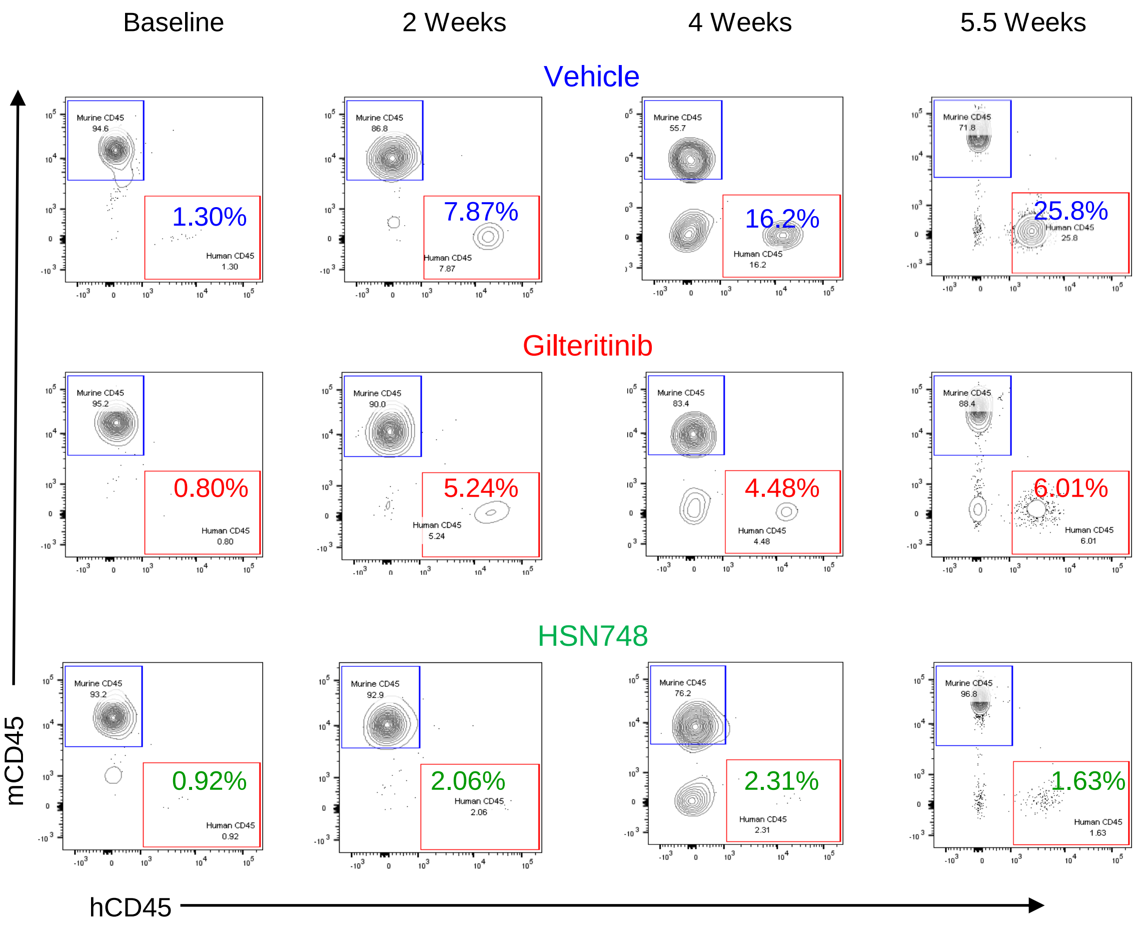

Supplementary Figure 11. Growth inhibitory effect of HSN748 compared to FDA-approved FLT3 inhibitor Gilteritinib on the development of AML in NSGS mice.

Representative flow profile of hCD45 frequency on biweekly peripheral blood assessment for the impact of HSN748 treatment on engraftment and propagation of leukemic cells. 5.5 Weeks time point quantitative data was presented in figure 7I.

Characterization of new compounds

Table of contents:

I. Chemistry:………………………………………………………………………………… S-2

Synthesis and characterization data of analogs

II. References ……………………………………………………………..………... S-21

III. HPLC data …………………………………………………………..……..… S-22

Table S3. HPLC Purity

HPLC chromatogram for compounds

III. ^{1}H and ^{13}C Spectra of analogs ………………………………………………………… S-27

I. Chemistry

General Considerations

All the solvents and reagents were purchased from widely available commercial sources and utilized as received. The ^{1}H and ^{13}C NMR spectra obtained in deuterated NMR solvents Methanol-d_4, Chloroform-*d*, or DMSO-d_6 using a 500 MHz spectrometer using internal standard tetramethylsilane. ^{1}H NMR data reported as follows: chemical shift (δ ppm) (multiplicity, coupling constant (Hz), integration). Spectral chemical shifts reported downfield order in parts per million (δ ppm). Multiplicities are reported as follows: s = singlet, d = doublet, t = triplet, q = quartet, m = multiplet, or combinations thereof. Electron spray ionization (ESI) technique and TOF mass analyzer were used to record high resolution mass spectra (HRMS). All the synthesized compounds were characterized using ^{1}H, ^{13}C NMR, and HRMS.

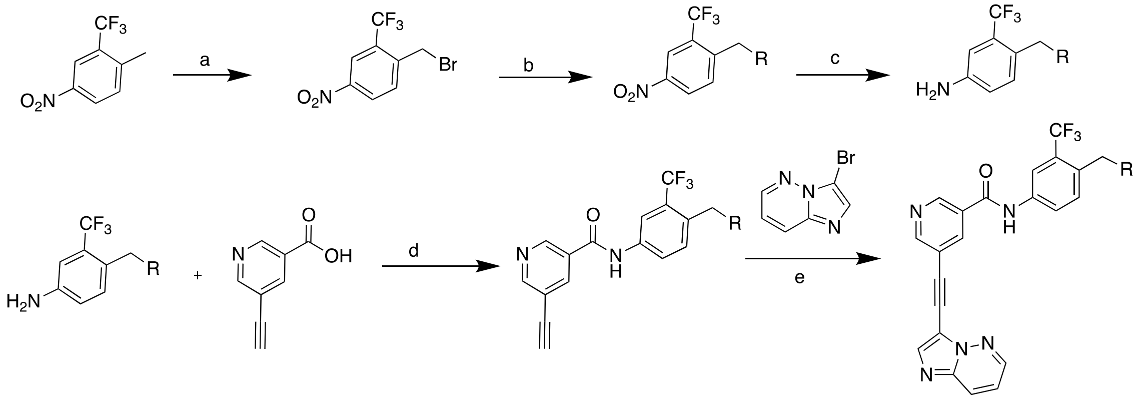

Scheme S-1. Synthetic route for the synthesis of analogs.

a) NBS (1.2 equiv), AIBN (0.1 equiv), DCE, 80 °C, 12 h. b) corresponding amine (1 equiv), DCM, TEA (3 equiv). c) Pd/C 10% (0.1 equiv) MeOH. d) HATU (1.2 equiv), DIPEA (3 equiv), DMF 50 °C. e) $Pd(PPh_3)_2Cl_2$ (5 mol%), PPh_3 (3 mol%), TEA (0.7 mL), CuI (3 mol%), DMF 80 °C.

Synthesis of 5-(imidazo[1,2-*b*]pyridazin-3-ylethynyl)-*N*-(4-((4-methylpiperazin-1-yl)methyl)-3-(trifluoromethyl)phenyl)nicotinamide (HSN748)[1].

5-((8-Amino-1,7-naphthyridin-5-yl)ethynyl)-*N*-(4-((4-methylpiperazin-1-yl)methyl)-3-(trifluoromethyl)phenyl)nicotinamide (HSN608)[2]

5-((3-Amino-6-fluoroisoquinolin-4-yl)ethynyl)-6-methyl-*N*-(4-((4-methylpiperazin-1-yl)methyl)-3-(trifluoromethyl)phenyl)nicotinamide (HSN431)[3]

1-(Bromomethyl)-4-nitro-2-(trifluoromethyl)benzene:[4]

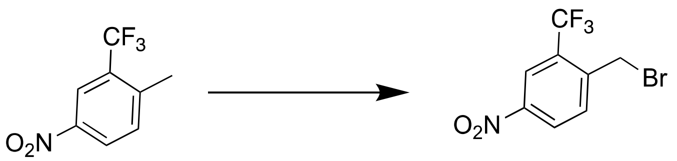

To a solution of 1-methyl-4-nitro-2-(trifluoromethyl) benzene (5 g, 24.4 mmol) in dichloroethane (100 mL) was added NBS (5.2 g, 29.2 mmol, 1.2 equiv.) and AIBN (400.6 mg, 2.4 mmol, 0.1 equiv.). The reaction mixture was allowed to stir at 80 ºC for 12h. The reaction mixture was concentrated and extracted with ethyl acetate (100 mL) and water (50 mL). The organic layer washed with brine solution (50 mL), dried over sodium sulphate and concentrated. Organic residue was purified via silica gel column chromatography to afford desired product as pale yellow liquid (lachrymating). **Yield** 60% (10 % EtoAc/Hexane)

General procedure for the synthesis of substartes

To a solution of 1-(Bromomethyl)-4-nitro-2-(trifluoromethyl)benzene (379 mg, 1.5 mmol) in dichloromethane (5 mL), corresponding amine (1.5 equiv) was added followed by addition of triethylamine (3 equiv). Reaction was allowed to stir at room temperature for an overnight. After completion, reaction was concentrated and aqueous solution of $NaHCO_3$ was added and extracted with dichloromethane. Organic layer washed with brine, dried over sodium sulphate and concentrated under reduced pressure. Purified over silica gel chromatography to get the desired product. (dichloromethans/methanol 97:3 to 95:5)

(*S*)-2-(4-nitro-2-(trifluoromethyl)benzyl)octahydropyrrolo[1,2-*a*]pyrazine

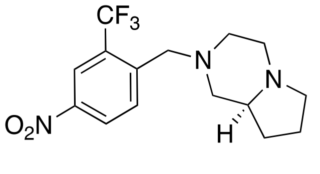

Pale yellow liquid (434 mg, 88%); ^{1}H NMR (500 MHz, Chloroform-*d*) δ 8.47 (s, 1H), 8.35 (dt, *J* = 8.6, 1.8 Hz, 1H), 8.08 (d, *J* = 8.6 Hz, 1H), 3.84 – 3.71 (m, 2H), 3.12 – 3.06 (m, 1H), 3.05 – 2.98 (m, 1H), 2.90 – 2.85 (m, 1H), 2.76 – 2.71 (m, 1H), 2.42 (td, *J* = 11.0, 2.7 Hz, 1H), 2.34 (td, *J* = 10.9, 2.8 Hz, 1H), 2.24 – 2.15 (m, 2H), 2.06 (t, *J* = 10.1 Hz, 1H), 1.88 – 1.79 (m, 1H), 1.79 – 1.71 (m, 2H), 1.46 – 1.37 (m, 1H); ^{13}C NMR (126 MHz, Chloroform-*d*) δ 146.4, 146.0,

131.5, 129.9 (q, J = 32.7), 126.4, 124.1 (q, J = 274.6 Hz), 121.4 (q, J = 6.3 Hz), 62.6, 57.7, 57.5, 53.1, 52.4, 51.3, 27.3, 21.2; HRMS (ESI) m/z calcd for $C_{15}H_{19}F_3N_3O_2$ $[M + H]^+$ 330.1429, found 330.1425.

3-Methyl-1-(4-nitro-2-(trifluoromethyl)benzyl)azetidin-3-ol

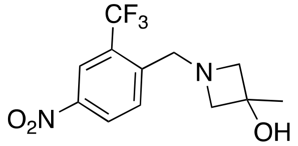

Pale yellow liquid (361 mg, 83%); ^{1}H NMR (500 MHz, Chloroform-d) δ 8.48 (s, 1H), 8.36 (d, J = 8.7 Hz, 1H), 7.97 (d, J = 8.7 Hz, 1H), 3.91 (s, 2H), 3.40 (d, J = 6.6 Hz, 2H), 3.12 (d, J = 6.9 Hz, 2H), 1.56 (s, 3H); ^{13}C NMR (126 MHz, Chloroform-d) δ 146.4, 145.3, 130.6, 129.3 (q, J = 32.7 Hz), 126.5, 124.1 (q, J = 275.9 Hz), 121.3 (q, J = 6.3 Hz), 68.9, 68.4, 58.6, 26.0**;** HRMS (ESI) m/z calcd for $C_{12}H_{14}F_3N_2O_3$ $[M + H]^+$ 291.0951, found 291.0952.

(*R*)-2,4-Dimethyl-1-(4-nitro-2-(trifluoromethyl)benzyl)piperazine

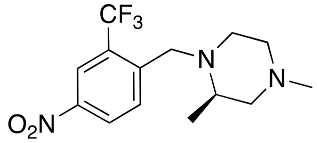

Pale yellow liquid (423 mg, 89%); ^{1}H NMR (500 MHz, Chloroform-d) δ 8.47 (d, J = 2.3 Hz, 1H), 8.35 (dd, J = 8.6, 2.4 Hz, 1H), 8.13 (d, J = 8.7 Hz, 1H), 4.14 (d, J = 16.2 Hz, 1H), 3.54 (d, J = 16.2 Hz, 1H), 2.91 – 2.76 (m, 3H), 2.67 – 2.58 (m, 1H), 2.56 – 2.48 (m, 1H), 2.40 (s, 4H), 2.17 (t, J = 10.4 Hz, 1H), 1.07 (d, J = 6.2 Hz, 3H); ^{13}C NMR (126 MHz, Chloroform-d) δ 146.9, 146.4, 131.4, 129.6 (q, J = 31.5 Hz), 126.4, 124.1 (q, J = 274.6 Hz), 121.4 (q, J = 6.3 Hz), 62.0, 55.1, 54.9, 53.3, 45.4; HRMS (ESI) m/z calcd for $C_{14}H_{19}F_3N_3O_2$ $[M + H]^+$ 318.1424, found 318.1428.

(*S*)-2,4-Dimethyl-1-(4-nitro-2-(trifluoromethyl)benzyl)piperazine

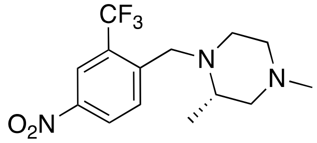

Pale yellow liquid (371 mg, 78%); ^{1}H NMR (500 MHz, Chloroform-d) δ 8.47 (d, J = 2.4 Hz, 1H), 8.34 (dd, J = 8.7, 2.4 Hz, 1H), 8.17 (d, J = 8.6 Hz, 1H), 4.19 – 4.05 (m, 2H), 3.51 (d, J = 16.3 Hz, 1H), 2.75 – 2.67 (m, 1H), 2.66 – 2.58 (m, 2H), 2.42 – 2.33 (m, 1H), 2.27 (s, 3H), 2.23 – 2.16 (m, 1H), 2.03 – 1.96 (m, 1H), 1.24 (td, J = 7.1, 0.8 Hz, 1H), 1.04 (d, J = 6.2 Hz, 3H); ^{13}C NMR (126 MHz, Chloroform-d) δ 147.4, 146.3, 131.4, 129.4 (q, J = 32.7 Hz), 126.3, 124.2 (q, J = 275.9 Hz), 121.2 (q, J = 6.3 Hz), 62.8, 55.7, 55.2, 53.5, 45.9; HRMS (ESI) m/z calcd for $C_{14}H_{19}F_3N_3O_2$ $[M + H]^+$ 318.1428, found 318.1424.

1-Isopropyl-4-(4-nitro-2-(trifluoromethyl)benzyl)piperazine

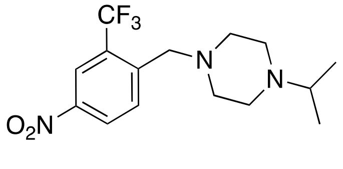

Pale yellow liquid (442 mg, 89%); ^{1}H NMR (500 MHz, Chloroform-*d*) δ 8.35 (s, 1H), 8.25 (dt, *J* = 8.8, 3.0 Hz, 1H), 7.99 (d, *J* = 8.6 Hz, 1H), 3.67 (d, *J* = 3.1 Hz, 2H), 2.87 – 2.76 (m, 1H), 2.71 – 2.54 (m, 8H), 1.15 – 1.00 (m, 6H); ^{13}C NMR (126 MHz, Chloroform-*d*) δ 146.3, 145.4, 131.6, 129.8 (q, *J* = 32.7 Hz), 126.3, 124.0 (q, *J* = 274.6 Hz), 121.3, 121.2, 57.4, 55.4, 52.2, 48.4, 18.0. HRMS (ESI) m/z calcd for $C_{15}H_{21}F_3N_3O_2$ $[M + H]^+$ 332.1580, found 332.1581.

(*R*)-*N*,*N*-Dimethyl-1-(4-nitro-2-(trifluoromethyl)benzyl)pyrrolidin-3-amine

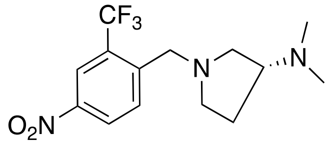

Pale yellow liquid (366 mg, 77%); ^{1}H NMR (500 MHz, Chloroform-*d*) δ 8.48 (d, *J* = 2.4 Hz, 1H), 8.35 (dd, *J* = 8.6, 2.4 Hz, 1H), 8.06 (d, *J* = 8.6 Hz, 1H), 3.85 (q, *J* = 16.0 Hz, 2H), 2.88 – 2.77 (m, 1H), 2.76 – 2.57 (m, 3H), 2.57 – 2.46 (m, 1H), 2.20 (d, *J* = 0.8 Hz, 6H), 2.10 – 1.96 (m, 1H), 1.83 – 1.73 (m, 1H). ^{13}C NMR (126 MHz, Chloroform-*d*) δ 146.3, 131.4, 129.3 (q, *J* = 30.2 Hz), 126.4, 124.2 (q, *J* = 274.6 Hz), 121.3 (q, *J* = 6.3 Hz), 65.4, 58.4, 55.5, 53.6, 43.8, 29.4; HRMS (ESI) m/z calcd for $C_{14}H_{19}F_3N_3O_2$ $[M + H]^+$ 318.1424, found 318.1428.

(*S*)-*N*,*N*-Dimethyl-1-(4-nitro-2-(trifluoromethyl)benzyl)pyrrolidin-3-amine

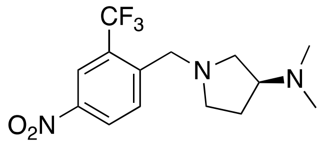

Pale yellow liquid (385 mg, 81%); ^{1}H NMR (500 MHz, Chloroform-*d*) δ 8.46 (d, *J* = 2.3 Hz, 1H), 8.34 (dd, *J* = 8.6, 2.4 Hz, 1H), 8.04 (d, *J* = 8.6 Hz, 1H), 3.91 – 3.78 (m, 2H), 2.94 (dq, *J* = 8.5, 6.5 Hz, 1H), 2.78 – 2.72 (m, 1H), 2.72 – 2.56 (m, 3H), 2.28 (s, 6H), 2.09 – 1.99 (m, 1H), 1.92 – 1.78 (m, 1H); ^{13}C NMR (126 MHz, Chloroform-*d*) δ 146.4, 146.0, 131.5, 129.4 (q, *J* = 31.5), 126.5, 124.2 (q, *J* = 274.2), 121.3 (q, *J* = 6.3 Hz), 65.3, 57.9, 55.4, 53.4, 43.3, 28.8; HRMS (ESI) m/z calcd for $C_{14}H_{19}F_3N_3O_2$ $[M + H]^+$ 318.1429, found 318.1425.

1-Methyl-4-(4-nitro-2-(trifluoromethyl)benzyl)-1,4-diazepane

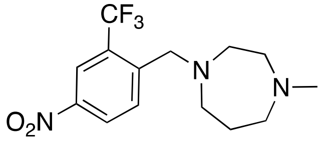

Yellow liquid (358 mg, 75%); ^{1}H NMR (500 MHz, Chloroform-*d*) δ 8.50 (d, *J* = 2.4 Hz, 1H), 8.38 (dd, *J* = 8.7, 2.4 Hz, 1H), 8.11 (d, *J* = 8.6 Hz, 1H), 3.91 (s, 2H), 2.99 – 2.92 (m, 2H), 2.85 (s, 3H), 2.78 (t, *J* = 6.0 Hz, 2H), 2.55 (s, 3H), 2.05 – 1.95 (m, 2H); ^{13}C NMR (126 MHz, Chloroform-*d*) δ 146.5, 146.2, 131.7, 129.8 (q, *J* = 32.7 Hz), 126.5, 124.2 (q, *J* = 274.6 Hz),

122.0, 121.6 (q, J = 6.3 Hz), 58.2, 58.0, 56.1, 54.4, 53.2, 46.1, 26.3; HRMS (ESI) m/z calcd for $C_{14}H_{19}F_3N_3O_2$ $[M + H]^+$ 318.1424, found 318.1425.

2-(4-(4-Nitro-2-(trifluoromethyl)benzyl)piperazin-1-yl)ethan-1-ol

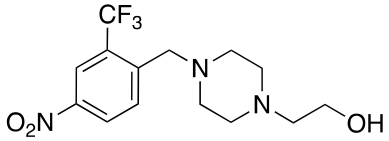

Pale yellow liquid (405 mg, 81%); ^{1}H NMR (500 MHz, Chloroform-d) δ 8.60 – 8.49 (m, 1H), 8.39 – 8.27 (m, 1H), 8.17 – 7.93 (m, 1H), 3.83 – 3.71 (m, 2H), 3.67 – 3.54 (m, 2H), 2.87 (s, 1H), 2.67 – 2.39 (m, 10H); ^{13}C NMR (126 MHz, Chloroform-d) δ 146.4, 145.7, 131.5, 129.9 (q, J = 30.2), 126.4, 124.1 (q, J = 274.6 Hz), 121.4, 59.2, 57.7, 53.2, 52.8; HRMS (ESI) m/z calcd for $C_{14}H_{19}F_3N_3O_3$ $[M + H]^+$ 334.1373, found 334.1373.

1-(2-Chloro-4-nitrobenzyl)-4-methylpiperazine:

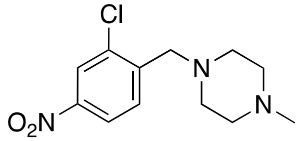

Synthesized from 1-(bromomethyl)-2-chloro-4-nitrobenzene and 1-methylpiperazine as substrates using general procedure. Pale yellow solid (351 mg, 87%); **^{1}H NMR** (500 MHz, Chloroform-d) δ 8.22 (d, J = 2.3 Hz, 1H), 8.09 (dd, J = 8.5, 2.3 Hz, 1H), 7.73 (d, J = 8.6 Hz, 1H), 3.67 (s, 2H), 2.62 – 2.42 (bs, 8H), 2.30 (s, 3H); **^{13}C NMR** (126 MHz, $CDCl_3$) δ 147.10, 144.00, 134.65, 130.56, 124.54, 121.55, 58.89, 55.12, 53.28, 46.04; **HRMS (ESI$^+$):** calcd. for $C_{12}H_{17}ClN_3O_2$ (MH^+) 270.1004, found 270.1003.

General procedure for the synthesis of substartes S-II via reduction of substartes S-I

A solution of nitro subsatrte (S-I, 3 equiv) in methanol (50 mL) was prepared under Argon condition. Pd/C (10 %, 0.1 equiv) was added followed by replacement of argon with hydrogen, purged with hydrogen balloon three times. Reaction was allowed to stir at room temperature for an overnight under hydrogen balloon. Upon completion reaction was filtered through celite bed. Filtrate concentrated to give the desired amine product.

(*S*)-4-((hexahydropyrrolo[1,2-*a*]pyrazin-2(1*H*)-yl)methyl)-3-(trifluoromethyl)aniline

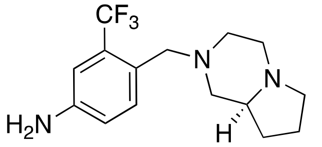

Pale yellow liquid (273 mg, 91%); ^{1}H NMR (500 MHz, Chloroform-d) δ 7.42 (d, J = 8.3 Hz, 1H), 6.88 (d, J = 2.5 Hz, 1H), 6.75 (dd, J = 8.3, 2.6 Hz, 1H), 3.84 (s, 2H), 3.59 – 3.45 (m, 2H), 3.11 – 3.01 (m, 1H), 2.99 – 2.93 (m, 1H), 2.88 (ddd, J = 10.8, 2.7, 1.5 Hz, 1H), 2.77 – 2.68 (m, 1H), 2.36 – 2.25 (m, 2H), 2.23 – 2.12 (m, 2H), 1.94 (t, J = 10.2 Hz, 1H), 1.84 – 1.76 (m, 1H), 1.73 – 1.65 (m, 2H), 1.46 – 1.36 (m, 1H); ^{13}C NMR (126 MHz, Chloroform-d) δ 145.2, 129.5

(q, J = 30.2 Hz), 126.6, 125.4 (q, J = 274.6 Hz), 117.7, 112.1 (q, J = 6.3 Hz), 62.7, 57.6, 57.2, 53.0, 52.0, 51.3, 27.2, 21.2; HRMS (ESI) m/z calcd for $C_{15}H_{21}F_3N_3$ $[M + H]^+$ 300.1688, found 300.1683.

1-(4-Amino-2-(trifluoromethyl)benzyl)-3-methylazetidin-3-ol

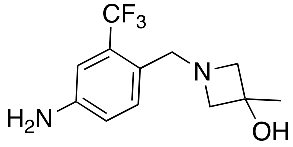

Yellow solid (236 mg, 94%); ^{1}H NMR (500 MHz, Chloroform-d) δ 7.28 (d, J = 8.3 Hz, 1H), 6.87 (d, J = 2.5 Hz, 1H), 6.72 (dd, J = 8.3, 2.5 Hz, 1H), 3.69 – 3.65 (m, 2H), 3.35 (s, 2H), 3.26 (d, J = 8.6 Hz, 2H), 3.06 (d, J = 8.5 Hz, 2H); ^{13}C NMR (126 MHz, Chloroform-d) δ 145.2, 130.7, 128.9 (q, J = 30.2 Hz), 125.8, 125.4 (q, J = 274.6 Hz), 117.8, 112.3 (q, J = 6.3 Hz), 68.5, 68.0, 58.4, 50.0, 25.97;HRMS (ESI) m/z calcd for $C_{12}H_{16}F_3N_2O$ $[M + H]^+$ 261.1210, found 261.1209.

(*R*)-4-((2,4-Dimethylpiperazin-1-yl)methyl)-3-(trifluoromethyl)aniline

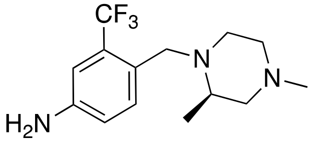

Yellow liquid (264 mg, 92%); ^{1}H NMR (500 MHz, Chloroform-d) δ 7.50 (d, J = 8.3 Hz, 1H), 6.88 (d, J = 2.6 Hz, 1H), 6.76 (dd, J = 8.4, 2.5 Hz, 1H), 3.98 (d, J = 14.4 Hz, 1H),3.86 (s, 2H), 3.18 (d, J = 14.3 Hz, 1H), 2.67 (dt, J = 11.2, 2.3 Hz, 1H), 2.61 (dt, J = 13.7, 4.2 Hz, 2H), 2.55 – 2.48 (m, 1H), 2.24 (s, 3H), 2.22 – 2.17 (m, 1H), 2.15 – 2.09 (m, 1H), 1.97 (t, J = 10.2 Hz, 1H), 1.06 (d, J = 6.2 Hz, 3H); ^{13}C NMR (126 MHz, Chloroform-d) δ 144.9, 131.7, 129.3 (q, J = 30.2 Hz), 127.7, 125.5 (q, J = 273.4 Hz), 117.8, 112.0 (q, J = 6.3 Hz), 62.8, 55.6, 55.3, 53.0, 45.8; HRMS (ESI) m/z calcd for $C_{14}H_{21}F_3N_3$ $[M + H]^+$ 288.1684, found 288.1682.

(*S*)-4-((2,4-Dimethylpiperazin-1-yl)methyl)-3-(trifluoromethyl)aniline

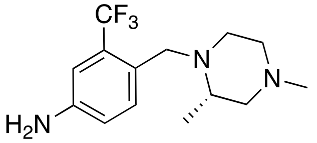

Yellow liquid (253 mg, 88%); ^{1}H NMR (500 MHz, Chloroform-d) δ 7.52 (d, J = 8.3 Hz, 1H), 7.00 – 6.83 (m, 1H), 6.78 (dt, J = 8.2, 1.9 Hz, 1H), 4.00 (d, J = 14.4 Hz, 1H), 3.21 (d, J = 14.4 Hz, 1H), 2.81 – 2.49 (m, 5H), 2.37 – 2.11 (m, 6H), 2.07 – 1.91 (m, 1H), 1.08 (dd, J = 6.2, 1.3 Hz, 3H); ^{13}C NMR (126 MHz, $CDCl_3$) δ 144.9, 131.8, 129.1(q, J = 30.2 Hz), 127.8, 125.5(q, J = 274.6 Hz), 117.8, 112.0, 112.0, 62.85, 55.4, 53.1, 45.8;HRMS (ESI) m/z calcd for $C_{14}H_{21}F_3N_3$ $[M + H]^+$ 288.1684, found 288.1682.

4-((4-isopropylpiperazin-1-yl)methyl)-3-(trifluoromethyl)aniline

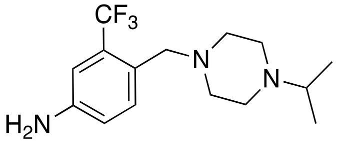

Yellow liquid (286 mg, 95%); ^{1}H NMR (500 MHz, Chloroform-*d*) δ 7.44 (d, *J* = 8.3 Hz, 1H), 6.91 (d, *J* = 2.5 Hz, 1H), 6.78 (d, *J* = 8.3 Hz, 1H), 3.77 (s, 2H), 3.53 (s, 2H), 2.79 – 2.66 (m, 1H), 2.65 – 2.48 (m, 8H), 1.09 (dd, *J* = 6.6, 2.2 Hz, 6H); ^{13}C NMR (126 MHz, Chloroform-*d*) δ 145.1, 132.0, 129.6 (q, *J* = 28.9 Hz), 126.6, 125.4 (q, *J* = 274.6 Hz), 117.7, 112.1 (q, *J* = 6.3 Hz), 57.8, 54.8, 52.9, 48.7, 18.5; HRMS (ESI) m/z calcd for $C_{15}H_{23}F_{3}N_{3}$ $[M + H]^{+}$ 302.1838, found 302.1834.

(*R*)-*N*,*N*-Dimethyl-1-(4-nitro-2-(trifluoromethyl)benzyl)pyrrolidin-3-amine

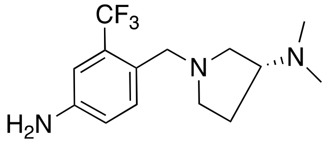

Yellow liquid (261 mg, 91%); ^{1}H NMR (500 MHz, Chloroform-*d*) δ 7.46 (d, *J* = 8.3 Hz, 1H), 6.91 (d, *J* = 2.5 Hz, 1H), 6.79 (dd, *J* = 8.3, 2.5 Hz, 1H), 3.76 (s, 2H), 3.64 (q, *J* = 14.2 Hz, 2H), 2.87 – 2.79 (m, 1H), 2.74 (dd, *J* = 9.1, 7.0 Hz, 1H), 2.67 (td, *J* = 8.4, 5.9 Hz, 1H), 2.58 (dd, *J* = 8.9, 6.0 Hz, 1H), 2.48 – 2.41 (m, 1H), 2.22 (s, 6H), 1.99 (dtd, *J* = 13.9, 8.4, 6.0 Hz, 1H), 1.73 (ddt, *J* = 12.0, 8.2, 6.0 Hz, 1H); ^{13}C NMR (126 MHz, Chloroform-*d*) δ 144.9, 131.6, 129.0 (q, *J* = 30.2 Hz), 127.4, 123.3 (q, *J* = 274.6), 117.8, 112.0, 65.4, 58.2, 55.5, 53.3, 43.6, 29.0; HRMS (ESI) m/z calcd for $C_{14}H_{21}F_{3}N_{3}$ $[M + H]^{+}$ 288.1687, found 288.1684.

(*S*)-1-(4-Amino-2-(trifluoromethyl)benzyl)-*N*,*N*-dimethylpyrrolidin-3-amine

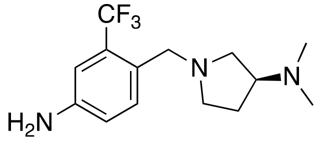

Yellow liquid (270 mg, 94%); ^{1}H NMR (500 MHz, Chloroform-*d*) δ 7.44 (d, *J* = 8.3 Hz, 1H), 6.90 (d, *J* = 2.5 Hz, 1H), 6.78 (dd, *J* = 8.3, 2.5 Hz, 1H), 3.78 (s, 2H), 3.63 (qd, *J* = 14.1, 1.5 Hz, 2H), 2.86 – 2.78 (m, 1H), 2.77 – 2.71 (m, 1H), 2.69 – 2.62 (m, 1H), 2.60 – 2.53 (m, 1H), 2.47 – 2.40 (m, 1H), 2.21 (s, 6H), 2.03 – 1.92 (m, 1H), 1.78 – 1.68 (m, 1H);^{13}C NMR (126 MHz, Chloroform-*d*) δ 145.0, 131.6, 129.0 (q, *J* = 30.2 Hz), 127.3, 125.5 (q, *J* = 274.6 Hz), 117.8, 112.0 (q, *J* = 6.3 Hz), 65.4, 58.1, 55.4, 53.3, 43.5, 29.0; HRMS (ESI) m/z calcd for $C_{14}H_{21}F_{3}N_{3}$ $[M + H]^{+}$ 288.16876, found 288.1682.

4-((4-Methyl-1,4-diazepan-1-yl)methyl)-3-(trifluoromethyl)aniline

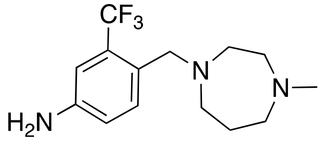

Pale Yellow liquid (255 mg, 89%); ^{1}H NMR (500 MHz, Chloroform-*d*) δ 7.44 (d, *J* = 8.3 Hz, 1H), 6.86 (s, 1H), 6.74 (dd, *J* = 8.5, 2.5 Hz, 1H), 3.93 (s, 2H), 3.59 (s, 2H), 2.80 – 2.72 (m, 2H), 2.69 – 2.60 (m, 6H), 2.39 (s, 3H), 1.90 – 1.79 (m, 2H). ^{13}C NMR (126 MHz, Chloroform-*d*) δ 145.3, 131.9, 129.3 (q, *J* = 30.2 Hz), 127.2, 125.5 (q, *J* = 274.6 Hz), 117.8, 112.0, 112.0, 58.1, 57.9, 56.2, 54.1, 53.0, 46.3, 26.6; HRMS (ESI) m/z calcd for $C_{14}H_{21}F_{3}N_{3}$ $[M + H]^{+}$ 288.1682, found 288.1683.

2-(4-(4-Amino-2-(trifluoromethyl)benzyl)piperazin-1-yl)ethan-1-ol

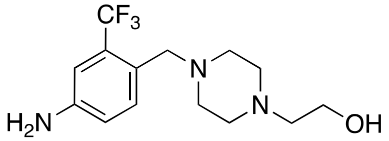

Yellow liquid (279 mg, 92%); ^{1}H NMR (500 MHz, Chloroform-*d*) δ 7.44 (d, *J* = 8.3 Hz, 1H), 6.89 (d, *J* = 2.5 Hz, 1H), 6.76 (dd, *J* = 8.3, 2.5 Hz, 1H), 3.68 (s, 2H), 3.60 (t, *J* = 5.4 Hz, 2H), 3.51 (d, *J* = 1.9 Hz, 2H), 2.59 – 2.39 (m, 10H); ^{13}C NMR (126 MHz, Chloroform-*d*) δ 145.2, 131.9, 129.5 (q, *J* = 30.2 Hz), 126.5, 125.4 (q, *J* = 274.6), 117.8, 112.0 (q, *J* = 6.3 Hz), 59.3, 57.8, 57.7, 53.0, 52.9; HRMS (ESI) m/z calcd for $C_{14}H_{21}F_3N_3O$ [M + H]$^+$ 304.1631, found 304.1631.

3-Chloro-4-((4-methylpiperazin-1-yl)methyl)aniline

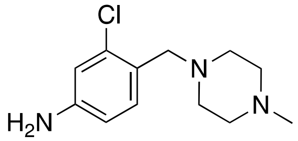

Pale yellow solid (229 mg, 96%); ^{1}H NMR (500 MHz, DMSO-*d*6) δ 7.00 (d, *J* = 8.2 Hz, 1H), 6.56 (d, *J* = 2.2 Hz, 1H), 6.45 (dd, *J* = 8.3, 2.3 Hz, 1H), 5.25 (s, 2H), 2.47 – 2.15 (m, 8H), 2.12 (s, 3H). ^{13}C NMR (126 MHz, DMSO-*d*6) δ 149.4, 134.2, 132.2, 122.0, 114.0, 113.0, 58.7, 55.2, 52.8, 46.1; HRMS (ESI) m/z calcd for $C_{12}H_{19}ClN_3$ [M + H]$^+$ 240.1262, found 240.1260

General procedure for the amide coupling

To a solution of amine (500 mg) and carboxylic acid substrate in DMF (10 mL), HATU (1.2 equiv) and DIPEA (3 equiv) was added. Reaction was allowed to stir at 50 °C for an overnight. After completion reaction was concentrated and extracted with ethyl acetate and water, washed with brine. Collected organic layer dried over sodium sulphate, concentrated and purified via silica gel chromatography to afford the pure desired compound (dichloromethans/methanol 97:3 to 95:5).

(*S*)-5-ethynyl-*N*-(4-((hexahydropyrrolo[1,2-*a*]pyrazin-2(1*H*)-yl)methyl)-3-(trifluoromethyl)phenyl)nicotinamide

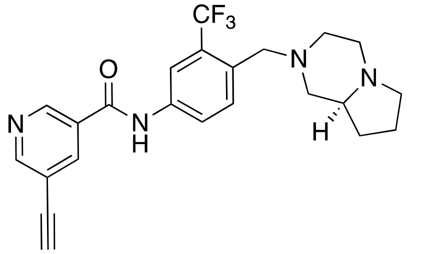

Pale yellow solid (210 mg, 49%); ^{1}H NMR (500 MHz, Methanol-d_4) δ 9.04 (s, 1H), 8.79 (s, 1H), 8.40 (t, J = 2.1 Hz, 1H), 8.11 (d, J = 2.2 Hz, 1H), 7.91 (dd, J = 8.5, 2.3 Hz, 1H), 7.75 (d, J = 8.4 Hz, 1H), 3.92 (s, 1H), 3.74 – 3.56 (m, 2H), 3.10 – 2.98 (m, 2H), 2.98 – 2.87 (m, 1H), 2.85 – 2.72 (m, 1H), 2.39 – 2.27 (m, 2H), 2.27 – 2.18 (m, 2H), 1.99 (t, J = 10.3 Hz, 1H), 1.87 – 1.73 (m, 3H), 1.46 – 1.36 (m, 1H); ^{13}C NMR (126 MHz, Methanol-d_4) δ 164.0, 154.0, 147.5, 138.4, 137.4, 133.2, 131.2, 130.3, 128.8 (q, J = 31.5 Hz), 125.3 (q, J = 274.6 Hz), 123.5, 119.7, 117.7, 117.6, 82.6, 78.6, 62.7, 57.3, 56.6, 52.5, 51.6, 50.9, 26.6, 20.5; HRMS (ESI) m/z calcd for $C_{23}H_{24}F_3N_4O$ [M + H]$^+$ 429.1902, found 429.1899.

5-Ethynyl-*N*-(4-((3-hydroxy-3-methylazetidin-1-yl)methyl)-3-(trifluoromethyl)phenyl)nicotinamide

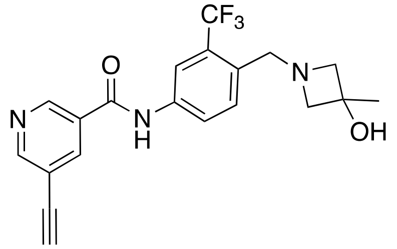

Pale yellow solid (198 mg, 51%); ^{1}H NMR (500 MHz, Methanol-d_4) δ 9.03 (s, 1H), 8.77 (s, 1H), 8.40 (d, J = 2.2 Hz, 1H), 8.14 (s, 1H), 7.94 (dd, J = 8.5, 2.3 Hz, 1H), 7.63 (d, J = 8.6 Hz, 1H), 3.98 – 3.88 (m, 3H), 3.53 – 3.45 (m, 2H), 3.23 (d, J = 7.9 Hz, 2H), 1.49 (s, 3H); ^{13}C NMR (126 MHz, Methanol-d_4) δ 164.0, 154.1, 147.5, 138.4, 137.7, 131.5, 130.3, 128.4 (q, J = 30.2 Hz), 125.2 (q, J = 274.6 Hz), 123.6, 119.7, 117.8, 117.8, 82.6, 78.6, 67.7, 67.2, 57.9, 24.7; HRMS (ESI) m/z calcd for $C_{20}H_{19}F_3N_3O_2$ [M + H]$^+$ 390.1424, found 390.1423.

(*R*)-*N*-(4-((2,4-Dimethylpiperazin-1-yl)methyl)-3-(trifluoromethyl)phenyl)-5-ethynylnicotinamide (5.3)

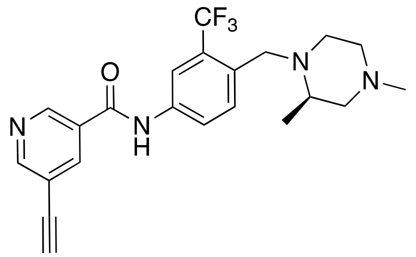

Pale yellow semi-solid (187 mg, 45%); ^{1}H NMR (500 MHz, Chloroform-d) δ 9.67 (s, 1H), 9.19 (d, J = 2.2 Hz, 1H), 8.74 (d, J = 2.0 Hz, 1H), 8.38 (d, J = 2.2 Hz, 1H), 8.04 (d, J = 2.3 Hz, 1H), 7.94 (dd, J = 8.4, 2.3 Hz, 1H), 7.65 (d, J = 8.5 Hz, 1H), 4.06 (d, J = 14.7 Hz, 1H), 3.33 – 3.23 (m, 2H), 3.01 – 2.89 (m, 2H), 2.83 – 2.73 (m, 1H), 2.68 – 2.60 (m, 1H), 2.56 – 2.43 (m, 4H), 2.38 – 2.27 (m, 1H), 1.40 (d, J = 6.6 Hz, 1H), 1.09 (d, J = 6.3 Hz, 3H);^{13}C NMR (126 MHz, $CDCl_3$) δ 163.7, 154.9, 148.1, 138.6, 136.9, 133.8, 131.1, 129.7, 128.7(q, J = 30.24 Hz), 125.1(q, J = 274.6 Hz), 124.0, 119.3, 118.5, 81.9, 79.4, 61.2, 54.6, 53.8, 52.8, 44.6; HRMS (ESI) m/z calcd for $C_{22}H_{24}F_3N_4O$ [M + H]$^+$ 417.1897, found 417.1895.

(*S*)-*N*-(4-((2,4-Dimethylpiperazin-1-yl)methyl)-3-(trifluoromethyl)phenyl)-5-ethynylnicotinamide

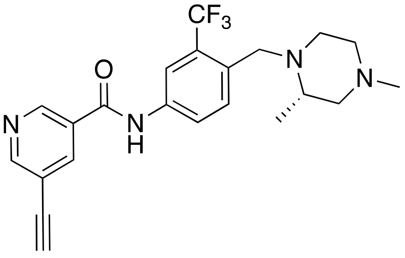

Pale yellow semi-solid (204 mg, 49%); ^{1}H NMR (500 MHz, Chloroform-*d*) δ 9.62 – 9.46 (m, 1H), 9.03 – 8.49 (m, 1H), 8.72 – 8.58 (m, 1H), 8.26 – 8.13 (m, 1H), 7.83 – 7.61 (m, 3H), 4.01 – 3.82 (m, 1H), 3.31 – 3.17 (m, 2H), 2.64 – 2.40 (m, 4H), 2.24 – 2.04 (m, 5H), 1.96 – 1.84 (m, 1H), 1.01 – 0.90 (m, 3H); ^{13}C NMR (126 MHz, $CDCl_3$) δ 163.9, 154.8, 147.6, 138.2, 136.0, 135.6, 131.0, 129.7, 128.7 (q, *J* = 30.2), 125.0 (q, *J* = 274.6), 124.0, 119.4, 118.2, 82.2, 79.1, 62.8, 55.5, 55.3, 53.1, 45.8, 30.9; HRMS (ESI) m/z calcd for $C_{22}H_{24}F_3N_4O$ [M + H]$^+$ 417.1902, found 417.1894.

5-Ethynyl-*N*-(4-((4-isopropylpiperazin-1-yl)methyl)-3-(trifluoromethyl)phenyl)nicotinamide

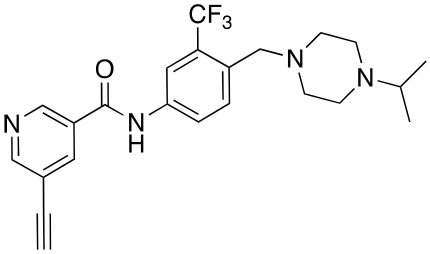

Pale yellow semi-solid (215 mg, 50%); ^{1}H NMR (500 MHz, Chloroform-*d*) δ 9.00 (d, *J* = 2.2 Hz, 1H), 8.97 – 8.87 (m, 1H), 8.75 (d, *J* = 2.1 Hz, 1H), 8.23 (d, *J* = 2.1 Hz, 1H), 7.81 (dd, *J* = 4.8, 2.5 Hz, 2H), 7.76 – 7.70 (m, 1H), 3.57 (s, 2H), 3.28 (s, 1H), 2.67 – 2.59 (m, 1H), 2.55 – 2.43 (m, 8H), 1.03 (d, *J* = 6.4 Hz, 6H); ^{13}C NMR (126 MHz, Chloroform-*d*) δ 163.5, 155.0, 147.3, 138.2, 136.0, 134.5, 131.3, 129.8, 129.3 (q, *J* = 30.2 Hz), 125.0 (q, *J* = 274.6 Hz), 123.7, 119.6, 118.0, 82.3, 79.1, 57.7, 54.4, 53.4, 48.7, 18.6; HRMS (ESI) m/z calcd for $C_{23}H_{26}F_3N_4O$ [M + H]$^+$ 431.2053, found 431.2052.

(*R*)-*N*-(4-((3-(Dimethylamino)pyrrolidin-1-yl)methyl)-3-(trifluoromethyl)phenyl)-5-ethynylnicotinamide

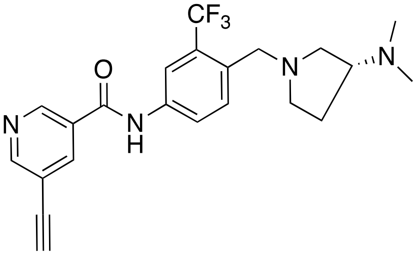

Pale yellow semi-solid (187 mg, 45%); ^{1}H NMR (500 MHz, Chloroform-*d*) δ 9.12 (s, 1H), 9.00 (d, *J* = 2.2 Hz, 1H), 8.73 (d, *J* = 2.0 Hz, 1H), 8.23 (t, *J* = 2.1 Hz, 1H), 7.84 (d, *J* = 2.3 Hz, 1H), 7.79 (dd, *J* = 8.5, 2.3 Hz, 1H), 7.67 (d, *J* = 8.5 Hz, 1H), 3.68 (q, *J* = 14.7 Hz, 2H), 3.28 (s, 1H), 2.83 – 2.73 (m, 1H), 2.72 – 2.66 (m, 1H), 2.64 – 2.53 (m, 2H), 2.47 – 2.40 (m, 1H), 2.16 (s, 6H), 1.99 – 1.91 (m, 1H), 1.74 – 1.66 (m, 1H); ^{13}C NMR (126 MHz, $CDCl_3$) δ 163.5, 154.9, 147.3, 138.3, 136.1, 134.8, 131.1, 129.9, 128.7 (q, *J* = 30.2 Hz), 125.0 (q, *J* = 274.6 Hz), 123.8,

119.6, 118.0, 82.2, 79.1, 65.4, 58.2, 55.4, 53.5, 43.6, 29.1; HRMS (ESI) m/z calcd for $C_{22}H_{24}F_3N_4O$ [M + H]$^+$ 417.1902, found 417.1895.

(*S*)-*N*-(4-((3-(Dimethylamino)pyrrolidin-1-yl)methyl)-3-(trifluoromethyl)phenyl)-5-ethynylnicotinamide

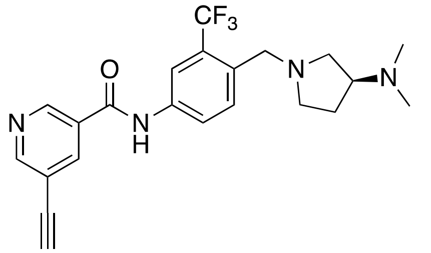

Pale yellow semi-solid (200 mg, 48%); ^{1}H NMR (500 MHz, Chloroform-*d*) δ 9.21 (s, 1H), 9.00 (s, 1H), 8.73 (s, 1H), 8.23 (s, 1H), 7.89 – 7.76 (m, 2H), 7.67 (d, *J* = 8.5 Hz, 1H), 3.68 (q, *J* = 14.8 Hz, 2H), 3.28 (s, 1H), 2.80 (t, *J* = 7.5 Hz, 1H), 2.69 (t, *J* = 8.2 Hz, 1H), 2.65 – 2.54 (m, 2H), 2.45 (dd, *J* = 9.2, 6.5 Hz, 1H), 2.18 (s, 6H), 2.02 – 1.90 (m, 1H), 1.71 (dq, *J* = 13.6, 6.9 Hz, 1H); ^{13}C NMR (126 MHz, $CDCl_3$) δ 163.6, 154.9, 147.4, 138.3, 136.1, 134.8, 131.1, 129.8, 128.7 (q, *J* = 31.5 Hz), 125.0 (q, *J* = 274.6 Hz), 123.8, 119.5, 118.1, 82.2, 79.1, 65.3, 58.1, 55.4, 53.4, 43.4, 28.9. HRMS (ESI) m/z calcd for $C_{22}H_{24}F_3N_4O$ [M + H]$^+$ 417.1902, found 417.1894

5-Ethynyl-*N*-(4-((4-methyl-1,4-diazepan-1-yl)methyl)-3-(trifluoromethyl)phenyl)nicotinamide

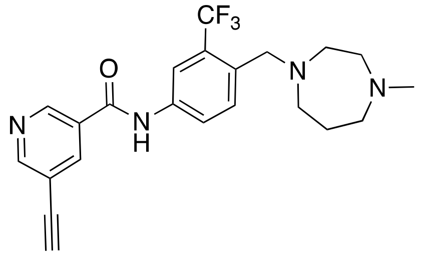

Pale yellow solid (133 mg, 32%); ^{1}H NMR (500 MHz, Methanol-d_4) δ 9.06 (s, 1H), 8.81 (s, 1H), 8.42 (s, 1H), 8.14 (s, 1H), 7.97 (d, *J* = 8.5 Hz, 1H), 7.84 (d, *J* = 8.5 Hz, 1H), 3.94 (s, 1H), 3.83 (s, 2H), 3.22 – 3.14 (m, 2H), 2.90 – 2.84 (m, 2H), 2.78 (s, 4H), 2.11 – 1.99 (m, 2H), 1.91 (s, 3H); ^{13}C NMR (126 MHz, MeOD) δ 164.1, 154.1, 147.5, 138.4, 137.6, 133.5, 131.3, 130.4, 128.3, 123.7 (q, *J* = 30.2 Hz), 123.2 (q, *J* = 274.6 Hz), 119.7, 117.8, 82.6, 78.6, 57.6, 57.1, 55.0, 53.7, 50.0, 43.8, 24.6; HRMS (ESI) m/z calcd for $C_{22}H_{24}F_3N_4O$ [M + H]$^+$ 417.1897, found 417.1899.

***N*-(4-(2-(Dimethylamino)ethoxy)-3-(trifluoromethyl)phenyl)-5-ethynylnicotinamide**

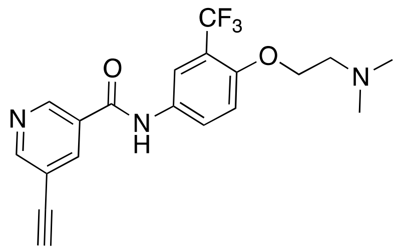

Pale yellow semi-solid (211 mg, 56%); ^{1}H NMR (500 MHz, Methanol-d_4) δ 9.03 (d, J = 2.2 Hz, 1H), 8.77 (d, J = 2.0 Hz, 1H), 8.38 (t, J = 2.1 Hz, 1H), 8.01 (d, J = 2.7 Hz, 1H), 7.89 (dd, J = 9.0, 2.7 Hz, 1H), 7.19 (d, J = 9.0 Hz, 1H), 4.29 (t, J = 5.2 Hz, 2H), 3.19 – 2.96 (m, 2H), 2.53 (s, 6H); ^{13}C NMR (126 MHz, Methanol- d_4) δ 163.8, 154.0, 152.9, 147.5, 138.3, 131.4, 130.3, 125.9, 124.5 (q, J = 272.1 Hz), 119.7, 119.6, 118.4 (q, J = 31.5 Hz), 113.5, 82.6, 78.7, 66.5, 57.1, 44.3; HRMS (ESI) m/z calcd for $C_{19}H_{19}F_3N_3O_2$ [M + H]$^+$ 378.1424, found 378.1425.

5-Ethynyl-*N*-(4-((4-(2-hydroxyethyl)piperazin-1-yl)methyl)-3-(trifluoromethyl)phenyl)nicotinamide

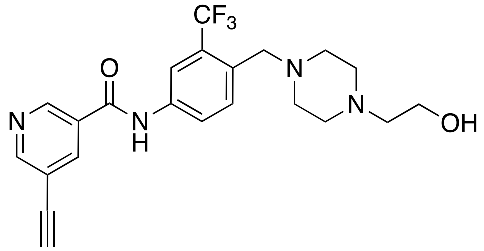

Pale yellow semi-solid (186 mg, 43%); ^{1}H NMR (500 MHz, Chloroform-d) δ 9.73 – 9.51 (m, 1H), 9.02 (s, 1H), 8.71 (s, 1H), 8.25 (s, 1H), 7.95 – 7.78 (m, 2H), 7.66 (d, J = 8.3 Hz, 1H), 3.84 (s, 2H), 3.66 – 3.49 (m, 4H), 3.28 (s, 1H), 2.62 – 2.41 (m, 8H); ^{13}C NMR (126 MHz, $CDCl_3$) δ 163.7, 154.8, 147.7, 138.4, 136.6, 133.8, 131.2, 129.9, 128.9(q, J = 30.2 Hz), 125.0(q, J = 274.6 Hz), 123.8, 119.4, 118.1, 82.2, 79.2, 59.2, 57.7, 57.6, 52.9, 52.9; HRMS (ESI) m/z calcd for $C_{22}H_{24}F_3N_4O_2$ [M + H]$^+$ 433.1851, found 433.1844.

***N*-(3-Chloro-4-((4-methylpiperazin-1-yl)methyl)phenyl)-5-ethynylnicotinamide**

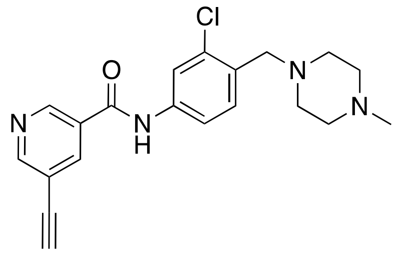

Pale yellow solid (133 mg, 67%); ^{1}H NMR (500 MHz, Chloroform-d) δ 8.96 (d, J = 2.2 Hz, 1H), 8.81 (s, 1H), 8.74 (d, J = 2.0 Hz, 1H), 8.19 (t, J = 2.1 Hz, 1H), 7.66 (d, J = 2.2 Hz, 1H), 7.45 – 7.35 (m, 2H), 3.54 (s, 2H), 3.29 (s, 1H), 2.60 – 2.35 (m, 8H), 2.25 (s, 3H); ^{13}C NMR (126 MHz, $CDCl_3$) δ 163.4, 154.9, 147.3, 138.2, 136.9, 134.5, 132.7, 131.0, 129.9, 121.5,

119.5, 118.9, 82.3, 79.2, 58.7, 55.1, 53.0, 46.0; HRMS (ESI$^+$): calcd. for $C_{20}H_{22}ClN_4O$ (MH$^+$) 369.1476, found 369.1474.

6-Ethynyl-*N*-(4-((4-methylpiperazin-1-yl)methyl)-3 (trifluoromethyl)phenyl)picolinamide

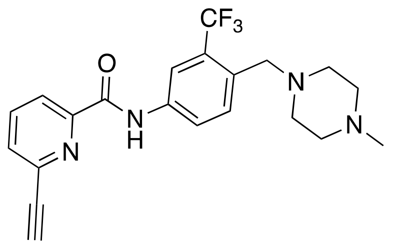

General procedure for Sonogashira coupling:

In a 25 mL round bottom flask containing bromo substrate (1 mmol), alkyne substrate (1.1 mmol), $PdCl_2(PPh_3)_2$ (5 mol%), PPh_3 (3 mol%), and CuI (3 mol%), anhydrous DMF (5 mL) and TEA (0.7 mL) was added under inert condition. Reaction mixture allowed to stir at 80 °C for an overnight. After completion reaction mixture was concentrated and extracted with ethyl acetate. Organic layer washed with brine solution (20 mL). Organic layer was passed through celite bad. Collected organic layer dried with sodium sulfate, concentrated and purified via silica gel column chromatography to yield the desired product using DCM/MeOH (95:5) as a solvent system.

(*S*)-*N*-(4-((hexahydropyrrolo[1,2-*a*]pyrazin-2(1*H*)-yl)methyl)-3-(trifluoromethyl)phenyl)-5-(imidazo[1,2-*b*]pyridazin-3-ylethynyl)nicotinamide

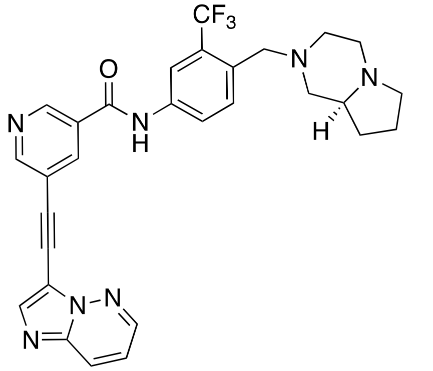

Off-white solid (73.6 mg, 54%); ^{1}H NMR (500 MHz, Methanol-*d*4) δ 9.08 – 9.03 (m, 1H), 8.96 – 8.88 (m, 1H), 8.62 (dd, *J* = 4.5, 1.6 Hz, 1H), 8.55 – 8.46 (m, 1H), 8.16 – 8.04 (m, 3H), 7.95 (dd, *J* = 8.6, 2.3 Hz, 1H), 7.76 (d, *J* = 8.4 Hz, 1H), 7.37 (dd, *J* = 9.2, 4.3 Hz, 1H), 3.79 – 3.66 (m, 2H), 3.59 (s, 2H), 3.25 – 3.09 (m, 2H), 3.00 – 2.92 (m, 1H), 2.87 – 2.77 (m, 1H), 2.68 – 2.57 (m, 2H), 2.56 – 2.48 (m, 1H), 2.48 – 2.37 (m, 1H), 2.21 – 2.10 (m, 1H), 1.98 – 1.82 (m, 4H); ^{13}C NMR (126 MHz, Methanol-*d*4) δ 164.0, 153.2, 147.4, 144.8, 140.1, 137.7, 137.6, 137.5, 132.8, 131.2, 130.4, 128.8 (q, *J* = 30.2 Hz), 125.2, 123.5, 123.2 (q, *J* = 273.4 Hz), 119.8, 119.4, 117.7, 112.2, 93.9, 79.8, 62.9, 62.8, 57.1, 55.4, 52.2, 50.7, 50.2, 26.0, 20.2; HRMS (ESI) m/z calcd for $C_{29}H_{27}F_3N_7O$ [M + H]$^+$ 546.2224, found 546.2219.

***N*-(4-((3-Hydroxy-3-methylazetidin-1-yl)methyl)-3-(trifluoromethyl)phenyl)-5-(imidazo[1,2-*b*]pyridazin-3-ylethynyl)nicotinamide(HSND-02)**

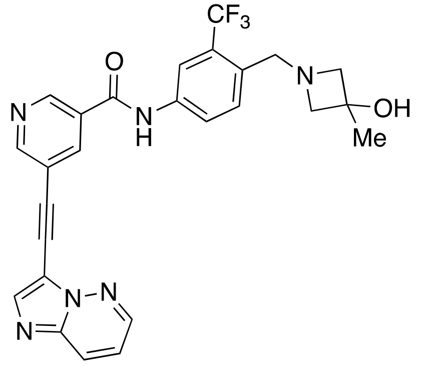

HSND-02

Pale yellow solid (59 mg, 47%); ^{1}H NMR (500 MHz, Methanol-d_4) δ 9.08 (d, J = 2.1 Hz, 1H), 8.94 (d, J = 2.0 Hz, 1H), 8.64 (dd, J = 4.6, 1.5 Hz, 1H), 8.55 (t, J = 2.2 Hz, 1H), 8.19 (d, J = 2.2 Hz, 1H), 8.15 – 8.09 (m, 2H), 8.00 (dd, J = 8.5, 2.3 Hz, 1H), 7.67 (d, J = 8.5 Hz, 1H), 7.38 (dd, J = 9.2, 4.4 Hz, 1H), 4.00 (s, 2H), 3.66 – 3.49 (m, 2H), 3.31 – 3.27 (m, 2H), 1.50 (s, 3H); ^{13}C NMR (126 MHz, Methanol-d_4) δ 164.1, 153.2, 147.5, 144.8, 140.1, 137.9, 137.7, 137.5, 131.1, 130.5, 130.4, 128.5 (q, J = 30.2 Hz), 125.2, 123.6, 123.1, 119.9, 119.5, 117.8, 112.2, 93.9, 79.8, 67.7, 67.2, 57.8, 24.6; HRMS (ESI) m/z calcd for $C_{26}H_{22}F_3N_6O_2$ [M + H]$^+$ 507.1750, found 507.1751.

(*R*)-*N*-(4-((2,4-Dimethylpiperazin-1-yl)methyl)-3-(trifluoromethyl)phenyl)-5-(imidazo[1,2-*b*]pyridazin-3-ylethynyl)nicotinamide

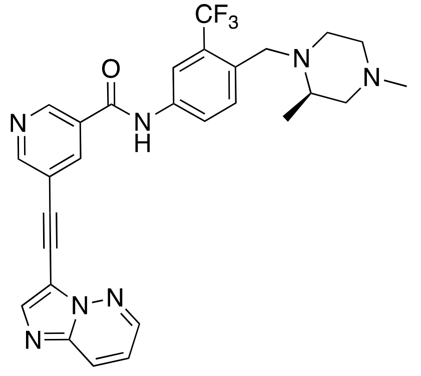

HSND-05

Pale yellow solid (76 mg, 57%); ^{1}H NMR (500 MHz, Methanol-d_4) δ 9.07 (d, J = 2.2 Hz, 1H), 8.91 (q, J = 2.3 Hz, 1H), 8.63 (dt, J = 3.0, 1.4 Hz, 1H), 8.52 (p, J = 2.1 Hz, 1H), 8.11 (ddt, J = 9.3, 5.9, 2.0 Hz, 3H), 7.94 (d, J = 8.6 Hz, 1H), 7.82 (d, J = 8.5 Hz, 1H), 7.37 (ddt, J = 9.2, 4.5, 1.3 Hz, 1H), 4.15 (d, J = 14.8 Hz, 1H), 3.34 (d, J = 11.1 Hz, 1H), 2.88 – 2.81 (m, 1H), 2.77 (d, J = 9.2 Hz, 1H), 2.73 – 2.65 (m, 1H), 2.58 (t, J = 7.0 Hz, 1H), 2.35 (s, 3H), 2.34 – 2.24 (m, 2H), 2.19 – 2.10 (m, 1H), 1.13 (s, 3H); ^{13}C NMR (126 MHz, Methanol-d_4) δ 164.0, 153.2, 147.5, 144.8, 140.1, 137.7, 137.5, 137.3, 134.2, 131.1, 130.5, 128.5 (q, J = 30.2 Hz), 125.4 (q,

J = 273.4 Hz), 125.2, 123.5, 119.9, 119.5, 117.6, 112.3, 93.9, 79.8, 61.8, 55.4, 54.6, 53.0, 44.2; HRMS (ESI) m/z calcd for $C_{28}H_{27}F_3N_7O$ $[M + H]^+$ 534.2223, found 534.2222.

(*S*)-*N*-(4-((2,4-Dimethylpiperazin-1-yl)methyl)-3-(trifluoromethyl)phenyl)-5-(imidazo[1,2-*b*]pyridazin-3-ylethynyl)nicotinamide

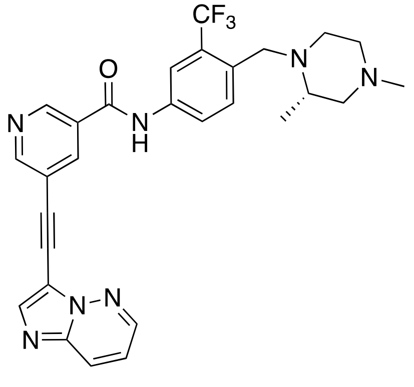

HSND-06

Pale yellow solid (86 mg, 65%); ^{1}H NMR (500 MHz, Methanol-d_4) δ 9.04 (d, J = 2.1 Hz, 1H), 8.88 (d, J = 2.0 Hz, 1H), 8.61 (dd, J = 4.5, 1.5 Hz, 1H), 8.49 (d, J = 2.2 Hz, 1H), 8.13 – 8.01 (m, 3H), 7.91 (dd, J = 8.5, 2.2 Hz, 1H), 7.80 (d, J = 8.5 Hz, 1H), 7.35 (dd, J = 9.2, 4.4 Hz, 1H), 4.13 (d, J = 14.7 Hz, 1H), 3.36 – 3.32 (m, 1H), 2.79 – 2.61 (m, 3H), 2.57 – 2.50 (m, 1H), 2.29 – 2.17 (m, 5H), 2.02 (t, J = 10.4 Hz, 1H), 1.10 (d, J = 6.2 Hz, 3H); ^{13}C NMR (126 MHz, MeOD) δ 163.9, 153.1, 147.4, 144.8, 140.1, 137.7, 137.5, 137.2, 134.3, 131.0, 130.4, 128.4 (q, J = 30.2 Hz), 125.4 (q, J = 274.6 Hz), 125.2, 123.5, 119.8, 119.4, 117.5, 112.2, 93.9, 79.8, 62.2, 55.6, 54.7, 53.1, 44.5; HRMS (ESI) m/z calcd for $C_{28}H_{27}F_3N_7O$ $[M + H]^+$ 534.2229, found 534.2222.

5-(Imidazo[1,2-*b*]pyridazin-3-ylethynyl)-*N*-(4-((4-isopropylpiperazin-1-yl)methyl)-3-(trifluoromethyl)phenyl)nicotinamide

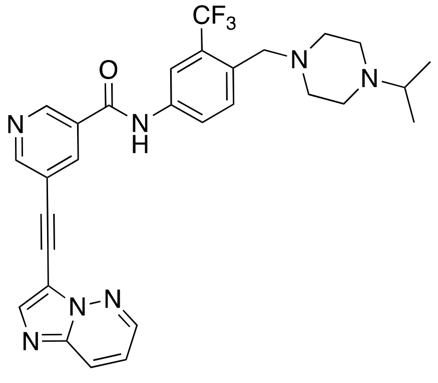

HSND-07

Off-white solid (77 mg, 56%); ^{1}H NMR (500 MHz, DMSO-d_6) δ 10.80 (s, 1H), 9.11 (d, J = 2.2 Hz, 1H), 8.99 (d, J = 2.0 Hz, 1H), 8.72 (dd, J = 4.5, 1.6 Hz, 1H), 8.56 (t, J = 2.2 Hz, 1H), 8.27 (d, J = 9.1 Hz, 2H), 8.21 (d, J = 2.4 Hz, 1H), 8.08 (dd, J = 8.5, 2.4 Hz, 1H), 7.76 (d, J = 8.5 Hz, 1H), 7.41 (dd, J = 9.2, 4.4 Hz, 1H), 3.68 (s, 2H), 3.51 – 3.44 (m, 1H), 3.39 (d, J = 12.8 Hz, 2H), 3.07 – 2.98 (m, 2H), 2.97 – 2.91 (m, 2H), 2.46 – 2.38 (m, 2H), 1.25 (d, J = 6.6 Hz, 6H); ^{13}C NMR (126 MHz, DMSO-d_6) δ 163.8, 154.1, 149.0, 145.6, 140.3, 139.4, 138.5, 137.6,

132.0, 131.8, 130.2, 128.1(q, J = 28.9 Hz), 126.7, 125.7 (q, J = 274.6 Hz), 124.0, 119.9, 119.0, 117.8, 111.6, 94.8, 81.1, 57.5, 56.8, 50.0, 49.0, 48.2, 17.0; HRMS (ESI) m/z calcd for $C_{29}H_{29}F_3N_7O$ [M + H]$^+$ 548.2380, found 548.2374.

(*R*)-*N*-(4-((3-(Dimethylamino)pyrrolidin-1-yl)methyl)-3-(trifluoromethyl)phenyl)-5-(imidazo[1,2-*b*]pyridazin-3-ylethynyl)nicotinamide (HSND-08)

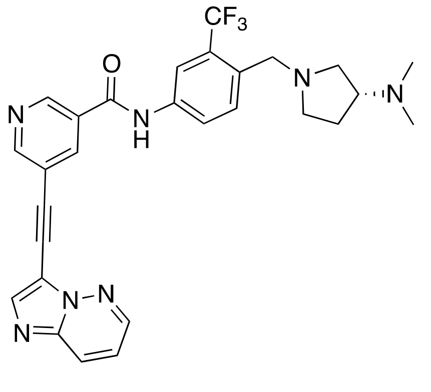

HSND-08

Pale yellow solid (85 mg, 64%); ^{1}H NMR (500 MHz, Methanol-d_4) δ 9.08 (d, J = 2.1 Hz, 1H), 8.93 (d, J = 2.0 Hz, 1H), 8.64 (dd, J = 4.5, 1.6 Hz, 1H), 8.54 (t, J = 2.1 Hz, 1H), 8.19 – 8.06 (m, 3H), 7.95 (dd, J = 8.5, 2.3 Hz, 1H), 7.76 (d, J = 8.5 Hz, 1H), 7.38 (dd, J = 9.2, 4.4 Hz, 1H), 3.77 (q, J = 14.3 Hz, 2H), 3.03 – 2.92 (m, 1H), 2.80 (dd, J = 9.4, 7.2 Hz, 1H), 2.72 – 2.60 (m, 2H), 2.50 (dd, J = 9.5, 6.5 Hz, 1H), 2.27 (s, 6H), 2.10 – 1.98 (m, 1H), 1.84 – 1.73 (m, 1H); ^{13}C NMR (126 MHz, Methanol-d_4) δ 164.0, 153.2, 147.4, 144.8, 137.7, 137.5, 137.4, 133.6, 131.1, 130.5, 128.4 (q, J = 28.9 Hz), 125.2, 123.5, 123.2 (q, J = 272.1 Hz), 119.9, 119.5, 117.6, 112.3, 93.9, 79.8, 65.1, 57.3, 55.2, 52.9, 42.0, 28.0; HRMS (ESI) m/z calcd for $C_{28}H_{27}F_3N_7O$ [M + H]$^+$ 534.2223, found 534.2222.

***N*-(4-((3-Hydroxy-3-methylazetidin-1-yl)methyl)-3-(trifluoromethyl)phenyl)-5-(imidazo[1,2-*b*]pyridazin-3-ylethynyl)nicotinamide (HSND-09)**

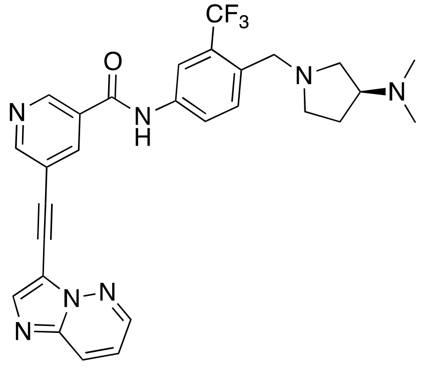

HSND-09

Pale yellow solid (81 mg, 61%); ^{1}H NMR (500 MHz, Methanol-d_4) δ 9.05 (s, 1H), 8.86 (s, 1H), 8.61 (dd, J = 4.5, 1.3 Hz, 1H), 8.49 (t, J = 2.0 Hz, 1H), 8.14 (s, 0H), 8.07 (d, J = 9.2 Hz, 2H), 7.94 (dd, J = 8.4, 2.3 Hz, 1H), 7.70 (d, J = 8.5 Hz, 1H), 7.65 (d, J = 12.1 Hz, 0H), 7.36 (dd, J = 9.2, 4.4 Hz, 1H), 3.77 (s, 2H), 3.71 – 3.63 (m, 1H), 2.94 – 2.81 (m, 2H), 2.80 – 2.68 (m, 7H),

2.50 (q, J = 7.9 Hz, 1H), 2.28 – 2.19 (m, 1H), 2.04 – 1.92 (m, 1H); ^{13}C NMR (126 MHz, Methanol-d_4) δ 163.9, 153.2, 147.5, 144.8, 137.7, 137.6, 137.5, 133.0, 131.2, 130.3, 128.3 (q, J = 31.5 Hz), 127.5, 125.3 (q, J = 273.4 Hz), 125.2, 123.6, 119.8, 119.5, 117.7, 94.0, 79.9, 65.1, 55.4, 54.6, 52.3, 40.5, 26.3; HRMS (ESI) m/z calcd for $C_{28}H_{27}F_3N_7O$ [M + H]$^+$ 534.2223, found 534.2223

5-(imidazo[1,2-*b*]pyridazin-3-ylethynyl)-*N*-(4-((4-methyl-1,4-diazepan-1-yl)methyl)-3-(trifluoromethyl)phenyl)nicotinamide (HSND10)

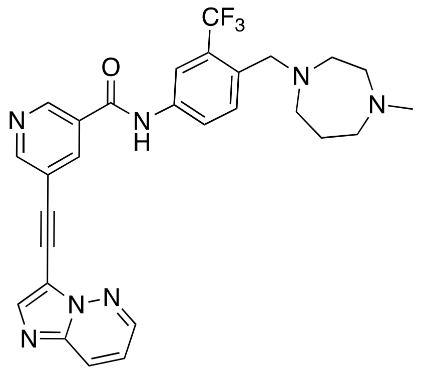

HSND-10

Off-white solid (67 mg, 50%); ^{1}H NMR (500 MHz, DMSO-d_6) δ 10.82 (s, 1H), 9.10 (d, J = 2.0 Hz, 1H), 8.96 (s, 1H), 8.70 (d, J = 4.3 Hz, 1H), 8.54 (d, J = 2.2 Hz, 1H), 8.25 (d, J = 6.1 Hz, 3H), 8.17 (s, 1H), 8.05 (d, J = 8.6 Hz, 1H), 7.81 (d, J = 8.5 Hz, 1H), 7.40 (dd, J = 9.2, 4.5 Hz, 1H), 3.73 (s, 2H), 3.12 – 3.08 (m, 2H), 3.05 – 3.00 (m, 2H), 2.78 – 2.73 (m, 2H), 2.67 – 2.63 (m, 2H), 2.62 (s, 3H), 1.89 (q, J = 5.8 Hz, 2H); ^{13}C NMR (126 MHz, DMSO-d_6) δ 163.8, 154.0, 148.9, 145.6, 140.3, 139.3, 138.2, 137.6, 133.6, 131.8, 130.2, 127.9 (q, J = 30.24 Hz), 126.6, 124.1, 123.6 (q, J = 274.6 Hz), 119.9, 119.0, 117.8, 111.5, 94.8, 81.0, 57.6, 56.9, 55.3, 53.9, 50.9, 44.8, 24.9; HRMS (ESI) m/z calcd for $C_{28}H_{27}F_3N_7O$ [M + H]$^+$ 534.2223, found 534.2222.

***N*-(4-(2-(Dimethylamino)ethoxy)-3-(trifluoromethyl)phenyl)-5-(imidazo[1,2-*b*]pyridazin-3-ylethynyl)nicotinamide (HSND-11)**

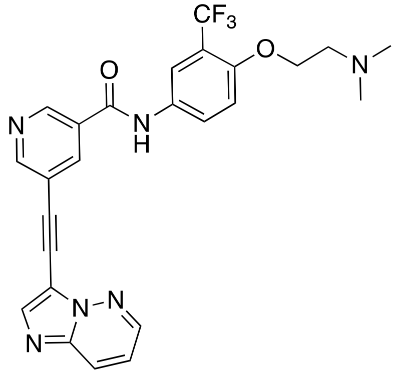

HSND-11

Pale yellow solid (73 mg, 59%); ^{1}H NMR (500 MHz, Methanol-d_4) δ 9.07 (d, *J* = 2.2 Hz, 1H), 8.93 (d, *J* = 2.0 Hz, 1H), 8.64 (dd, *J* = 4.5, 1.6 Hz, 1H), 8.53 (t, *J* = 2.1 Hz, 1H), 8.15 – 8.07 (m, 2H), 8.03 (d, *J* = 2.7 Hz, 1H), 7.92 (dd, *J* = 9.0, 2.7 Hz, 1H), 7.38 (dd, *J* = 9.2, 4.4 Hz, 1H), 7.22 (d, *J* = 9.0 Hz, 1H), 4.24 (t, *J* = 5.4 Hz, 2H), 2.84 (t, *J* = 5.4 Hz, 2H), 2.38 (s, 6H); ^{13}C NMR (126 MHz, Methanol-d_4) δ 163.9, 153.3, 153.1, 147.4, 144.8, 140.1, 137.7, 137.5, 131.2, 130.6, 125.9, 125.2, 124.5 (q, *J* = 272.1), 119.9, 119.7, 119.4, 118.5 (q, *J* = 30.2 Hz), 113.4, 112.3, 93.9, 79.7, 67.3, 57.41, 44.7; HRMS (ESI) m/z calcd for $C_{25}H_{22}F_3N_6O_2$ [M + H]$^+$ 495.1750, found 495.1751.

***N*-(4-((4-(2-Hydroxyethyl)piperazin-1-yl)methyl)-3-(trifluoromethyl)phenyl)-5-(imidazo[1,2-*b*]pyridazin-3-ylethynyl)nicotinamide (HSND-12)**

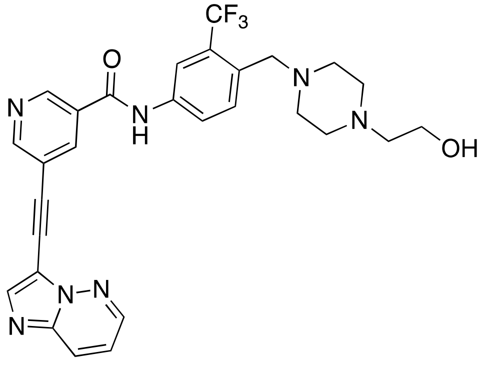

HSND-12

Pale yellow solid (62 mg, 45%); ^{1}H NMR (500 MHz, Methanol-d_4) δ 9.08 (d, *J* = 2.2 Hz, 1H), 8.93 (d, *J* = 2.0 Hz, 1H), 8.64 (dd, *J* = 4.4, 1.6 Hz, 1H), 8.54 (t, *J* = 2.1 Hz, 1H), 8.15 (d, *J* = 2.3 Hz, 1H), 8.12 – 8.08 (m, 2H), 7.95 (dd, *J* = 8.5, 2.3 Hz, 1H), 7.79 (d, *J* = 8.5 Hz, 1H), 7.38 (dd, *J* = 9.2, 4.4 Hz, 1H), 3.70 (t, *J* = 6.0 Hz, 2H), 3.67 (s, 2H), 2.69 – 2.54 (m, 10H); ^{13}C NMR (126 MHz, Methanol-d_4) δ 164.1, 153.2, 147.4, 144.8, 140.1, 137.7, 137.5, 133.0, 131.2, 130.5, 128.9 (q, *J* = 30.24 Hz), 125.3 (q, *J* = 274.6 Hz), 125.2, 123.5, 119.9, 119.4, 117.7, 112.2, 93.9, 79.8, 59.7, 58.0, 57.5, 53.0, 52.1; HRMS (ESI) m/z calcd for $C_{28}H_{27}F_3N_7O_2$ [M + H]$^+$ 550.2172, found 550.2165.

***N*-(3-Chloro-4-((4-methylpiperazin-1-yl)methyl)phenyl)-5-(imidazo[1,2-*b*]pyridazin-3-ylethynyl)nicotinamide (HSND-13)**

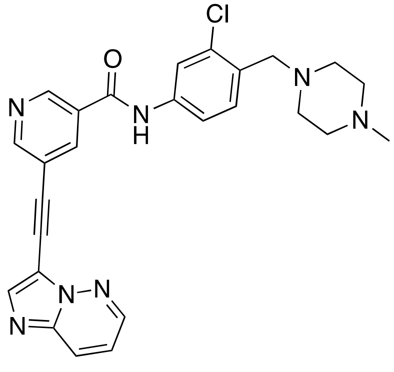

HSND-13

Off-white solid (69 mg, 57%); ^{1}H NMR (500 MHz, Methanol-*d*4) δ 9.06 (d, *J* = 2.2 Hz, 1H), 8.94 (d, *J* = 2.0 Hz, 1H), 8.64 (dd, *J* = 4.4, 1.6 Hz, 1H), 8.53 (t, *J* = 2.1 Hz, 1H), 8.13 – 8.08 (m, 2H), 7.93 (d, *J* = 2.2 Hz, 1H), 7.63 (dd, *J* = 8.4, 2.2 Hz, 1H), 7.48 (d, *J* = 8.4 Hz, 1H), 7.39 (dd, *J* = 9.2, 4.5 Hz, 1H), 3.66 (s, 2H), 2.59 (s, 8H), 2.33 (s, 3H); ^{13}C NMR (126 MHz, Methanol-*d*4) δ 164.0, 153.1, 147.4, 144.8, 140.1, 138.5, 137.7, 137.5, 134.3, 131.2, 131.0, 130.6, 125.2, 121.0, 119.9, 119.5, 118.7, 112.3, 93.9, 79.7, 58.1, 54.3, 51.9, 44.3; HRMS (ESI) m/z calcd for $C_{26}H_{25}ClN_7O$ $[M + H]^+$ 486.1803, found 486.1799.

Methyl 6-((trimethylsilyl)ethynyl)picolinate

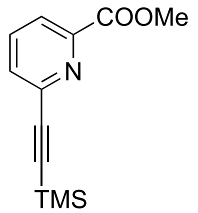

methyl 6-bromopicolinate

To a solution of methyl 6-bromopicolinate (0.5 g, 2.31 mmol) in THF (10 mL), $Pd(PPh_3)_2Cl_2$ (81 mg, 5 mol%,), CuI (13 mg, 3 mol%) was added. Reaction degassed and filled with argon followed by addition of triethylamine (5 mL) and ethynyltrimethylsilane (5 equiv). Reaction was allowed to stir at 60 °C for 6h under inert condition. After completion reaction was concentrated and extracted with ethyl acetate (100 mL) and water (50 mL X 2). Organic layer washed with brine, dried over sodium sulphate and concentrated. Obtained dark residue was purified via silica gel column chromatography(Hexanes/Ethylacetate 95:5 to 90:10) to give the desired product as an pale gray solid.

Off-white solid (296 mg, 55%); ^{1}H NMR (500 MHz, Methanol-*d*4) δ 8.08 (dd, *J* = 7.8, 1.1 Hz, 1H), 7.97 (td, *J* = 7.8, 1.0 Hz, 1H), 7.71 (dd, *J* = 7.8, 1.1 Hz, 1H), 4.85 (s, 2H), 3.96 (d, *J* = 1.0 Hz, 3H), 0.27 (s, 6H); ^{13}C NMR (126 MHz, Methanol-d4) δ 164.7, 147.8, 142.8, 138.0, 130.4, 124.2, 102.4, 95.6, 51.9, -1.8; HRMS (ESI) m/z calcd for $C_{12}H_{14}NO_2Si[M]^+$ 233.0872, found 233.0866.

6-Ethynylpicolinic acid

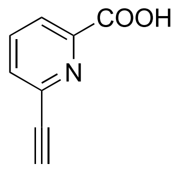

To a solution of methyl 6-((trimethylsilyl)ethynyl)picolinate (250 mg, 1.07 mmol) in methanol (4 mL)was added 2 N aqueous sodium hydroxide (1 mL) at 0 °C and reaction was allowed to stir at room temperature for 4h. After completion, reaction was acidified with aqueous hydrochloric acid. Reaction mixture was concentrated and extracted with ethyl acetate (100 mL X2) and water (50 mL X 2). Organic layer collected, dried over sodium sulphate, concentrated which gave desired product as light brown solid (134 mg, 85%); ^{1}H NMR (500s MHz, Methanol-*d*4) δ 8.12 (d, *J* = 7.8 Hz, 1H), 7.98 (t, *J* = 7.9 Hz, 1H), 7.75 (d, *J* = 7.8 Hz,

1H), 3.82 (s, 1H); ^{13}C NMR (126 MHz, Methanol-d_4) δ 165.6, 148.4, 142.3, 138.07, 130.5, 124.3, 81.2, 79.1. HRMS (ESI) m/z calcd for $C_8H_5NO_2$ [M + H]$^+$ 148.0399, found 148.0392.

6-(Imidazo[1,2-*b*]pyridazin-3-ylethynyl)-*N*-(4-((4-methylpiperazin-1-yl)methyl)-3-(trifluoromethyl)phenyl)picolinamide (HSND-23)

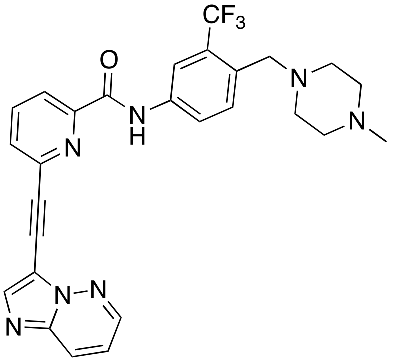

HSND-23

From 0.25 mmol

Pale yellow solid (82 mg, 63%); ^{1}H NMR (500 MHz, Methanol-d_4) δ 8.67 (dd, J = 4.4, 1.3 Hz, 1H), 8.29 (d, J = 2.2 Hz, 1H), 8.21 (dd, J = 7.9, 1.1 Hz, 1H), 8.18 – 8.12 (m, 2H), 8.08 (d, J = 7.8 Hz, 1H), 8.02 (dd, J = 8.5, 2.3 Hz, 1H), 7.88 (dd, J = 7.8, 1.0 Hz, 1H), 7.78 (d, J = 8.5 Hz, 1H), 7.40 (dd, J = 9.2, 4.4 Hz, 1H), 3.66 (s, 2H), 2.54 (bs, 8H), 2.31 (s, 3H); ^{13}C NMR (126 MHz, Methanol-d_4) δ 162.5, 150.1, 144.9, 141.4, 138.3, 137.1, 132.8, 131.2, 129.7, 128.9 (q, J = 30.2 Hz), 125.3, 123.3, 123.2 (q, J = 267.1 Hz), 121.7, 119.6, 117.4, 96.9, 76.1, 57.4, 54.5, 52.2, 44.5; HRMS (ESI) m/z calcd for $C_{27}H_{25}F_3N_7O$ [M + H]$^+$ 520.2067, found 520.2065.

II. References

1. Larocque, E.; Chu, E. F. Y.; Naganna, N.; Sintim, H. O. Nicotinamide–Ponatinib Analogues as Potent Anti-CML and Anti-AML Compounds. *ACS omega* ***2020***, *5*, 2690-2698.
2. Wang, M.; Naganna, N.; Sintim, H. O. Identification of Nicotinamide Aminonaphthyridine Compounds as Potent Ret Kinase Inhibitors and Antitumor Activities against Ret Rearranged Lung Adenocarcinoma. *Bioorg. Chem.* **2019**, *90*, 103052.
3. Naganna, N.; Opoku-Temeng, C.; Choi, E. Y.; Larocque, E.; Chang, E. T.; Carter-Cooper, B. A.; Wang, M.; Torregrosa-Allen, S. E.; Elzey, B. D.; Lapidus, R. G.; Sintim, H. O. Amino Alkynylisoquino- line and Alkynylnaphthyridine Compounds Potently Inhibit Acute Myeloid Leukemia Proliferation in Mice. *EBioMedicine* **2019**, *40*, 231−239.
4. Wenge, Z.; Xiaotian, Z.; Xianming, D.; Lei, W. Preparation of pyrazine derivatives and related heterocycles as kinase inhibitors for the treatment of cancer and other kinase-mediated diseases. **2020**, PCT Int. WO 2020206583 A1.

III. HPLC data

Table S3. HPLC Purity

**Compound**[method]	**Retention Time**	**% Purity area**
**HSN748**[a]	11.16	99
**HSN608**[b]	11.38	100
**HSN431**[e]	19.9	99
**HSND01**[a]	11.4	95
**HSND02**[b]	11.2	100
**HSND05**[d]	24.3	95
**HSND06**[b]	11.3	97
**HSND07**[a]	11.5	100
**HSND08**[a]	11.3	100
**HSND09**[e]	15.5	100
**HSND10**[e]	12.0	96
**HSND11**[a]	10.0	98
**HSND12**[c]	12.9	100
**HSND13**[b]	11.1	100
**HSND23**[a]	11.5	100

[a] UV detection wavelength 280 nm, Agilent Eclipse instrument; C18 column (3 µm, 4.6 × 100 mm^2); method: 0 → 5 min 50% B, 5 → 10 min 50 to 100% B, 10 →15 min 100% B (A: 0.1% NH_4OH in H_2O, B: MeOH). 25 °C.

[b] UV detection wavelength 254 nm, Agilent Eclipse instrument; C18 column (3 µm, 4.6 × 100 mm^2); method: 0 → 5 min 50% B, 5 → 10 min 50 to 100% B, 10 →15 min 100% B (A: 0.1% NH_4OH in H_2O, B: MeOH). 25 °C.

[c] UV detection wavelength 254 nm, Agilent Eclipse instrument; C18 column (3 µm, 4.6 × 100 mm^2); method: 0 → 10 min 50% B, 10 → 15 min 50 to 100% B, 15 →20 min 100% B (A: 0.1% NH_4OH in H_2O, B: MeOH). 25 °C.

[d] UV detection wavelength 280 nm, Agilent Eclipse instrument; C18 column 5C_{18}-MS-II COSMOSIL (4.6ID × 250 mm); method: 0 → 10 min 50% B, 10 → 12 min 50 to 90% B, 12 →25 min 90% B, 25 → 30 min 90 to 50% B (A: 0.1% NH_4OH in H_2O, B: MeOH), 25 °C.

[e] UV detection wavelength 280 nm, Agilent Eclipse instrument; C18 column 5C18-MS-II COSMOSIL (4.6ID × 250 mm); method: 0 → 5 min 50% B, 5 → 8 min 50 to 90% B, 8 →30 min 90% B (A: 0.1% NH_4OH in H_2O, B: MeOH), 25 °C.

HPLC chromatogram for compounds

Compound HSN748

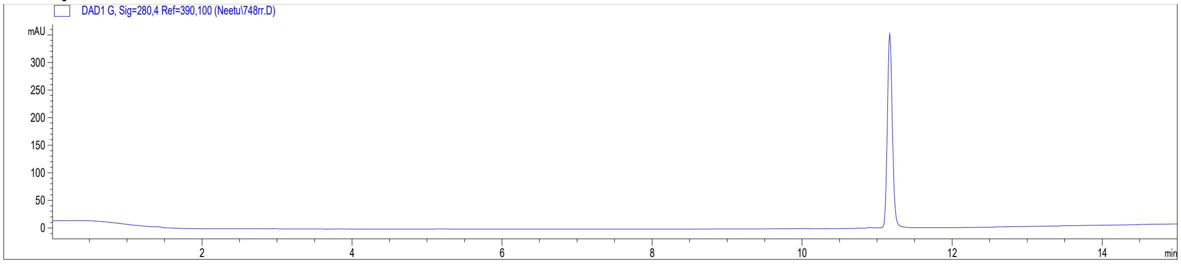

Compound HSN608

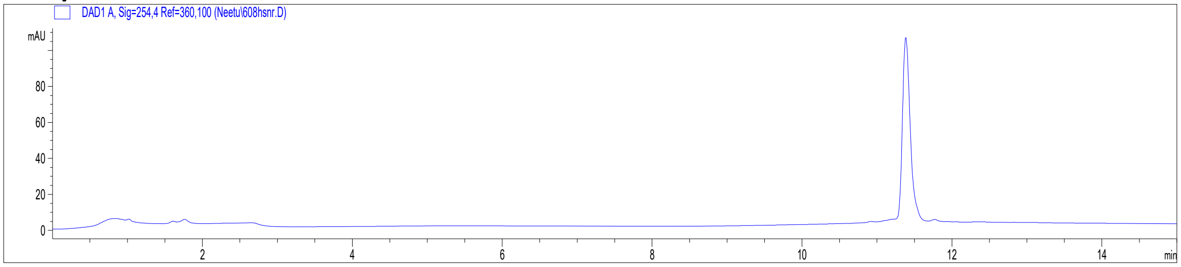

Compound HSN431

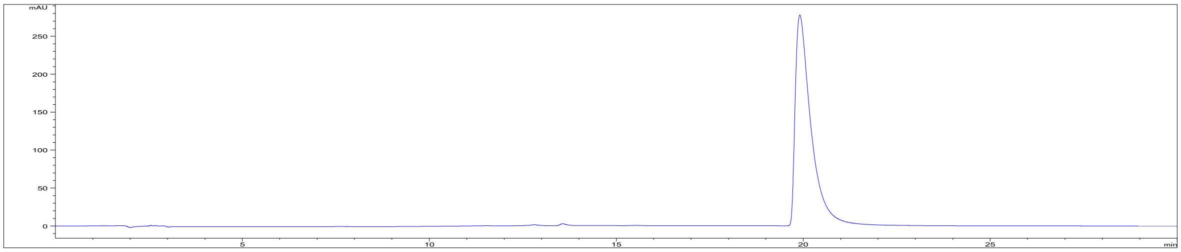

Compound HSND01

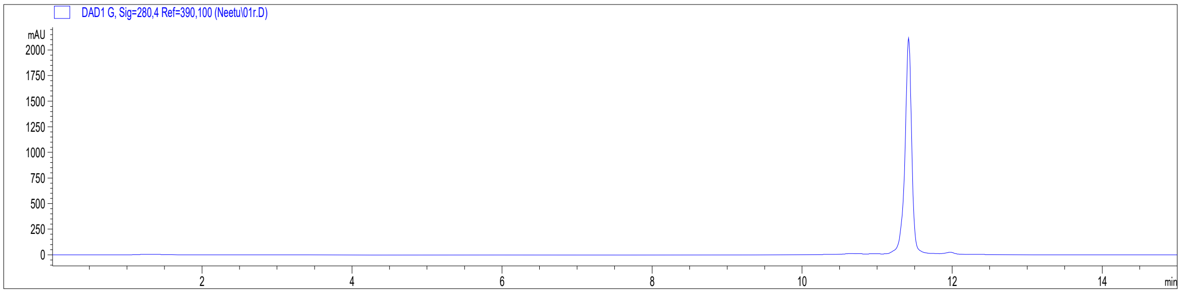

Compound HSND02

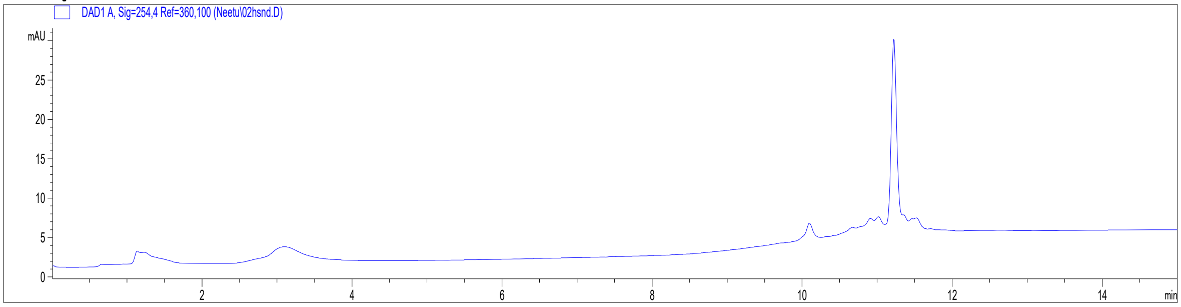

Compound HSND05

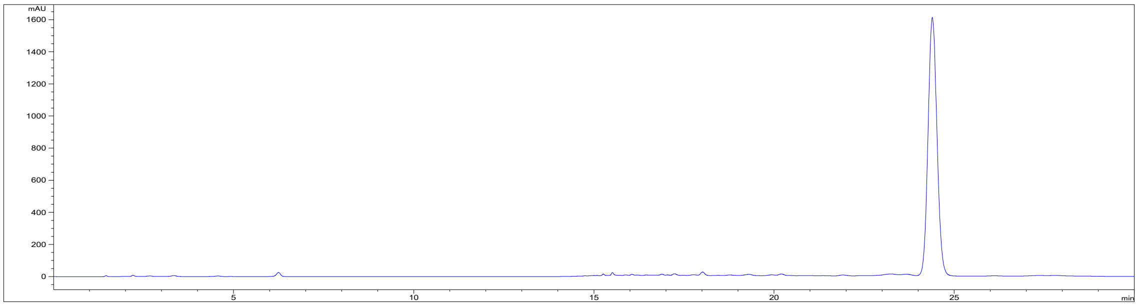

Compound HSND06

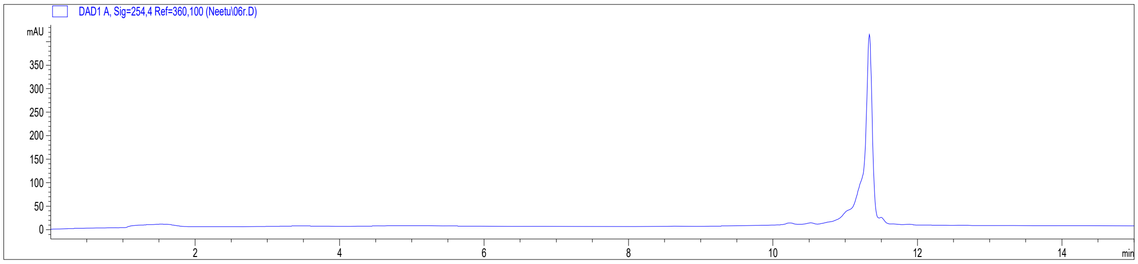

Compound HSND07

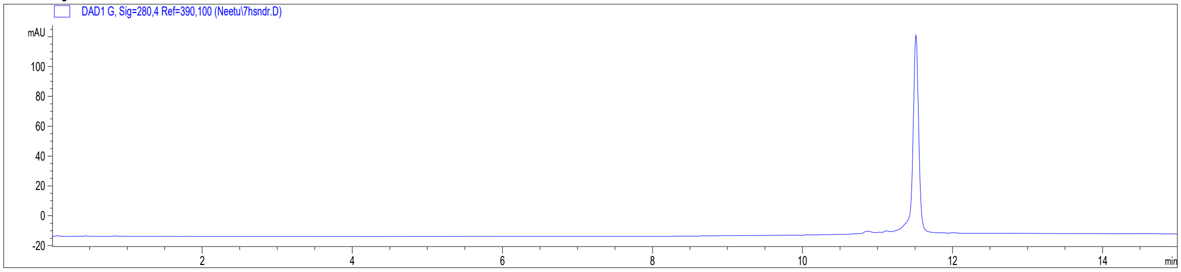

Compound HSND08

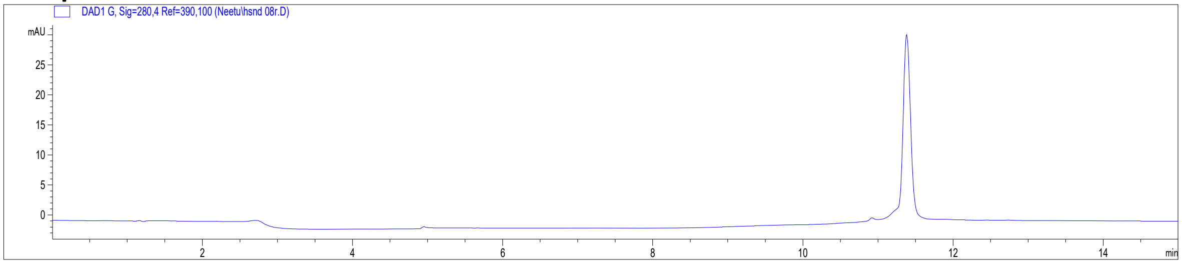

Compound HSND09

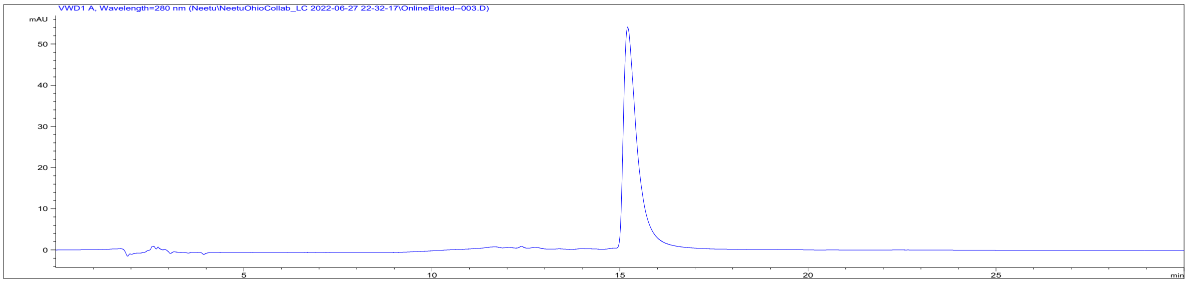

Compound HSND10

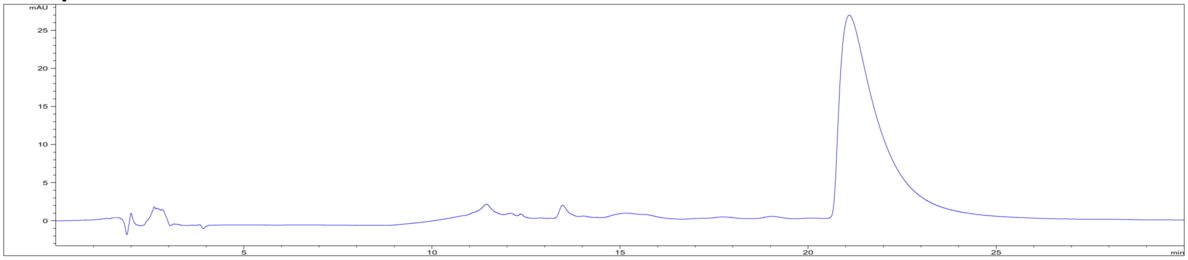

Compound HSND11

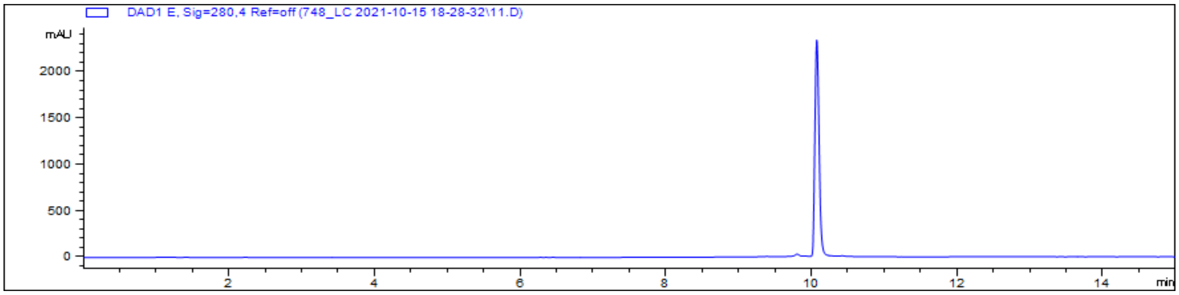

Compound HSND12

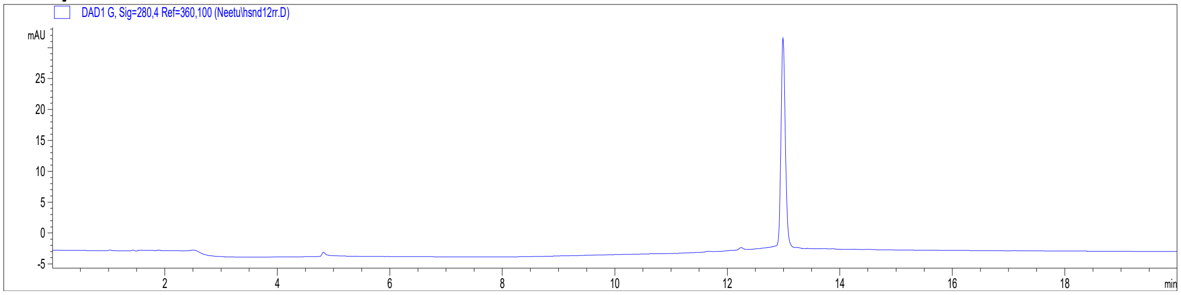

Compound HSND13

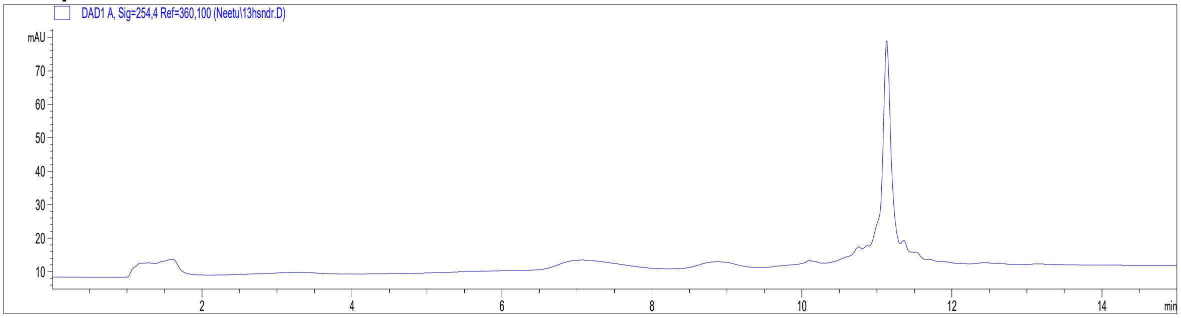

Compound HSND23

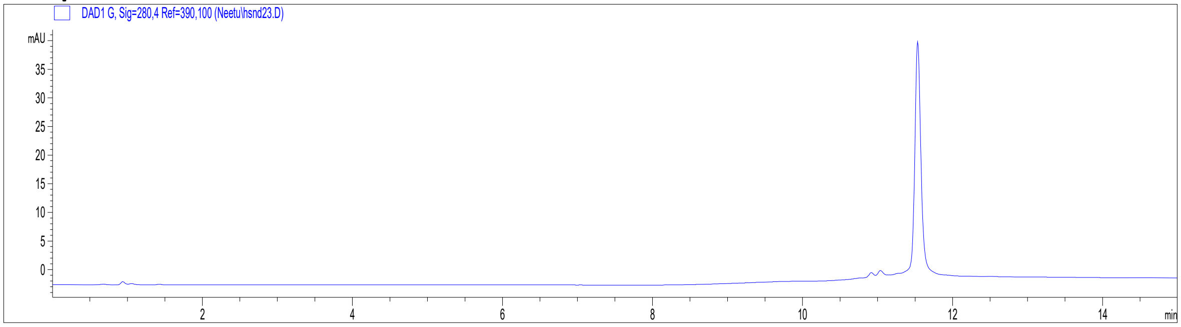

^{1}H and ^{13}C spectra's of analogs

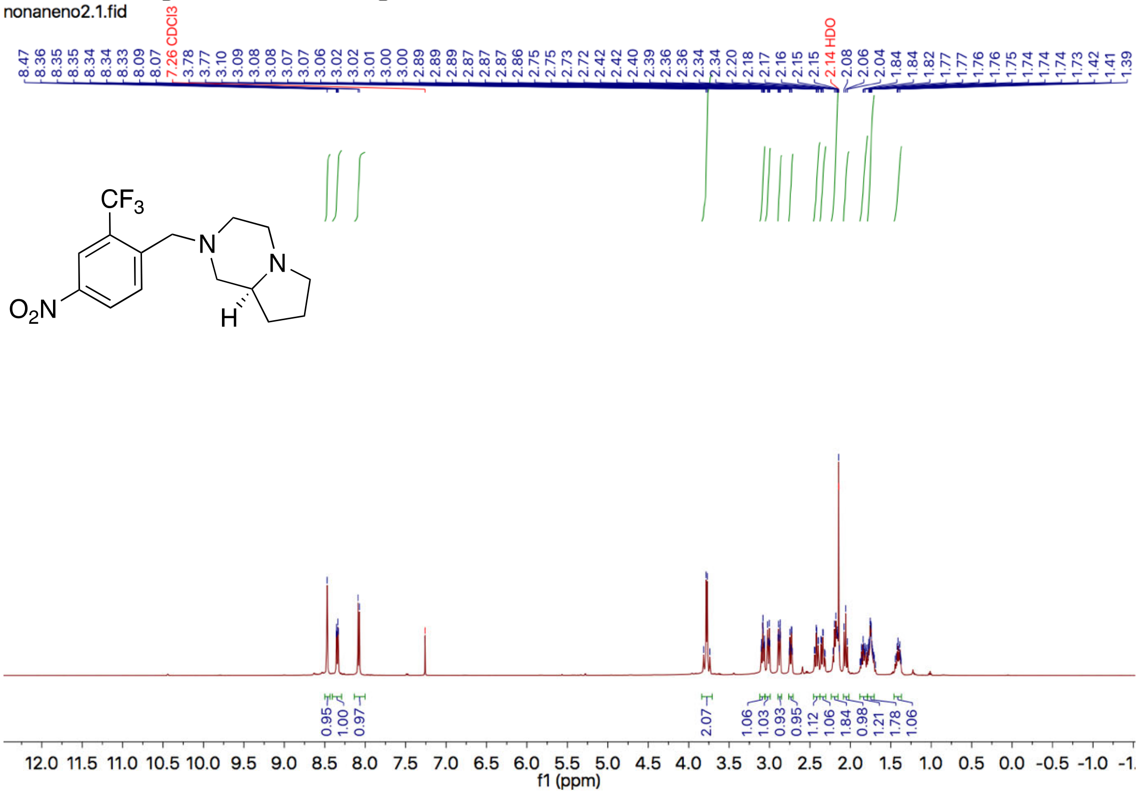

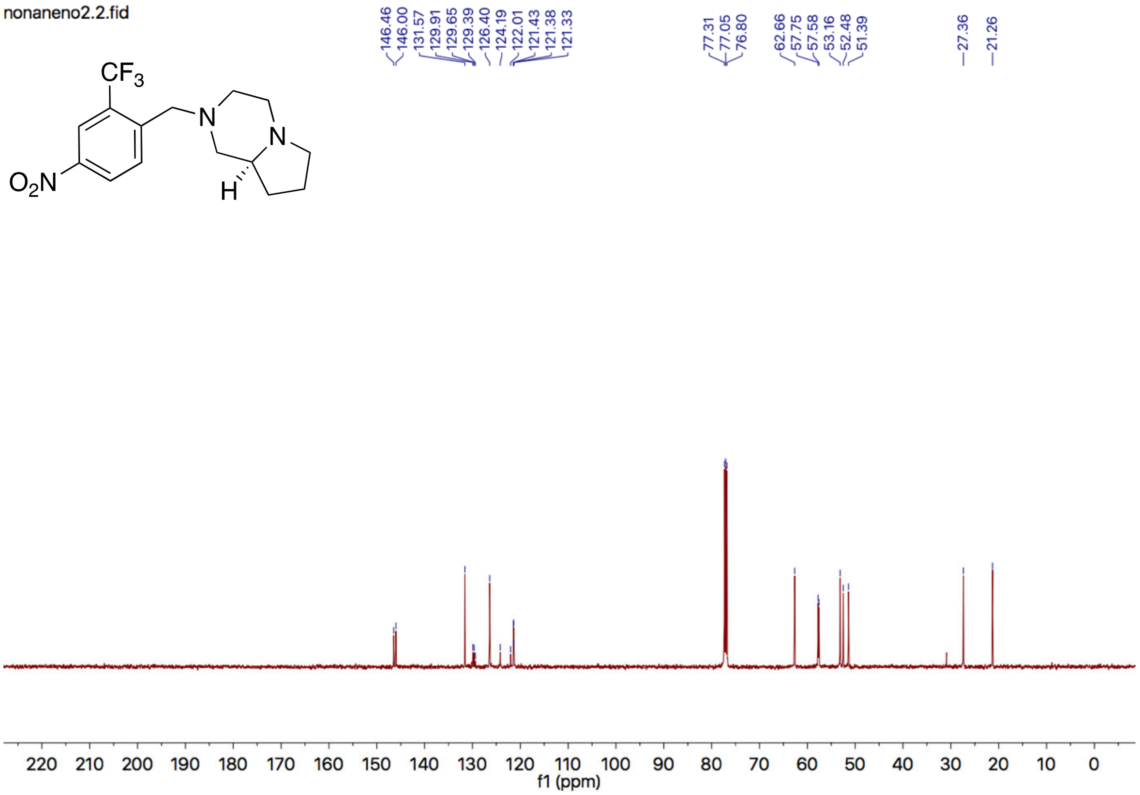

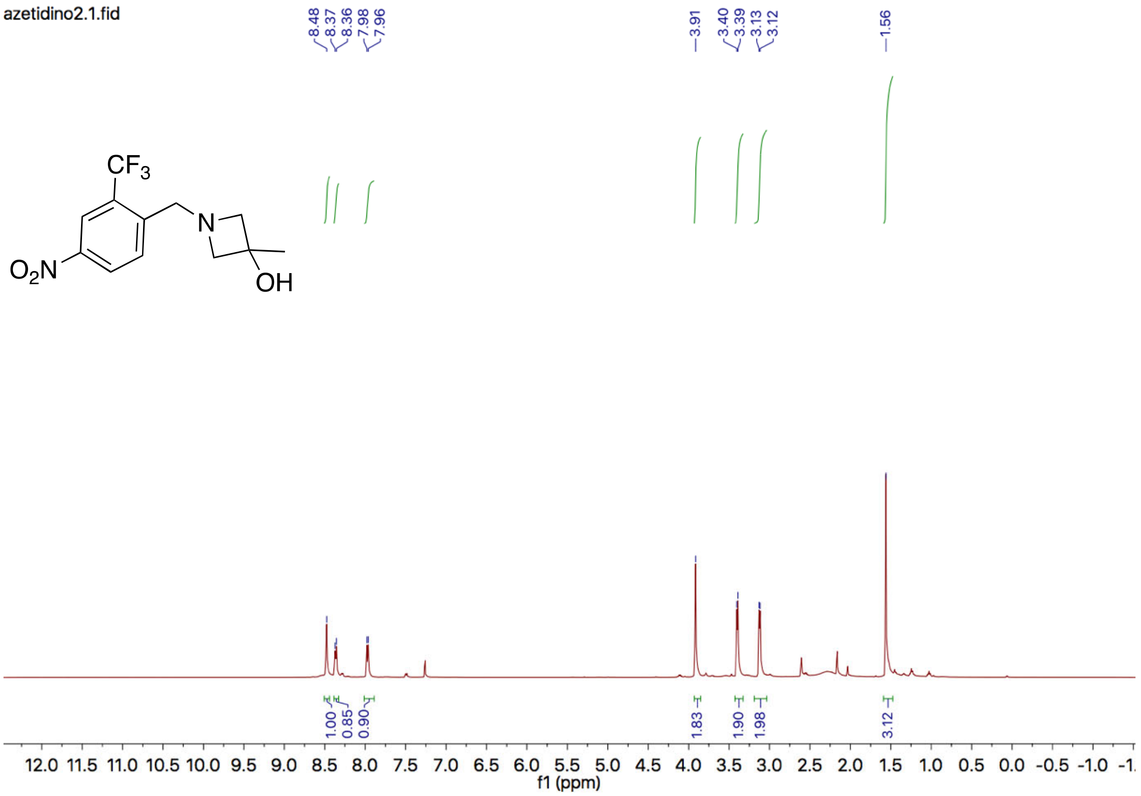
azetidino2.1.fid
8.48
8.37
8.36
7.98
7.96
3.91
3.40
3.39
3.13
3.12
1.56
CF3
O2N
N
OH
1.00
0.85
0.90
1.83
1.90
1.98
3.12
12.0 11.5 11.0 10.5 10.0 9.5 9.0 8.5 8.0 7.5 7.0 6.5 6.0 5.5 5.0 4.5 4.0 3.5 3.0 2.5 2.0 1.5 1.0 0.5 0.0 -0.5 -1.0
f1 (ppm)

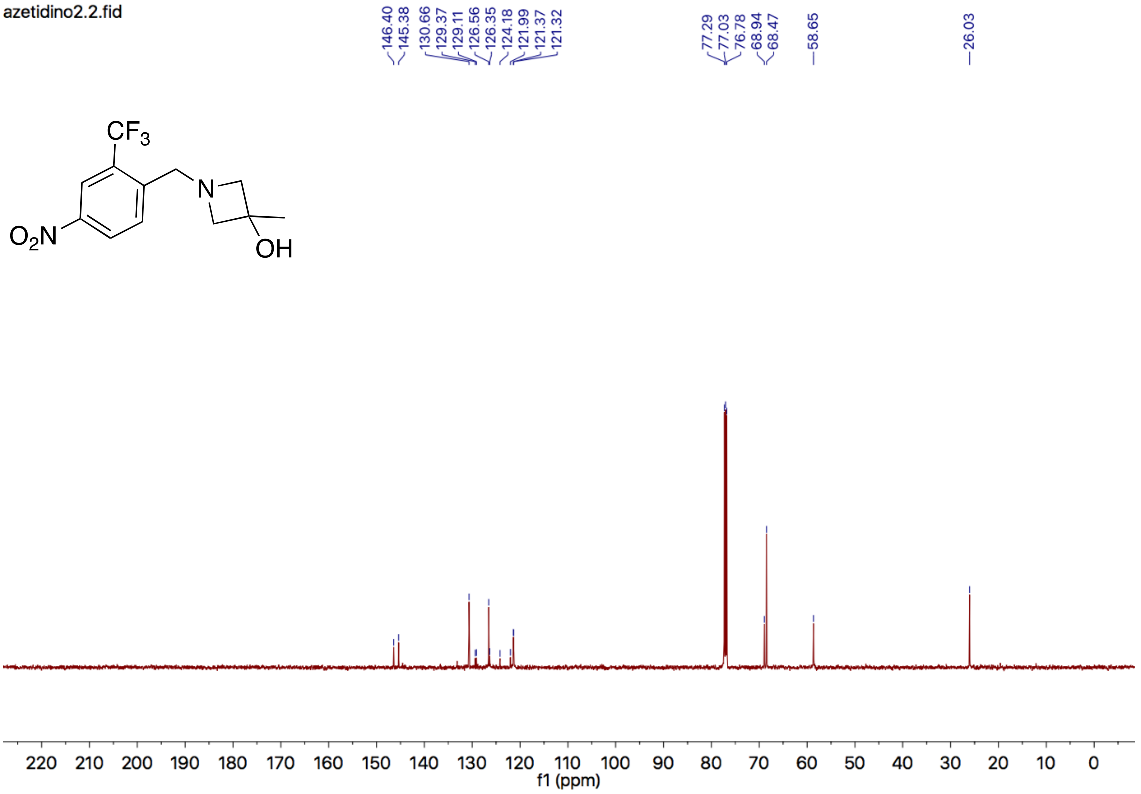
azetidino2.2.fid
146.40
145.38
130.66
129.37
129.11
126.56
126.35
124.18
121.99
121.37
121.32
77.29
77.03
76.78
68.94
68.47
58.65
26.03
CF3
O2N
N
OH
220 210 200 190 180 170 160 150 140 130 120 110 100 90 80 70 60 50 40 30 20 10 0
f1 (ppm)

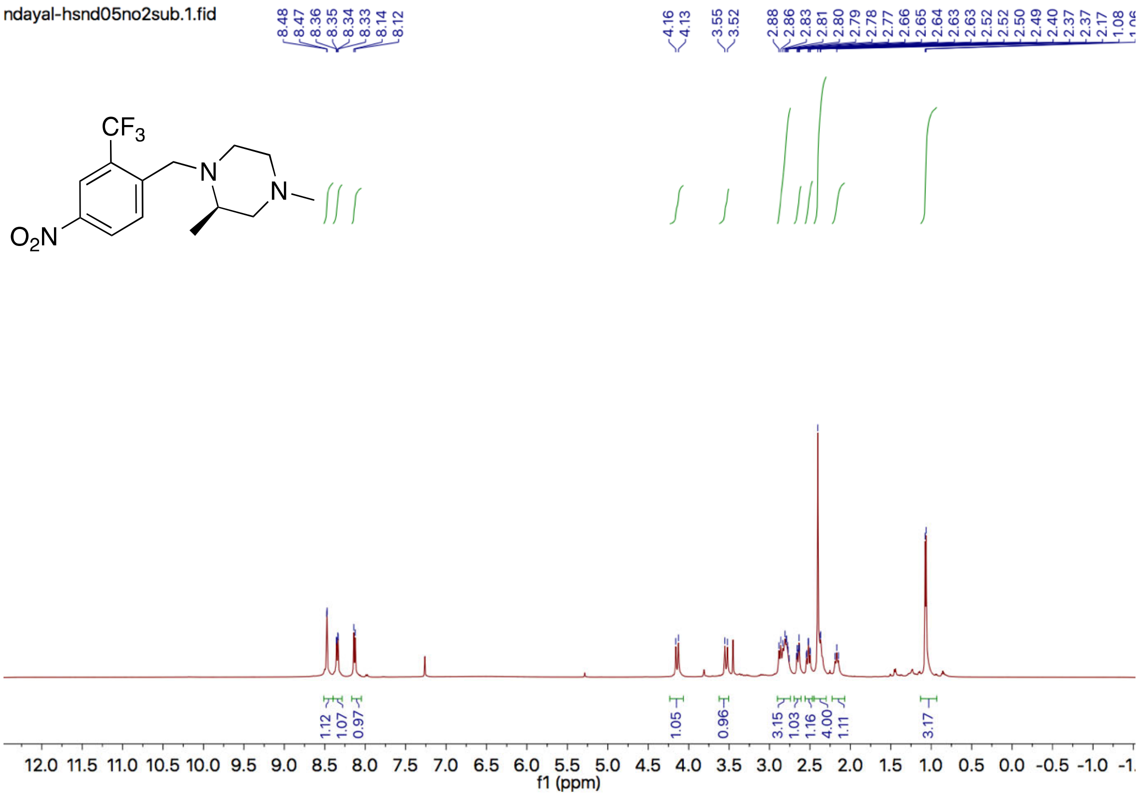
ndayal-hsnd05no2sub.1.fid
8.48
8.47
8.36
8.35
8.34
8.33
8.14
8.12
4.16
4.13
3.55
3.52
2.88
2.86
2.83
2.81
2.80
2.79
2.78
2.77
2.66
2.65
2.64
2.63
2.63
2.52
2.52
2.50
2.49
2.40
2.37
2.37
2.17
1.08
CF3
N
N
O2N
1.12
1.07
0.97
1.05
0.96
3.15
1.03
1.16
4.00
1.11
3.17
f1 (ppm)

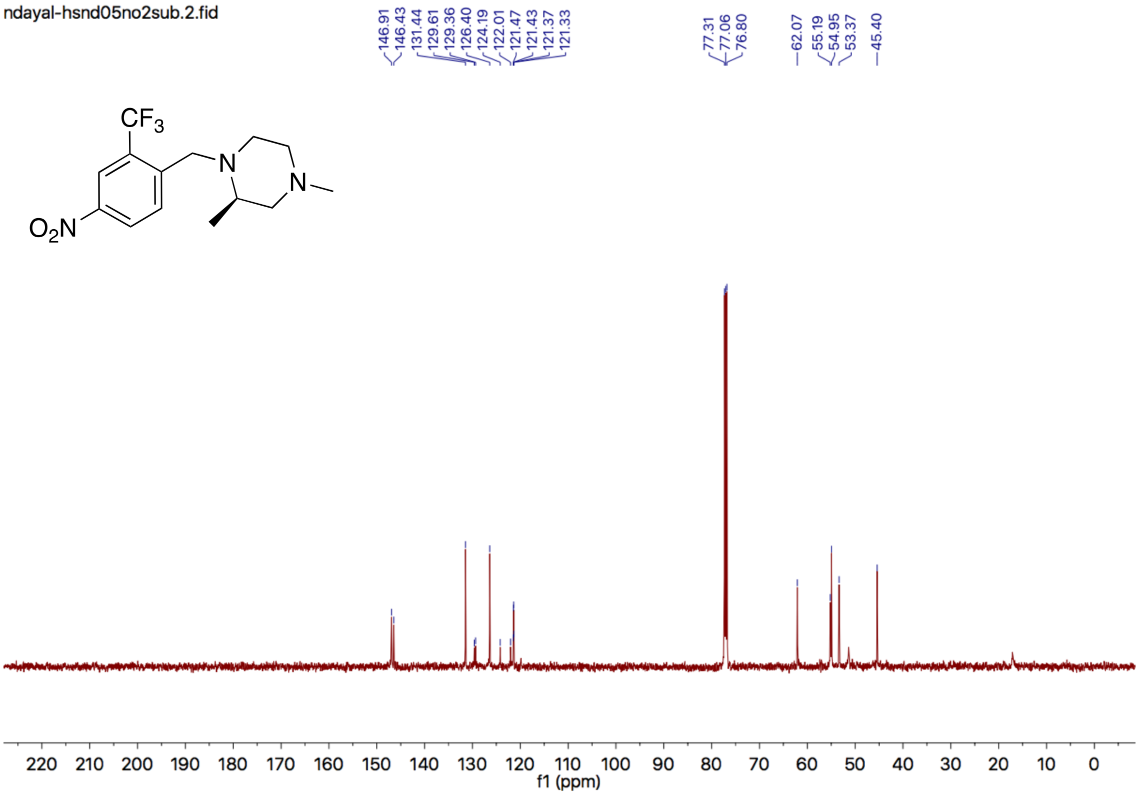
ndayal-hsnd05no2sub.2.fid
146.91
146.43
131.44
129.61
129.36
126.40
124.19
122.01
121.47
121.43
121.37
121.33
77.31
77.06
76.80
62.07
55.19
54.95
53.37
45.40
CF3
N
N
O2N
f1 (ppm)

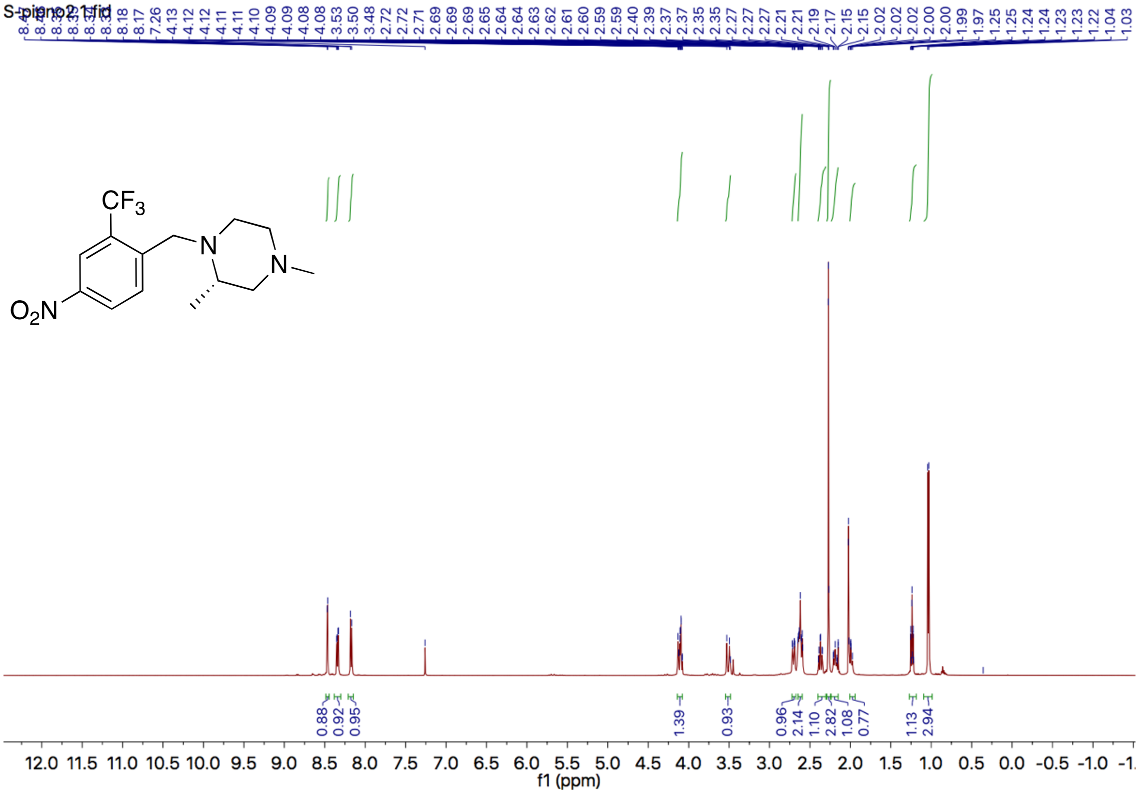
S-pipno2.1.fid
8.18 8.17 7.26 4.13 4.12 4.12 4.11 4.11 4.10 4.09 4.09 4.08 4.08 3.53 3.50 3.48 2.72 2.72 2.71 2.69 2.69 2.69 2.65 2.64 2.64 2.63 2.62 2.61 2.60 2.59 2.59 2.40 2.39 2.37 2.37 2.35 2.35 2.27 2.27 2.27 2.21 2.21 2.19 2.17 2.15 2.15 2.02 2.02 2.02 2.00 2.00 1.99 1.97 1.25 1.25 1.24 1.24 1.23 1.23 1.22 1.04 1.03
CF3
N
N
O2N
0.88 0.92 0.95 1.39 0.93 0.96 2.14 1.10 2.82 1.08 0.77 1.13 2.94
f1 (ppm)

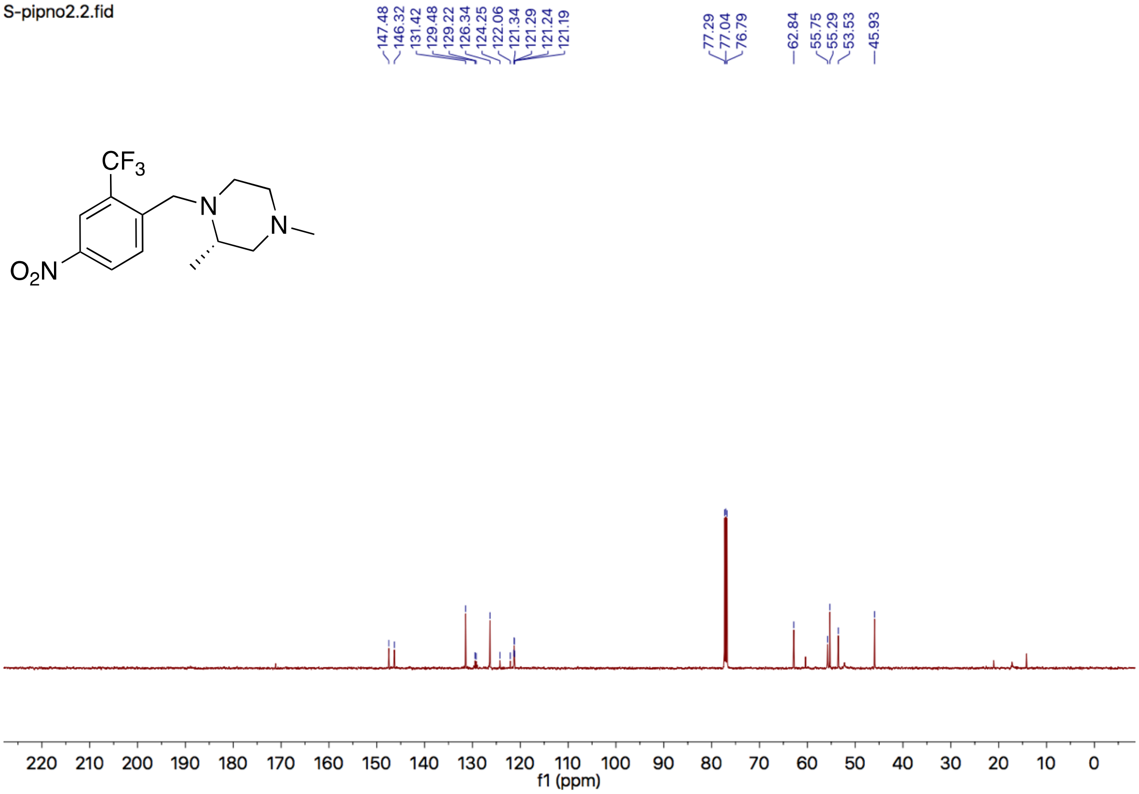
S-pipno2.2.fid
147.48 146.32 131.42 129.48 129.22 126.34 124.25 122.06 121.34 121.29 121.24 121.19
77.29 77.04 76.79
62.84 55.75 55.29 53.53 45.93
CF3
N
N
O2N
f1 (ppm)

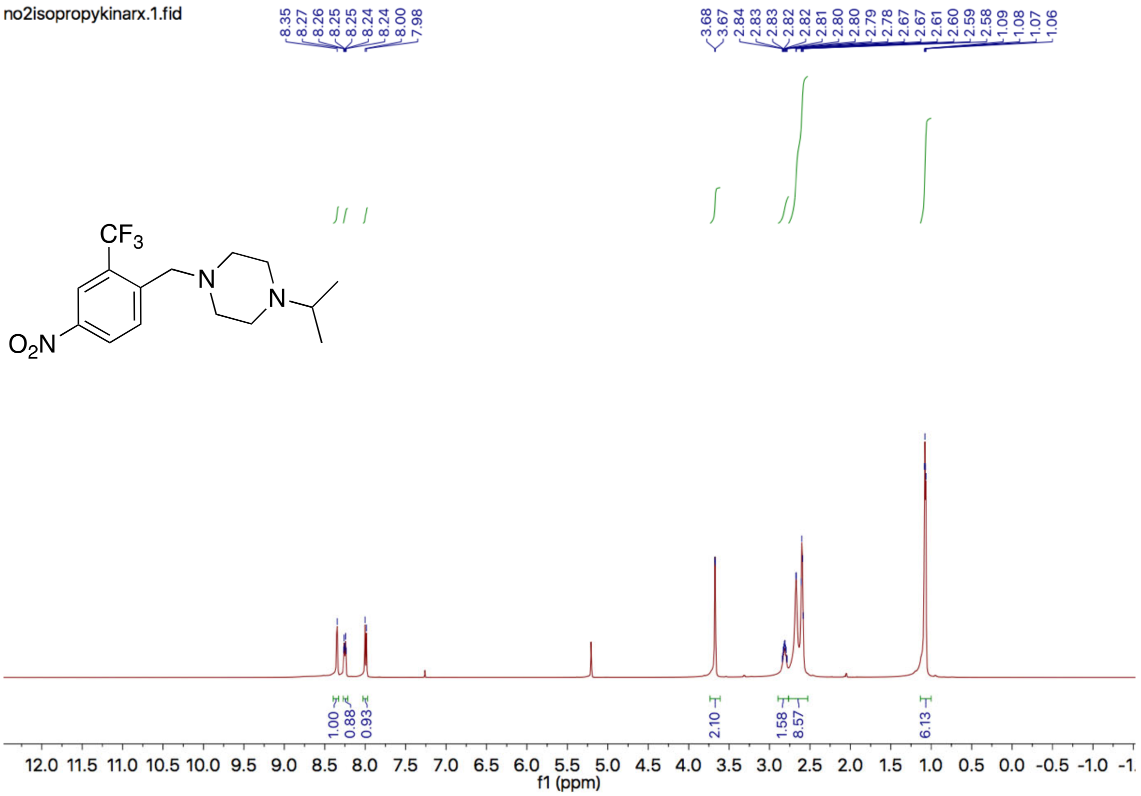
no2isopropykinarx.1.fid
8.35 8.27 8.26 8.25 8.25 8.24 8.24 8.00 7.98
3.68 3.67 2.84 2.83 2.83 2.82 2.82 2.81 2.80 2.80 2.79 2.78 2.67 2.67 2.61 2.60 2.59 2.58 1.09 1.08 1.07 1.06
CF3
N
N
O2N
1.00 0.88 0.93
2.10
1.58 8.57
6.13
12.0 11.5 11.0 10.5 10.0 9.5 9.0 8.5 8.0 7.5 7.0 6.5 6.0 5.5 5.0 4.5 4.0 3.5 3.0 2.5 2.0 1.5 1.0 0.5 0.0 -0.5 -1.0
f1 (ppm)

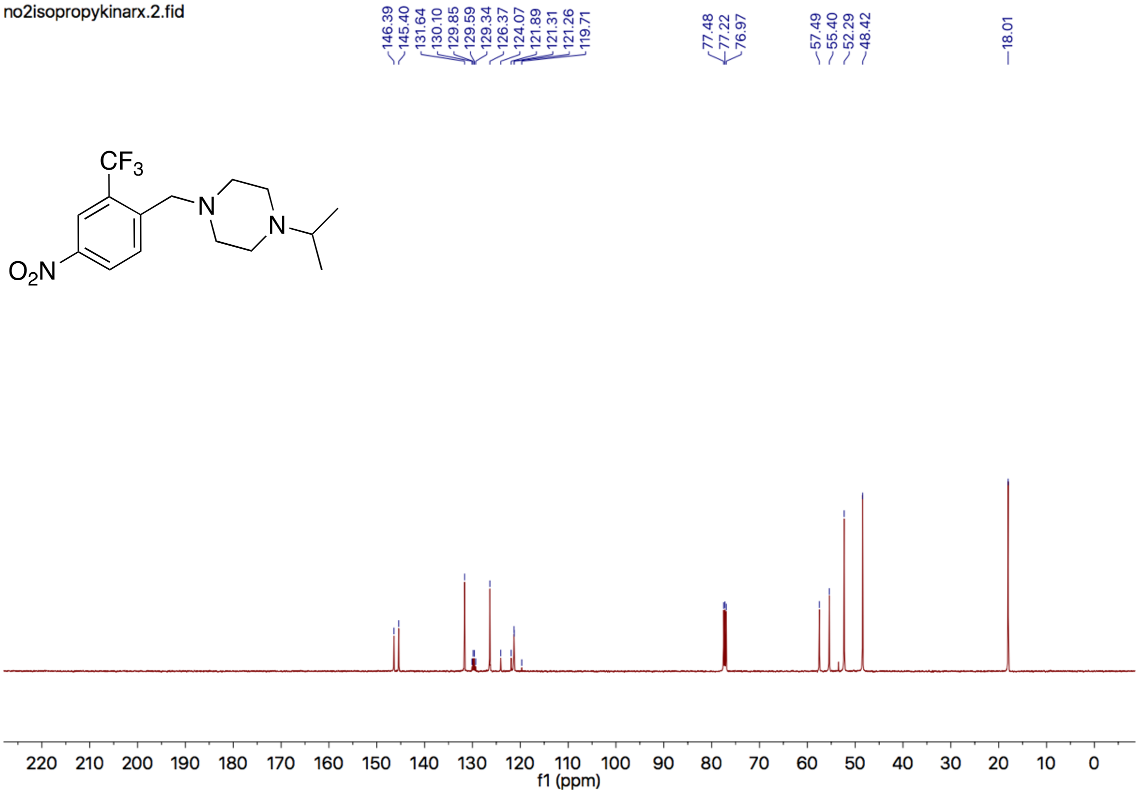
no2isopropykinarx.2.fid
146.39 145.40 131.64 130.10 129.85 129.59 129.34 126.37 124.07 121.89 121.31 121.26 119.71
77.48 77.22 76.97
57.49 55.40 52.29 48.42
18.01
CF3
N
N
O2N
220 210 200 190 180 170 160 150 140 130 120 110 100 90 80 70 60 50 40 30 20 10 0
f1 (ppm)

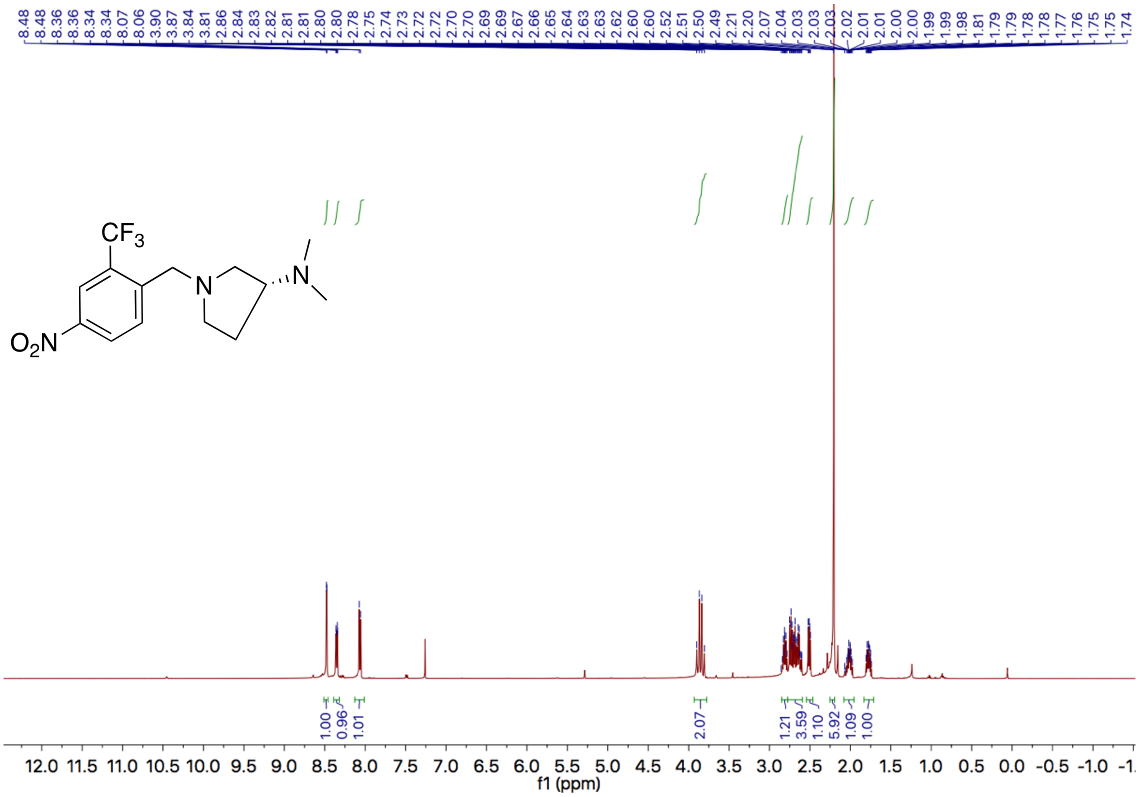
CF3
N
N
O2N
1.00
0.96
1.01
2.07
1.21
3.59
1.10
5.92
1.09
1.00
f1 (ppm)

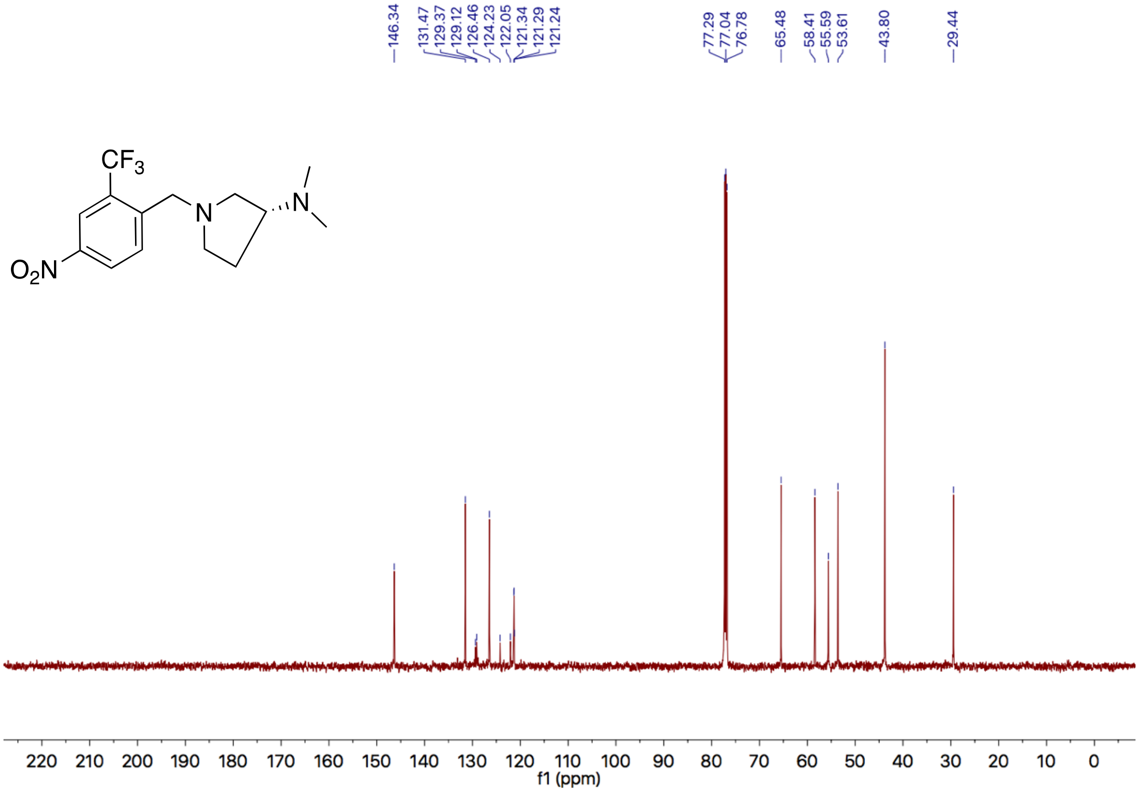
146.34
131.47
129.37
129.12
126.46
124.23
122.05
121.34
121.29
121.24
77.29
77.04
76.78
65.48
58.41
55.59
53.61
43.80
29.44
CF3
N
N
O2N
f1 (ppm)

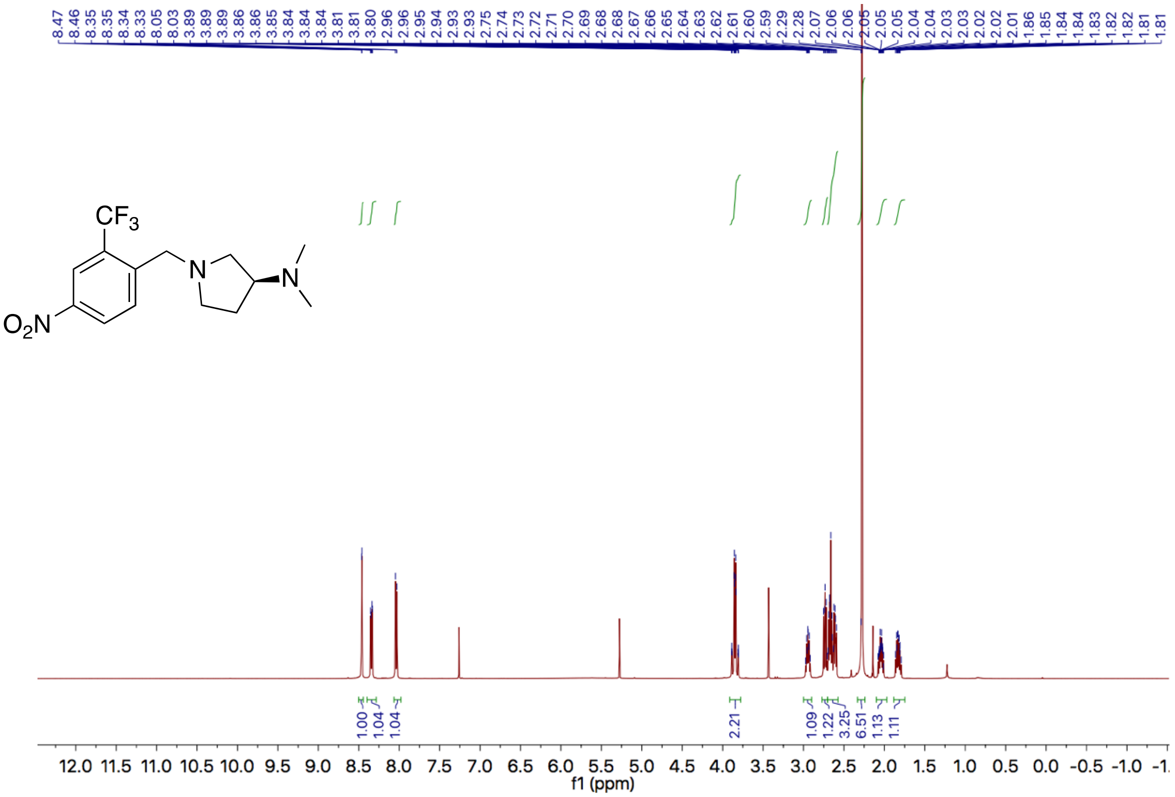
8.47 8.46 8.35 8.35 8.34 8.33 8.05 8.03 3.89 3.89 3.89 3.86 3.86 3.85 3.84 3.84 3.84 3.81 3.81 3.80 2.96 2.96 2.95 2.94 2.93 2.93 2.75 2.74 2.73 2.72 2.71 2.70 2.69 2.68 2.68 2.67 2.66 2.65 2.64 2.63 2.62 2.61 2.60 2.59 2.29 2.28 2.07 2.06 2.06 2.05 2.05 2.05 2.04 2.04 2.03 2.03 2.02 2.02 2.01 1.86 1.85 1.84 1.84 1.83 1.82 1.82 1.81 1.81
CF3
O2N
N
N
1.00 1.04 1.04 2.21 1.09 1.22 3.25 6.51 1.13 1.11
12.0 11.5 11.0 10.5 10.0 9.5 9.0 8.5 8.0 7.5 7.0 6.5 6.0 5.5 5.0 4.5 4.0 3.5 3.0 2.5 2.0 1.5 1.0 0.5 0.0 -0.5 -1.0
f1 (ppm)

S-176

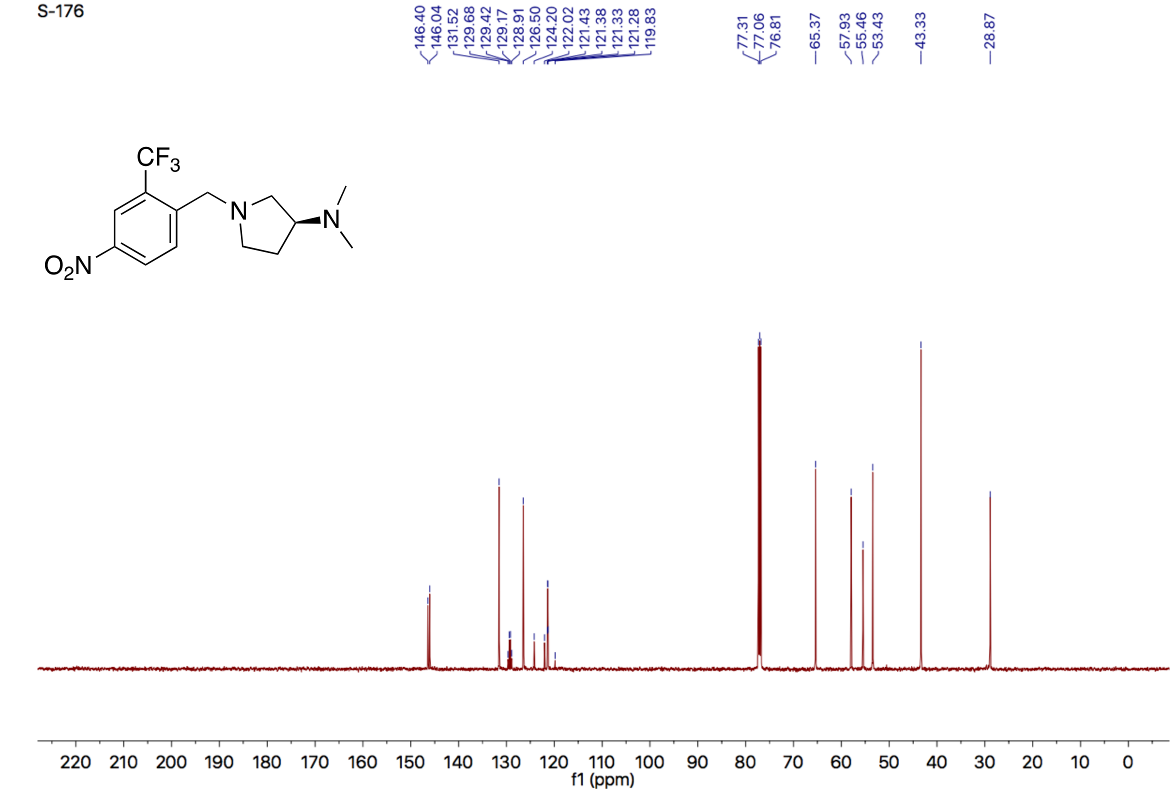
146.40 146.04 131.52 129.68 129.42 129.17 128.91 126.50 124.20 122.02 121.43 121.38 121.33 121.28 119.83
77.31 77.06 76.81
65.37
57.93 55.46 53.43
43.33
28.87
CF3
O2N
N
N
220 210 200 190 180 170 160 150 140 130 120 110 100 90 80 70 60 50 40 30 20 10 0
f1 (ppm)

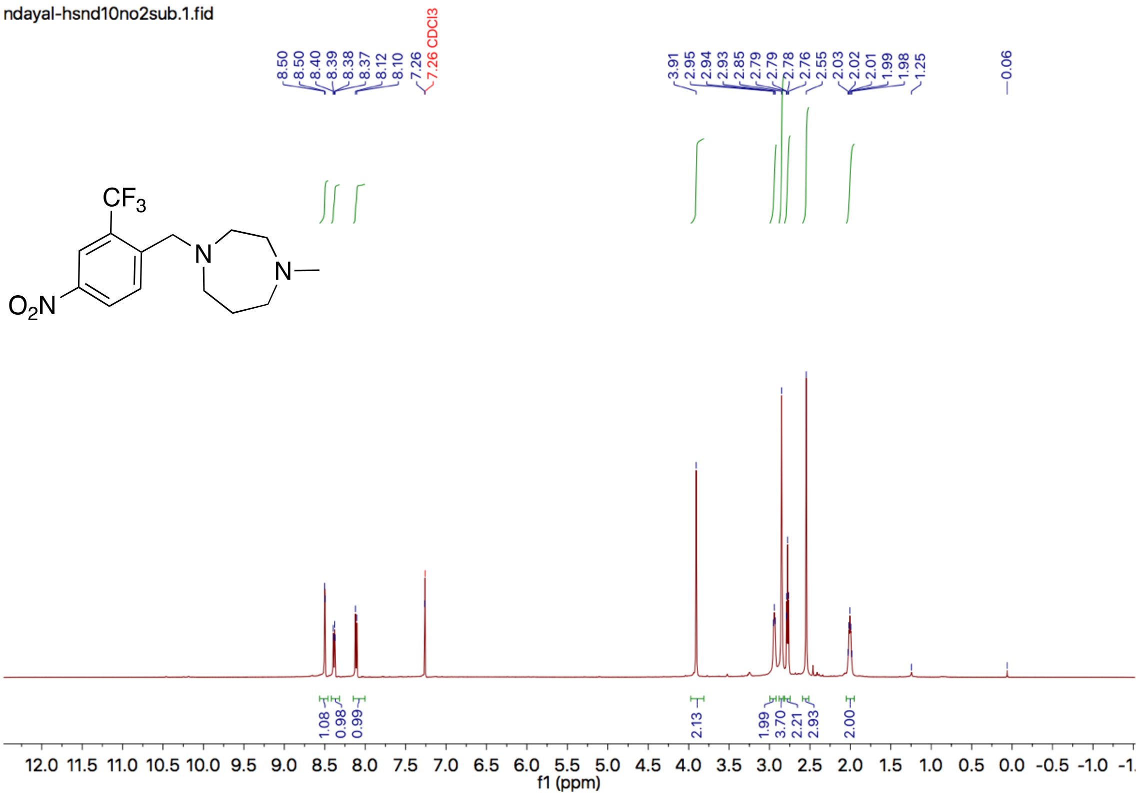
ndayal-hsnd10no2sub.1.fid
8.50
8.50
8.40
8.39
8.38
8.37
8.12
8.10
7.26
7.26 CDCl3
3.91
2.95
2.94
2.93
2.85
2.79
2.79
2.78
2.76
2.55
2.03
2.02
2.01
1.99
1.98
1.25
0.06
CF3
N
N
O2N
1.08
0.98
0.99
2.13
1.99
3.70
2.21
2.93
2.00
12.0 11.5 11.0 10.5 10.0 9.5 9.0 8.5 8.0 7.5 7.0 6.5 6.0 5.5 5.0 4.5 4.0 3.5 3.0 2.5 2.0 1.5 1.0 0.5 0.0 -0.5 -1.0
f1 (ppm)

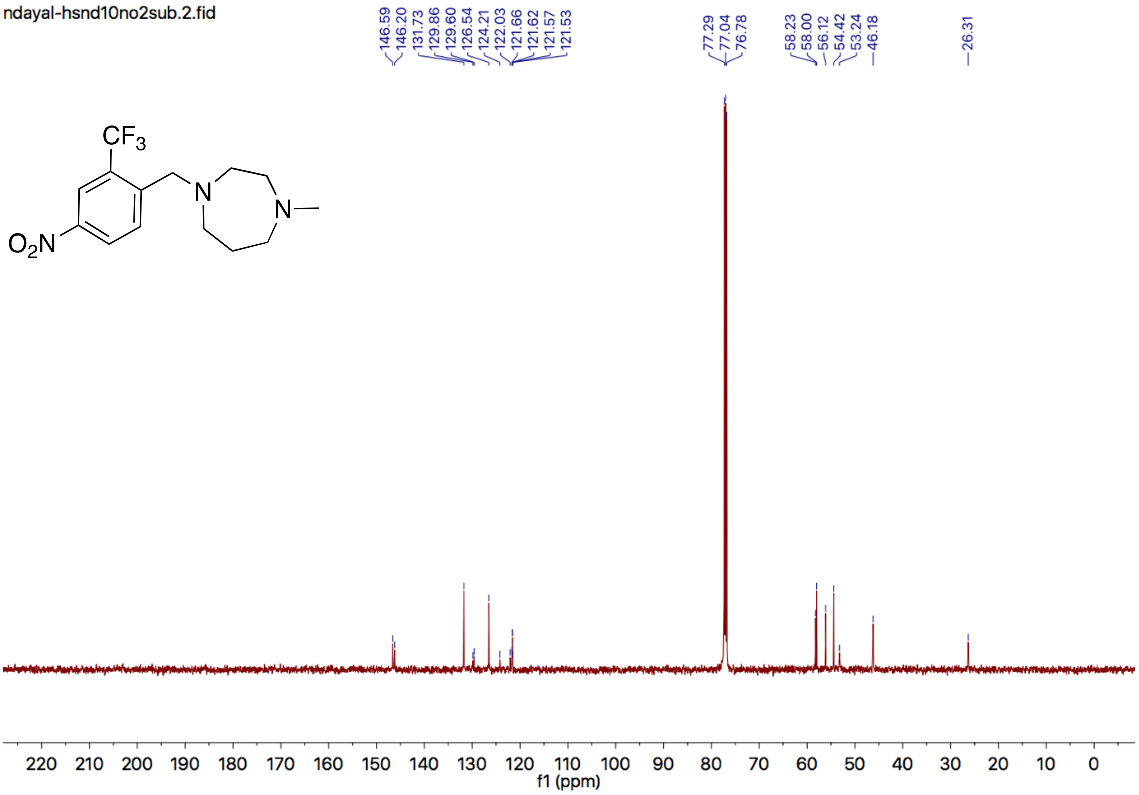
ndayal-hsnd10no2sub.2.fid
146.59
146.20
131.73
129.86
129.60
126.54
124.21
122.03
121.66
121.62
121.57
121.53
77.29
77.04
76.78
58.23
58.00
56.12
54.42
53.24
46.18
26.31
CF3
N
N
O2N
220 210 200 190 180 170 160 150 140 130 120 110 100 90 80 70 60 50 40 30 20 10 0
f1 (ppm)

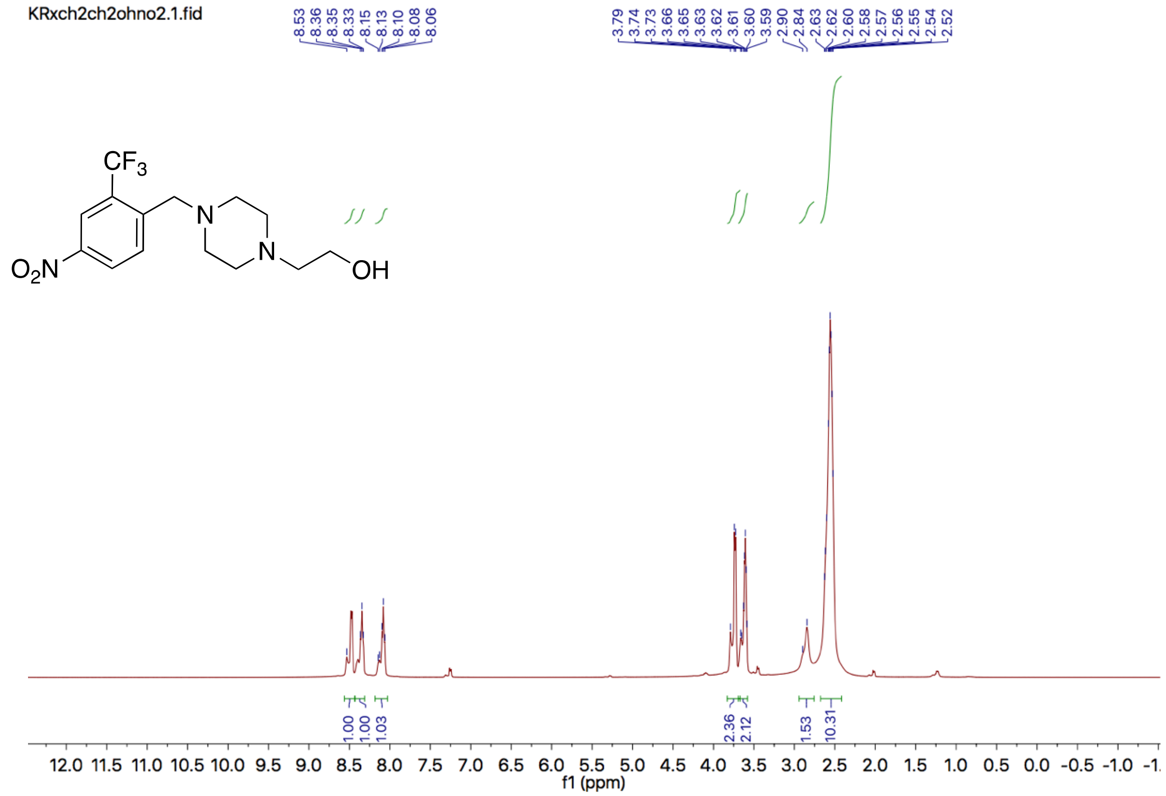
KRxch2ch2ohno2.1.fid
8.53 8.36 8.35 8.33 8.15 8.13 8.10 8.08 8.06
3.79 3.74 3.73 3.66 3.65 3.63 3.62 3.61 3.60 3.59 2.90 2.84 2.63 2.62 2.60 2.58 2.57 2.56 2.55 2.54 2.52
CF3
N
N
OH
O2N
1.00 1.00 1.03 2.36 2.12 1.53 10.31
12.0 11.5 11.0 10.5 10.0 9.5 9.0 8.5 8.0 7.5 7.0 6.5 6.0 5.5 5.0 4.5 4.0 3.5 3.0 2.5 2.0 1.5 1.0 0.5 0.0 -0.5 -1.0
f1 (ppm)

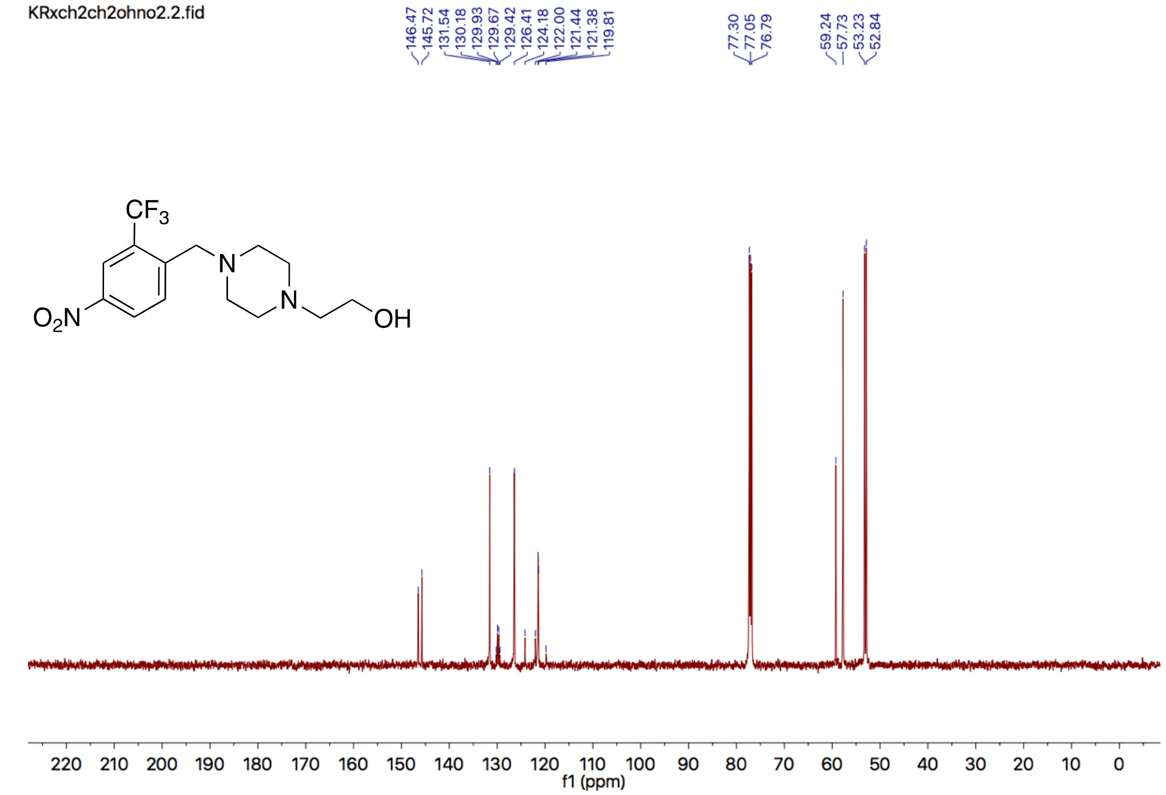
KRxch2ch2ohno2.2.fid
146.47 145.72 131.54 130.18 129.93 129.67 129.42 126.41 124.18 122.00 121.44 121.38 119.81
77.30 77.05 76.79
59.24 57.73 53.23 52.84
CF3
N
N
OH
O2N
220 210 200 190 180 170 160 150 140 130 120 110 100 90 80 70 60 50 40 30 20 10 0
f1 (ppm)

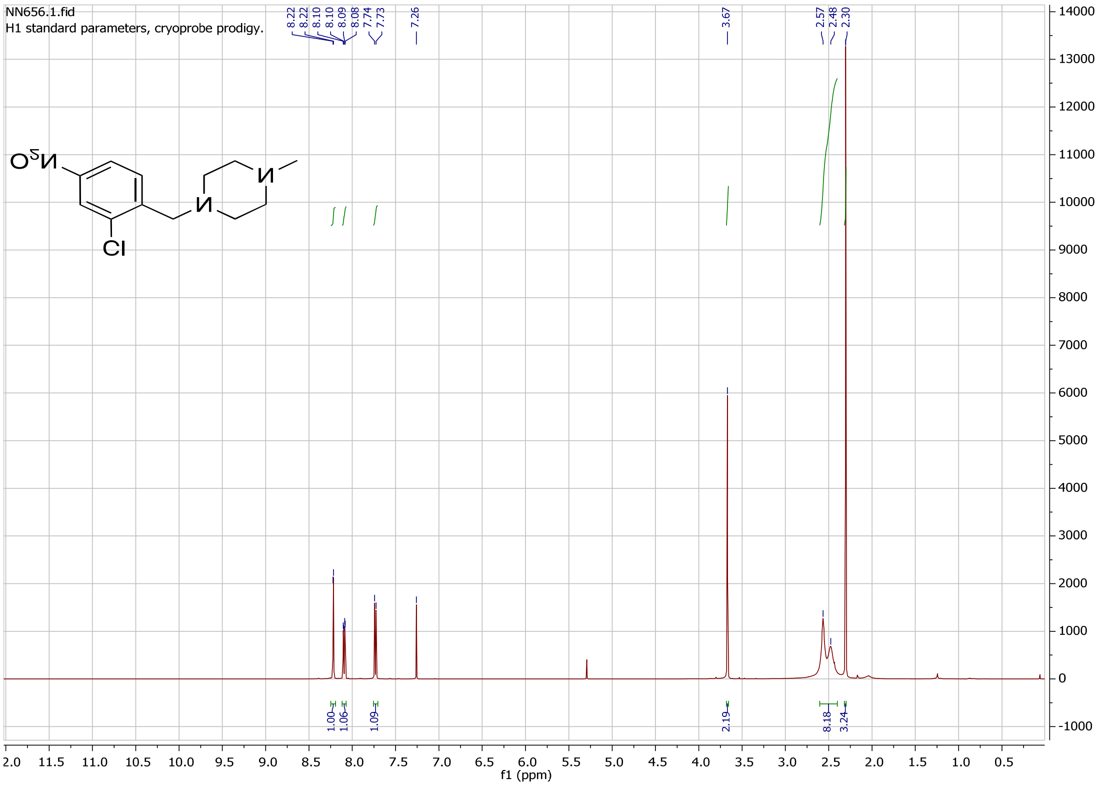
NN656.1.fid
H1 standard parameters, cryoprobe prodigy.
8.22
8.22
8.10
8.10
8.09
8.08
7.74
7.73
7.26
3.67
2.57
2.48
2.30
1.00
1.06
1.09
2.19
8.18
3.24
Cl
f1 (ppm)

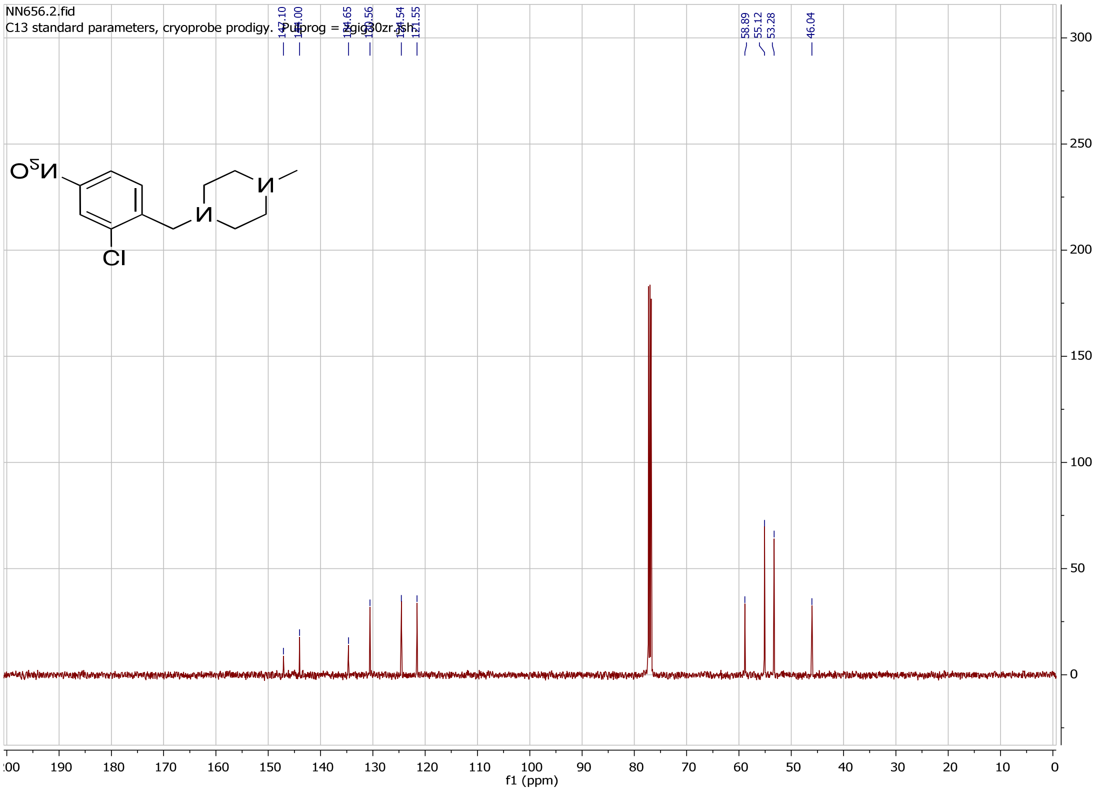
NN656.2.fid
C13 standard parameters, cryoprobe prodigy. Pulprog = zgig30zr.jsh
58.89
55.12
53.28
46.04
Cl
f1 (ppm)

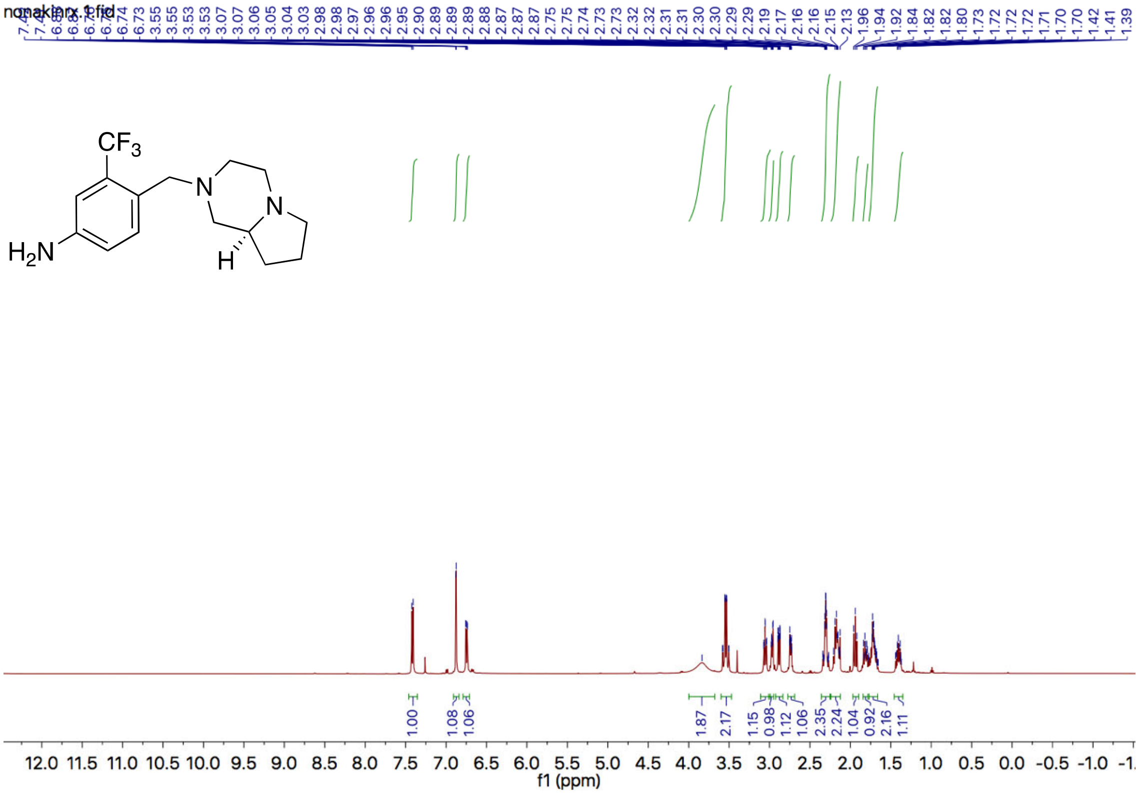
nonakinrx.1.fid
6.74
6.73
3.55
3.55
3.53
3.53
3.07
3.07
3.06
3.05
3.04
3.03
2.98
2.98
2.97
2.96
2.96
2.95
2.90
2.89
2.89
2.89
2.88
2.87
2.87
2.87
2.75
2.75
2.74
2.73
2.73
2.32
2.32
2.31
2.31
2.30
2.30
2.29
2.29
2.19
2.17
2.16
2.16
2.15
2.13
1.96
1.94
1.92
1.84
1.82
1.82
1.80
1.73
1.72
1.72
1.72
1.71
1.71
1.70
1.42
1.41
1.39
CF3
N
N
H2N
H
1.00
1.08
1.06
1.87
2.17
1.15
0.98
1.12
1.06
2.35
2.24
1.04
0.92
2.16
1.11
12.0 11.5 11.0 10.5 10.0 9.5 9.0 8.5 8.0 7.5 7.0 6.5 6.0 5.5 5.0 4.5 4.0 3.5 3.0 2.5 2.0 1.5 1.0 0.5 0.0 -0.5 -1.0
f1 (ppm)

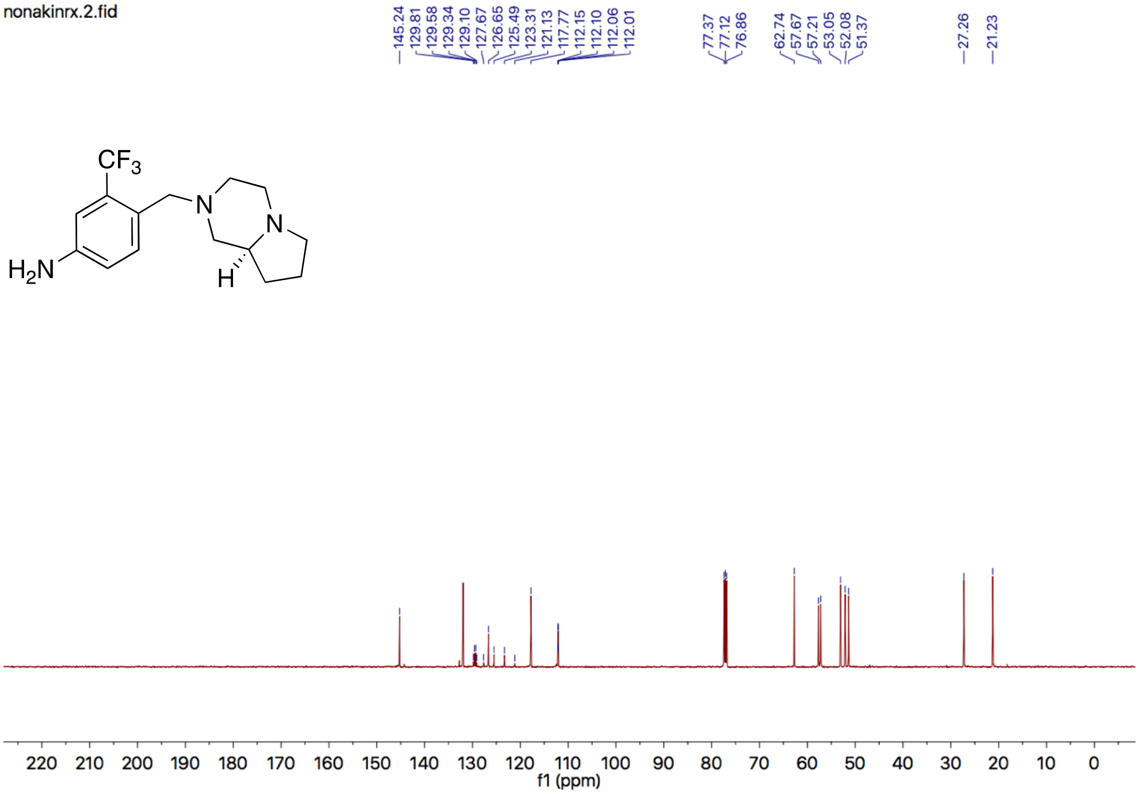
nonakinrx.2.fid
145.24
129.81
129.58
129.34
129.10
127.67
126.65
125.49
123.31
121.13
117.77
112.15
112.10
112.06
112.01
77.37
77.12
76.86
62.74
57.67
57.21
53.05
52.08
51.37
27.26
21.23
CF3
N
N
H2N
H
220 210 200 190 180 170 160 150 140 130 120 110 100 90 80 70 60 50 40 30 20 10 0
f1 (ppm)

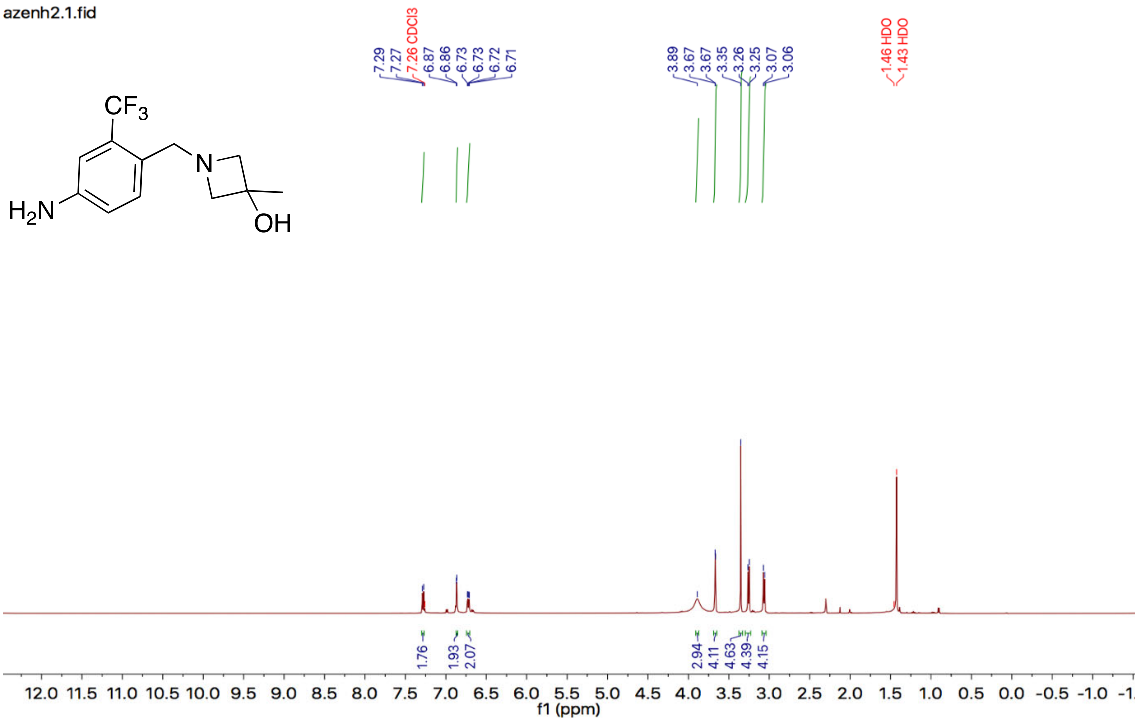
azenh2.1.fid
7.29
7.27
7.26 CDCl3
6.87
6.86
6.73
6.73
6.72
6.71
3.89
3.67
3.67
3.35
3.26
3.25
3.07
3.06
1.46 HDO
1.43 HDO
CF3
H2N
N
OH
1.76
1.93
2.07
2.94
4.11
4.63
4.39
4.15
12.0 11.5 11.0 10.5 10.0 9.5 9.0 8.5 8.0 7.5 7.0 6.5 6.0 5.5 5.0 4.5 4.0 3.5 3.0 2.5 2.0 1.5 1.0 0.5 0.0 -0.5 -1.0
f1 (ppm)

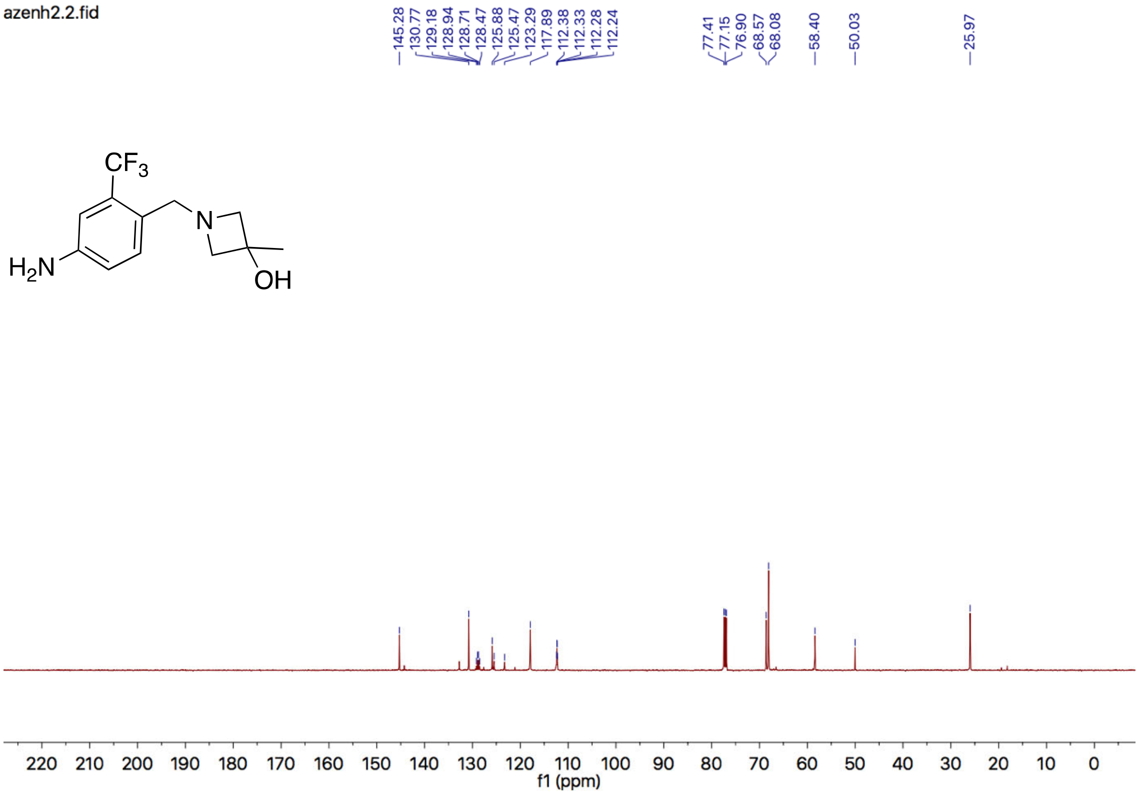
azenh2.2.fid
145.28
130.77
129.18
128.94
128.71
128.47
125.88
125.47
123.29
117.89
112.38
112.33
112.28
112.24
77.41
77.15
76.90
68.57
68.08
58.40
50.03
25.97
CF3
H2N
N
OH
220 210 200 190 180 170 160 150 140 130 120 110 100 90 80 70 60 50 40 30 20 10 0
f1 (ppm)

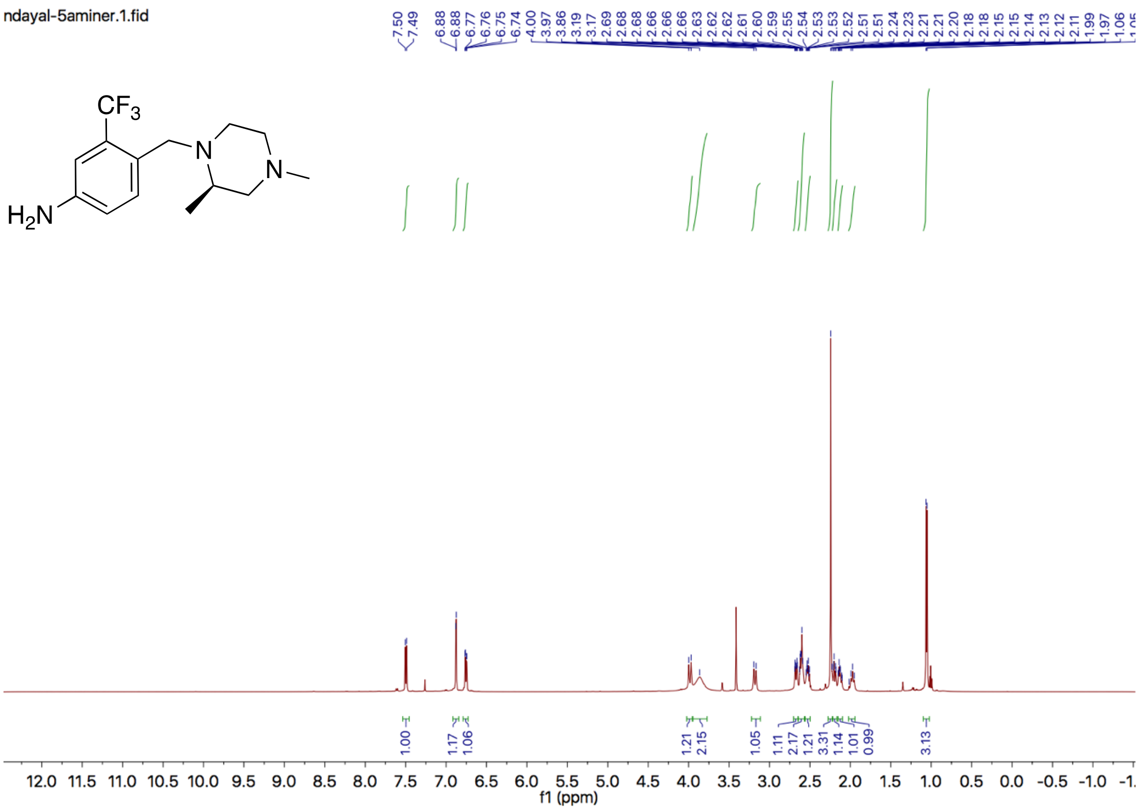
ndayal-5aminer.1.fid
CF3
N
N
H2N
7.50
7.49
6.88
6.88
6.77
6.76
6.75
6.74
4.00
3.97
3.86
3.19
3.17
2.69
2.68
2.68
2.66
2.66
2.66
2.63
2.62
2.62
2.61
2.60
2.59
2.55
2.54
2.53
2.53
2.52
2.51
2.51
2.24
2.23
2.21
2.21
2.20
2.18
2.15
2.15
2.14
2.13
2.12
2.11
1.99
1.97
1.06
1.00
1.17
1.06
1.21
2.15
1.05
1.11
2.17
1.21
3.31
1.14
1.01
0.99
3.13
12.0 11.5 11.0 10.5 10.0 9.5 9.0 8.5 8.0 7.5 7.0 6.5 6.0 5.5 5.0 4.5 4.0 3.5 3.0 2.5 2.0 1.5 1.0 0.5 0.0 -0.5 -1.0
f1 (ppm)

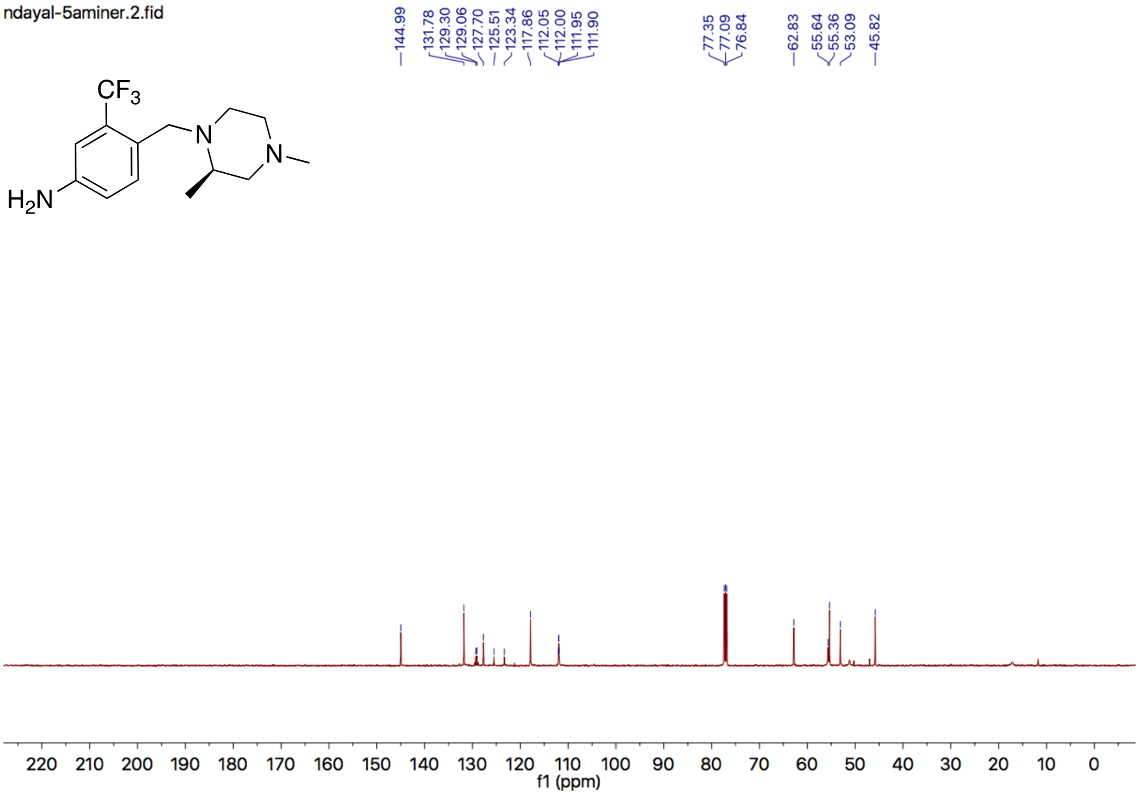
ndayal-5aminer.2.fid
CF3
N
N
H2N
144.99
131.78
129.30
129.06
127.70
125.51
123.34
117.86
112.05
112.00
111.95
111.90
77.35
77.09
76.84
62.83
55.64
55.36
53.09
45.82
220 210 200 190 180 170 160 150 140 130 120 110 100 90 80 70 60 50 40 30 20 10 0
f1 (ppm)

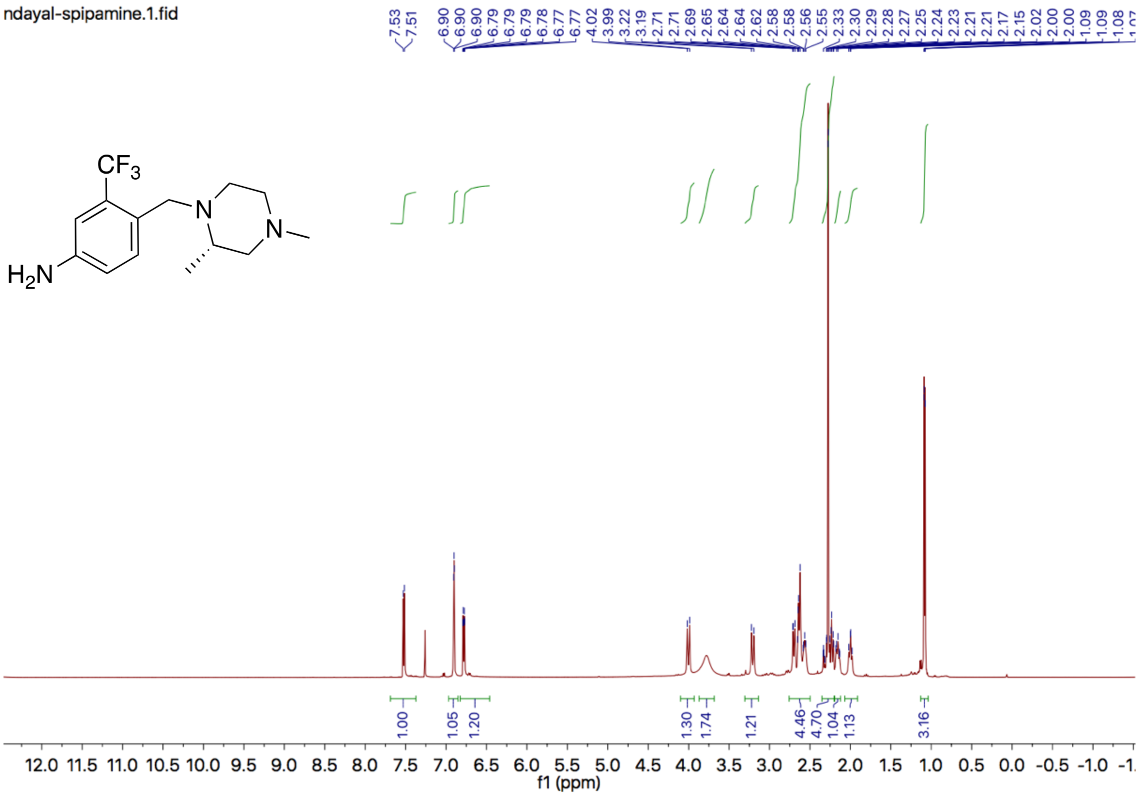
ndayal-spipamine.1.fid
7.53
7.51
6.90
6.90
6.90
6.79
6.79
6.79
6.78
6.77
6.77
4.02
3.99
3.22
3.19
2.71
2.71
2.69
2.65
2.64
2.64
2.62
2.58
2.58
2.56
2.55
2.33
2.30
2.29
2.28
2.27
2.25
2.24
2.23
2.21
2.21
2.17
2.15
2.02
2.00
2.00
1.09
1.09
1.08
CF3
N
N
H2N
1.00
1.05
1.20
1.30
1.74
1.21
4.46
4.70
1.04
1.13
3.16
12.0 11.5 11.0 10.5 10.0 9.5 9.0 8.5 8.0 7.5 7.0 6.5 6.0 5.5 5.0 4.5 4.0 3.5 3.0 2.5 2.0 1.5 1.0 0.5 0.0 -0.5 -1.0
f1 (ppm)

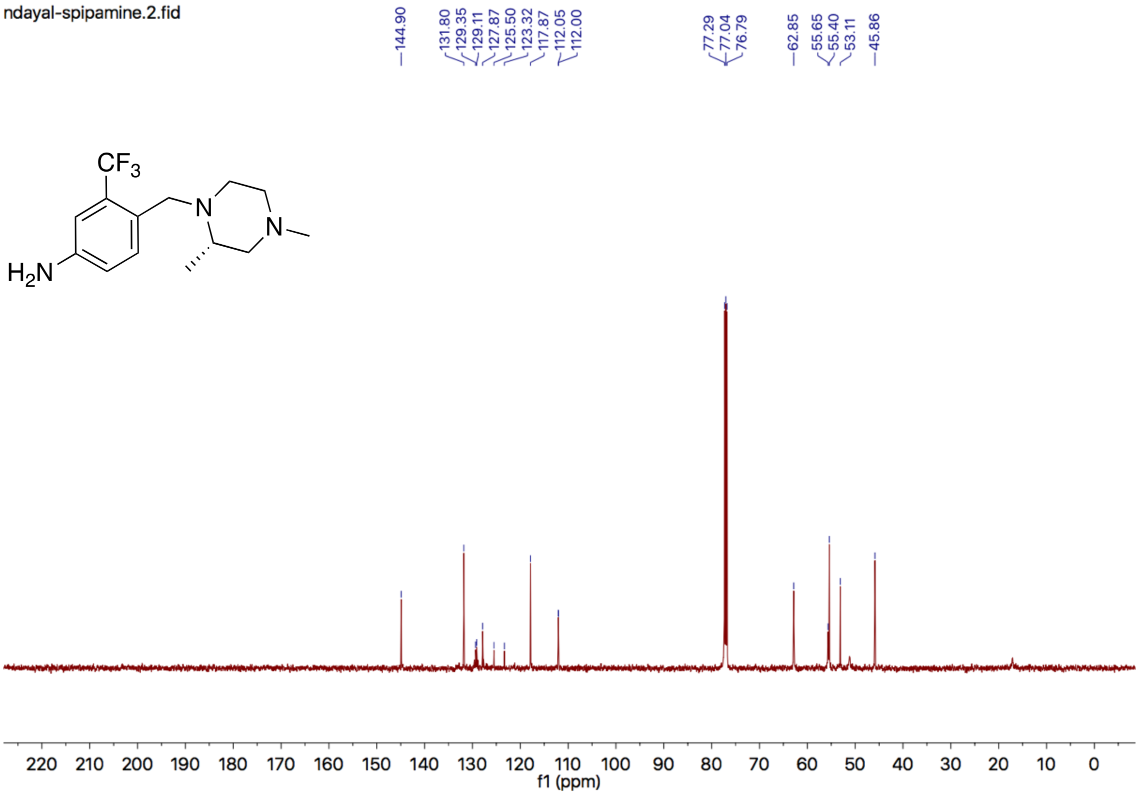
ndayal-spipamine.2.fid
144.90
131.80
129.35
129.11
127.87
125.50
123.32
117.87
112.05
112.00
77.29
77.04
76.79
62.85
55.65
55.40
53.11
45.86
CF3
N
N
H2N
220 210 200 190 180 170 160 150 140 130 120 110 100 90 80 70 60 50 40 30 20 10 0
f1 (ppm)

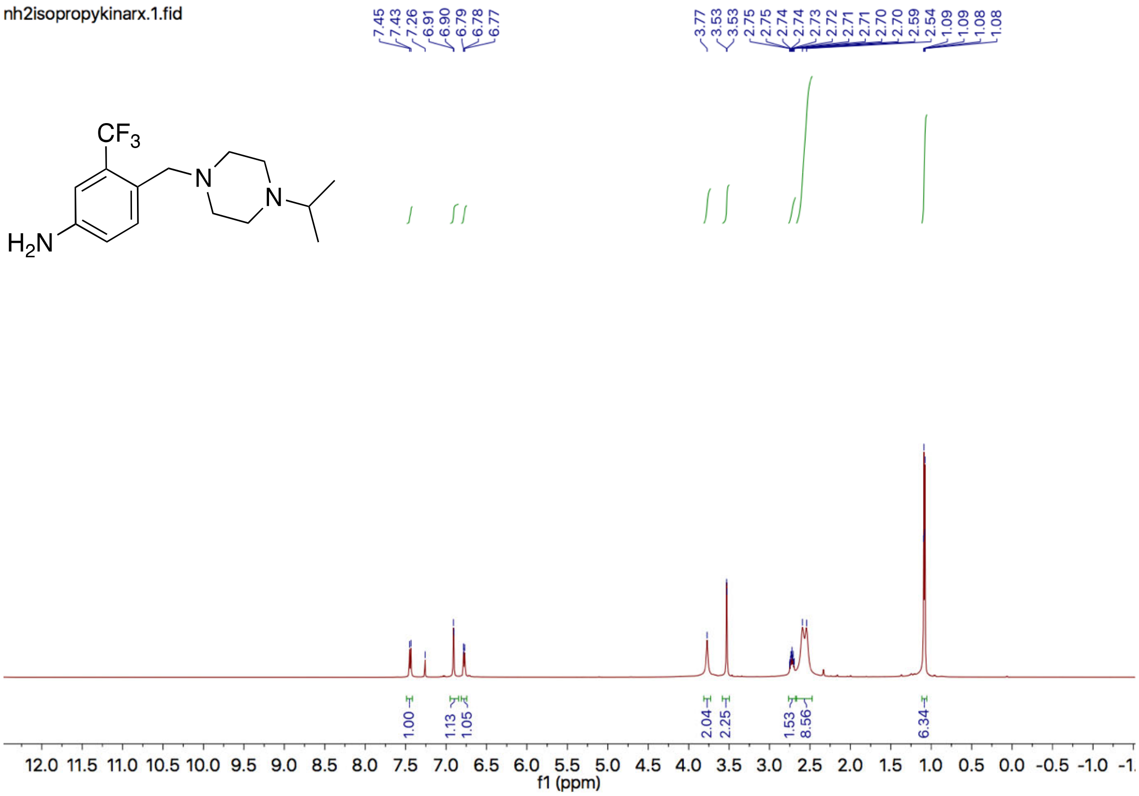
nh2isopropykinarx.1.fid
7.45
7.43
7.26
6.91
6.90
6.79
6.78
6.77
3.77
3.53
3.53
2.75
2.75
2.74
2.74
2.73
2.72
2.71
2.71
2.70
2.70
2.59
2.54
1.09
1.09
1.08
1.08
CF_3
N
N
H_2N
1.00
1.13
1.05
2.04
2.25
1.53
8.56
6.34
f1 (ppm)

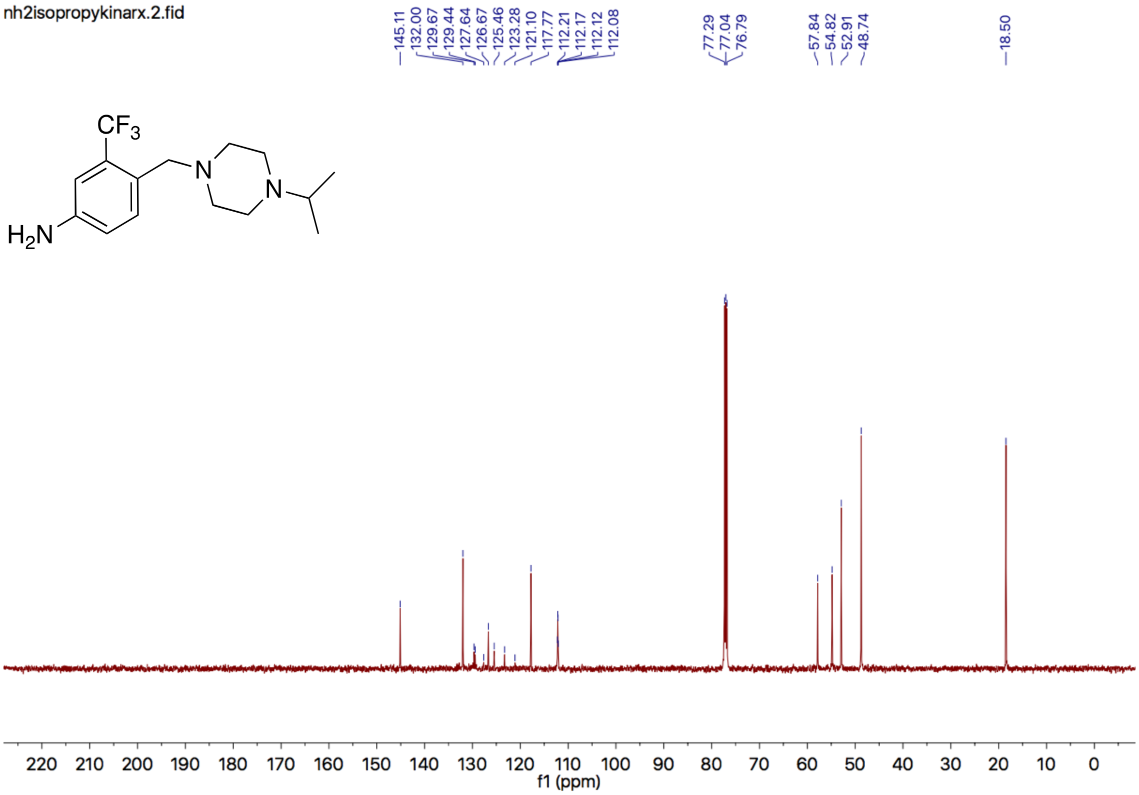
nh2isopropykinarx.2.fid
145.11
132.00
129.67
129.44
127.64
126.67
125.46
123.28
121.10
117.77
112.21
112.17
112.12
112.08
77.29
77.04
76.79
57.84
54.82
52.91
48.74
18.50
CF_3
N
N
H_2N
f1 (ppm)

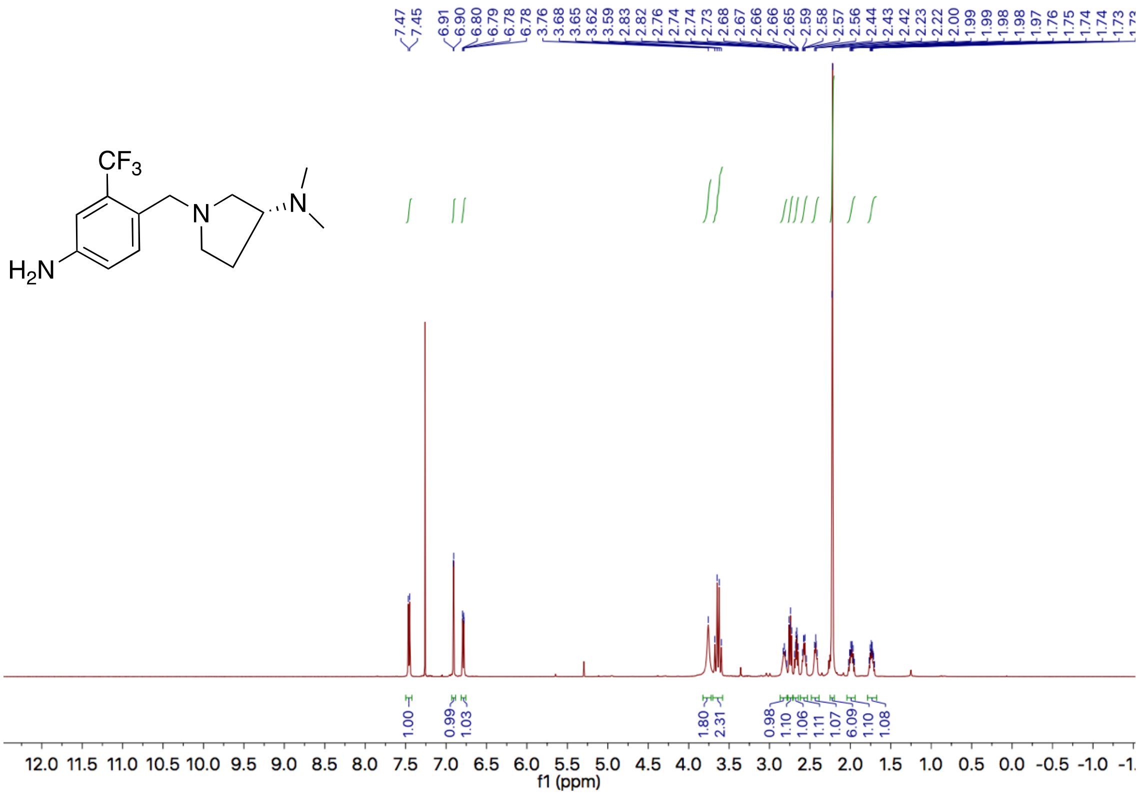
7.47
7.45
6.91
6.90
6.80
6.79
6.78
6.78
3.76
3.68
3.65
3.62
3.59
2.83
2.82
2.76
2.74
2.74
2.73
2.68
2.67
2.66
2.66
2.65
2.59
2.58
2.57
2.56
2.44
2.43
2.42
2.23
2.22
2.00
1.99
1.98
1.98
1.97
1.76
1.75
1.74
1.73
CF_3
N
N
H_2N
1.00
0.99
1.03
1.80
2.31
0.98
1.10
1.06
1.11
1.07
6.09
1.10
1.08
f1 (ppm)

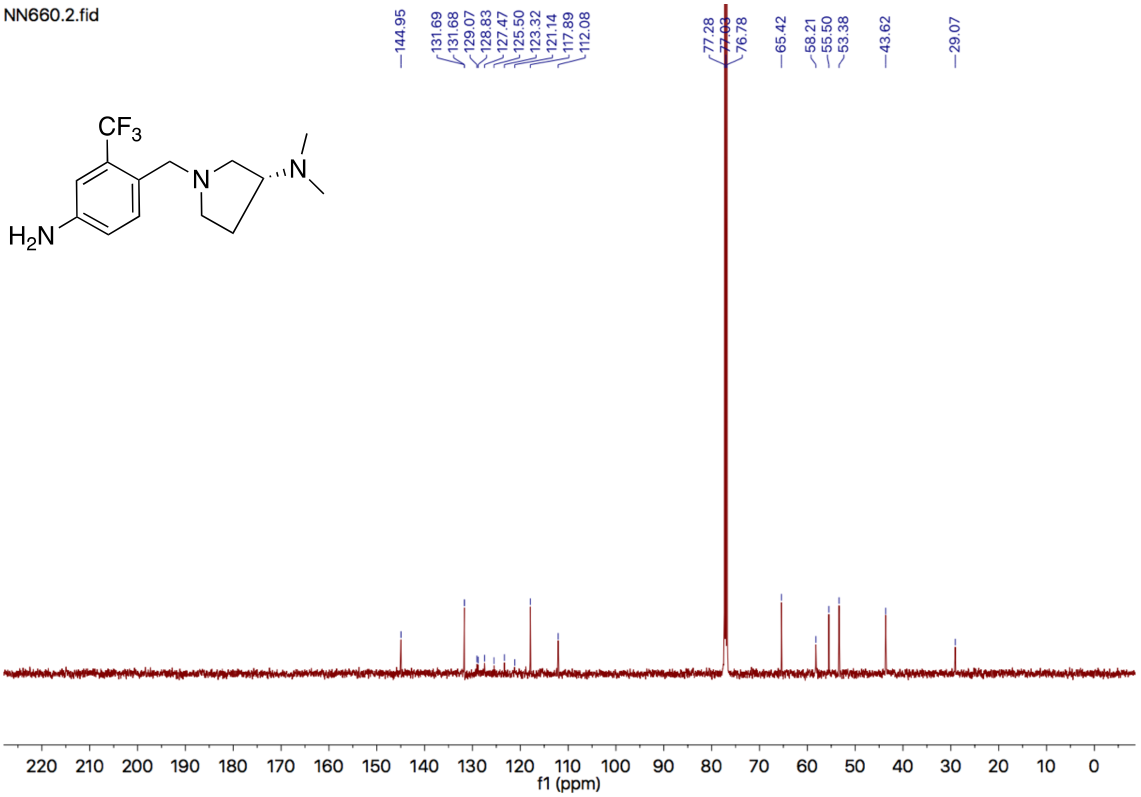
NN660.2.fid
144.95
131.69
131.68
129.07
128.83
127.47
125.50
123.32
121.14
117.89
112.08
77.28
77.03
76.78
65.42
58.21
55.50
53.38
43.62
29.07
CF_3
N
N
H_2N
f1 (ppm)

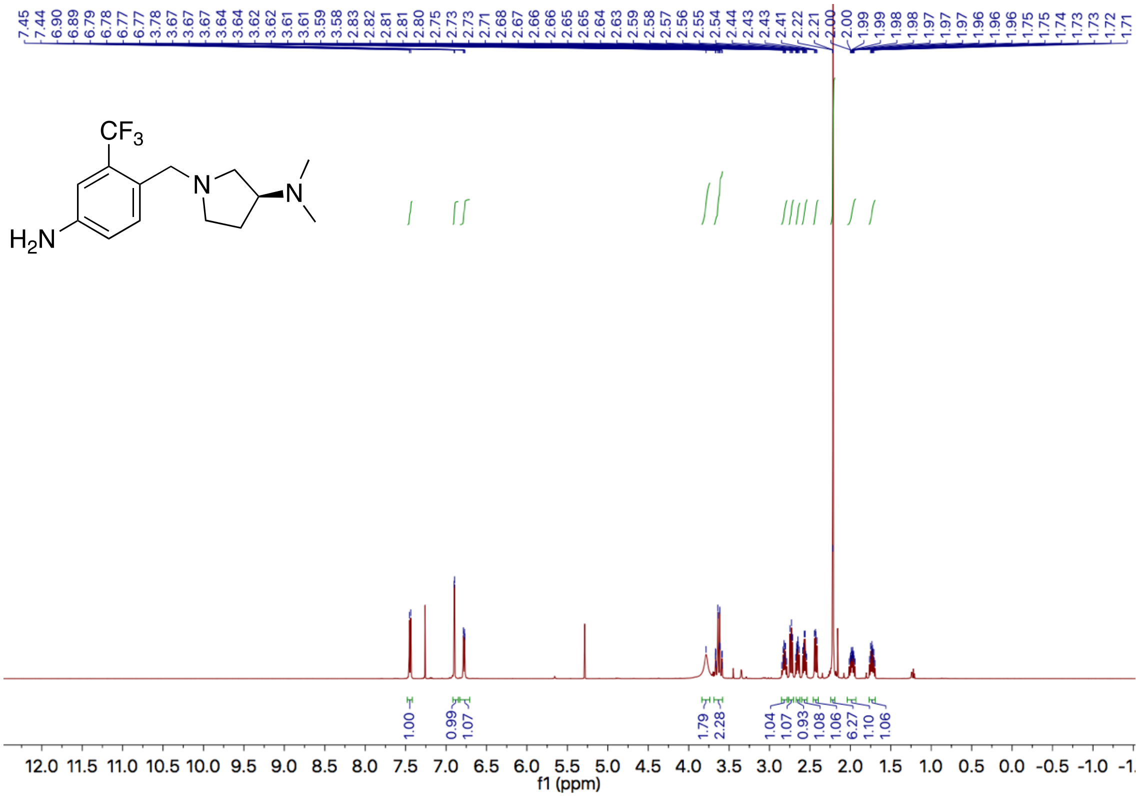
CF3
N
N
H2N
7.45 7.44 6.90 6.89 6.79 6.78 6.77 6.77 3.78 3.67 3.67 3.67 3.64 3.64 3.62 3.62 3.61 3.61 3.59 3.58 2.83 2.82 2.82 2.81 2.81 2.80 2.75 2.73 2.73 2.71 2.68 2.67 2.66 2.65 2.65 2.64 2.63 2.59 2.58 2.57 2.56 2.55 2.54 2.44 2.43 2.43 2.41 2.22 2.21 2.00 2.00 1.99 1.99 1.98 1.98 1.97 1.97 1.97 1.96 1.96 1.96 1.75 1.75 1.74 1.73 1.73 1.72 1.71
1.00 0.99 1.07 1.79 2.28 1.04 1.07 0.93 1.08 1.06 6.27 1.10 1.06
12.0 11.5 11.0 10.5 10.0 9.5 9.0 8.5 8.0 7.5 7.0 6.5 6.0 5.5 5.0 4.5 4.0 3.5 3.0 2.5 2.0 1.5 1.0 0.5 0.0 -0.5 -1.0
f1 (ppm)

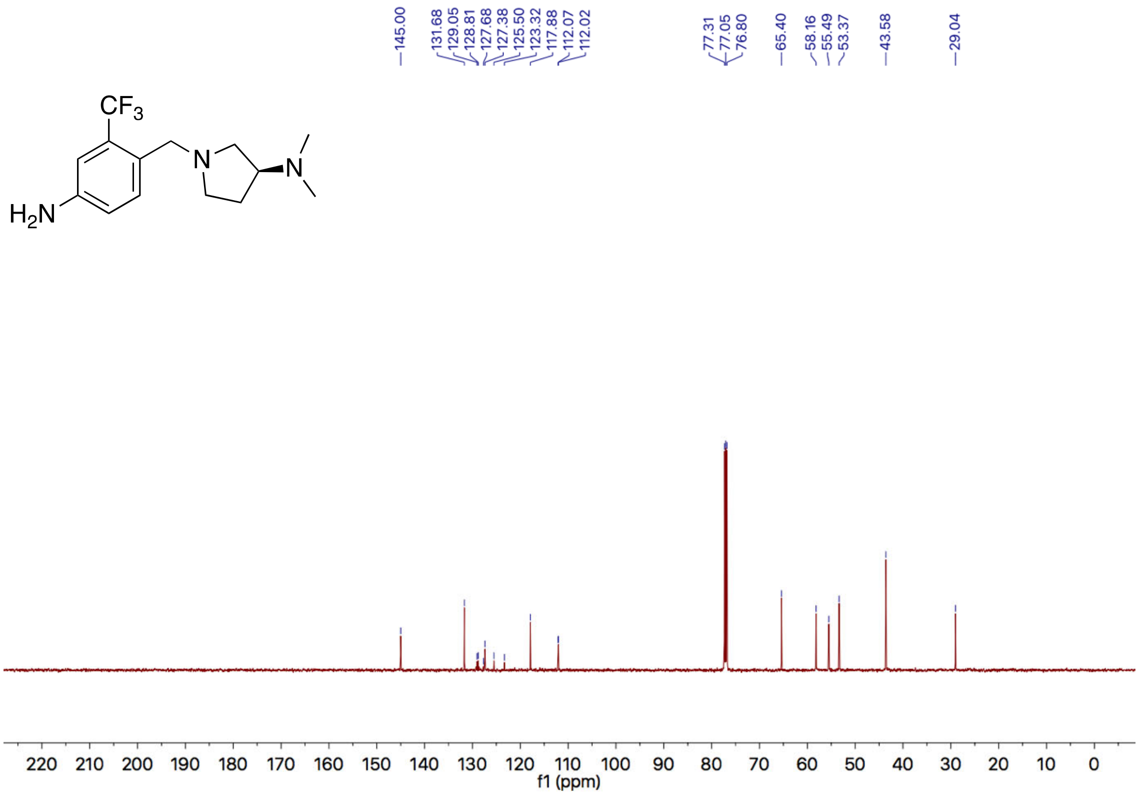
CF3
N
N
H2N
145.00 131.68 129.05 128.81 127.68 127.38 125.50 123.32 117.88 112.07 112.02 77.31 77.05 76.80 65.40 58.16 55.49 53.37 43.58 29.04
220 210 200 190 180 170 160 150 140 130 120 110 100 90 80 70 60 50 40 30 20 10 0
f1 (ppm)

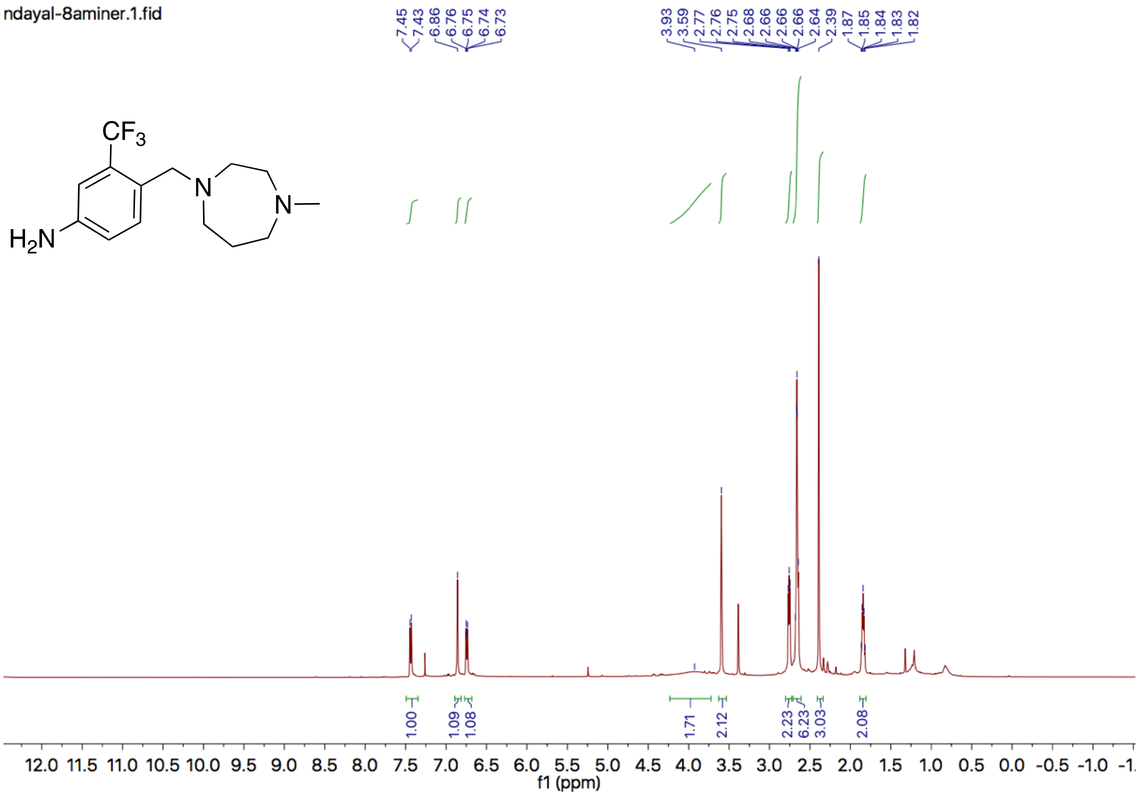
ndayal-8aminer.1.fid
7.45
7.43
6.86
6.76
6.75
6.74
6.73
3.93
3.59
2.77
2.76
2.75
2.68
2.66
2.66
2.66
2.64
2.39
1.87
1.85
1.84
1.83
1.82
CF3
N
N
H2N
1.00
1.09
1.08
1.71
2.12
2.23
6.23
3.03
2.08
f1 (ppm)

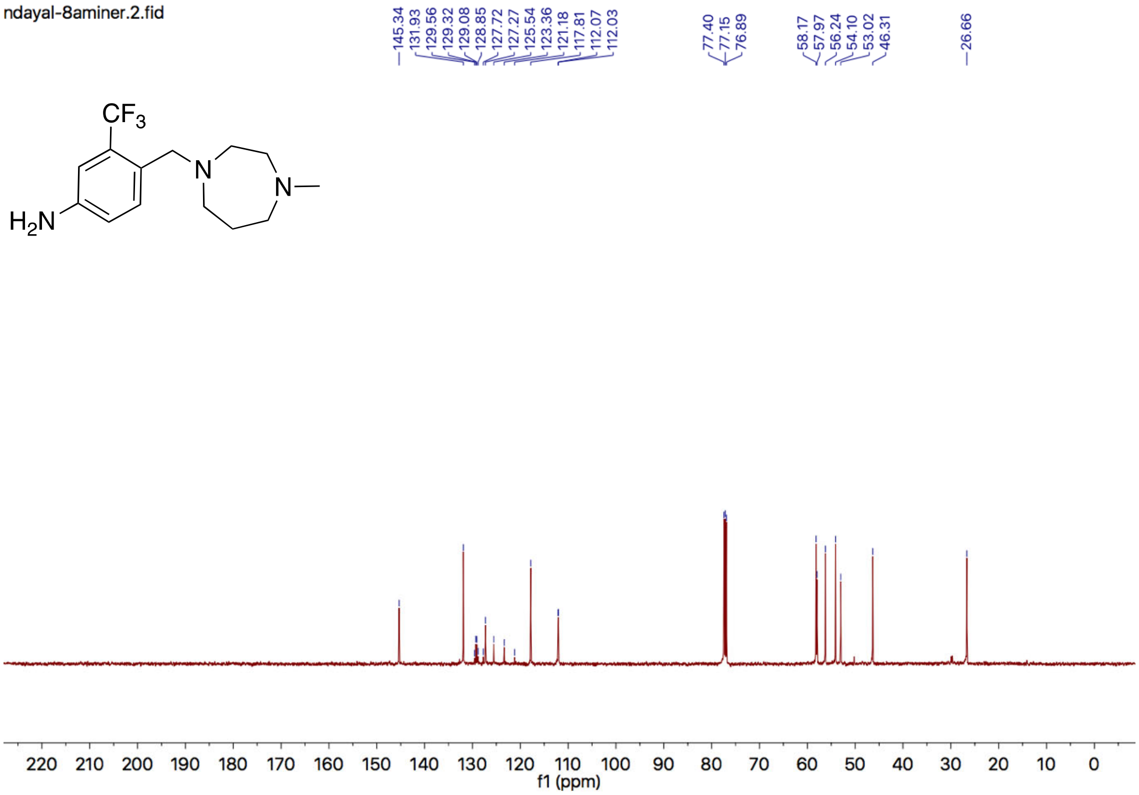
ndayal-8aminer.2.fid
145.34
131.93
129.56
129.32
129.08
128.85
127.72
127.27
125.54
123.36
121.18
117.81
112.07
112.03
77.40
77.15
76.89
58.17
57.97
56.24
54.10
53.02
46.31
26.66
CF3
N
N
H2N
f1 (ppm)

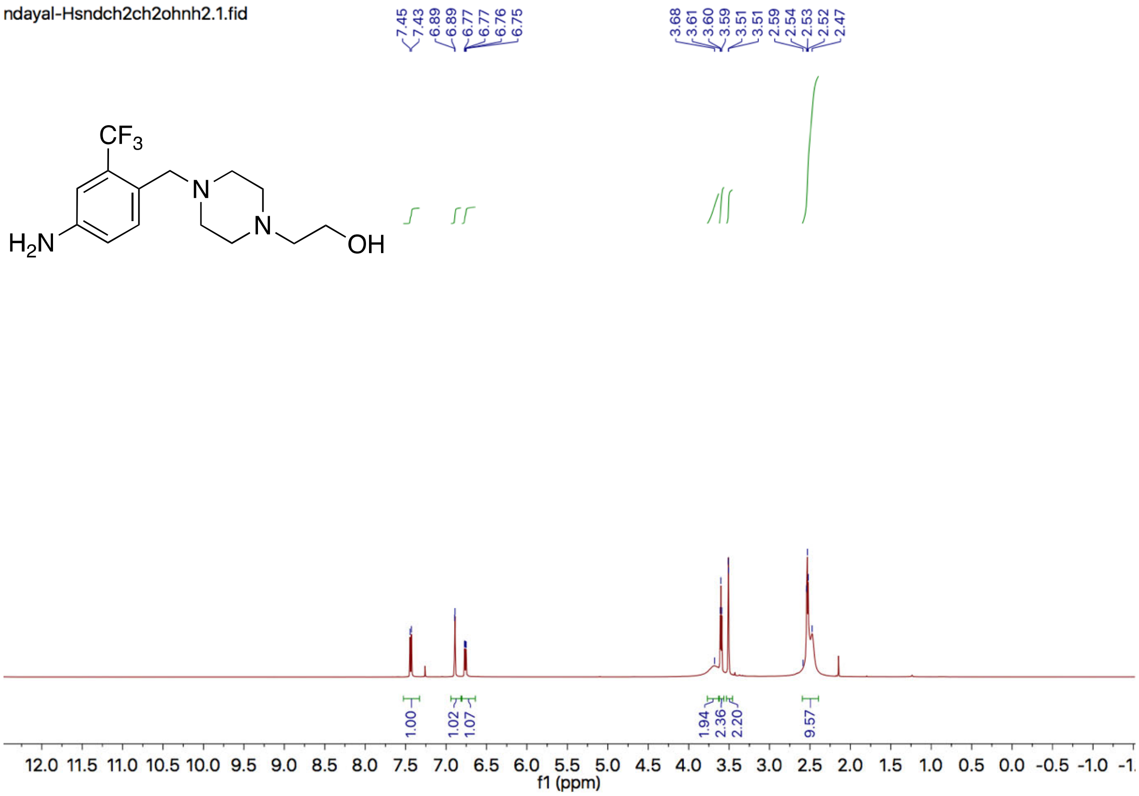
ndayal-Hsndch2ch2ohnh2.1.fid
7.45
7.43
6.89
6.89
6.77
6.77
6.76
6.75
3.68
3.61
3.60
3.59
3.51
3.51
2.59
2.54
2.53
2.52
2.47
1.00
1.02
1.07
1.94
2.20
9.57
f1 (ppm)

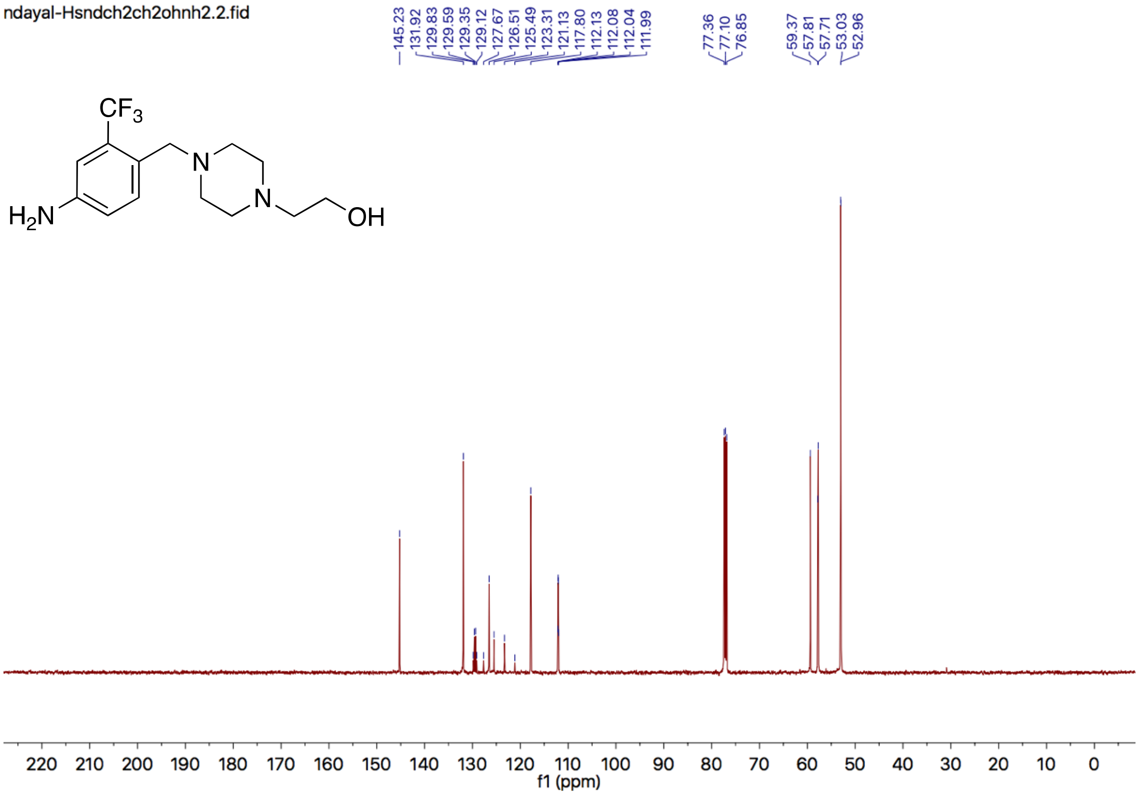
ndayal-Hsndch2ch2ohnh2.2.fid
145.23
131.92
129.83
129.59
129.35
129.12
127.67
126.51
125.49
123.31
121.13
117.80
112.13
112.08
112.04
111.99
77.36
77.10
76.85
59.37
57.81
57.71
53.03
52.96
f1 (ppm)

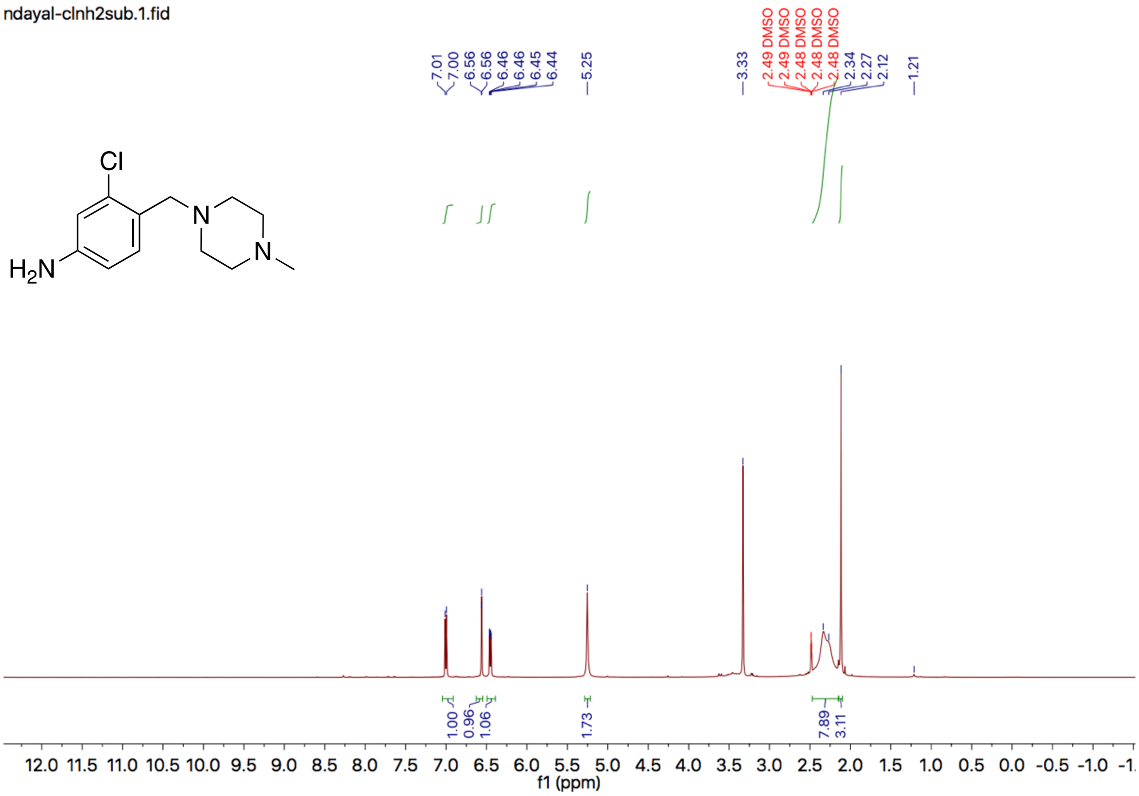
ndayal-clnh2sub.1.fid
7.01
7.00
6.56
6.56
6.46
6.46
6.45
6.44
5.25
3.33
2.49 DMSO
2.49 DMSO
2.48 DMSO
2.48 DMSO
2.48 DMSO
2.34
2.27
2.12
1.21
Cl
N
N
H2N
1.00
0.96
1.06
1.73
7.89
3.11
12.0 11.5 11.0 10.5 10.0 9.5 9.0 8.5 8.0 7.5 7.0 6.5 6.0 5.5 5.0 4.5 4.0 3.5 3.0 2.5 2.0 1.5 1.0 0.5 0.0 -0.5 -1.0
f1 (ppm)

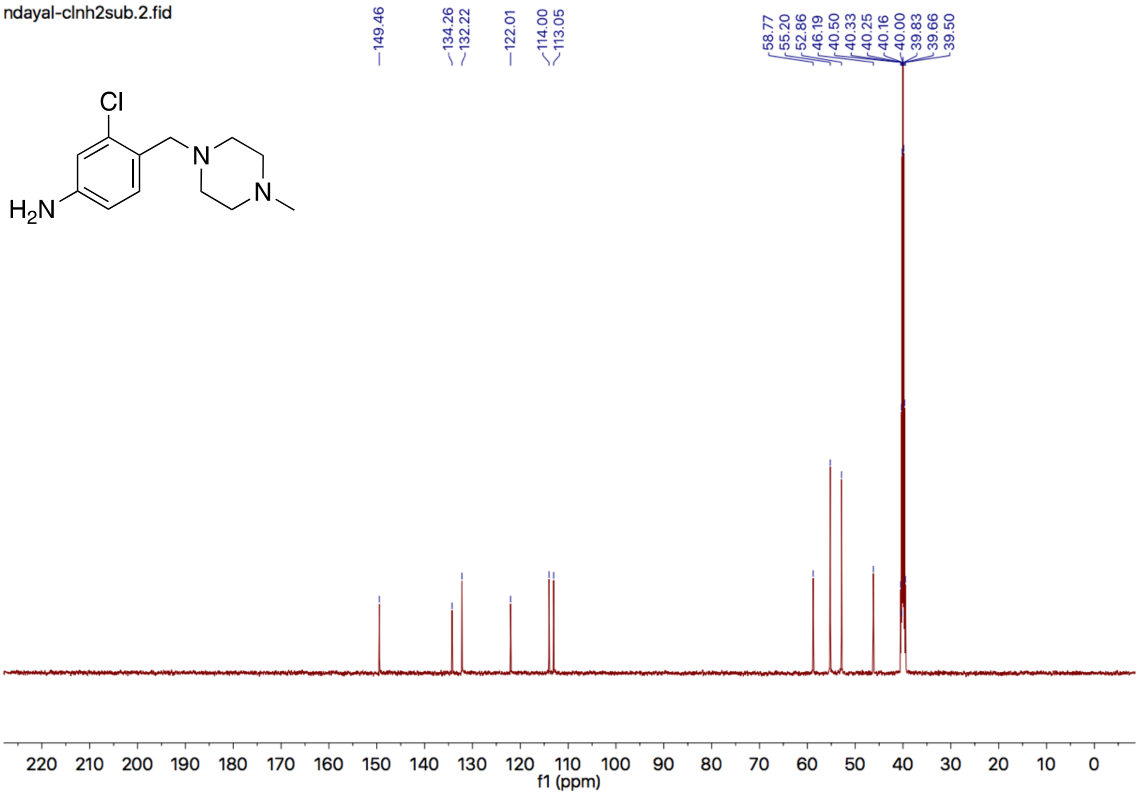
ndayal-clnh2sub.2.fid
149.46
134.26
132.22
122.01
114.00
113.05
58.77
55.20
52.86
46.19
40.50
40.33
40.25
40.16
40.00
39.83
39.66
39.50
Cl
N
N
H2N
220 210 200 190 180 170 160 150 140 130 120 110 100 90 80 70 60 50 40 30 20 10 0
f1 (ppm)

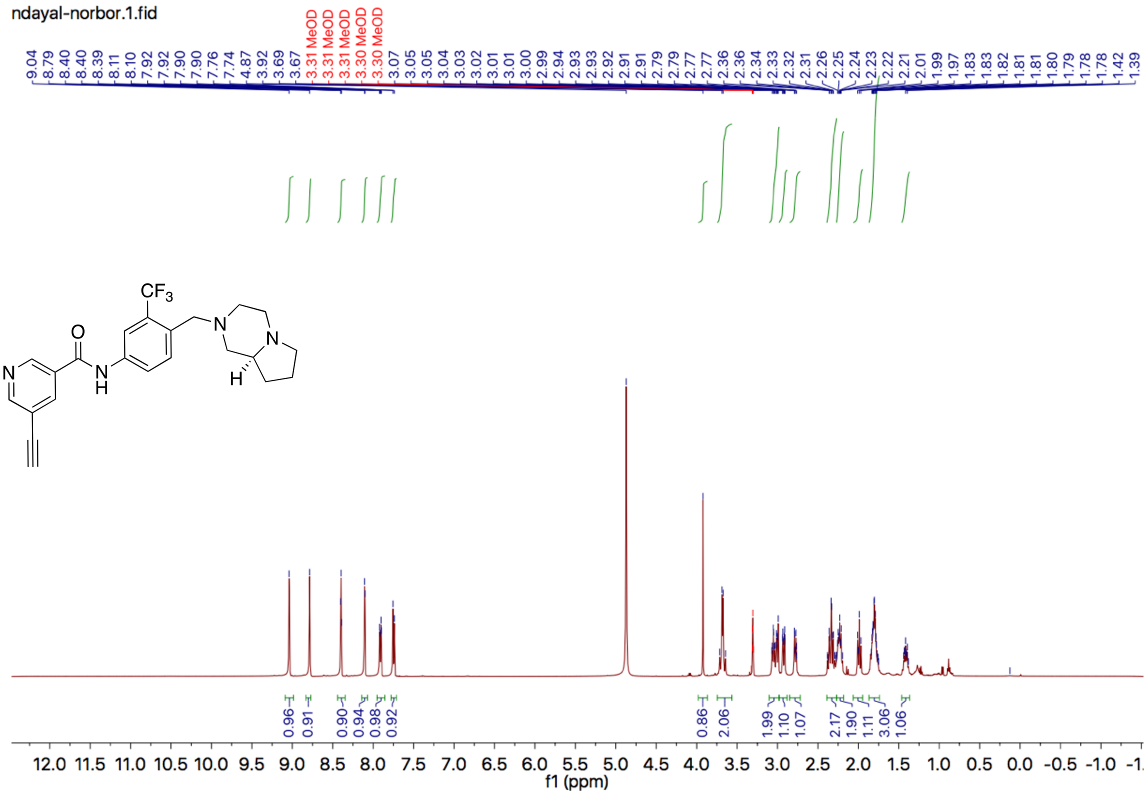
ndayal-norbor.1.fid
9.04 8.79 8.40 8.40 8.39 8.11 8.10 7.92 7.92 7.90 7.90 7.76 7.74 4.87 3.92 3.69 3.67
3.31 MeOD 3.31 MeOD 3.31 MeOD 3.30 MeOD 3.30 MeOD
3.07 3.05 3.05 3.04 3.03 3.02 3.01 3.01 3.00 2.99 2.94 2.93 2.93 2.92 2.91 2.91 2.79 2.79 2.77 2.77 2.36 2.36 2.34 2.33 2.32 2.31 2.26 2.25 2.24 2.23 2.22 2.21 2.01 1.99 1.97 1.83 1.83 1.81 1.81 1.80 1.79 1.78 1.78 1.42 1.39
CF3
O
N
H
0.96 0.91 0.90 0.94 0.98 0.92 0.86 2.06 1.99 1.10 1.07 2.17 1.90 1.11 3.06 1.06
f1 (ppm)

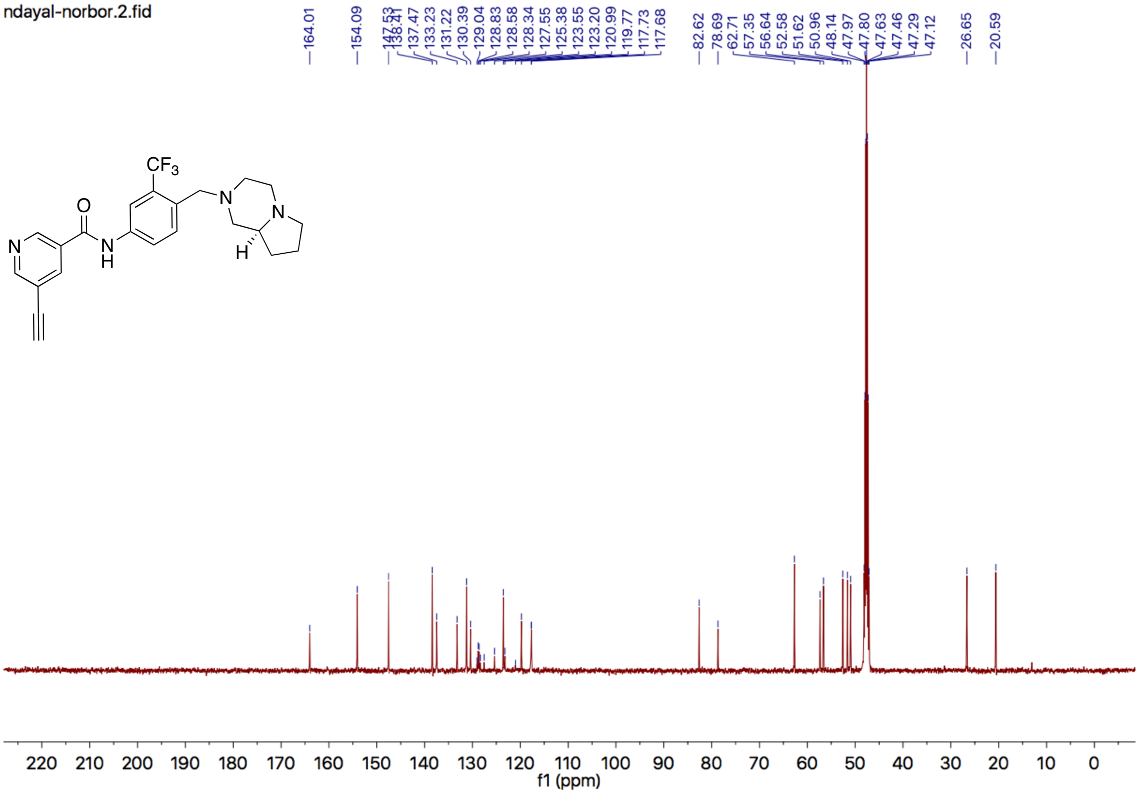
ndayal-norbor.2.fid
164.01 154.09 147.53 138.41 137.47 133.23 131.22 130.39 129.04 128.83 128.58 128.34 127.55 125.38 123.55 123.20 120.99 119.77 117.68
82.62 78.69 62.71 57.35 56.64 52.58 51.62 50.96 48.14 47.97 47.80 47.63 47.46 47.29 47.12 26.65 20.59
CF3
O
N
H
f1 (ppm)

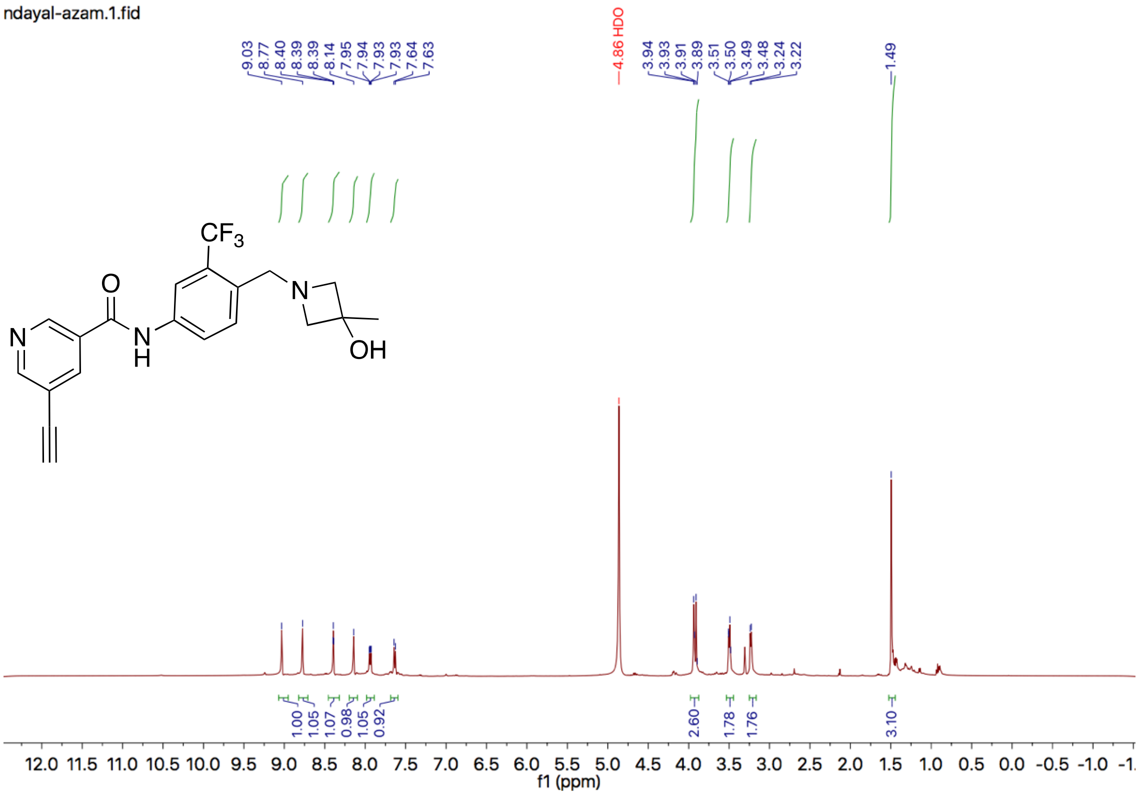
ndayal-azam.1.fid
9.03
8.77
8.40
8.39
8.39
8.14
7.95
7.94
7.93
7.93
7.64
7.63
4.86 HDO
3.94
3.93
3.91
3.89
3.51
3.50
3.49
3.48
3.24
3.22
1.49
CF3
O
N
H
N
N
OH
1.00
1.05
1.07
0.98
1.05
0.92
2.60
1.78
1.76
3.10
12.0 11.5 11.0 10.5 10.0 9.5 9.0 8.5 8.0 7.5 7.0 6.5 6.0 5.5 5.0 4.5 4.0 3.5 3.0 2.5 2.0 1.5 1.0 0.5 0.0 -0.5 -1.0
f1 (ppm)

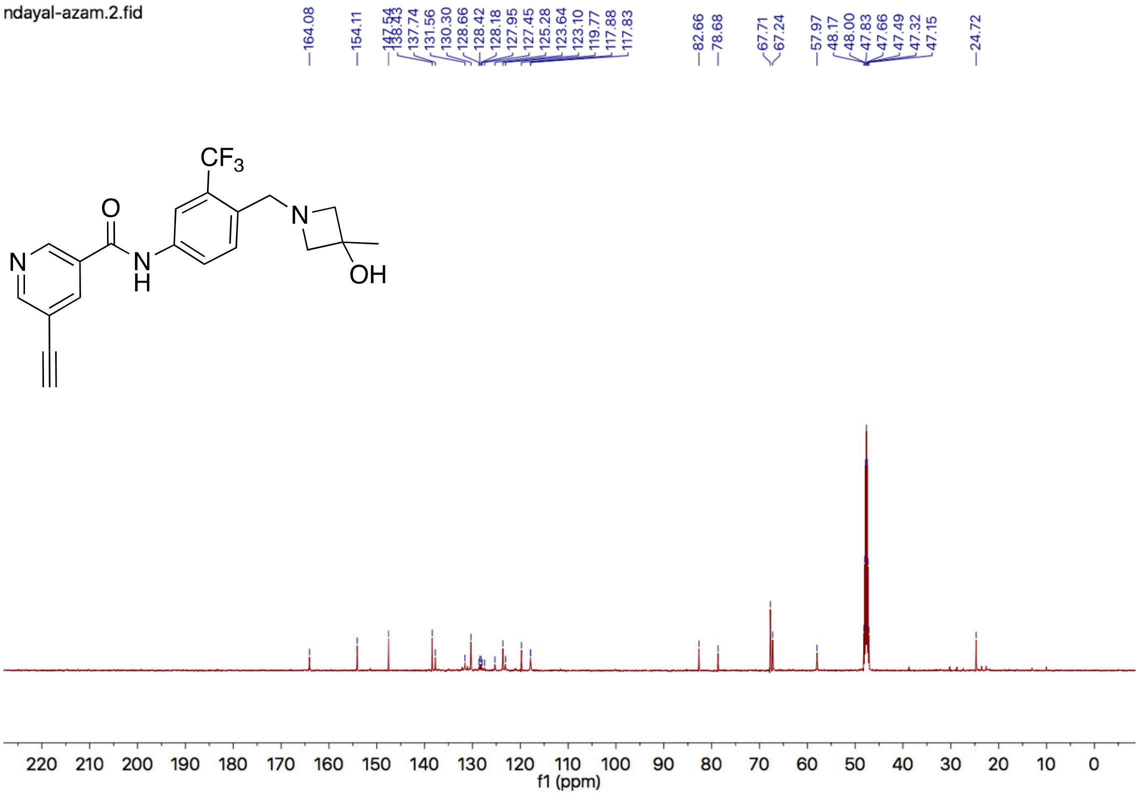
ndayal-azam.2.fid
164.08
154.11
147.54
138.43
137.74
131.56
130.30
128.66
128.42
128.18
127.95
127.45
125.28
123.64
123.10
119.77
117.88
117.83
82.66
78.68
67.71
67.24
57.97
48.17
48.00
47.83
47.66
47.49
47.32
47.15
24.72
CF3
O
N
H
N
N
OH
220 210 200 190 180 170 160 150 140 130 120 110 100 90 80 70 60 50 40 30 20 10 0
f1 (ppm)

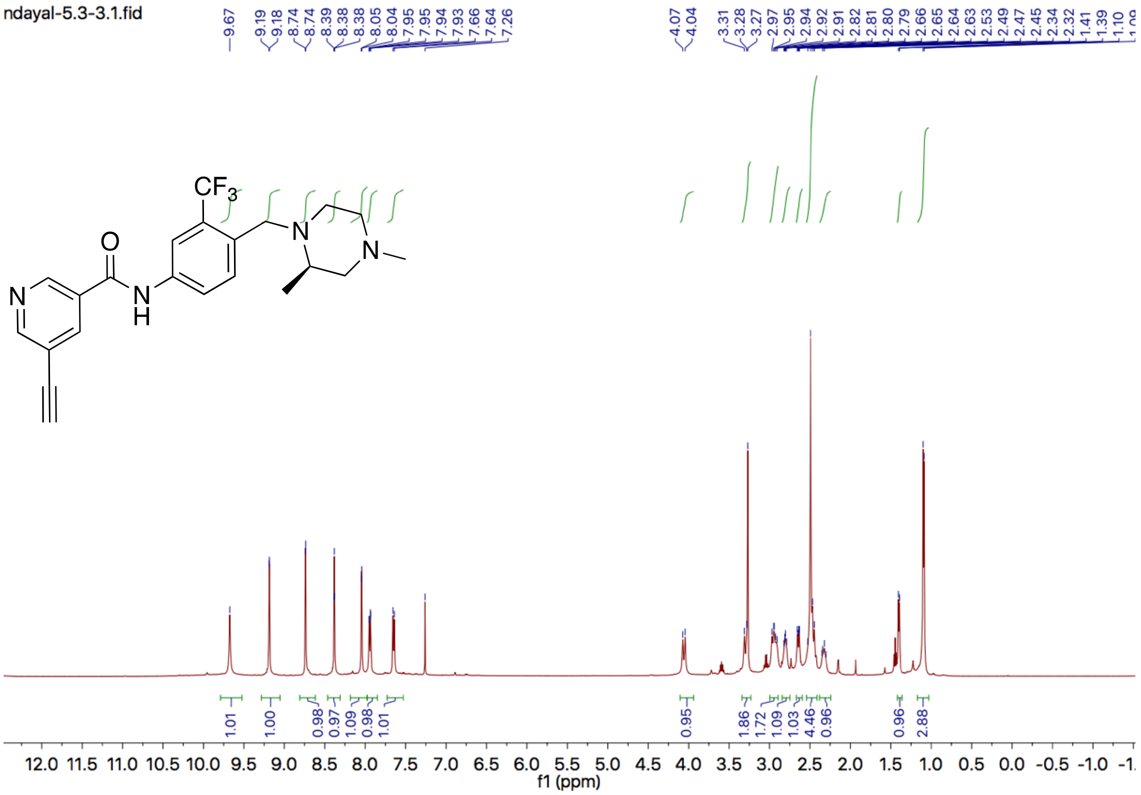
ndayal-5.3-3.1.fid
f1 (ppm)

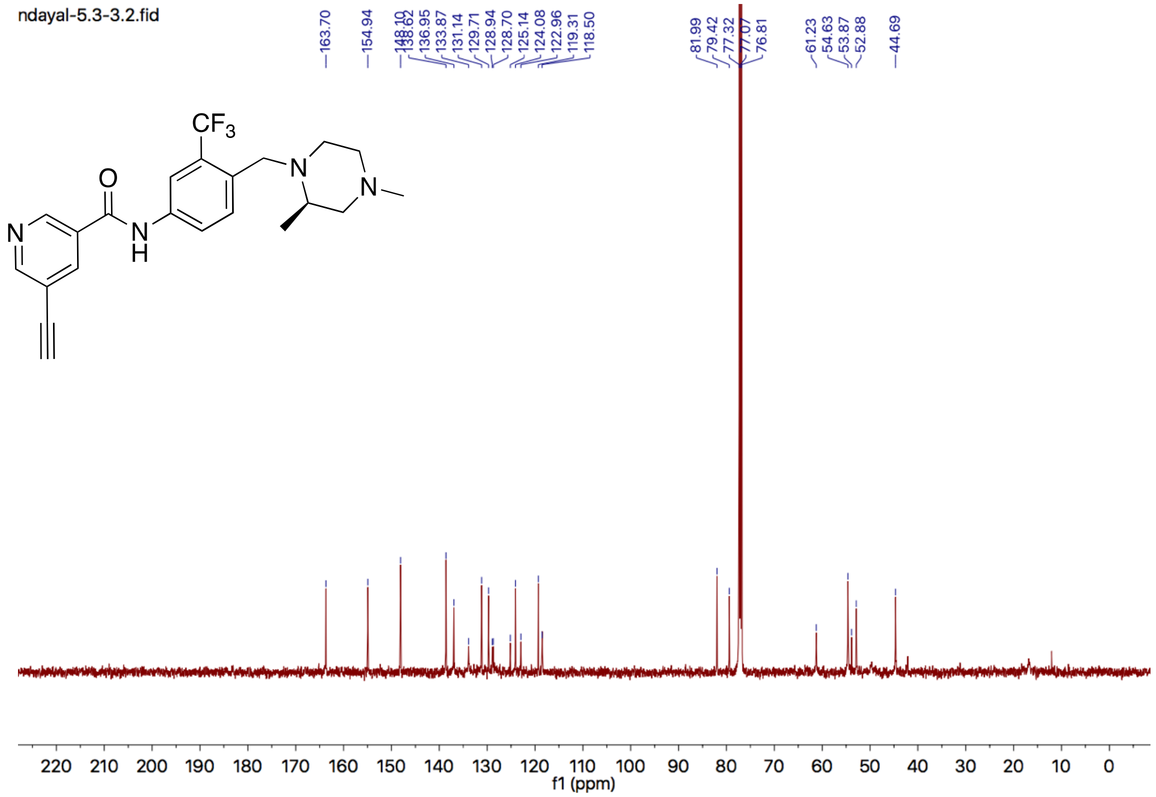
ndayal-5.3-3.2.fid
f1 (ppm)

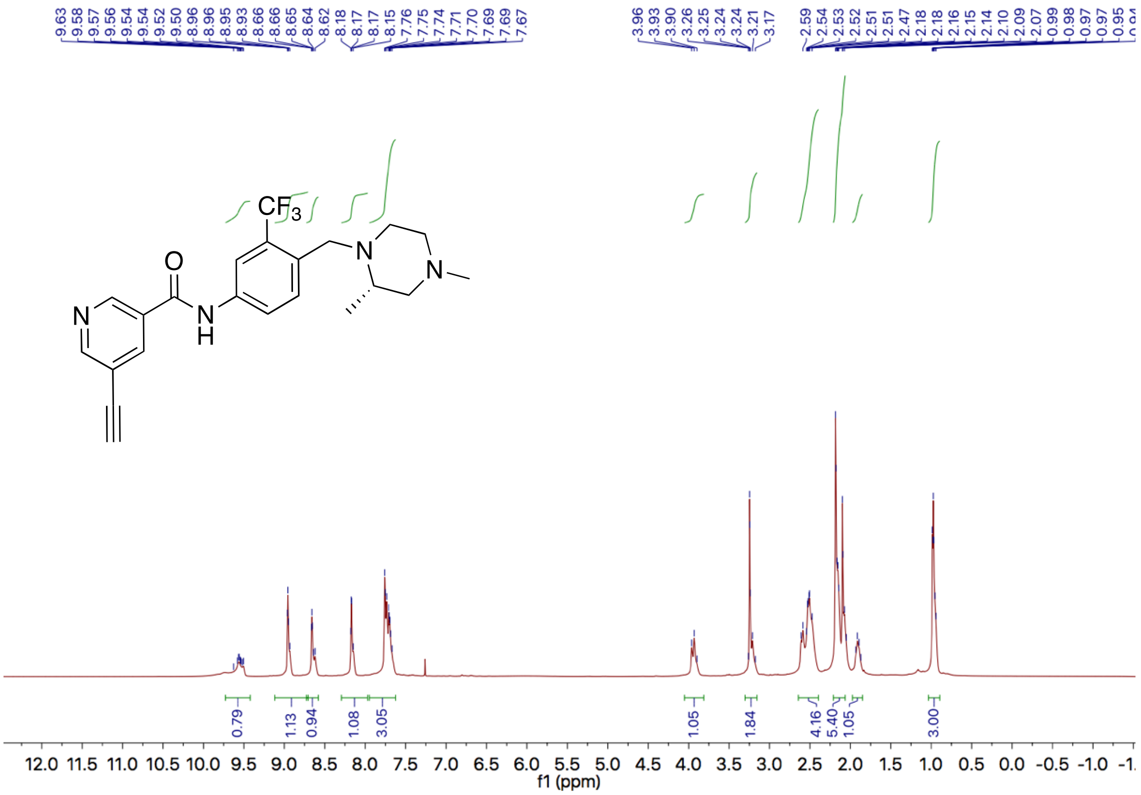

9.63 9.58 9.57 9.56 9.54 9.54 9.52 9.50 8.96 8.96 8.95 8.93 8.66 8.66 8.65 8.64 8.62 8.18 8.17 8.17 8.15 7.76 7.75 7.74 7.71 7.70 7.69 7.69 7.67
3.96 3.93 3.90 3.26 3.25 3.24 3.24 3.21 3.17
2.59 2.54 2.53 2.52 2.51 2.51 2.47 2.18 2.18 2.16 2.15 2.14 2.10 2.09 2.07 0.99 0.98 0.97 0.97 0.95
CF3
O
N
N
H
N
N
0.79
1.13
0.94
1.08
3.05
1.05
1.84
4.16
5.40
1.05
3.00
12.0 11.5 11.0 10.5 10.0 9.5 9.0 8.5 8.0 7.5 7.0 6.5 6.0 5.5 5.0 4.5 4.0 3.5 3.0 2.5 2.0 1.5 1.0 0.5 0.0 -0.5 -1.0
f1 (ppm)

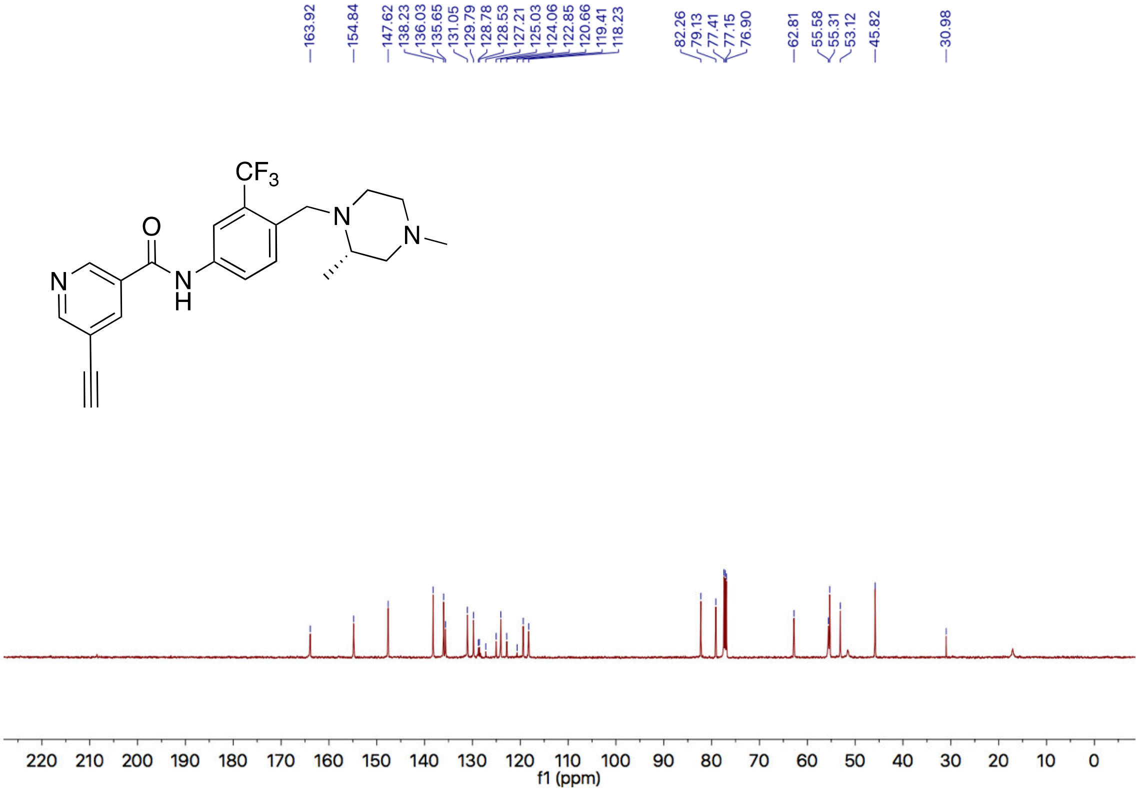

163.92
154.84
147.62 138.23 136.03 135.65 131.05 129.79 128.78 128.53 127.21 125.03 124.06 122.85 120.66 119.41 118.23
82.26 79.13 77.41 77.15 76.90
62.81
55.58 55.31 53.12
45.82
30.98
CF3
O
N
N
H
N
N
220 210 200 190 180 170 160 150 140 130 120 110 100 90 80 70 60 50 40 30 20 10 0
f1 (ppm)

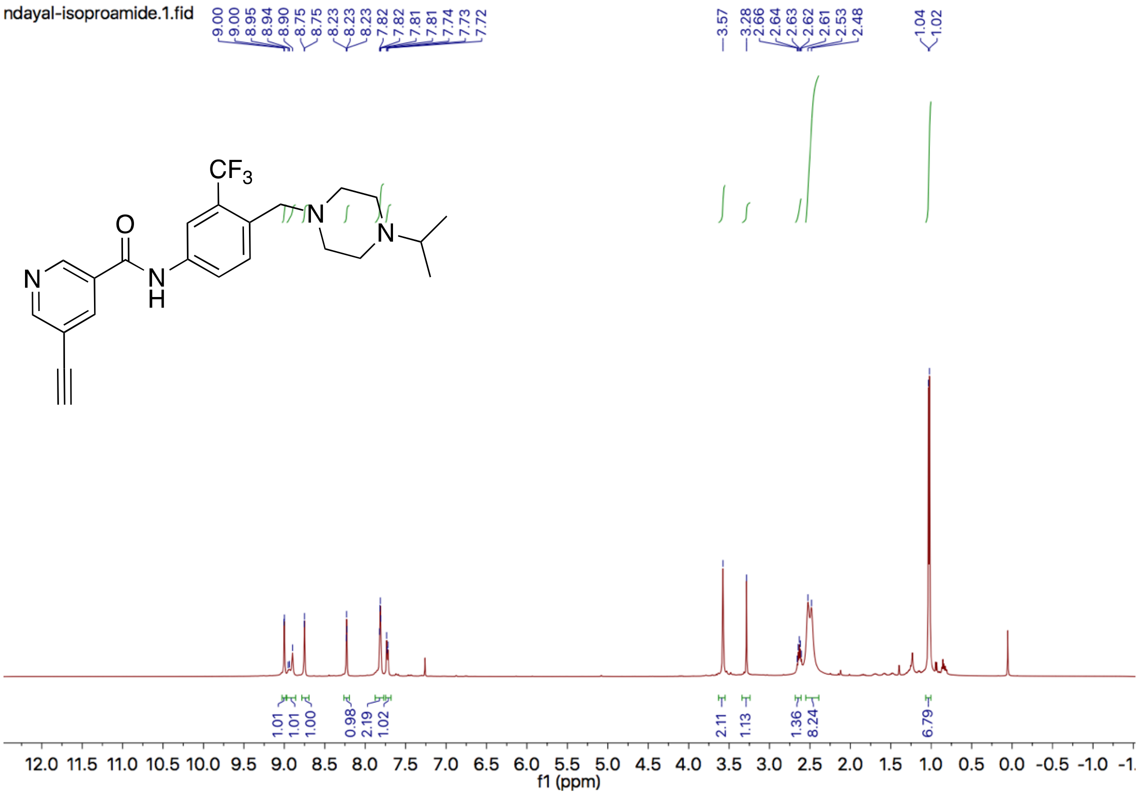
ndayal-isoproamide.1.fid
9.00 9.00 8.95 8.94 8.90 8.75 8.75 8.23 8.23 8.23 7.82 7.82 7.81 7.81 7.74 7.73 7.72
3.57 3.28 2.66 2.64 2.63 2.62 2.61 2.53 2.48
1.04 1.02
CF3
O
N
N
H
N
N
1.01 1.01 1.00 0.98 2.19 1.02 2.11 1.13 1.36 8.24 6.79
12.0 11.5 11.0 10.5 10.0 9.5 9.0 8.5 8.0 7.5 7.0 6.5 6.0 5.5 5.0 4.5 4.0 3.5 3.0 2.5 2.0 1.5 1.0 0.5 0.0 -0.5 -1.0
f1 (ppm)

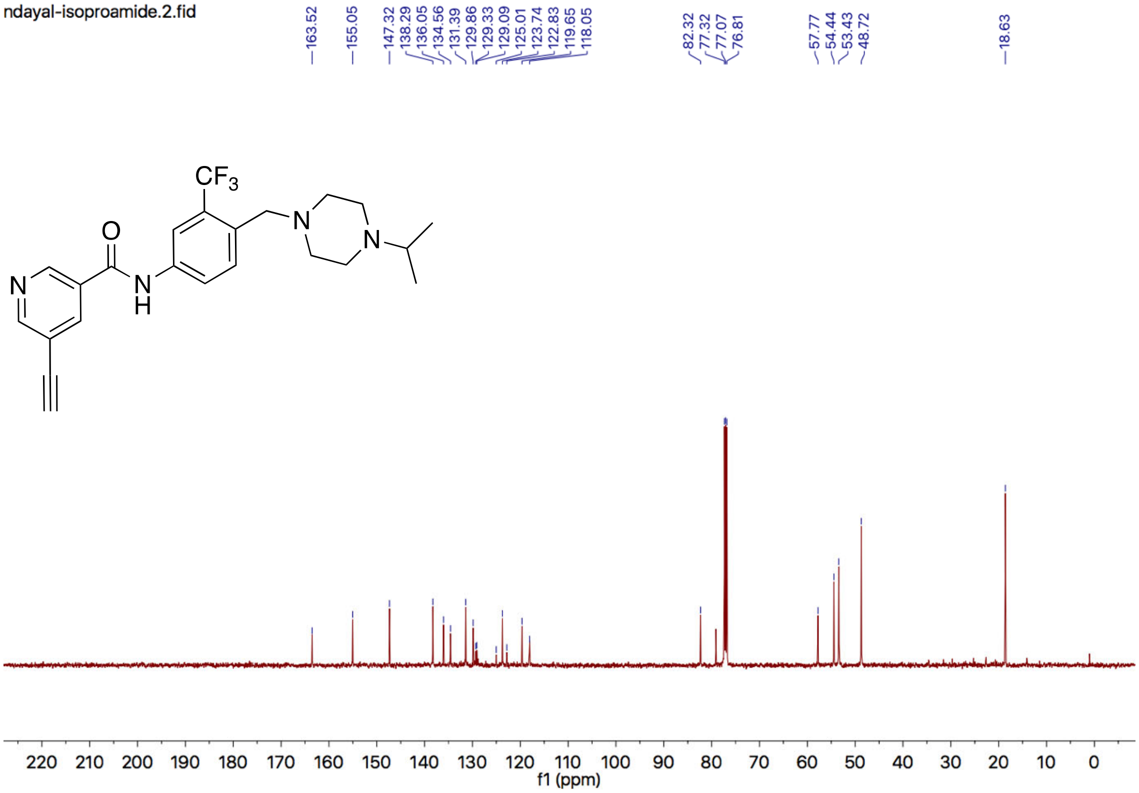
ndayal-isoproamide.2.fid
163.52 155.05 147.32 138.29 136.05 134.56 131.39 129.86 129.33 129.09 125.01 123.74 122.83 119.65 118.05
82.32 77.32 77.07 76.81
57.77 54.44 53.43 48.72
18.63
CF3
O
N
N
H
N
N
220 210 200 190 180 170 160 150 140 130 120 110 100 90 80 70 60 50 40 30 20 10 0
f1 (ppm)

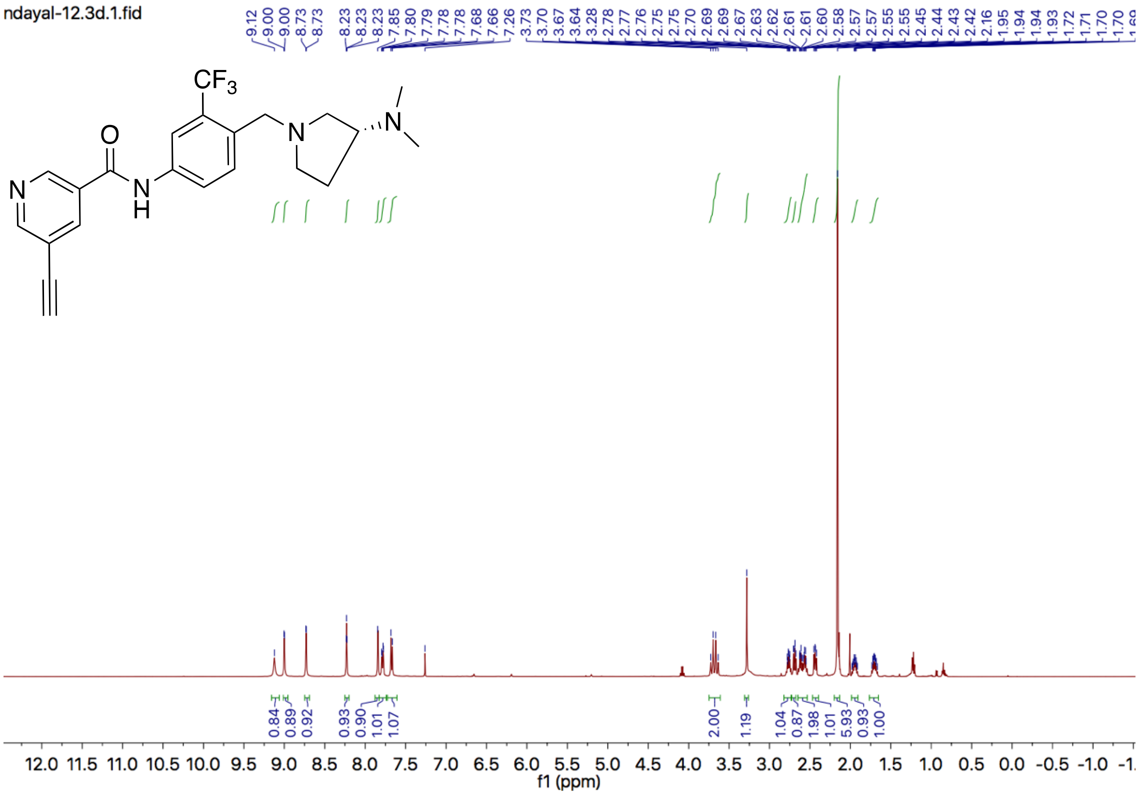
ndayal-12.3d.1.fid
9.12 9.00 9.00 8.73 8.73 8.23 8.23 8.23 7.85 7.80 7.79 7.78 7.78 7.68 7.66 7.26 3.73 3.70 3.67 3.64 3.28 2.78 2.77 2.76 2.75 2.75 2.70 2.69 2.69 2.67 2.63 2.62 2.61 2.61 2.60 2.58 2.57 2.57 2.55 2.55 2.45 2.44 2.43 2.42 2.16 1.95 1.94 1.93 1.72 1.71 1.70
CF3
O
N
H
N
N
0.84 0.89 0.92 0.93 0.90 1.01 1.07 2.00 1.19 1.04 0.87 1.98 1.01 5.93 0.93 1.00
12.0 11.5 11.0 10.5 10.0 9.5 9.0 8.5 8.0 7.5 7.0 6.5 6.0 5.5 5.0 4.5 4.0 3.5 3.0 2.5 2.0 1.5 1.0 0.5 0.0 -0.5 -1.0
f1 (ppm)

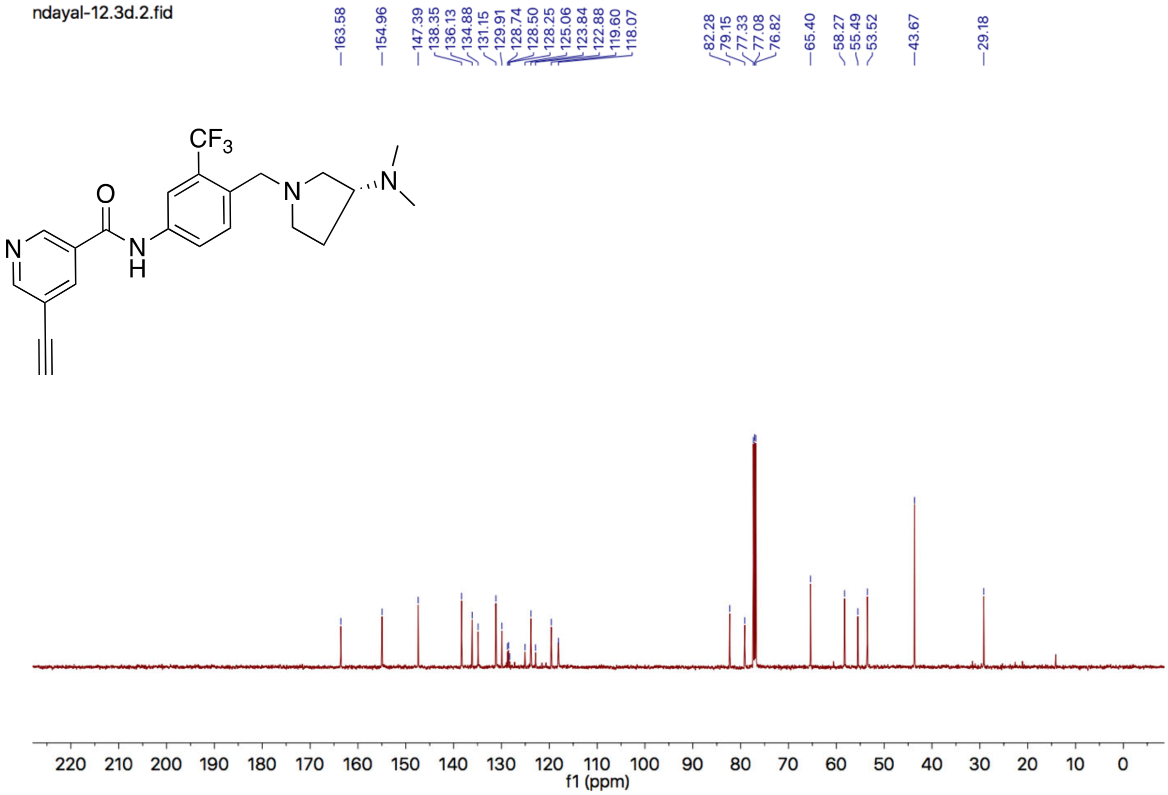
ndayal-12.3d.2.fid
163.58 154.96 147.39 138.35 136.13 134.88 131.15 129.91 128.74 128.50 128.25 125.06 123.84 122.88 119.60 118.07 82.28 79.15 77.33 77.08 76.82 65.40 58.27 55.49 53.52 43.67 29.18
CF3
O
N
H
N
N
220 210 200 190 180 170 160 150 140 130 120 110 100 90 80 70 60 50 40 30 20 10 0
f1 (ppm)

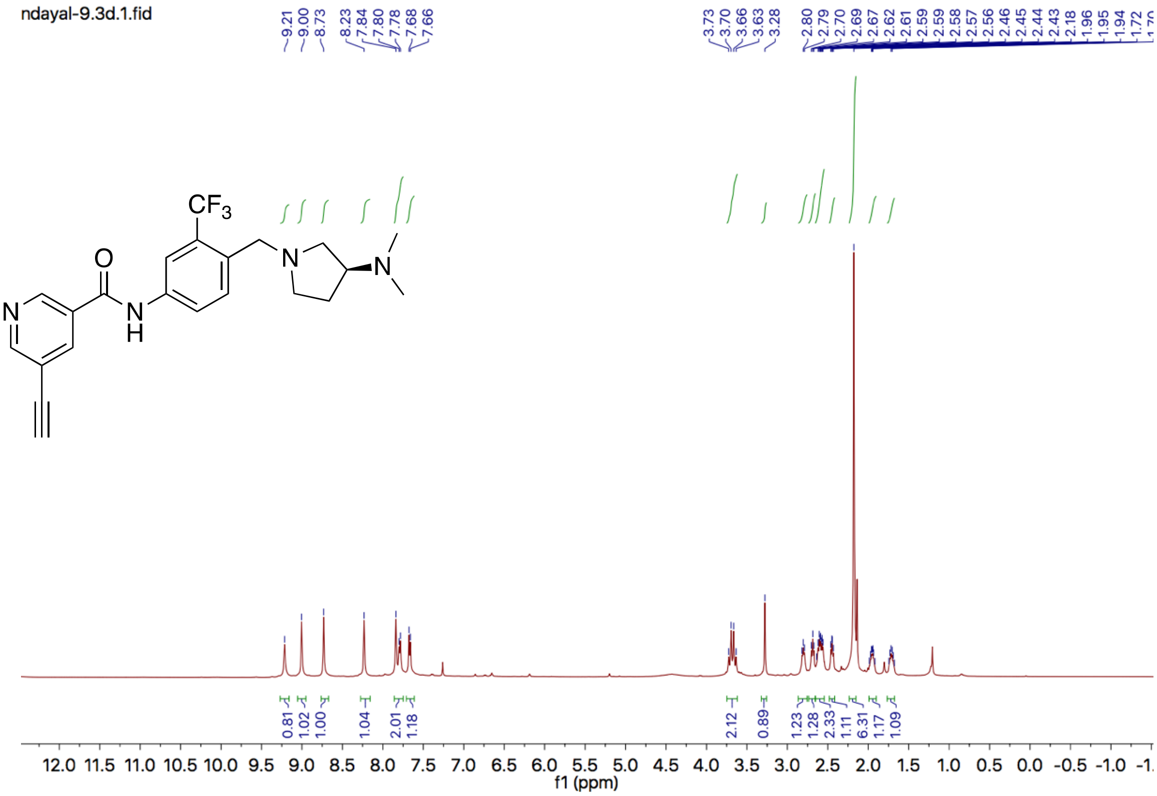
ndayal-9.3d.1.fid
9.21
9.00
8.73
8.23
7.84
7.80
7.78
7.68
7.66
3.73
3.70
3.66
3.63
3.28
2.80
2.79
2.70
2.69
2.67
2.62
2.61
2.59
2.59
2.58
2.57
2.56
2.46
2.45
2.44
2.43
2.18
1.96
1.95
1.94
1.72
CF3
O
N
H
N
N
0.81
1.02
1.00
1.04
2.01
1.18
2.12
0.89
1.23
1.28
2.33
1.11
6.31
1.17
1.09
f1 (ppm)

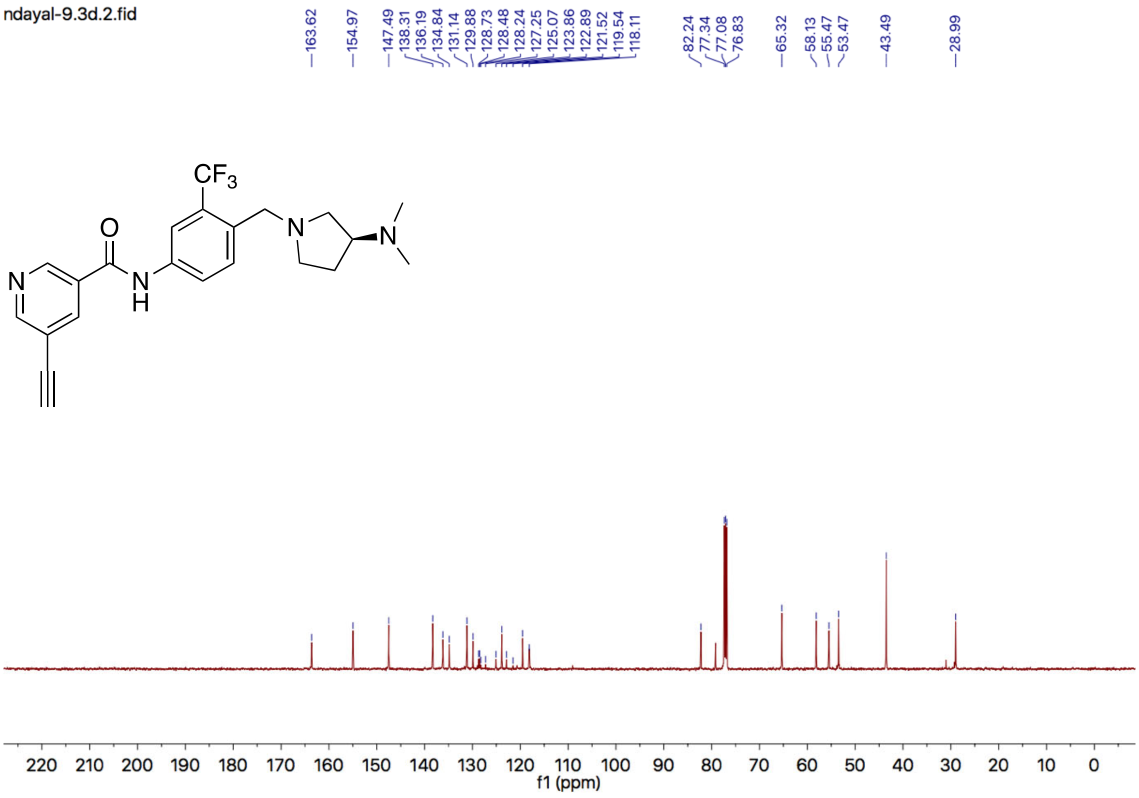
ndayal-9.3d.2.fid
163.62
154.97
147.49
138.31
136.19
134.84
131.14
129.88
128.73
128.48
128.24
127.25
125.07
123.86
122.89
121.52
119.54
118.11
82.24
77.34
77.08
76.83
65.32
58.13
55.47
53.47
43.49
28.99
CF3
O
N
H
N
N
f1 (ppm)

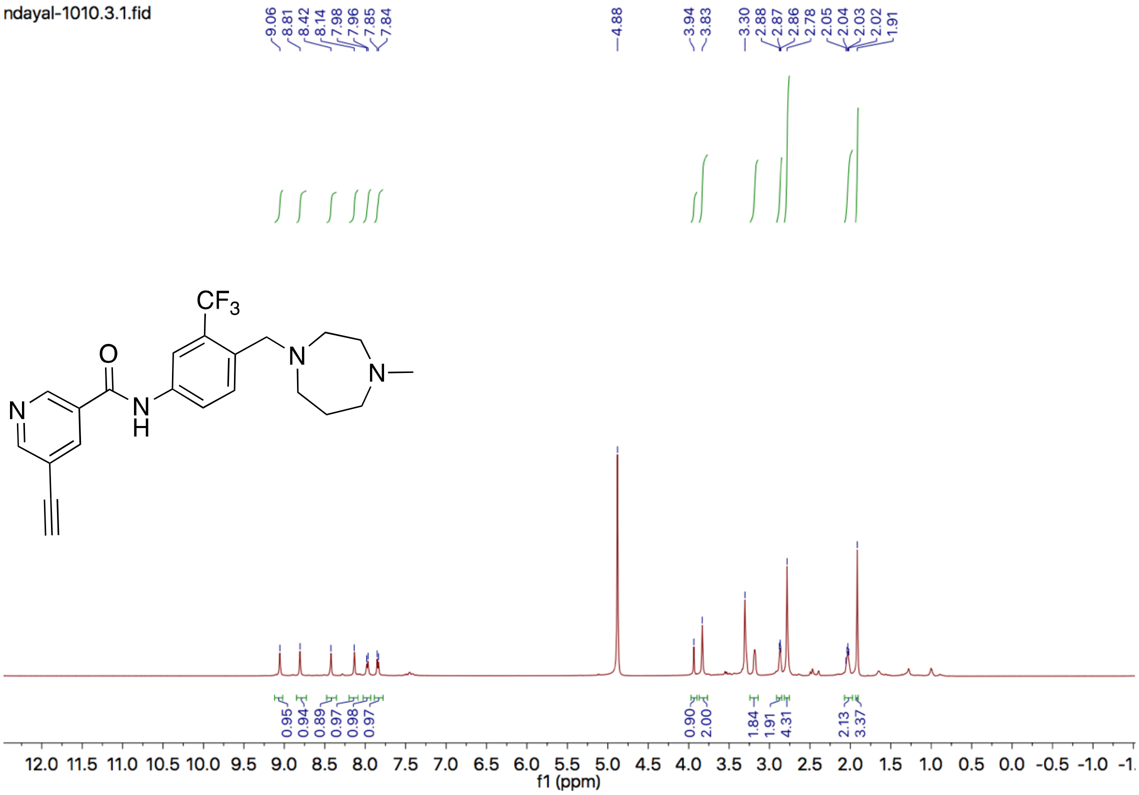

ndayal-1010.3.1.fid
9.06 8.81 8.42 8.14 7.98 7.96 7.85 7.84
4.88
3.94 3.83
3.30 2.88 2.87 2.86 2.78 2.05 2.04 2.03 2.02 1.91
CF3
O
N
H
N
N
0.95 0.94 0.89 0.97 0.98 0.97
0.90 2.00
1.84 1.91 4.31
2.13 3.37
f1 (ppm)

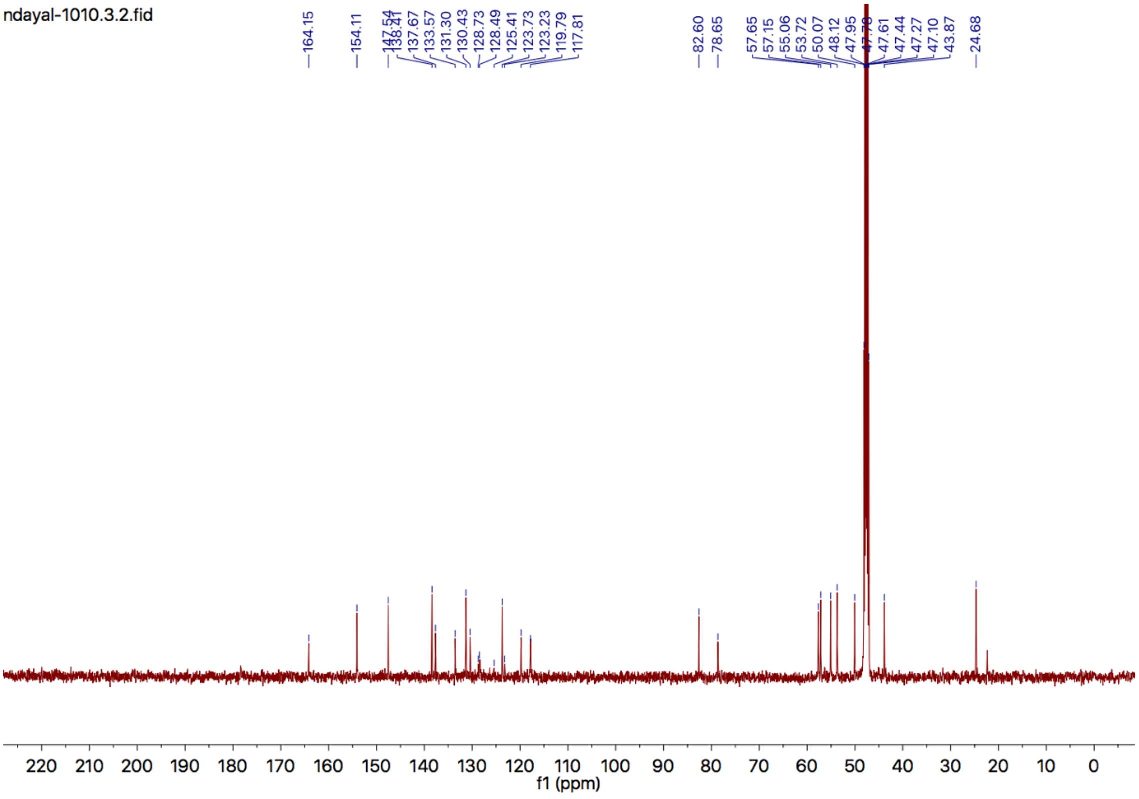

ndayal-1010.3.2.fid
164.15
154.11
137.67 133.57 131.30 130.43 128.73 128.49 125.41 123.73 123.23 119.79 117.81
82.60 78.65
57.65 57.15 55.06 53.72 50.07 48.12 47.95 47.61 47.44 47.27 47.10 43.87
24.68
f1 (ppm)

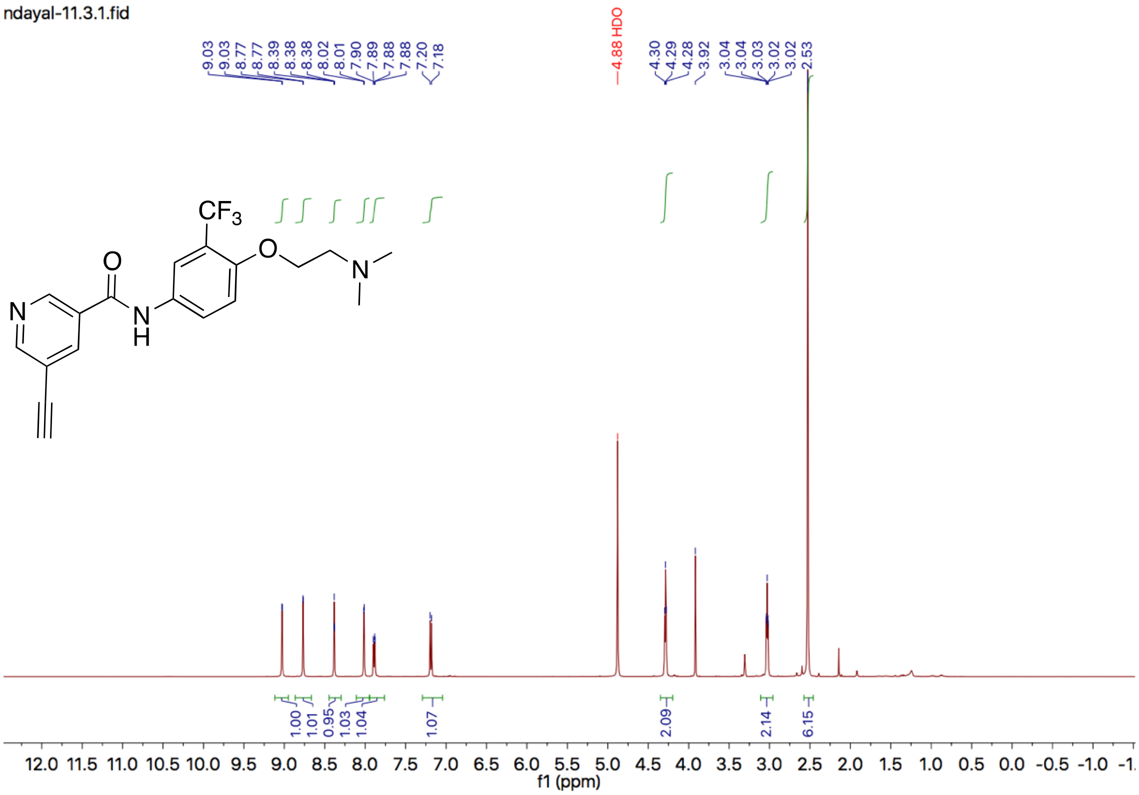
ndayal-11.3.1.fid
9.03 9.03 8.77 8.77 8.39 8.38 8.38 8.02 8.01 7.90 7.89 7.88 7.88 7.20 7.18
4.88 HDO
4.30 4.29 4.28 3.92 3.04 3.04 3.03 3.02 3.02 2.53
1.00 1.01 0.95 1.03 1.04 1.07 2.09 2.14 6.15
CF3
O
N
NH
f1 (ppm)

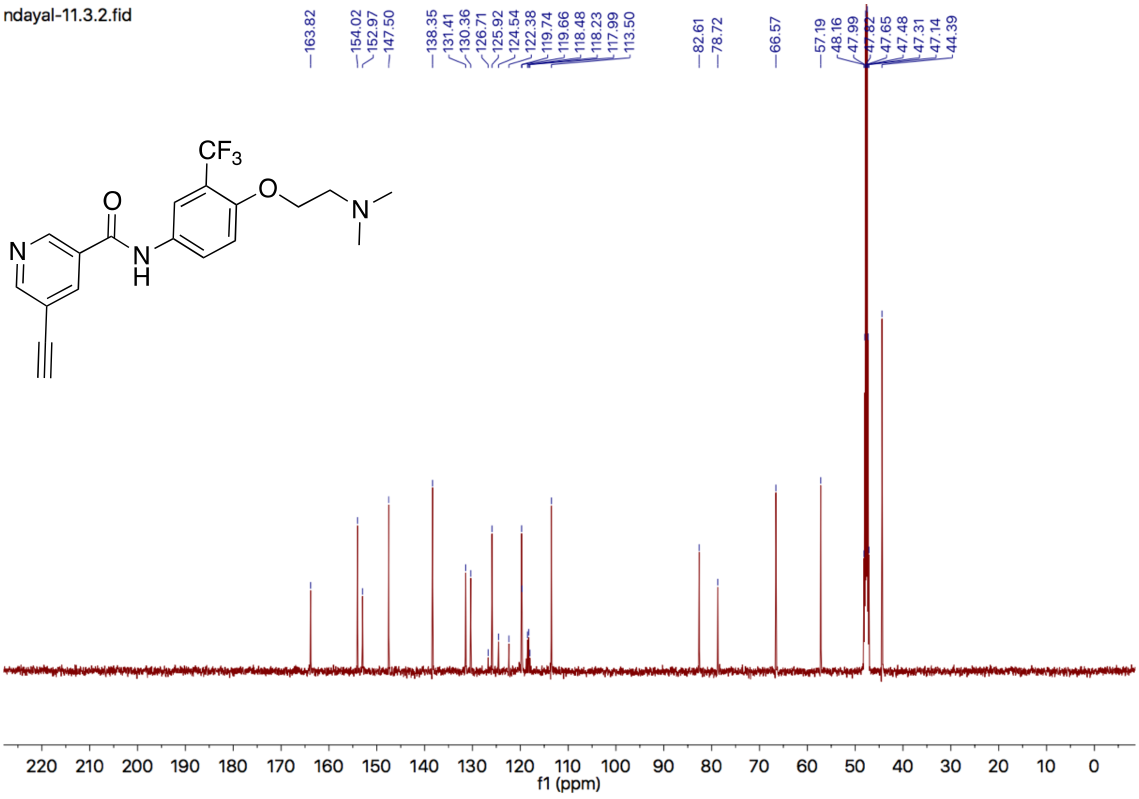
ndayal-11.3.2.fid
163.82
154.02 152.97 147.50
138.35 131.41 130.36 126.71 125.92 124.54 122.38 119.74 119.66 118.48 118.23 117.99 113.50
82.61 78.72
66.57
57.19 48.16 47.99 47.82 47.65 47.48 47.31 47.14 44.39
CF3
O
N
NH
f1 (ppm)

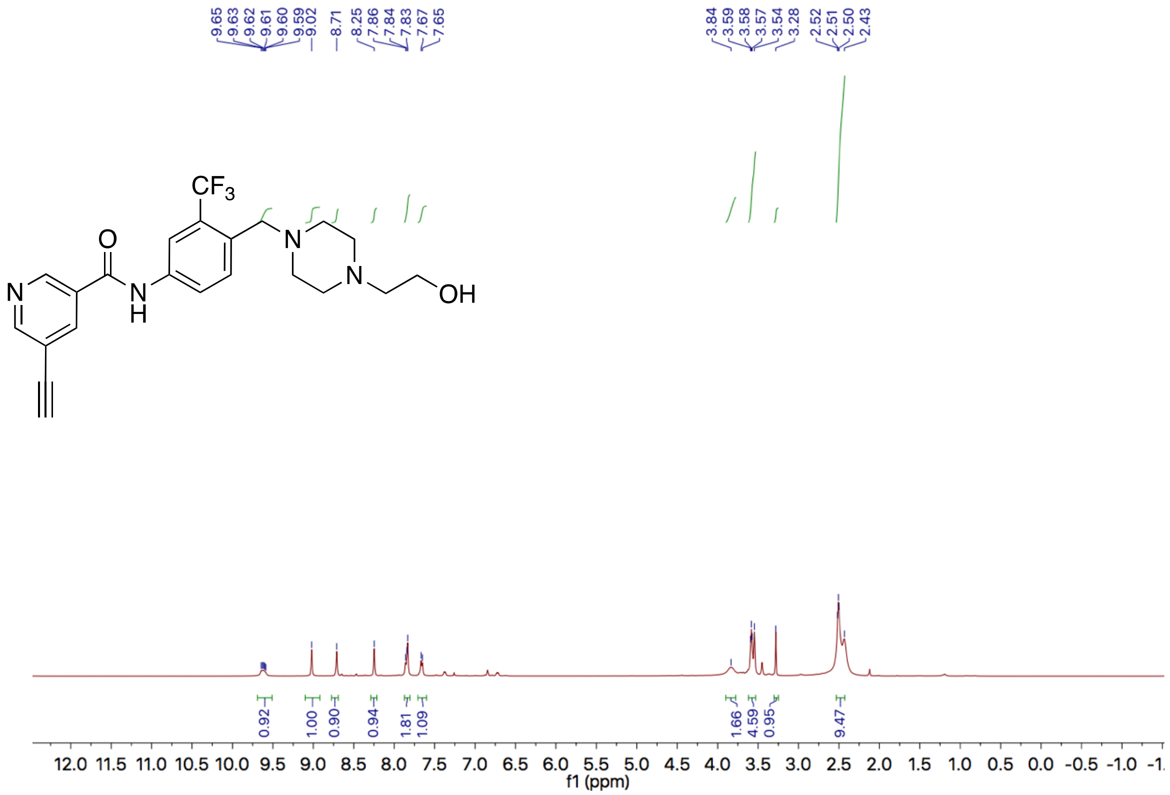

9.65
9.63
9.62
9.61
9.60
9.59
9.02
8.71
8.25
7.86
7.84
7.83
7.67
7.65
3.84
3.59
3.58
3.57
3.54
3.28
2.52
2.51
2.50
2.43
CF3
O
N
H
N
N
OH
0.92
1.00
0.90
0.94
1.81
1.09
1.66
4.59
0.95
9.47
12.0 11.5 11.0 10.5 10.0 9.5 9.0 8.5 8.0 7.5 7.0 6.5 6.0 5.5 5.0 4.5 4.0 3.5 3.0 2.5 2.0 1.5 1.0 0.5 0.0 -0.5 -1.0
f1 (ppm)

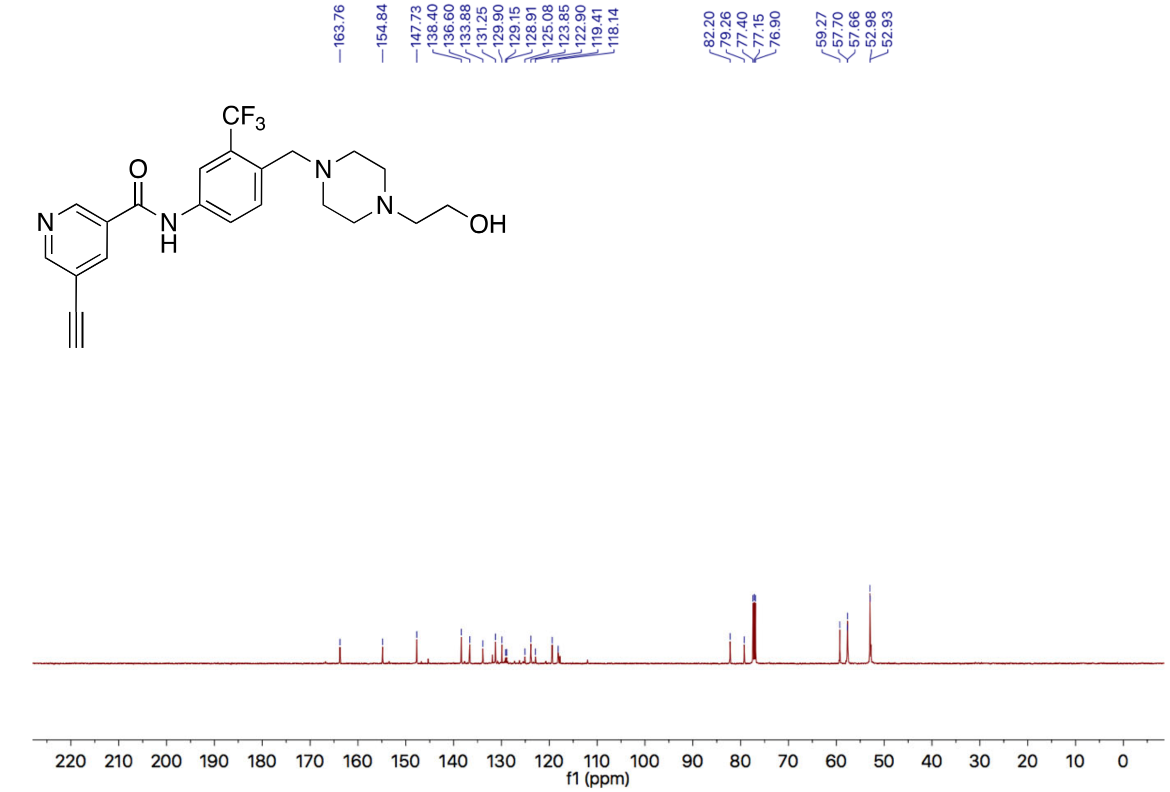

163.76
154.84
147.73
138.40
136.60
133.88
131.25
129.90
129.15
128.91
125.08
123.85
122.90
119.41
118.14
82.20
79.26
77.40
77.15
76.90
59.27
57.70
57.66
52.98
52.93
CF3
O
N
H
N
N
OH
220 210 200 190 180 170 160 150 140 130 120 110 100 90 80 70 60 50 40 30 20 10 0
f1 (ppm)

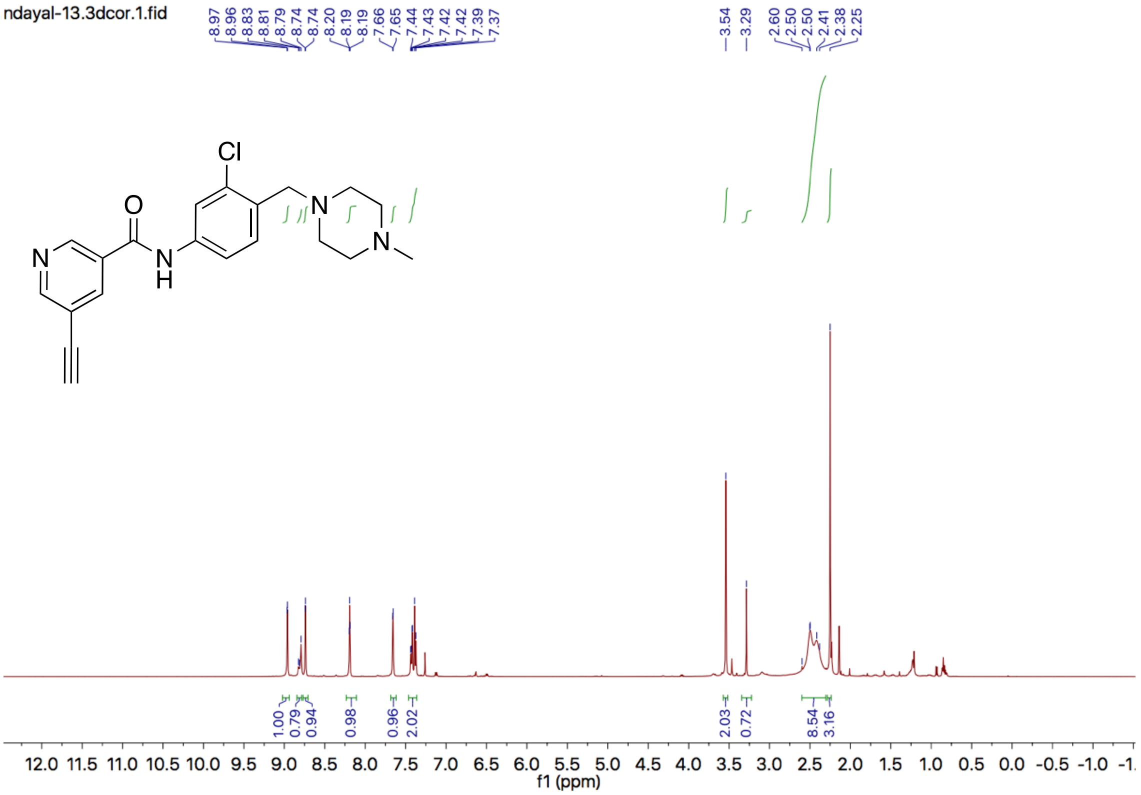
ndayal-13.3dcor.1.fid
8.97 8.96 8.83 8.81 8.79 8.74 8.74 8.20 8.19 8.19 7.66 7.65 7.44 7.43 7.42 7.42 7.39 7.37
3.54 3.29 2.60 2.50 2.50 2.41 2.38 2.25
Cl
O
N
N
H
N
N
1.00 0.79 0.94 0.98 0.96 2.02 2.03 0.72 8.54 3.16
12.0 11.5 11.0 10.5 10.0 9.5 9.0 8.5 8.0 7.5 7.0 6.5 6.0 5.5 5.0 4.5 4.0 3.5 3.0 2.5 2.0 1.5 1.0 0.5 0.0 -0.5 -1.0
f1 (ppm)

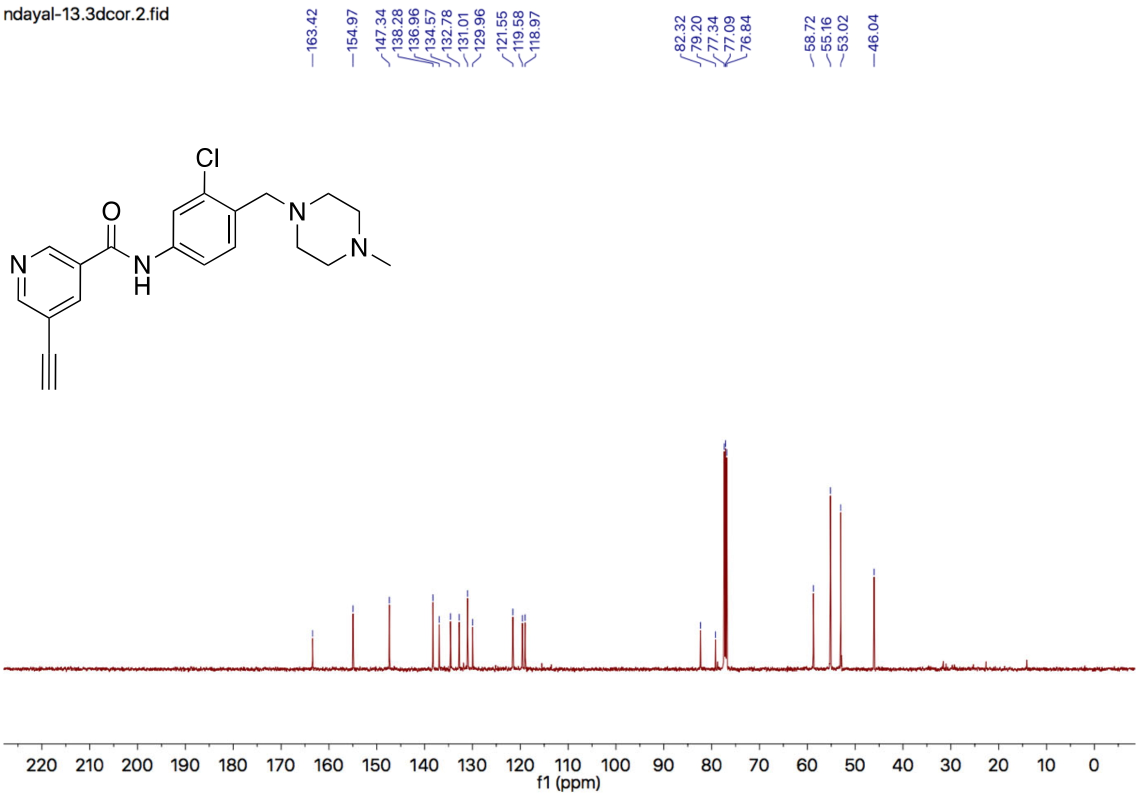
ndayal-13.3dcor.2.fid
163.42 154.97 147.34 138.28 136.96 134.57 132.78 131.01 129.96 121.55 119.58 118.97
82.32 79.20 77.34 77.09 76.84 58.72 55.16 53.02 46.04
Cl
O
N
N
H
N
N
220 210 200 190 180 170 160 150 140 130 120 110 100 90 80 70 60 50 40 30 20 10 0
f1 (ppm)

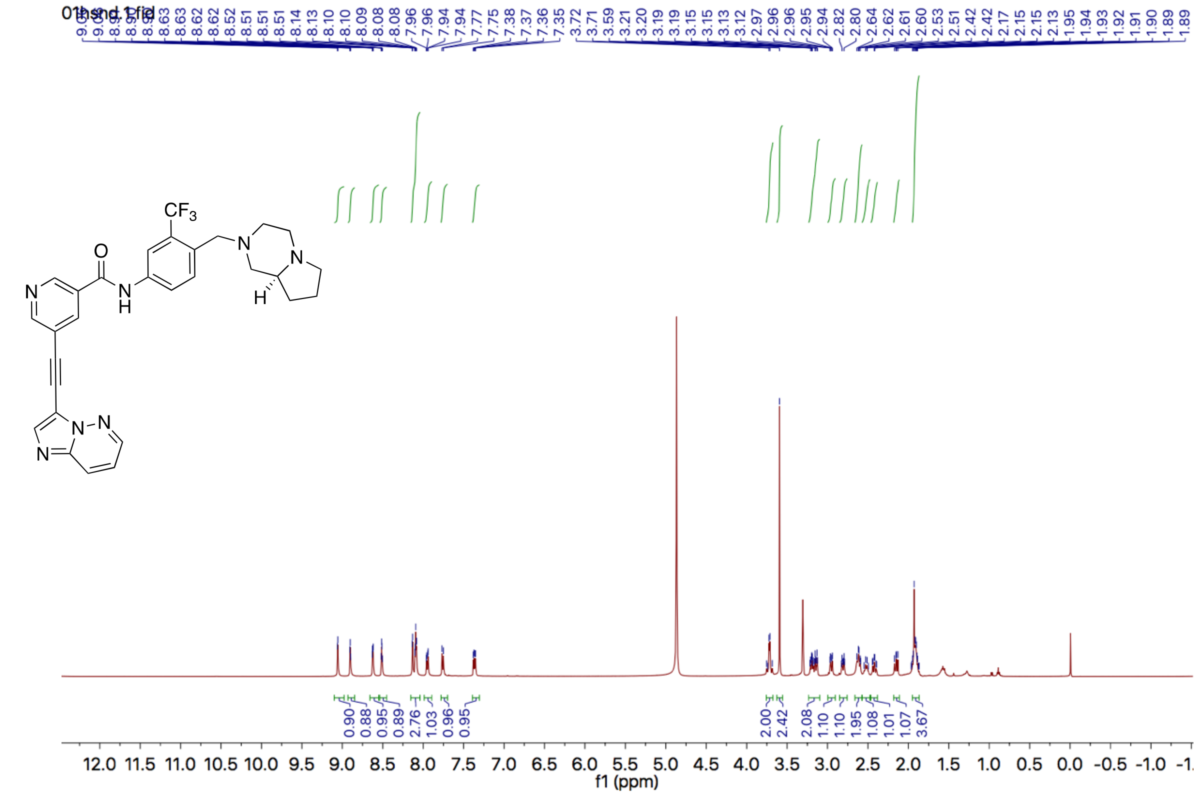
01hsnd.1.fid
8.63 8.63 8.62 8.62 8.52 8.51 8.51 8.51 8.14 8.13 8.10 8.10 8.09 8.08 8.08 7.96 7.96 7.94 7.94 7.77 7.75 7.38 7.37 7.36 7.35 3.72 3.71 3.59 3.21 3.20 3.19 3.19 3.15 3.15 3.13 3.12 2.97 2.96 2.96 2.95 2.94 2.82 2.80 2.64 2.62 2.61 2.60 2.53 2.51 2.42 2.42 2.17 2.15 2.15 2.13 1.95 1.94 1.93 1.92 1.91 1.90 1.89 1.89
CF3
O
N
N
H
N
N
H
N
N
N
0.90 0.88 0.95 0.89 2.76 1.03 0.96 0.95
2.00 2.42 2.08 1.10 1.10 1.95 1.08 1.01 1.07 3.67
12.0 11.5 11.0 10.5 10.0 9.5 9.0 8.5 8.0 7.5 7.0 6.5 6.0 5.5 5.0 4.5 4.0 3.5 3.0 2.5 2.0 1.5 1.0 0.5 0.0 -0.5 -1.0
f1 (ppm)

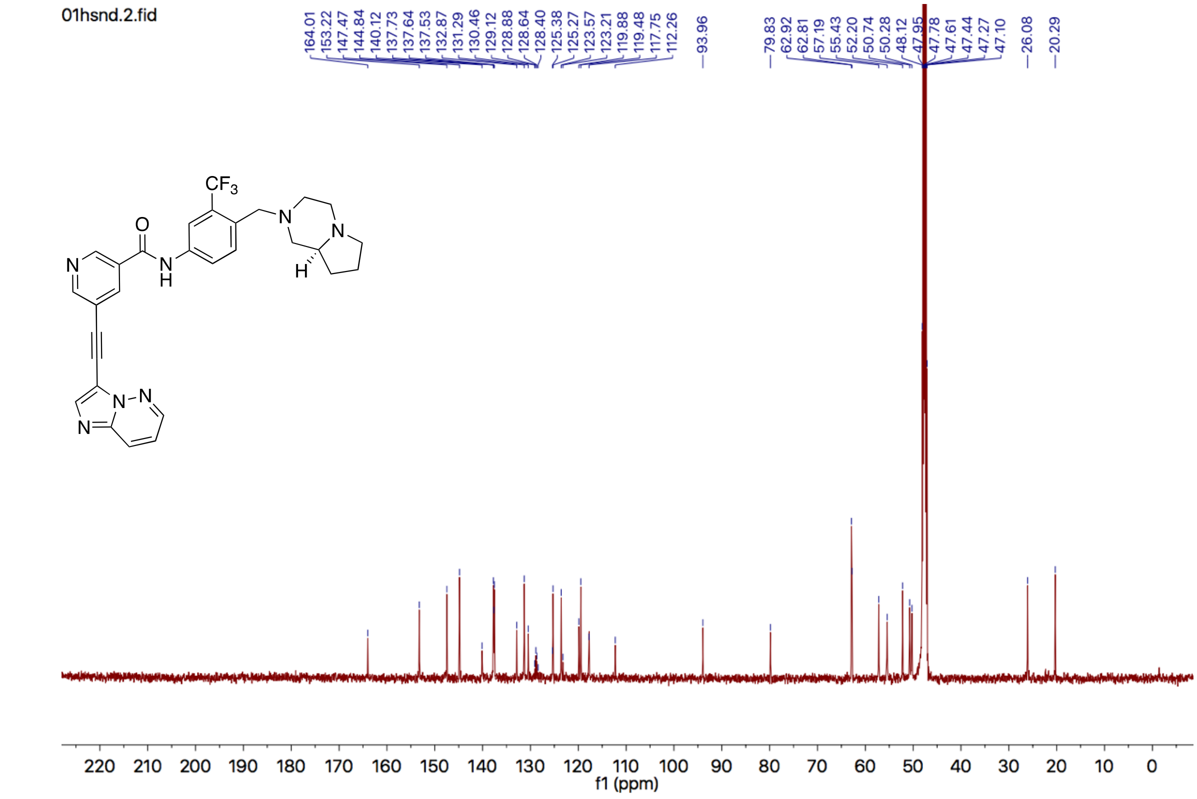
01hsnd.2.fid
164.01 153.22 147.47 144.84 140.12 137.73 137.64 137.53 132.87 131.29 130.46 129.12 128.88 128.64 128.40 125.38 125.27 123.57 123.21 119.88 119.48 117.75 112.26
93.96
79.83 62.92 62.81 57.19 55.43 52.20 50.74 50.28 48.12 47.95 47.78 47.61 47.44 47.27 47.10
26.08
20.29
CF3
O
N
N
H
N
N
H
N
N
N
220 210 200 190 180 170 160 150 140 130 120 110 100 90 80 70 60 50 40 30 20 10 0
f1 (ppm)

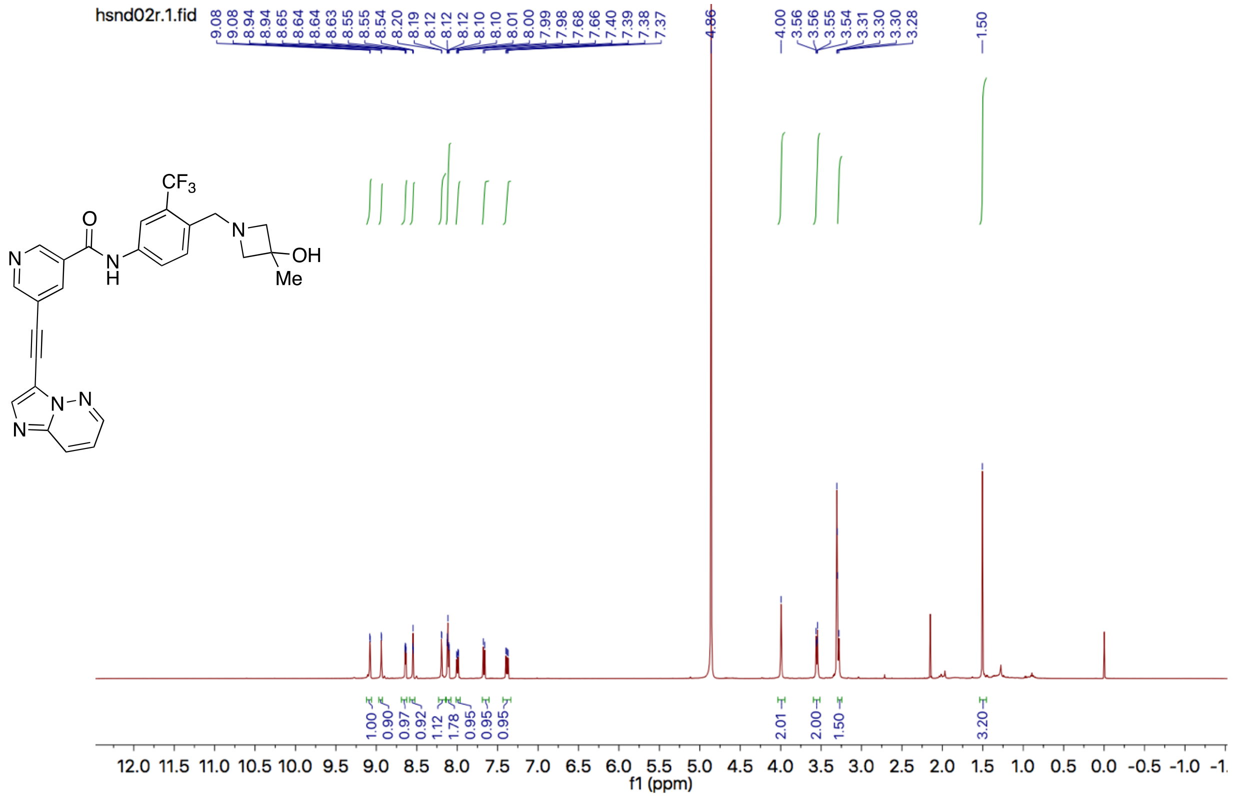
hsnd02r.1.fid
f1 (ppm)

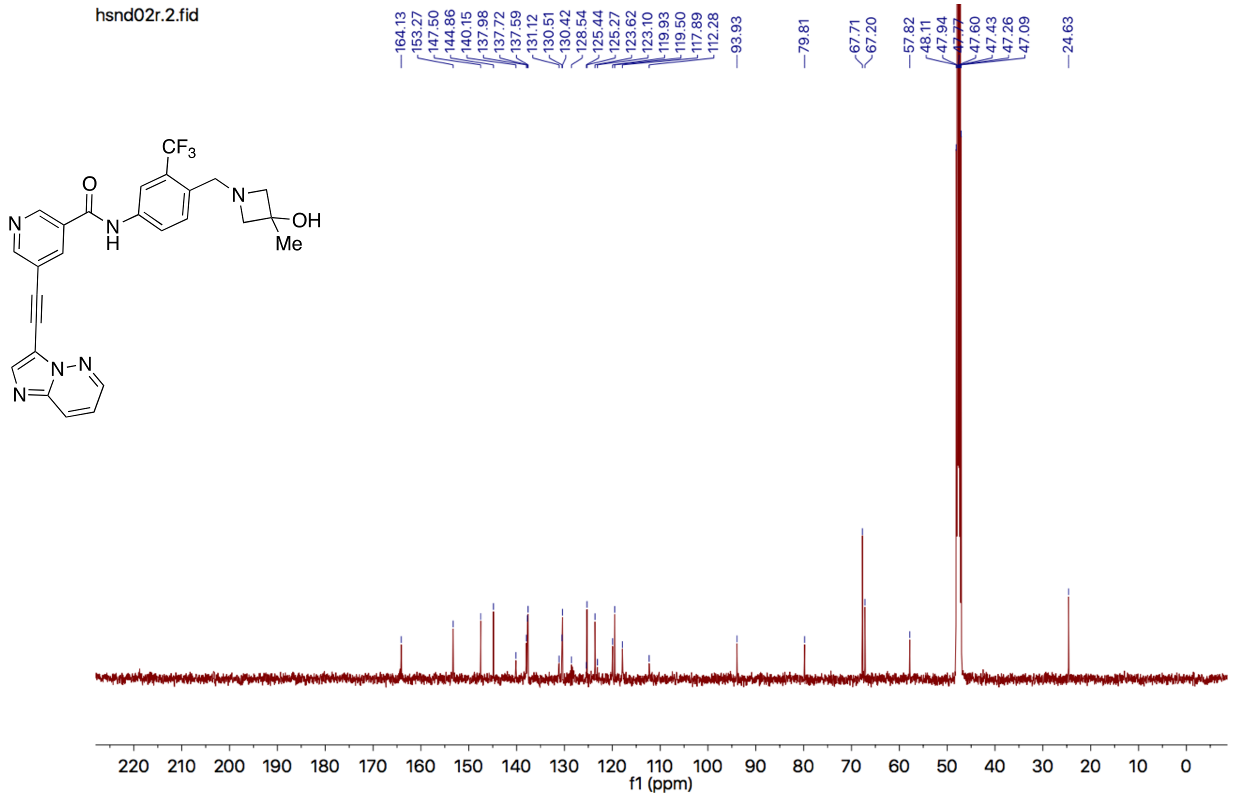
hsnd02r.2.fid
f1 (ppm)

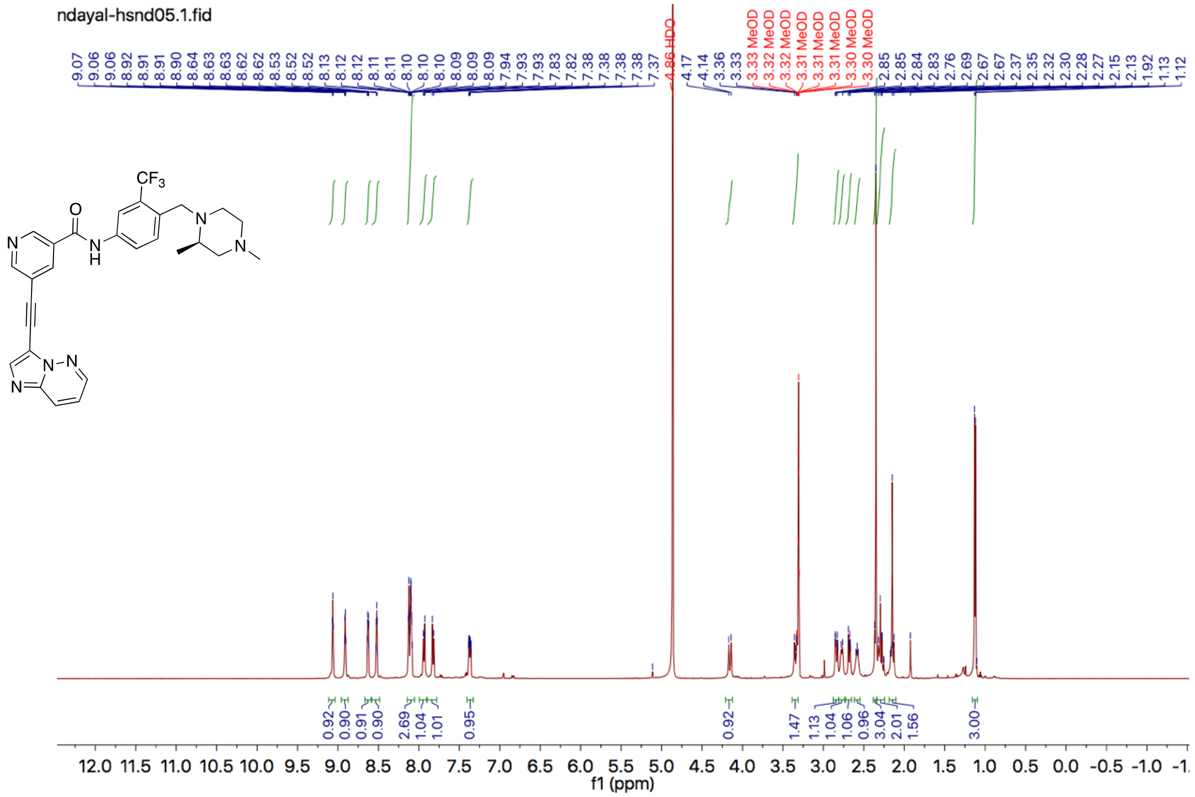
ndayal-hsnd05.1.fid
9.07 9.06 9.06 8.92 8.91 8.91 8.90 8.64 8.63 8.63 8.62 8.62 8.53 8.52 8.52 8.13 8.12 8.12 8.11 8.11 8.10 8.10 8.10 8.09 8.09 7.94 7.93 7.93 7.83 7.82 7.38 7.38 7.38 7.38 7.37 4.86 HDO 4.17 4.14 3.36 3.33 3.33 MeOD 3.32 MeOD 3.32 MeOD 3.31 MeOD 3.31 MeOD 3.31 MeOD 3.30 MeOD 3.30 MeOD 2.85 2.85 2.84 2.83 2.76 2.69 2.67 2.67 2.37 2.35 2.32 2.30 2.28 2.27 2.15 2.13 1.92 1.13 1.12
CF3
O
N
NH
N
N
N
N
N
N
0.92 0.90 0.91 0.90 2.69 1.04 1.01 0.95 0.92 1.47 1.13 1.04 1.06 0.96 3.04 2.01 1.56 3.00
12.0 11.5 11.0 10.5 10.0 9.5 9.0 8.5 8.0 7.5 7.0 6.5 6.0 5.5 5.0 4.5 4.0 3.5 3.0 2.5 2.0 1.5 1.0 0.5 0.0 -0.5 -1.0 -1.
f1 (ppm)

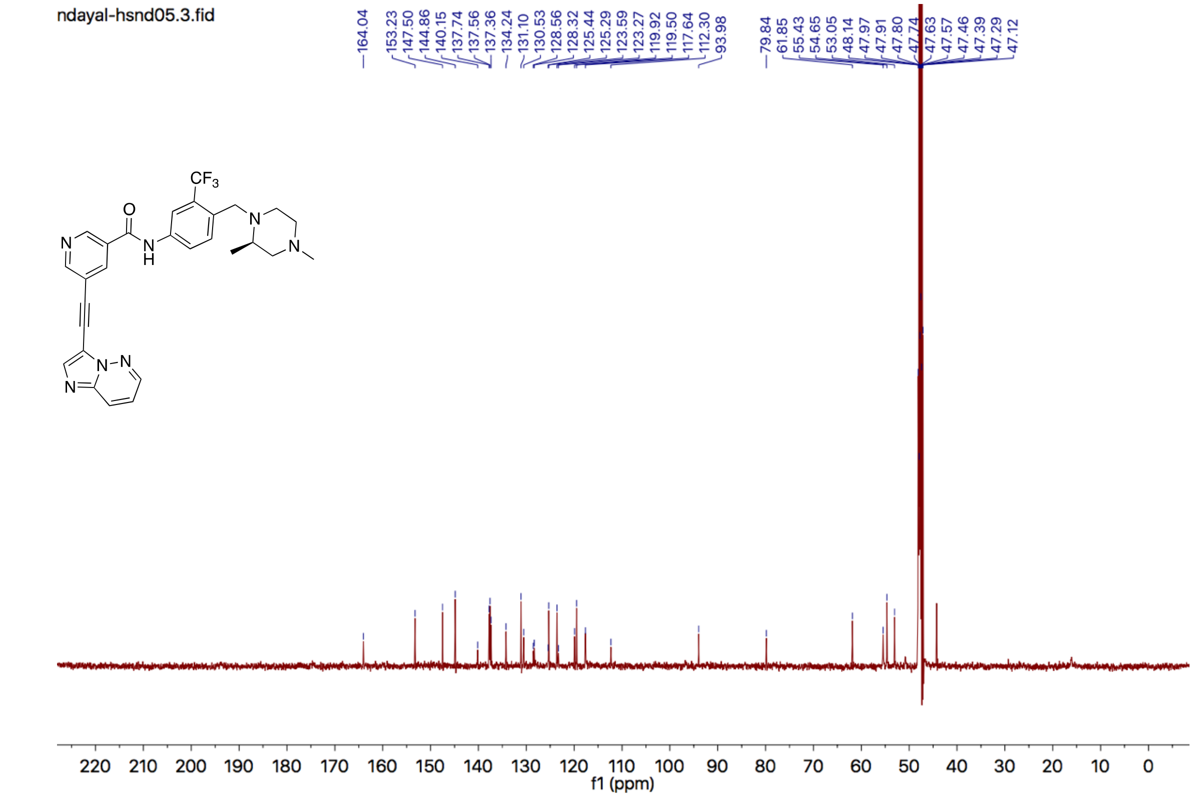
ndayal-hsnd05.3.fid
164.04 153.23 147.50 144.86 140.15 137.74 137.56 137.36 134.24 131.10 130.53 128.56 128.32 125.44 125.29 123.59 123.27 119.92 119.50 117.64 112.30 93.98 79.84 61.85 55.43 54.65 53.05 48.14 47.97 47.91 47.80 47.74 47.63 47.57 47.46 47.39 47.29 47.12
CF3
O
N
NH
N
N
N
N
N
N
220 210 200 190 180 170 160 150 140 130 120 110 100 90 80 70 60 50 40 30 20 10 0
f1 (ppm)

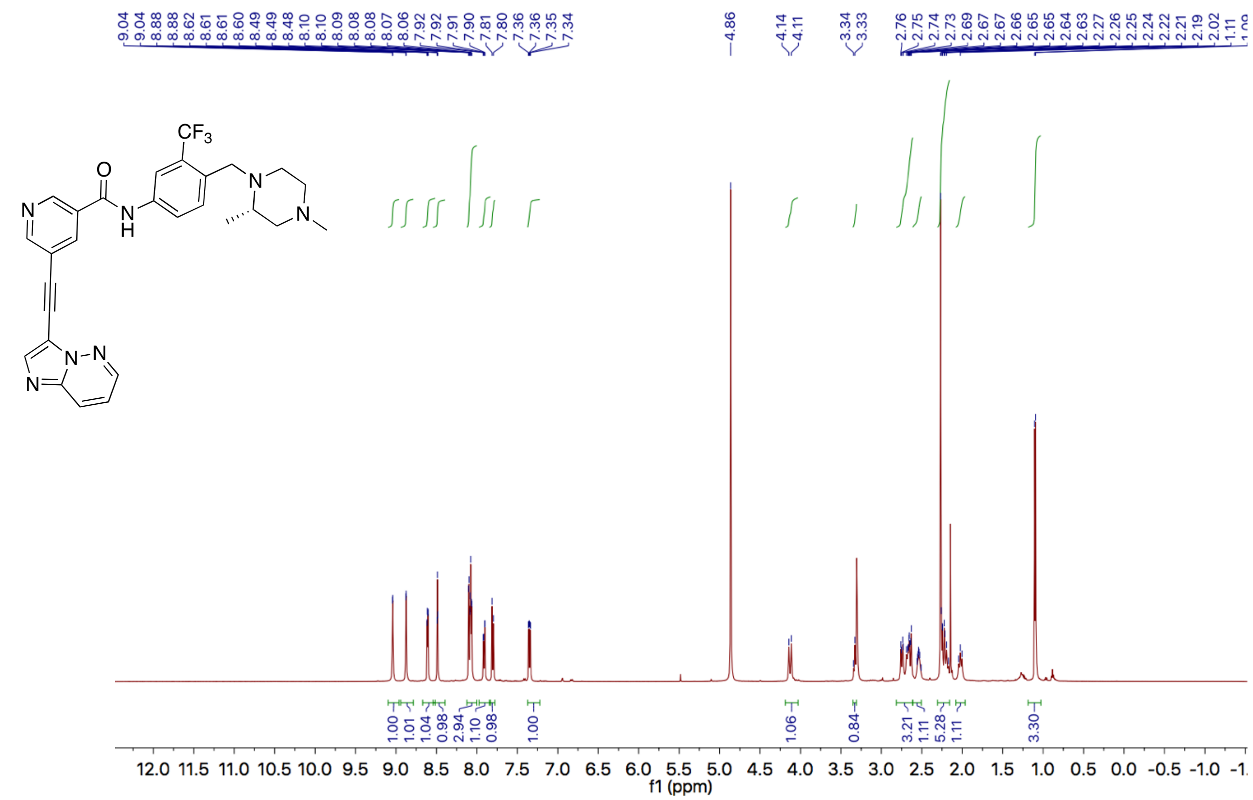
9.04 9.04 8.88 8.88 8.62 8.61 8.61 8.60 8.49 8.49 8.48 8.10 8.10 8.09 8.08 8.08 8.07 8.06 7.92 7.92 7.91 7.90 7.81 7.80 7.36 7.36 7.35 7.34
4.86
4.14 4.11
3.34 3.33
2.76 2.75 2.74 2.73 2.69 2.67 2.67 2.66 2.65 2.65 2.64 2.63 2.27 2.26 2.25 2.24 2.22 2.21 2.19 2.02 1.11
CF_3
O
N
N
H
N
N
N
N
N
1.00 1.01 1.04 0.98 2.94 1.10 0.98 1.00 1.06 0.84 3.21 1.11 5.28 1.11 3.30
12.0 11.5 11.0 10.5 10.0 9.5 9.0 8.5 8.0 7.5 7.0 6.5 6.0 5.5 5.0 4.5 4.0 3.5 3.0 2.5 2.0 1.5 1.0 0.5 0.0 -0.5 -1.0
f1 (ppm)

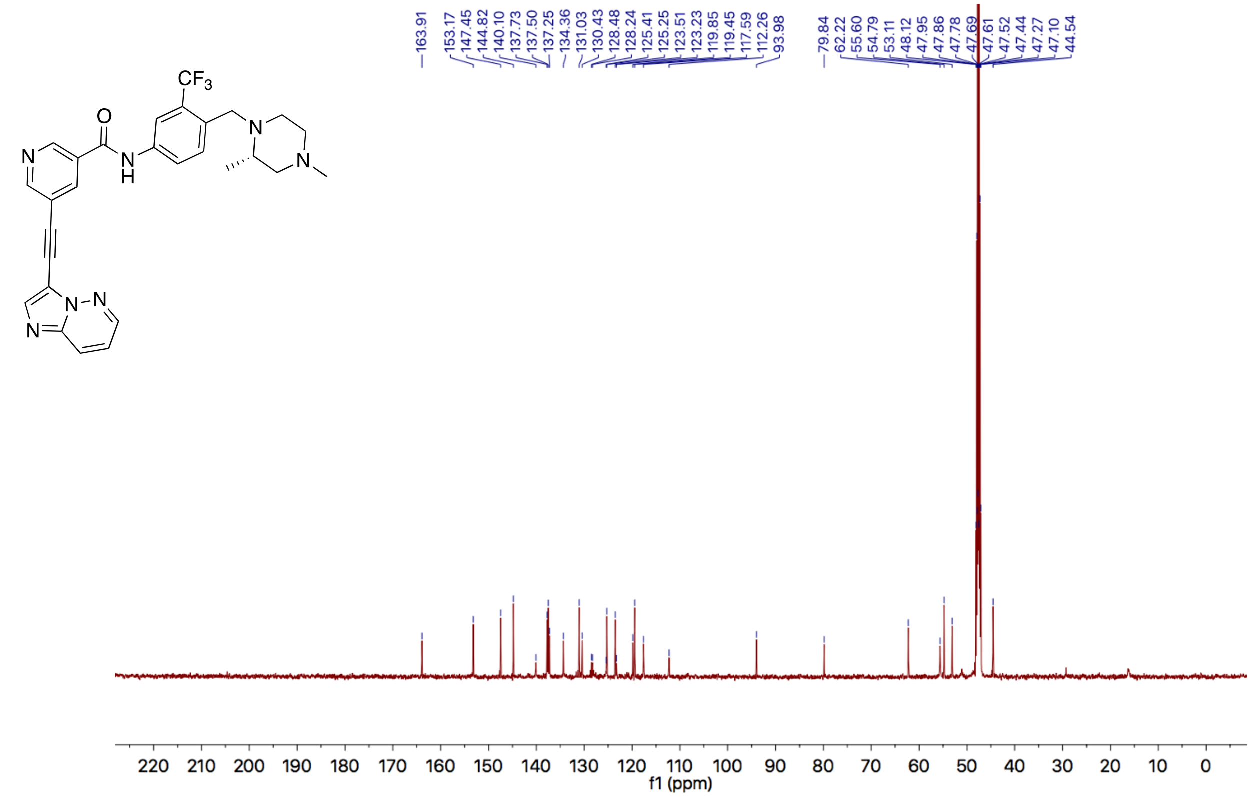
163.91
153.17 147.45 144.82 140.10 137.73 137.50 137.25 134.36 131.03 130.43 128.48 128.24 125.41 125.25 123.51 123.23 119.85 119.45 117.59 112.26 93.98
79.84 62.22 55.60 54.79 53.11 48.12 47.95 47.86 47.78 47.69 47.61 47.52 47.44 47.27 47.10 44.54
CF_3
O
N
N
H
N
N
N
N
N
220 210 200 190 180 170 160 150 140 130 120 110 100 90 80 70 60 50 40 30 20 10 0
f1 (ppm)

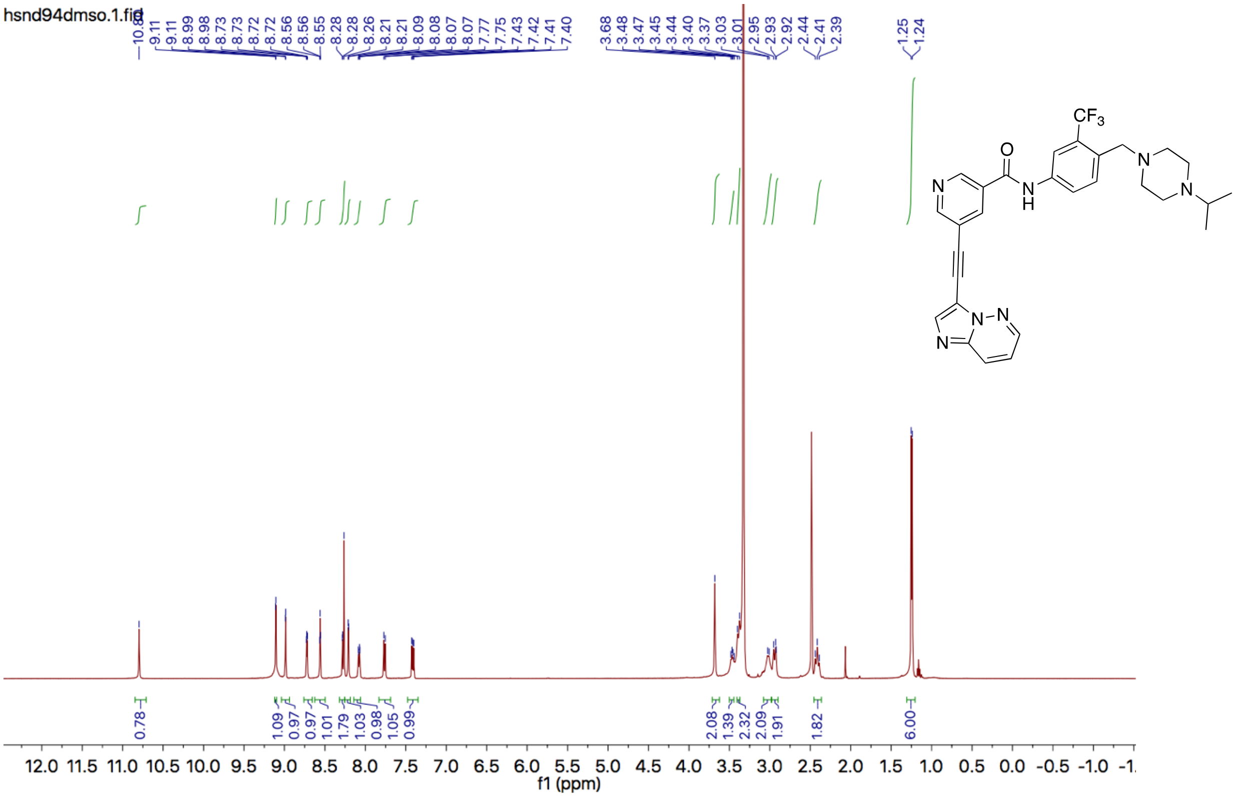
hsnd94dmso.1.fid
10.80 9.11 9.11 8.99 8.98 8.73 8.73 8.72 8.72 8.56 8.56 8.55 8.28 8.28 8.26 8.21 8.21 8.09 8.08 8.07 8.07 7.77 7.75 7.43 7.42 7.41 7.40
3.68 3.48 3.47 3.45 3.44 3.40 3.37 3.03 3.01 2.95 2.93 2.92 2.44 2.41 2.39
1.25 1.24
0.78 1.09 0.97 0.97 1.01 1.79 1.03 0.98 1.05 0.99
2.08 1.39 2.32 2.09 1.91 1.82 6.00
12.0 11.5 11.0 10.5 10.0 9.5 9.0 8.5 8.0 7.5 7.0 6.5 6.0 5.5 5.0 4.5 4.0 3.5 3.0 2.5 2.0 1.5 1.0 0.5 0.0 -0.5 -1.0
f1 (ppm)
CF3
O
N
H
N
N
N
N
N
N

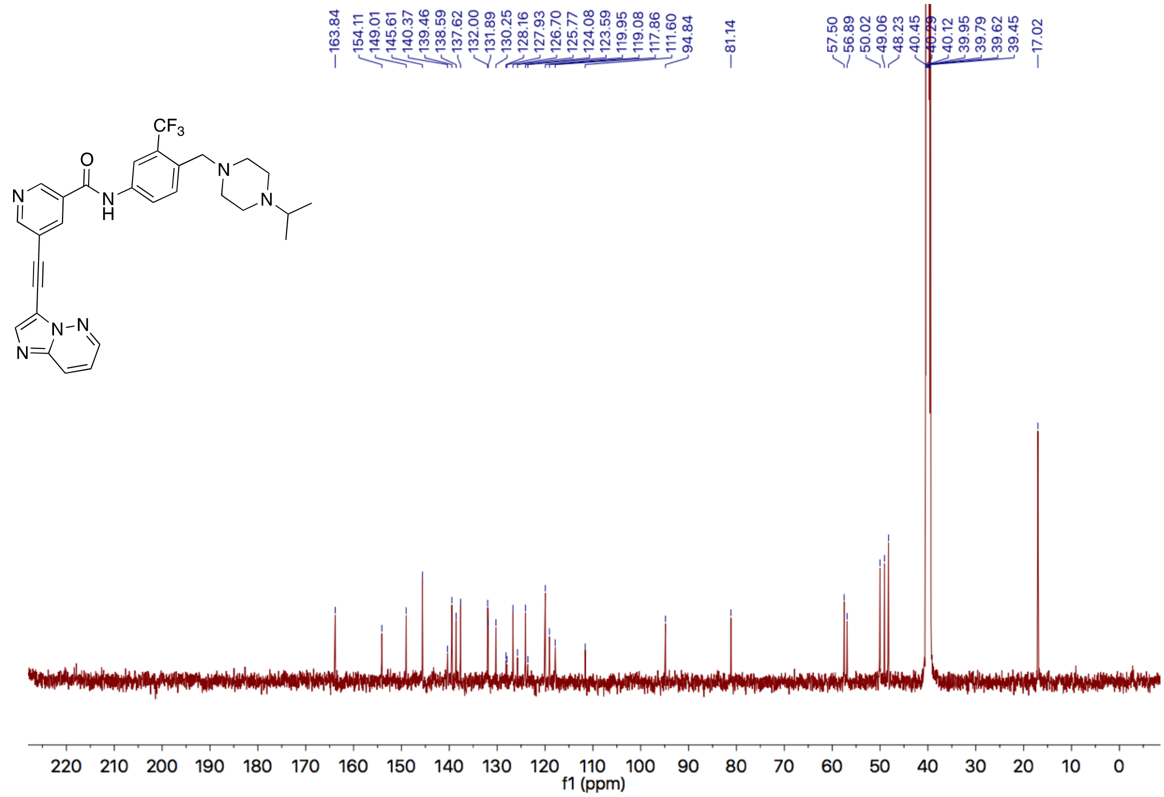
163.84 154.11 149.01 145.61 140.37 139.46 138.59 137.62 132.00 131.89 130.25 128.16 127.93 126.70 125.77 124.08 123.59 119.95 119.08 117.86 111.60 94.84 81.14
57.50 56.89 50.02 49.06 48.23 40.45 40.29 40.12 39.95 39.79 39.62 39.45 17.02
CF3
O
N
H
N
N
N
N
N
N
220 210 200 190 180 170 160 150 140 130 120 110 100 90 80 70 60 50 40 30 20 10 0
f1 (ppm)

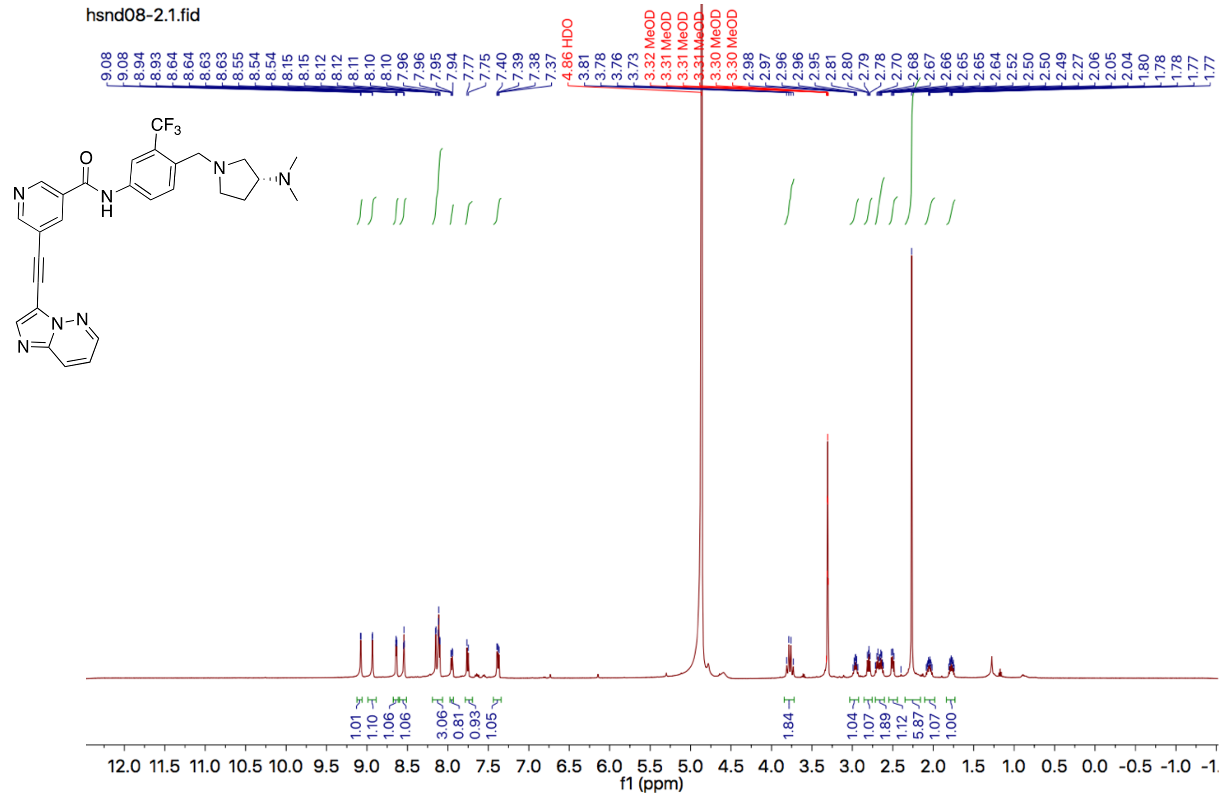
hsnd08-2.1.fid
9.08 9.08 8.94 8.93 8.64 8.64 8.63 8.63 8.55 8.54 8.54 8.15 8.15 8.12 8.12 8.11 8.10 8.10 7.96 7.95 7.94 7.77 7.75 7.40 7.39 7.38 7.37
4.86 HDO
3.81 3.78 3.76 3.73
3.32 MeOD 3.31 MeOD 3.31 MeOD 3.30 MeOD 3.30 MeOD
2.98 2.97 2.96 2.96 2.95 2.81 2.80 2.79 2.78 2.70 2.68 2.67 2.66 2.65 2.65 2.64 2.52 2.50 2.50 2.49 2.27 2.06 2.05 2.04 1.80 1.78 1.78 1.77 1.77
CF3
O
N
H
N
N
N
N
N
N
1.01 1.10 1.06 1.06 3.06 0.81 0.93 1.05 1.84 1.04 1.07 1.89 1.12 5.87 1.07 1.00
12.0 11.5 11.0 10.5 10.0 9.5 9.0 8.5 8.0 7.5 7.0 6.5 6.0 5.5 5.0 4.5 4.0 3.5 3.0 2.5 2.0 1.5 1.0 0.5 0.0 -0.5 -1.0
f1 (ppm)

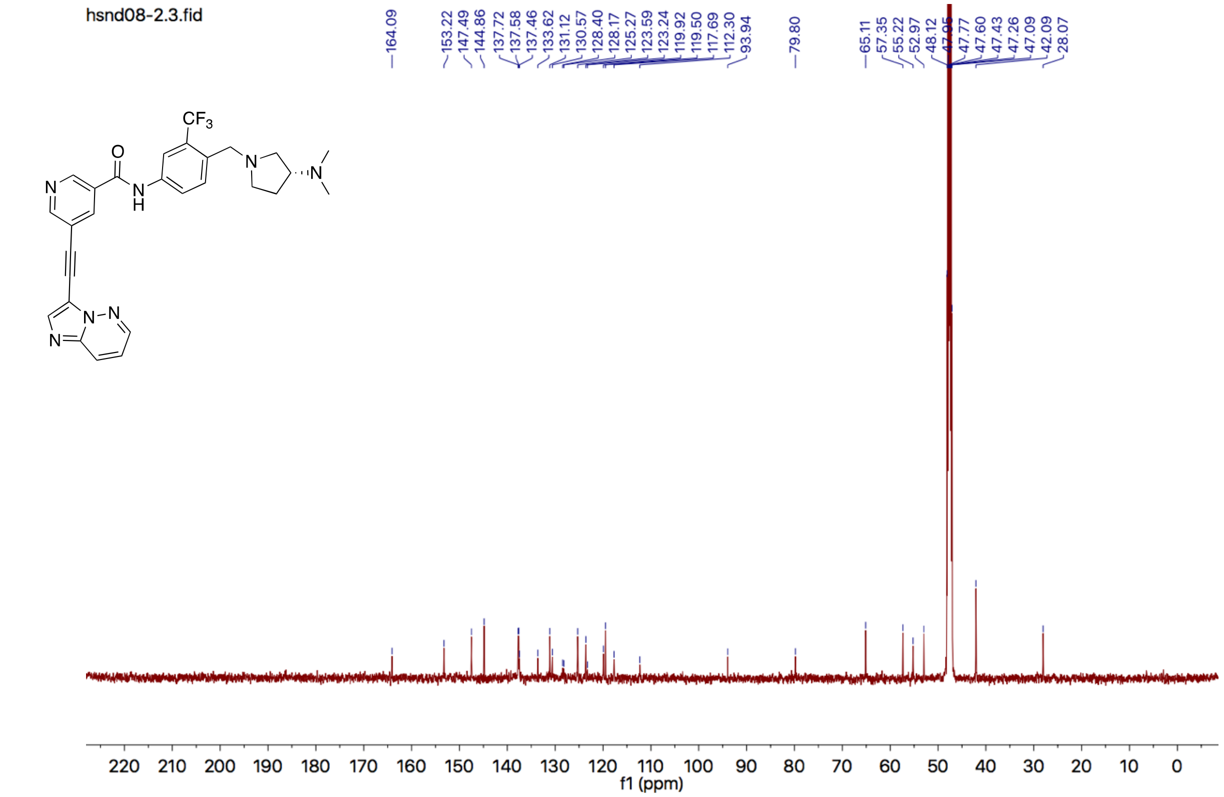
hsnd08-2.3.fid
164.09
153.22 147.49 144.86 137.72 137.58 137.46 133.62 131.12 130.57 128.40 128.17 125.27 123.59 123.24 119.92 119.50 117.69 112.30 93.94
79.80
65.11 57.35 55.22 52.97 48.12 47.95 47.77 47.60 47.43 47.26 47.09 42.09 28.07
CF3
O
N
H
N
N
N
N
N
N
220 210 200 190 180 170 160 150 140 130 120 110 100 90 80 70 60 50 40 30 20 10 0
f1 (ppm)

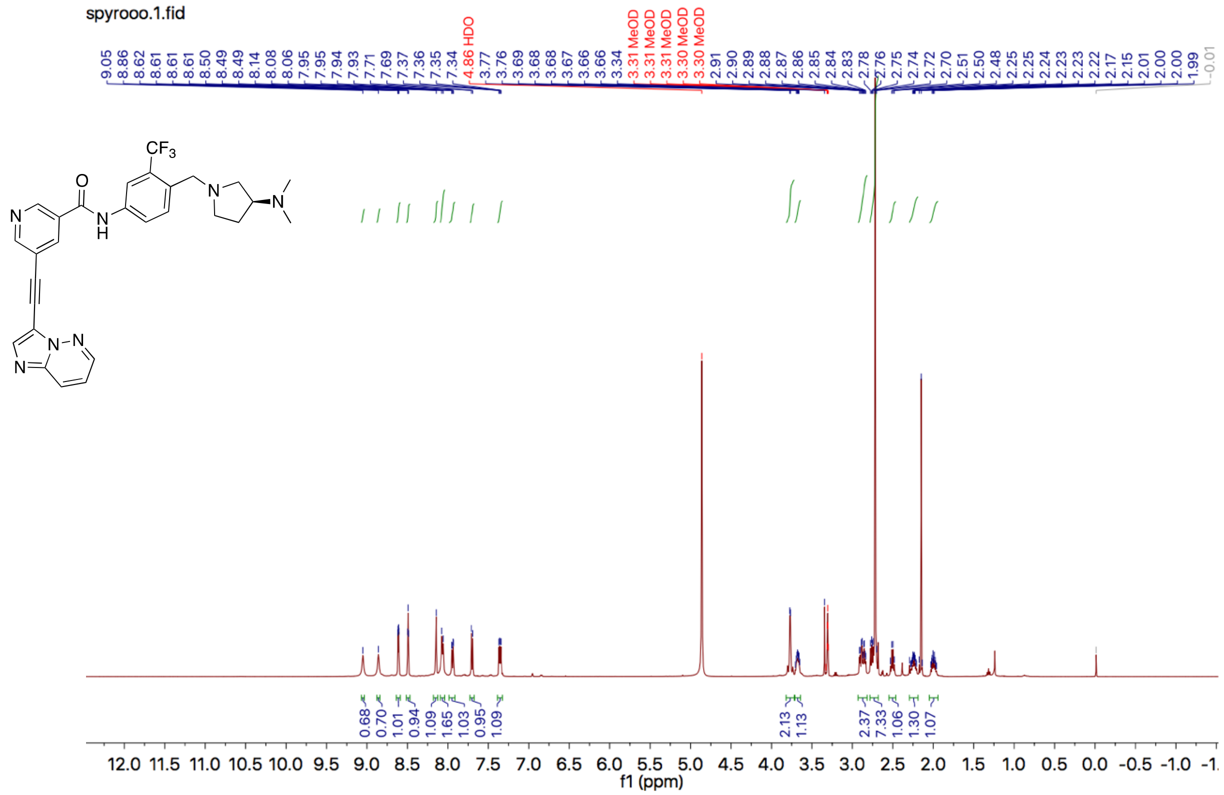
spyrooo.1.fid
9.05 8.86 8.62 8.61 8.61 8.61 8.50 8.49 8.49 8.14 8.08 8.06 7.95 7.95 7.94 7.93 7.71 7.69 7.37 7.36 7.35 7.34
4.86 HDO
3.77 3.76 3.69 3.68 3.67 3.66 3.66 3.34
3.31 MeOD 3.31 MeOD 3.31 MeOD 3.30 MeOD 3.30 MeOD
2.91 2.90 2.89 2.88 2.87 2.86 2.85 2.84 2.83 2.78 2.76 2.75 2.74 2.72 2.70 2.51 2.50 2.48 2.25 2.25 2.24 2.23 2.23 2.22 2.17 2.15 2.01 2.00 1.99
-0.01
CF3
O
N
NH
N
N
N
N
N
0.68 0.70 1.01 0.94 1.09 1.65 1.03 0.95 1.09
2.13 1.13
2.37 7.33 1.06 1.30 1.07
12.0 11.5 11.0 10.5 10.0 9.5 9.0 8.5 8.0 7.5 7.0 6.5 6.0 5.5 5.0 4.5 4.0 3.5 3.0 2.5 2.0 1.5 1.0 0.5 0.0 -0.5 -1.0
f1 (ppm)

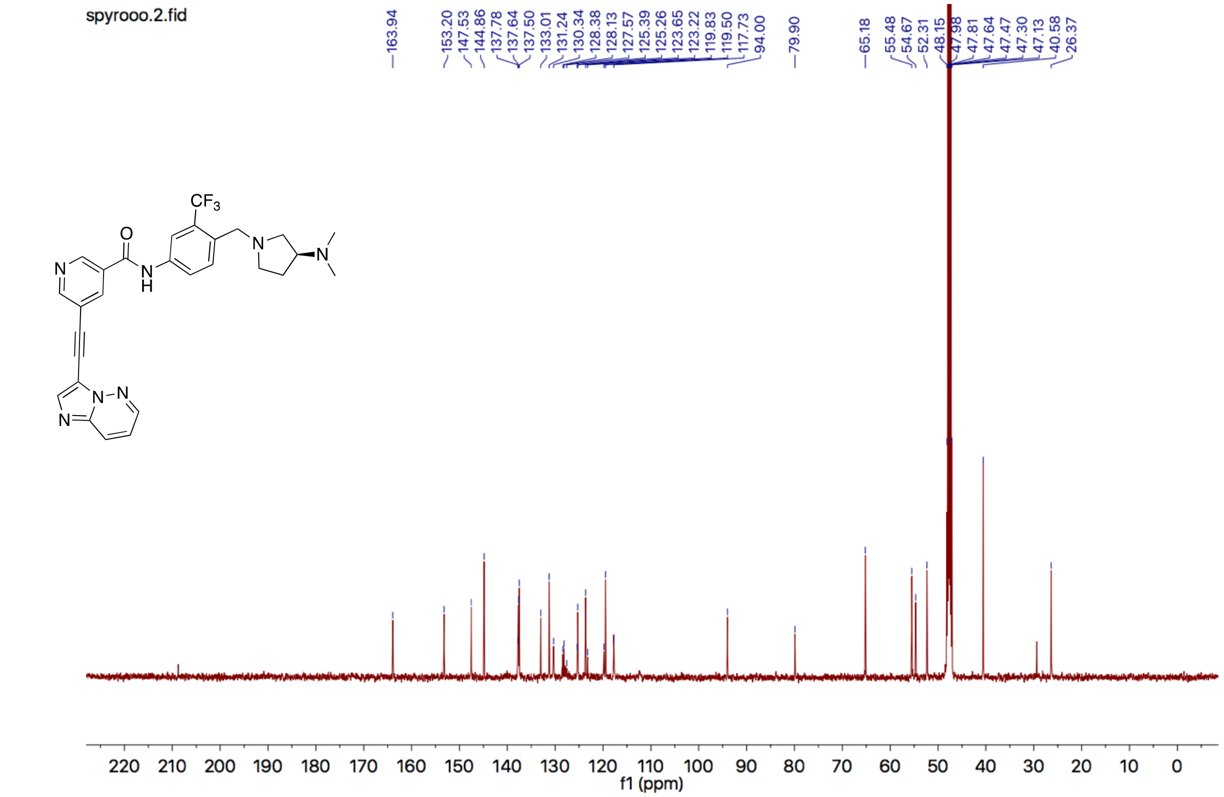
spyrooo.2.fid
163.94
153.20 147.53 144.86 137.78 137.64 137.50 133.01 131.24 130.34 128.38 128.13 127.57 125.39 125.26 123.65 123.22 119.83 119.50 117.73 94.00
79.90
65.18
55.48 54.67 52.31 48.15 47.98 47.81 47.64 47.47 47.30 47.13 40.58 26.37
CF3
O
N
NH
N
N
N
N
N
220 210 200 190 180 170 160 150 140 130 120 110 100 90 80 70 60 50 40 30 20 10 0
f1 (ppm)

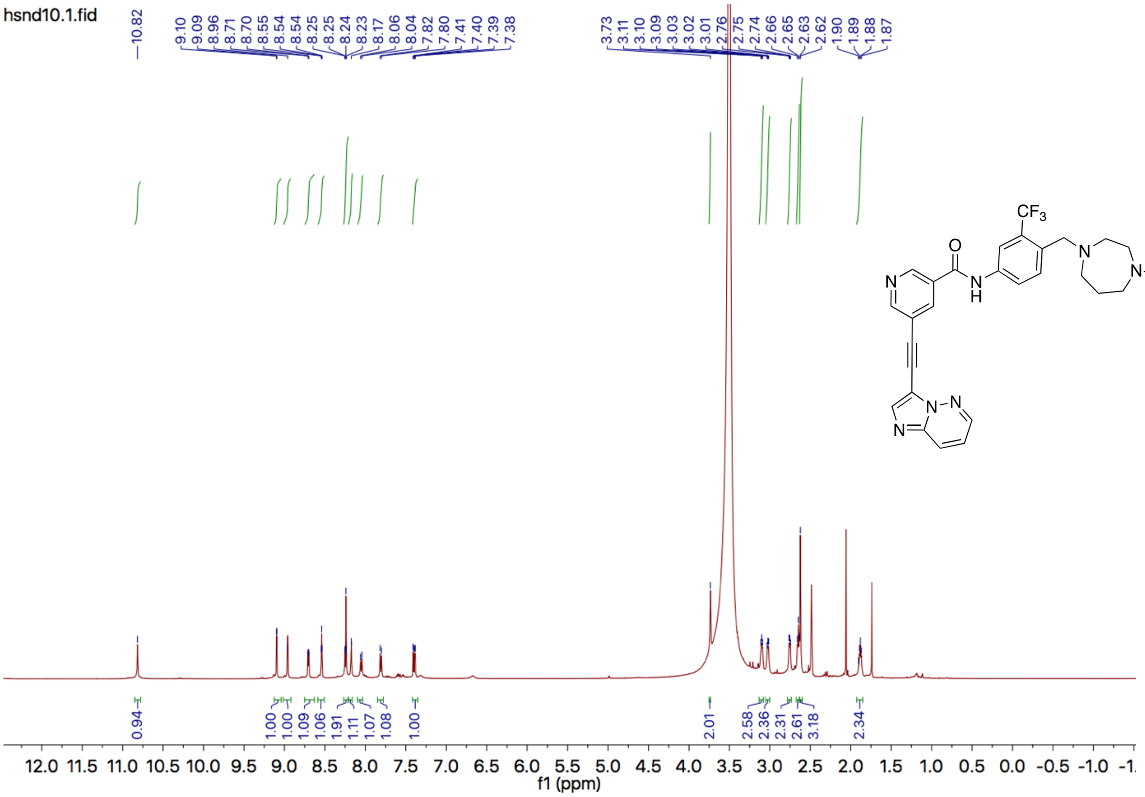
hsnd10.1.fid
10.82
9.10 9.09 8.96 8.71 8.70 8.55 8.54 8.54 8.25 8.24 8.23 8.17 8.06 8.04 7.82 7.80 7.41 7.40 7.39 7.38
3.73 3.11 3.10 3.09 3.03 3.02 3.01 2.76 2.75 2.74 2.66 2.65 2.63 2.62 1.90 1.89 1.88 1.87
CF3
O
N
H
N
N
N
N
N
N
0.94 1.00 1.00 1.09 1.06 1.91 1.11 1.07 1.08 1.00 2.01 2.58 2.36 2.31 2.61 3.18 2.34
f1 (ppm)

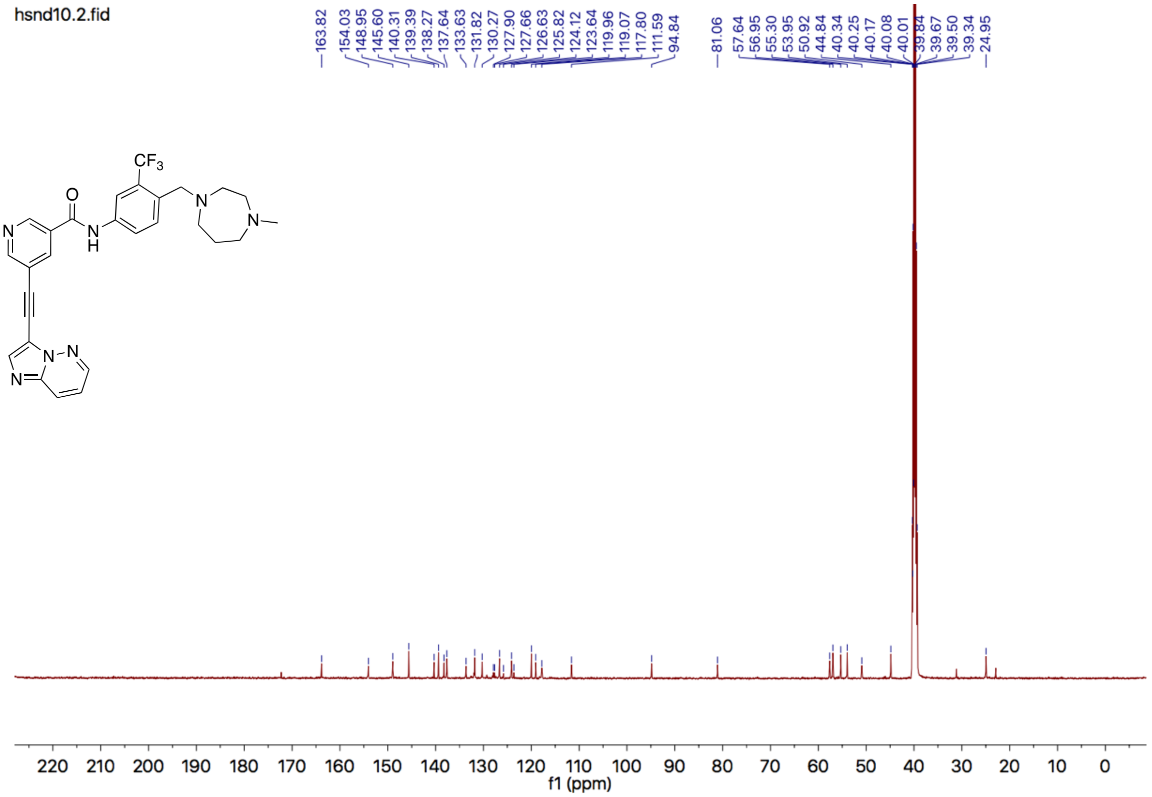
hsnd10.2.fid
163.82 154.03 148.95 145.60 140.31 139.39 138.27 137.64 133.63 131.82 130.27 127.90 127.66 126.63 125.82 124.12 123.64 119.96 119.07 117.80 111.59 94.84
81.06 57.64 56.95 55.30 53.95 50.92 44.84 40.34 40.25 40.17 40.08 40.01 39.84 39.67 39.50 39.34 24.95
CF3
O
N
H
N
N
N
N
N
N
f1 (ppm)

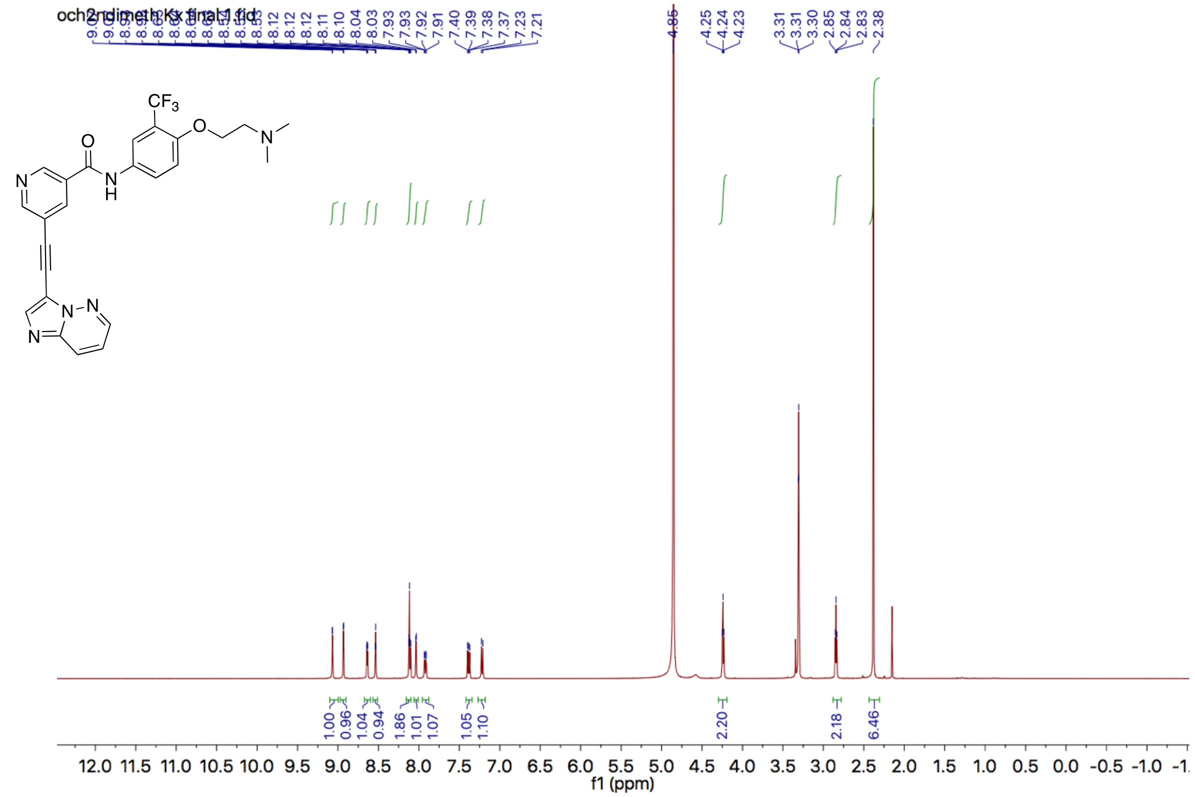
och2ndimeth Kx final.1.fid
8.12
8.12
8.12
8.11
8.10
8.04
8.03
7.93
7.93
7.92
7.91
7.40
7.39
7.38
7.37
7.23
7.21
4.85
4.25
4.24
4.23
3.31
3.31
3.30
2.85
2.84
2.83
2.38
CF3
O
N
N
H
N
O
N
N
N
1.00
0.96
1.04
0.94
1.86
1.01
1.07
1.05
1.10
2.20
2.18
6.46
12.0 11.5 11.0 10.5 10.0 9.5 9.0 8.5 8.0 7.5 7.0 6.5 6.0 5.5 5.0 4.5 4.0 3.5 3.0 2.5 2.0 1.5 1.0 0.5 0.0 -0.5 -1.0
f1 (ppm)

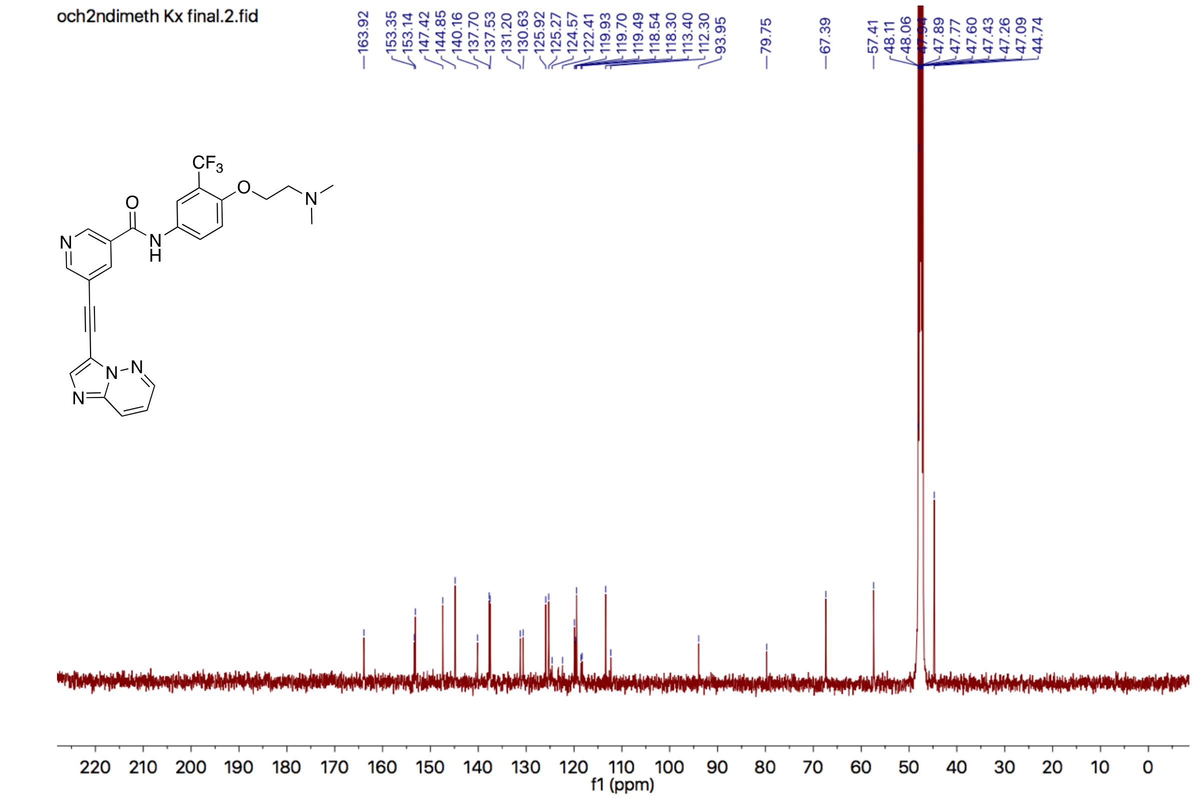
och2ndimeth Kx final.2.fid
163.92
153.35
153.14
147.42
144.85
140.16
137.70
137.53
131.20
130.63
125.92
125.27
124.57
122.41
119.93
119.70
119.49
118.54
118.30
113.40
112.30
93.95
79.75
67.39
57.41
48.11
48.06
47.89
47.77
47.60
47.43
47.26
47.09
44.74
CF3
O
N
N
H
N
O
N
N
N
220 210 200 190 180 170 160 150 140 130 120 110 100 90 80 70 60 50 40 30 20 10 0
f1 (ppm)

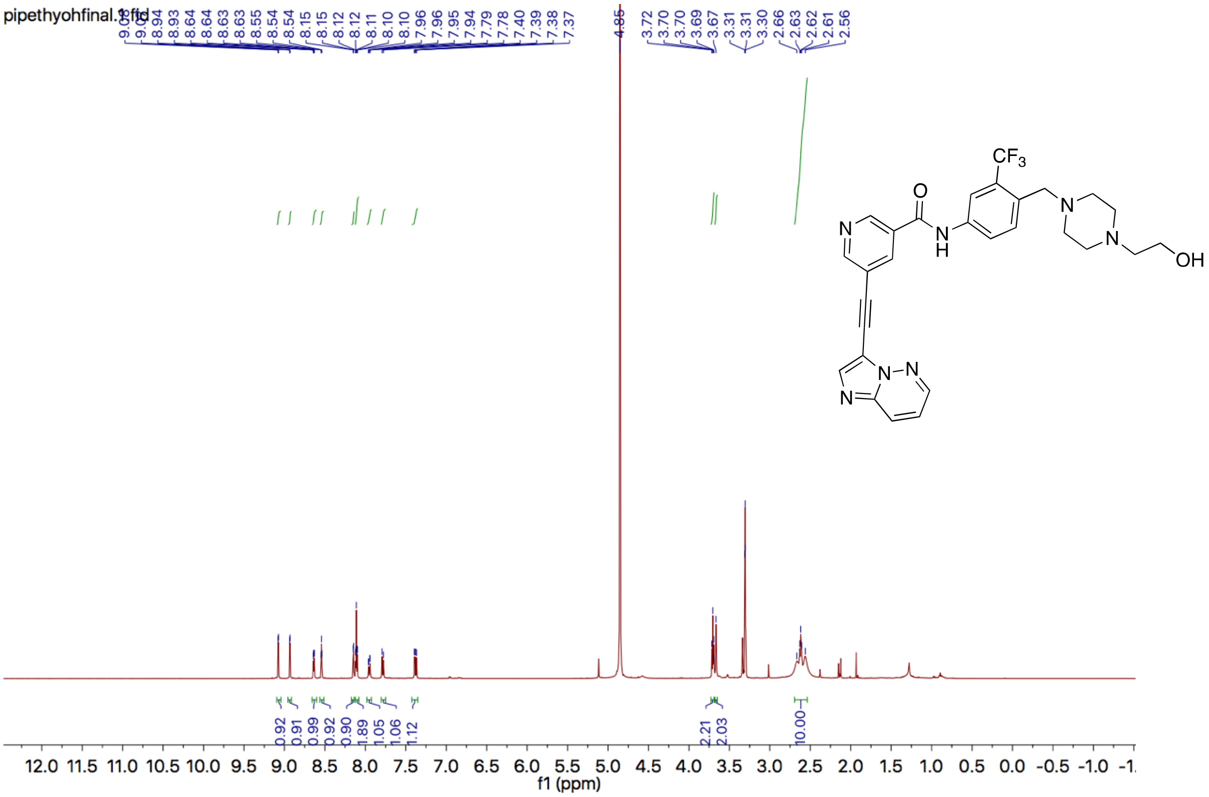
pipethyohfinal.1.fid
9.05 9.04 8.94 8.93 8.64 8.64 8.63 8.55 8.54 8.54 8.15 8.15 8.12 8.12 8.11 8.10 8.10 7.96 7.96 7.95 7.94 7.79 7.78 7.40 7.39 7.38 7.37
4.85
3.72 3.70 3.70 3.69 3.67 3.31 3.31 3.30 2.66 2.63 2.62 2.61 2.56
0.92 0.91 0.99 0.92 0.90 1.89 1.05 1.06 1.12
2.21 2.03
10.00
CF3
O
N
H
OH
f1 (ppm)

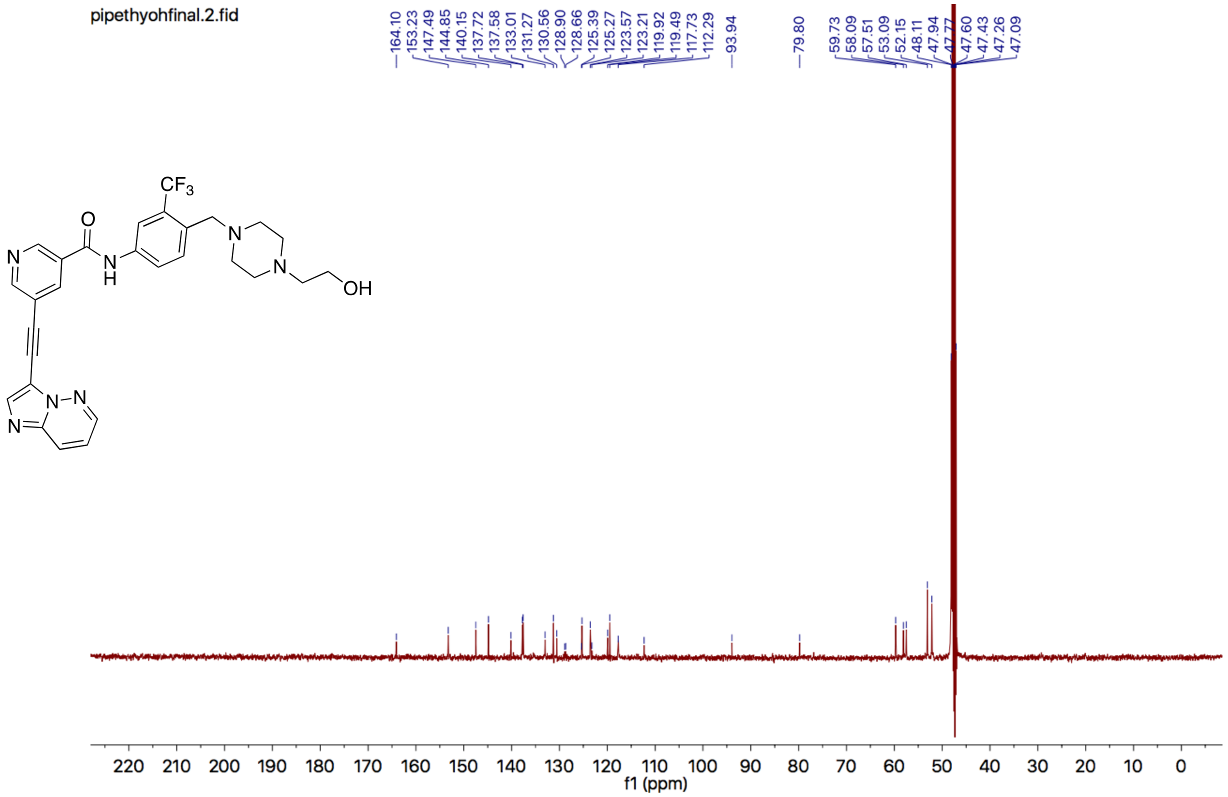
pipethyohfinal.2.fid
164.10 153.23 147.49 144.85 140.15 137.72 137.58 133.01 131.27 130.56 128.90 128.66 125.39 125.27 123.57 123.21 119.92 119.49 117.73 112.29 93.94
79.80
59.73 58.09 57.51 53.09 52.15 48.11 47.94 47.77 47.60 47.43 47.26 47.09
CF3
O
N
H
OH
f1 (ppm)

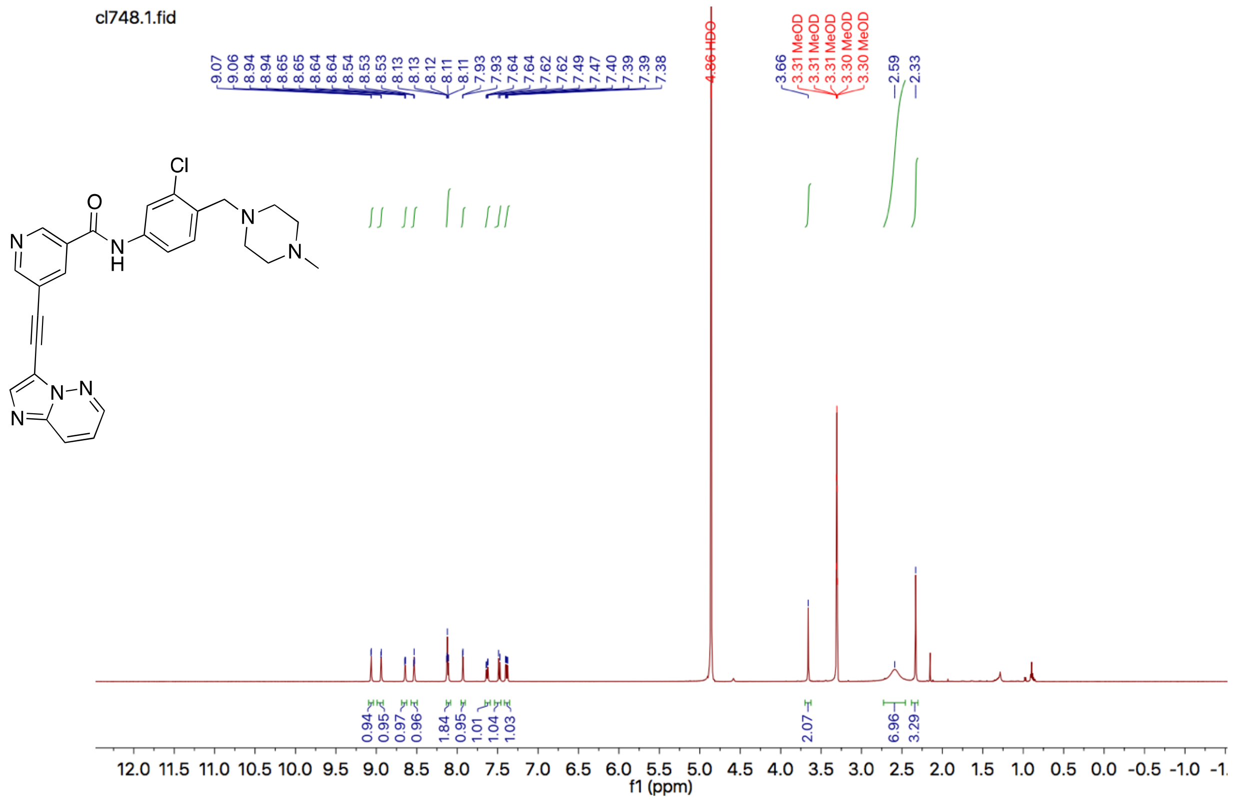
cl748.1.fid
9.07 9.06 8.94 8.94 8.65 8.65 8.64 8.64 8.54 8.53 8.53 8.13 8.13 8.12 8.11 8.11 7.93 7.93 7.64 7.64 7.62 7.62 7.49 7.47 7.40 7.39 7.39 7.38
4.86 HDO
3.66 3.31 MeOD 3.31 MeOD 3.31 MeOD 3.30 MeOD 3.30 MeOD
2.59 2.33
0.94 0.95 0.97 0.96 1.84 0.95 1.01 1.04 1.03 2.07 6.96 3.29
f1 (ppm)

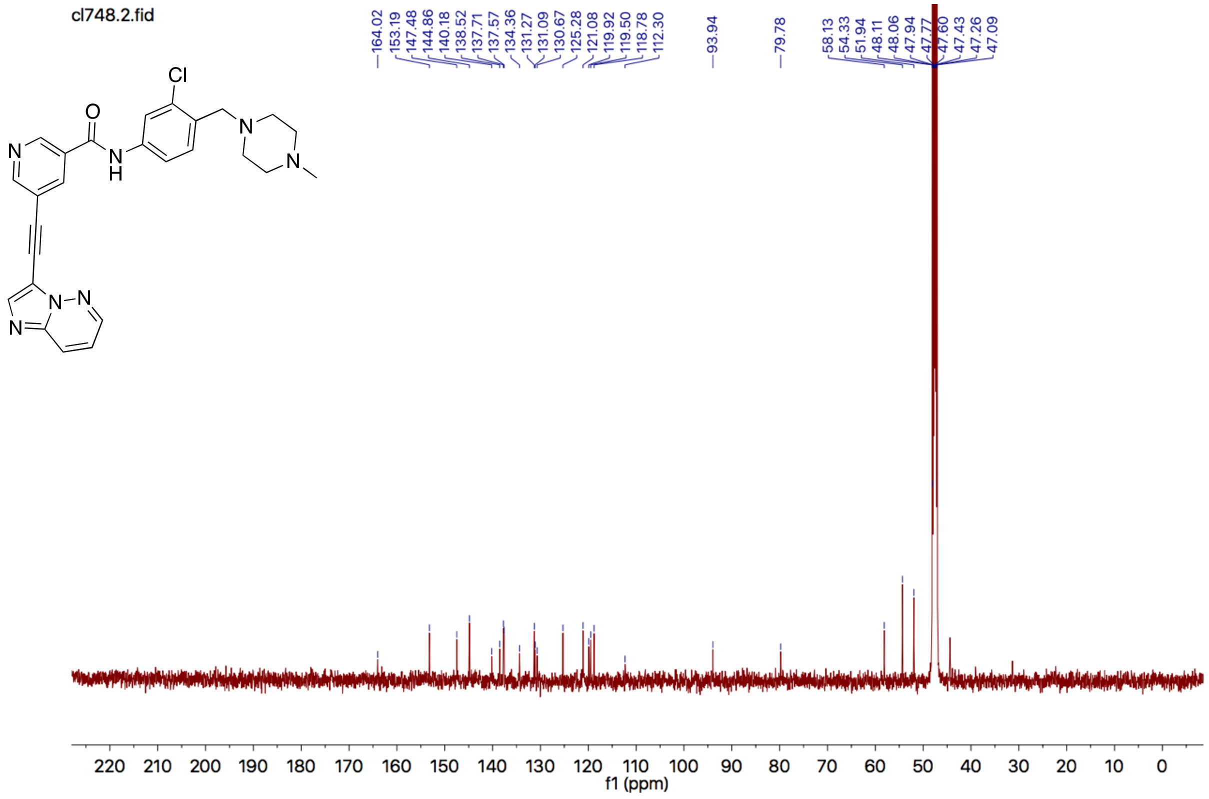
cl748.2.fid
164.02 153.19 147.48 144.86 140.18 138.52 137.71 137.57 134.36 131.27 131.09 130.67 125.28 121.08 119.92 119.50 118.78 112.30
93.94
79.78
58.13 54.33 51.94 48.11 48.06 47.94 47.77 47.60 47.43 47.26 47.09
f1 (ppm)

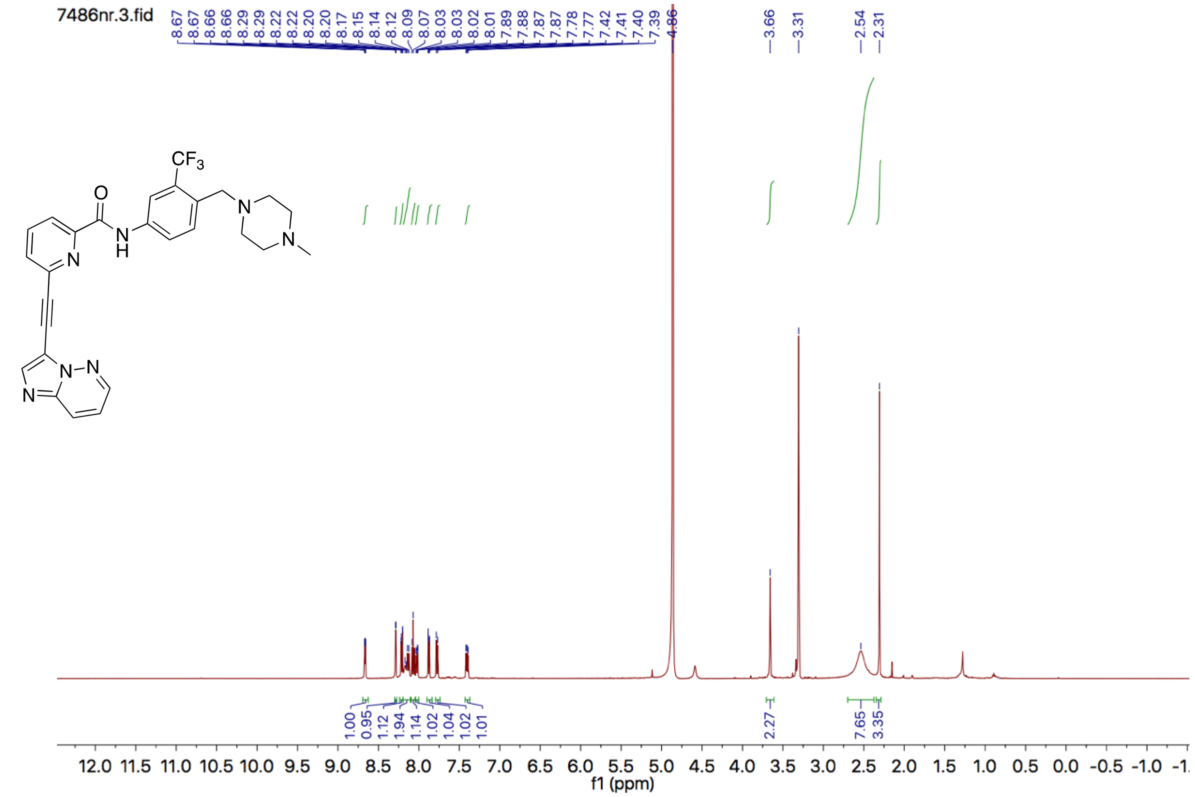
7486nr.3.fid
8.67 8.67 8.66 8.66 8.29 8.29 8.22 8.22 8.20 8.20 8.17 8.15 8.14 8.12 8.09 8.07 8.03 8.02 8.01 7.89 7.88 7.87 7.87 7.78 7.77 7.42 7.41 7.40 7.39 4.86
3.66
3.31
2.54
2.31
1.00 0.95 1.12 1.94 1.14 1.02 1.04 1.02 1.01
2.27
7.65
3.35
CF3
O
N
H
N
N
N
N
N
N
12.0 11.5 11.0 10.5 10.0 9.5 9.0 8.5 8.0 7.5 7.0 6.5 6.0 5.5 5.0 4.5 4.0 3.5 3.0 2.5 2.0 1.5 1.0 0.5 0.0 -0.5 -1.0
f1 (ppm)

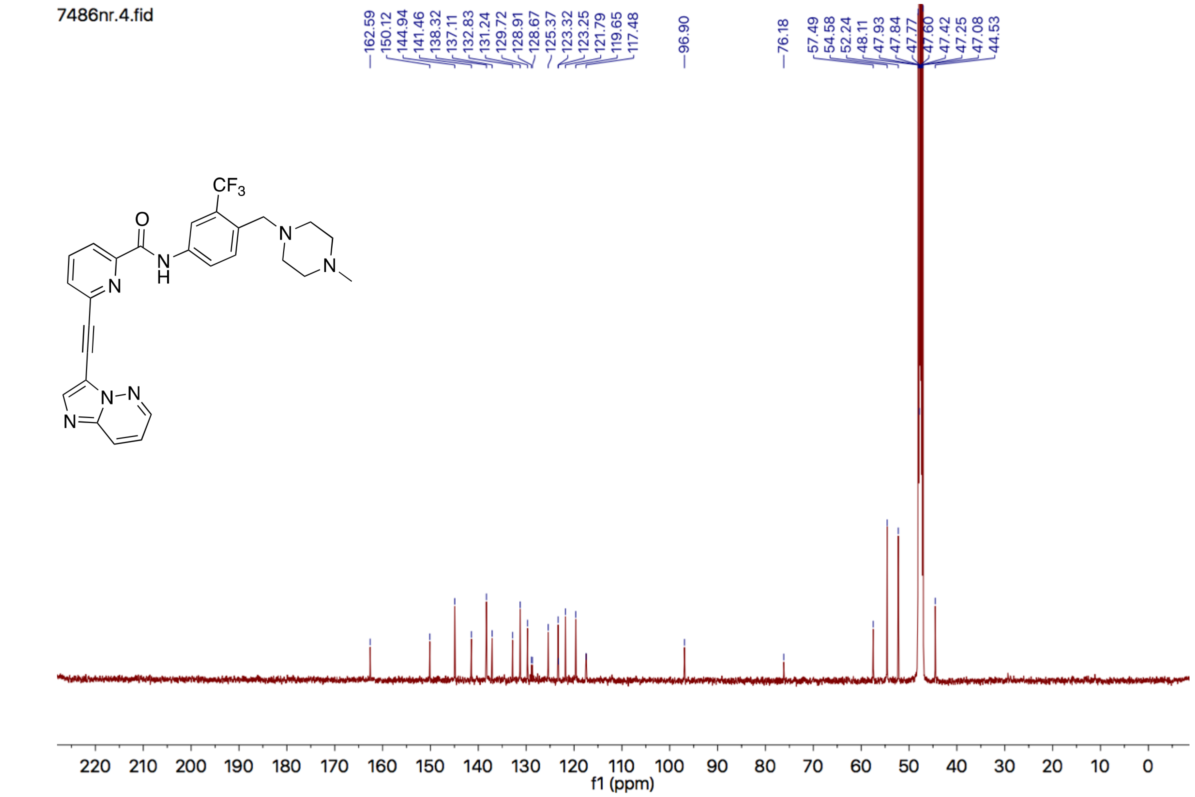
7486nr.4.fid
162.59 150.12 144.94 141.46 138.32 137.11 132.83 131.24 129.72 128.91 128.67 125.37 123.32 123.25 121.79 119.65 117.48
96.90
76.18
57.49 54.58 52.24 48.11 47.93 47.84 47.77 47.60 47.42 47.25 47.08 44.53
CF3
O
N
H
N
N
N
N
N
N
220 210 200 190 180 170 160 150 140 130 120 110 100 90 80 70 60 50 40 30 20 10 0
f1 (ppm)
