# Supplementary material for: Alkynyl nicotinamides show antileukemic activity in drug-resistant acute myeloid leukemia
Source: J Clin Invest. 2024 Jun 17;134(12):e169245. doi: 10.1172/JCI169245 (PMC11178545; doi:10.1172/JCI169245)
Supplement: Unedited blot and gel images [file jci-134-169245-s277.pdf]

Figure 2F

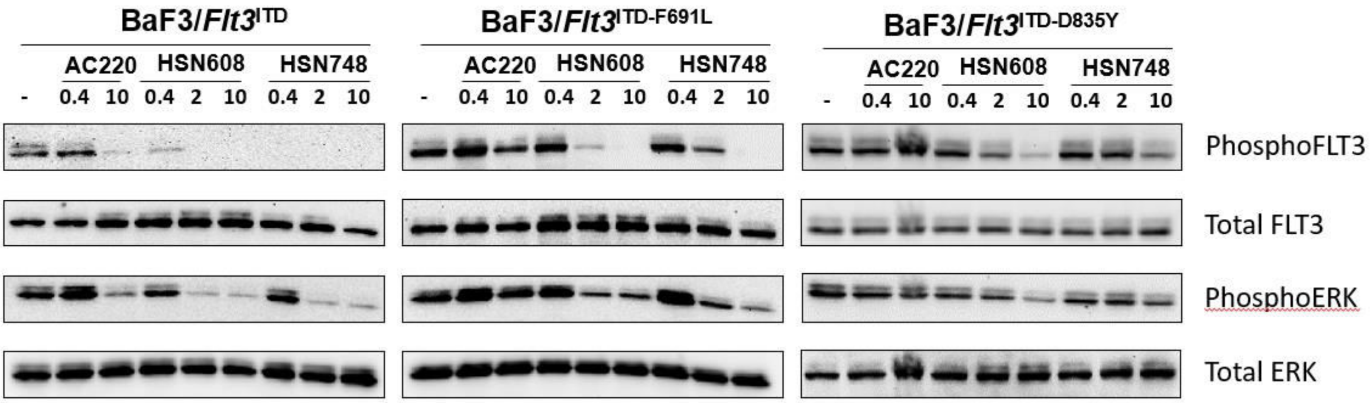
BaF3/Flt3ITD
BaF3/Flt3ITD-F691L
BaF3/Flt3ITD-D835Y
AC220
HSN608
HSN748
- 0.4 10 0.4 2 10 0.4 2 10
PhosphoFLT3
Total FLT3
PhosphoERK
Total ERK

BaF3/*Flt3*ITD

Figure 2F

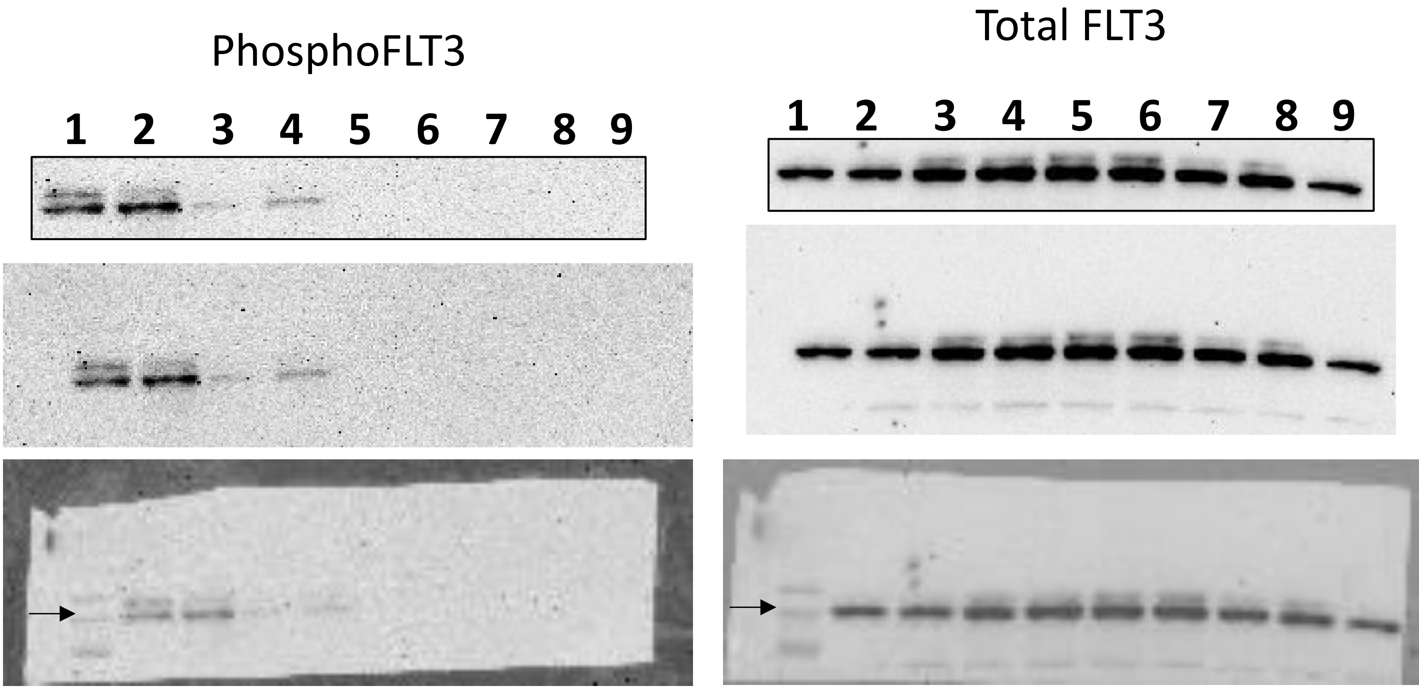

1- BaF3/*Flt3*ITD - 0
2- BaF3/*Flt3*ITD - 0.4nM AC220
3- BaF3/*Flt3*ITD - 10nM AC220
4- BaF3/*Flt3*ITD - 0.4nM HSN608
5- BaF3/*Flt3*ITD - 2nM HSN608
6- BaF3/*Flt3*ITD - 10nM HSN608
7- BaF3/*Flt3*ITD - 0.4nM HSN748
8- BaF3/*Flt3*ITD - 2nM HSN748
9- BaF3/*Flt3*ITD - 10nM HSN748

Figure 2F

BaF3/*Flt3*^{ITD}

PhosphoERK

Total ERK

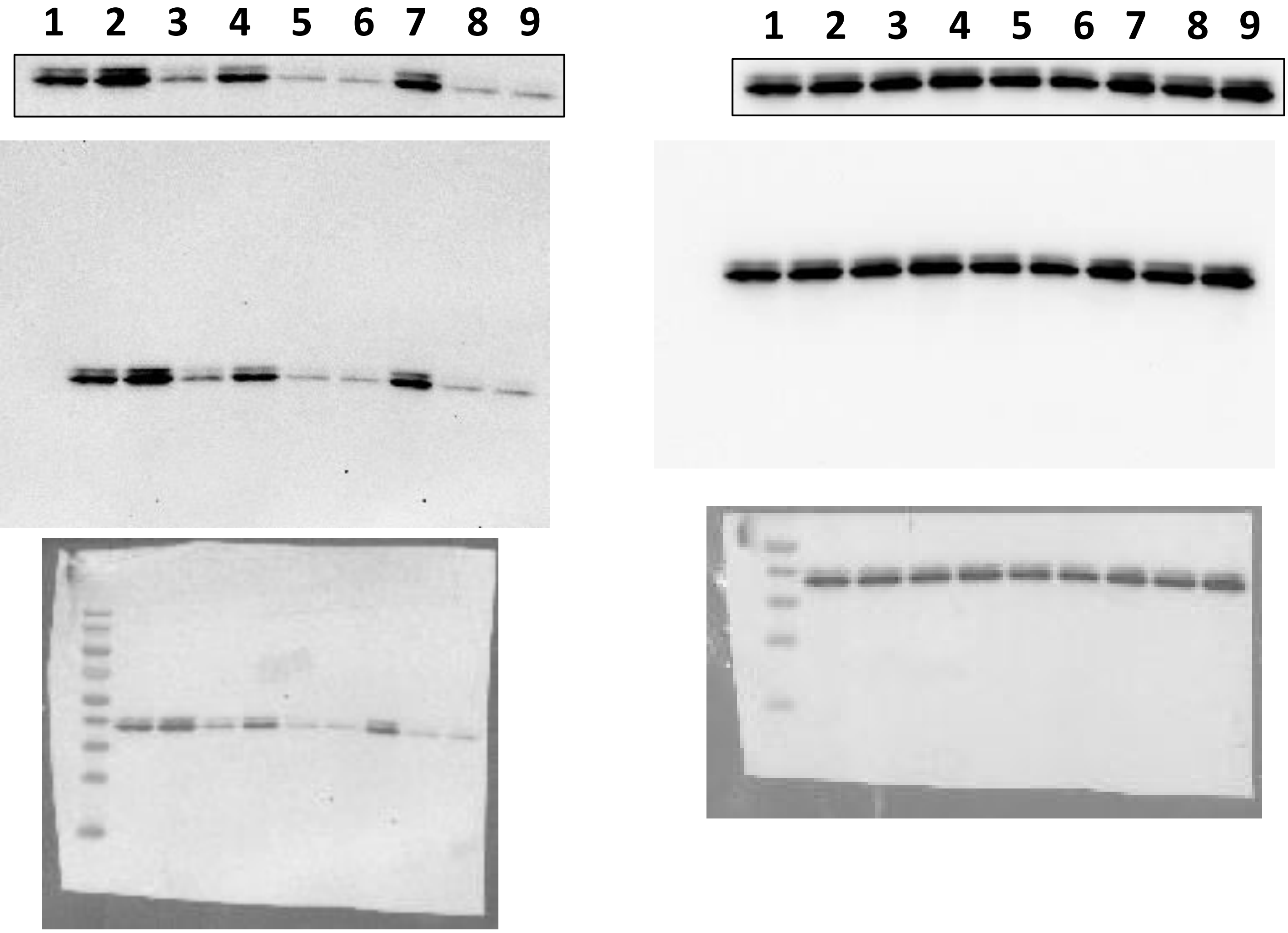

1- BaF3/*Flt3*^{ITD} - 0
2- BaF3/*Flt3*^{ITD} - 0.4nM AC220
3- BaF3/*Flt3*^{ITD} - 10nM AC220
4- BaF3/*Flt3*^{ITD} - 0.4nM HSN608
5- BaF3/*Flt3*^{ITD} - 2nM HSN608
6- BaF3/*Flt3*^{ITD} - 10nM HSN608
7- BaF3/*Flt3*^{ITD} - 0.4nM HSN748
8- BaF3/*Flt3*^{ITD} - 2nM HSN748
9- BaF3/*Flt3*^{ITD} - 10nM HSN748

BaF3/*Flt3*$^{\text{ITD-F691L}}$

Figure 2F

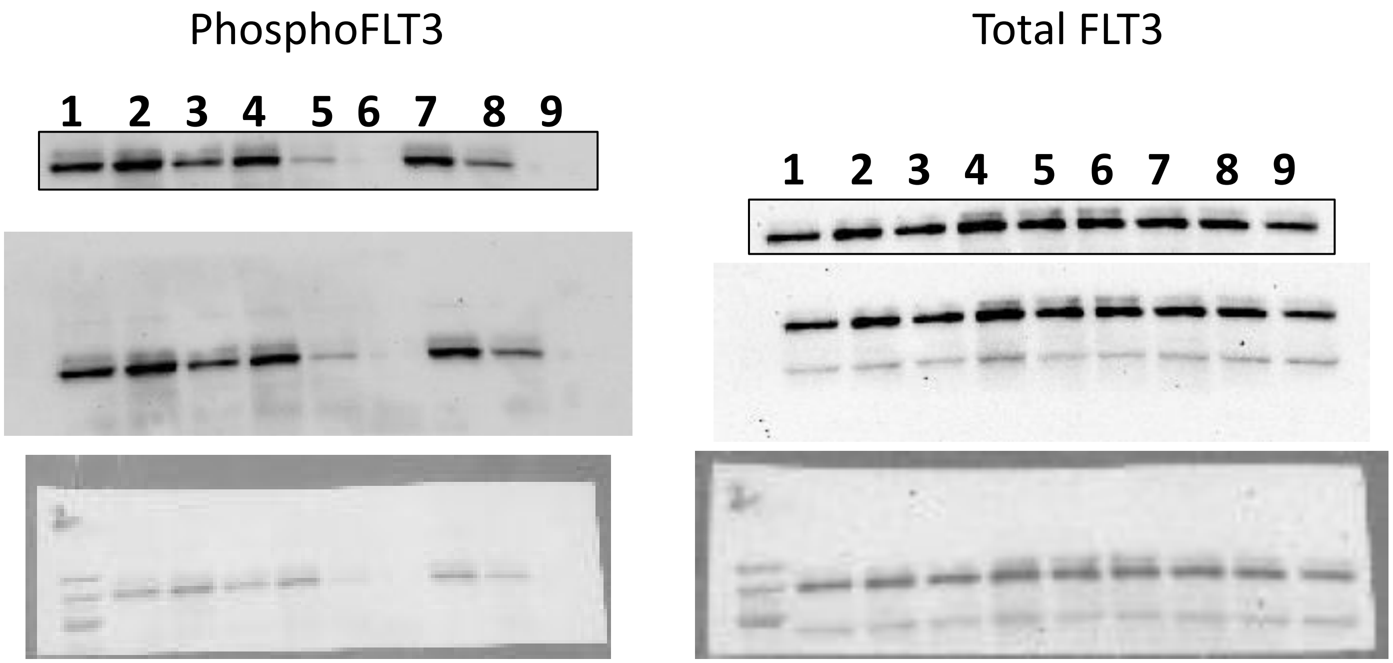

1- BaF3/*Flt3*$^{\text{ITD-F691L}}$ - 0
2- BaF3/*Flt3*$^{\text{ITD-F691L}}$ - 0.4nM AC220
3- BaF3/*Flt3*$^{\text{ITD-F691L}}$ - 10nM AC220
4- BaF3/*Flt3*$^{\text{ITD-F691L}}$ - 0.4nM HSN608
5- BaF3/*Flt3*$^{\text{ITD-F691L}}$ - 2nM HSN608
6- BaF3/*Flt3*$^{\text{ITD-F691L}}$ - 10nM HSN608
7- BaF3/*Flt3*$^{\text{ITD-F691L}}$ - 0.4nM HSN748
8- BaF3/*Flt3*$^{\text{ITD-F691L}}$ - 2nM HSN748
9- BaF3/*Flt3*$^{\text{ITD-F691L}}$ - 10nM HSN748

BaF3/*Flt3*$^{ITD-F691L}$

Figure 2F

PhosphoERK

Total ERK

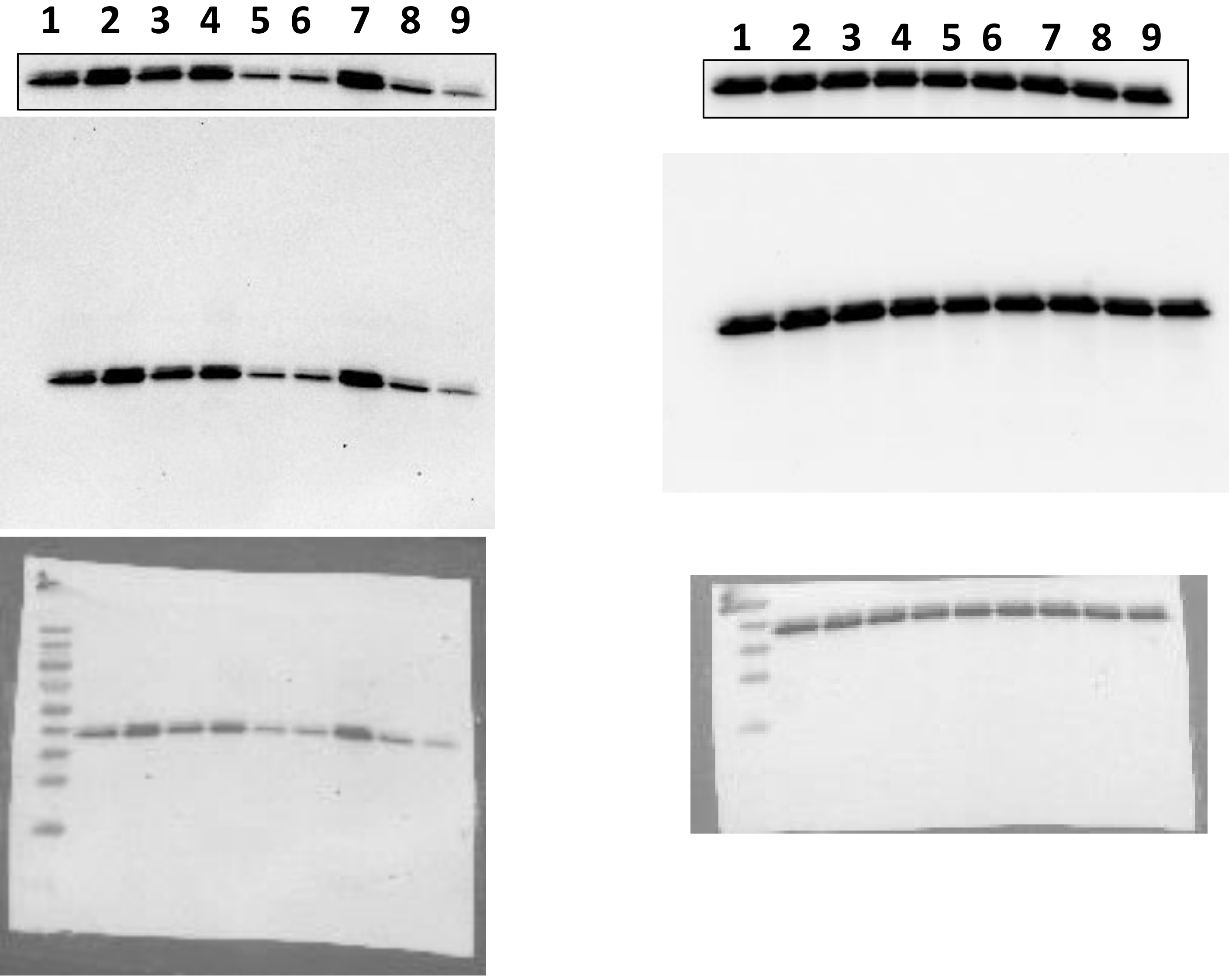

1- BaF3/*Flt3*$^{ITD-F691L}$ - 0
2- BaF3/*Flt3*$^{ITD-F691L}$ - 0.4nM AC220
3- BaF3/*Flt3*$^{ITD-F691L}$ - 10nM AC220
4- BaF3/*Flt3*$^{ITD-F691L}$ - 0.4nM HSN608
5- BaF3/*Flt3*$^{ITD-F691L}$ - 2nM HSN608
6- BaF3/*Flt3*$^{ITD-F691L}$ - 10nM HSN608
7- BaF3/*Flt3*$^{ITD-F691L}$ - 0.4nM HSN748
8- BaF3/*Flt3*$^{ITD-F691L}$ - 2nM HSN748
9- BaF3/*Flt3*$^{ITD-F691L}$ - 10nM HSN748

BaF3/*Flt3*$^{\text{ITD-D835Y}}$

Figure 2F

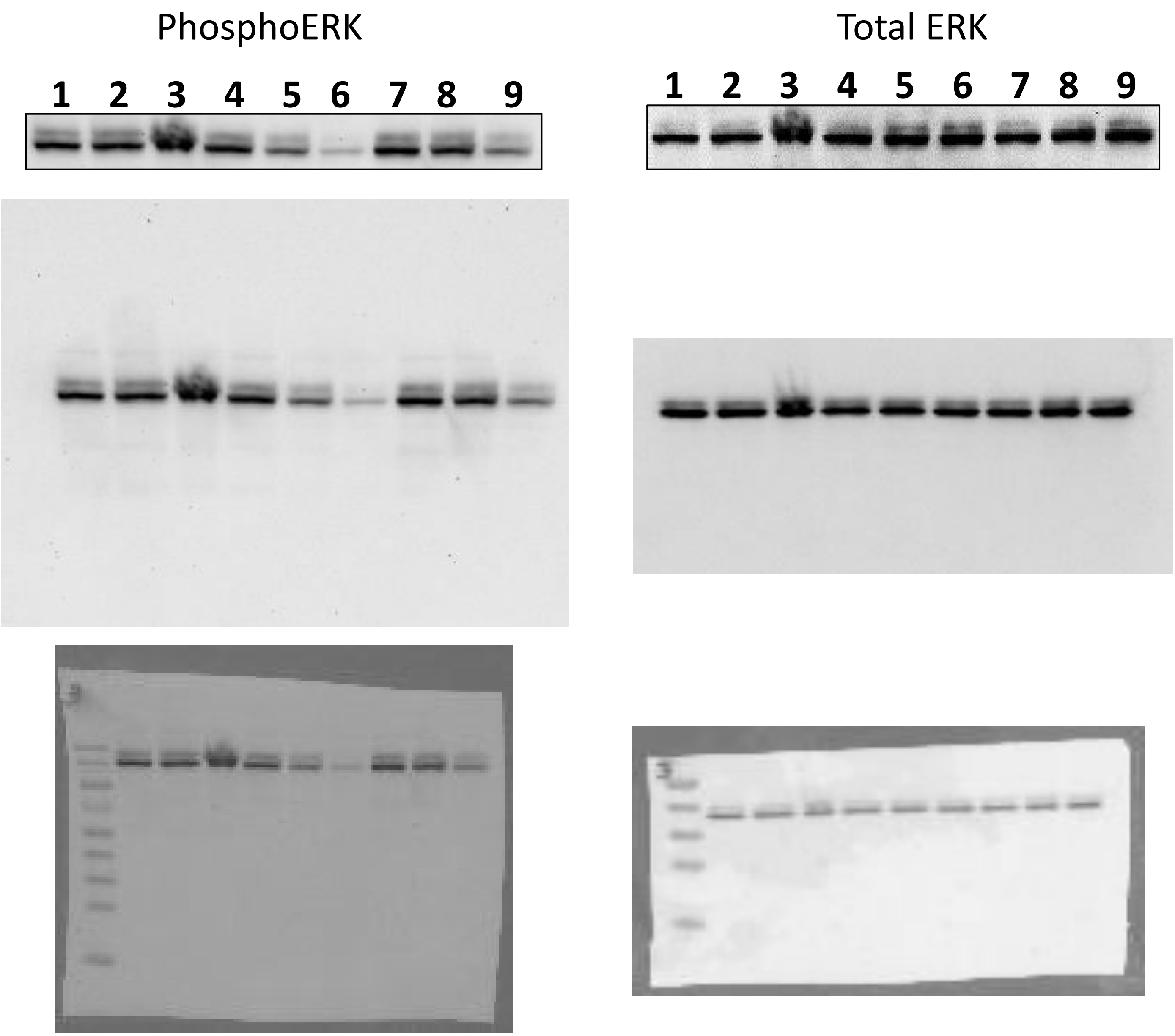

1- BaF3/*Flt3*$^{\text{ITD-D835Y}}$ - 0
2- BaF3/*Flt3*$^{\text{ITD-D835Y}}$ - 0.4nM AC220
3- BaF3/*Flt3*$^{\text{ITD-D835Y}}$ - 10nM AC220
4- BaF3/*Flt3*$^{\text{ITD-D835Y}}$ - 0.4nM HSN608
5- BaF3/*Flt3*$^{\text{ITD-D835Y}}$ - 2nM HSN608
6- BaF3/*Flt3*$^{\text{ITD-D835Y}}$ - 10nM HSN608
7- BaF3/*Flt3*$^{\text{ITD-D835Y}}$ - 0.4nM HSN748
8- BaF3/*Flt3*$^{\text{ITD-D835Y}}$ - 2nM HSN748
9- BaF3/*Flt3*$^{\text{ITD-D835Y}}$ - 10nM HSN748

BaF3/*Flt3*ITD-D835Y

Figure 2F

PhosphoFLT3

Total FLT3

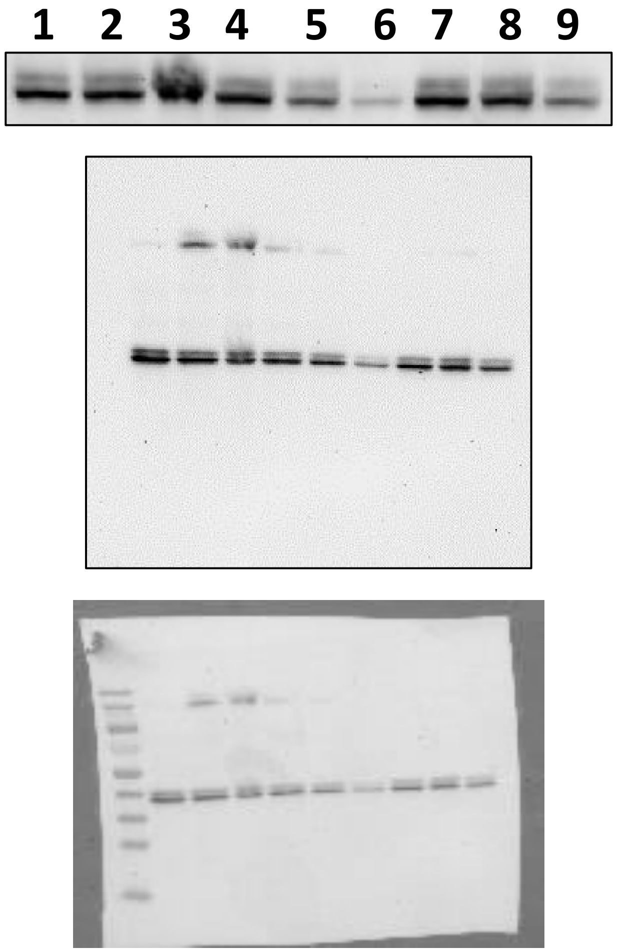

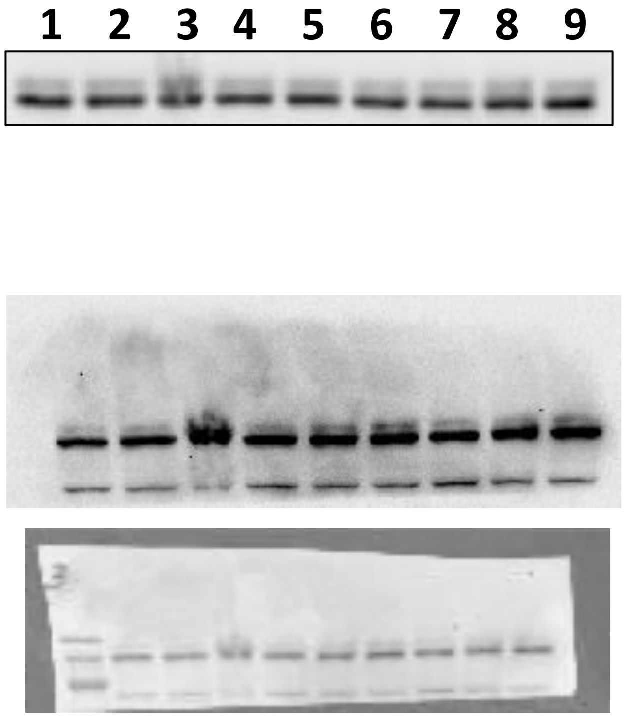

1- BaF3/*Flt3*ITD-D835Y - 0
2- BaF3/*Flt3*ITD-D835Y - 0.4nM AC220
3- BaF3/*Flt3*ITD-D835Y - 10nM AC220
4- BaF3/*Flt3*ITD-D835Y - 0.4nM HSN608
5- BaF3/*Flt3*ITD-D835Y - 2nM HSN608
6- BaF3/*Flt3*ITD-D835Y - 10nM HSN608
7- BaF3/*Flt3*ITD-D835Y - 0.4nM HSN748
8- BaF3/*Flt3*ITD-D835Y - 2nM HSN748
9- BaF3/*Flt3*ITD-D835Y - 10nM HSN748

Figure 2G

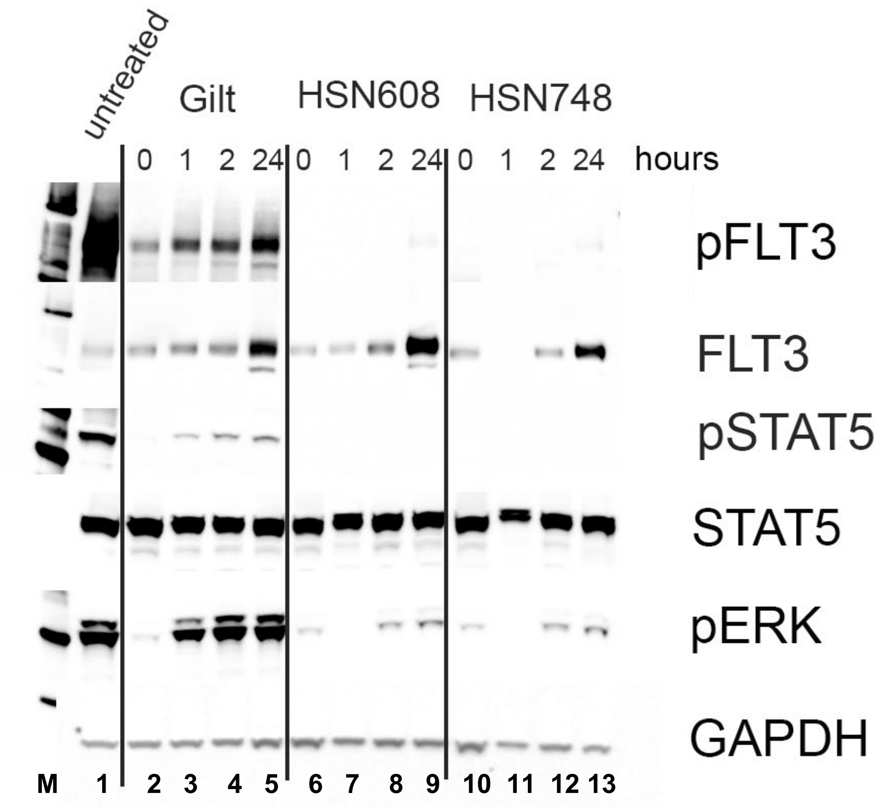

M Marker
1. MV411 cells Untreated
2. MV411 cells treated with Gilteritinib for 0 hour
3. MV411 cells treated with Gilteritinib for 1 hour
4. MV411 cells treated with Gilteritinib for 2 hour
5. MV411 cells treated with Gilteritinib for 24 hour
6. MV411 cells treated with HSN608 for 0 hour
7. MV411 cells treated with HSN608 for 1 hour
8. MV411 cells treated with HSN608 for 2 hour
9. MV411 cells treated with HSN608 for 24 hour
10. MV411 cells treated with HSN748 for 0 hour
11. MV411 cells treated with HSN748 for 1 hour
12. MV411 cells treated with HSN748 for 2 hour
13. MV411 cells treated with HSN748 for 24 hour

Figure 3D

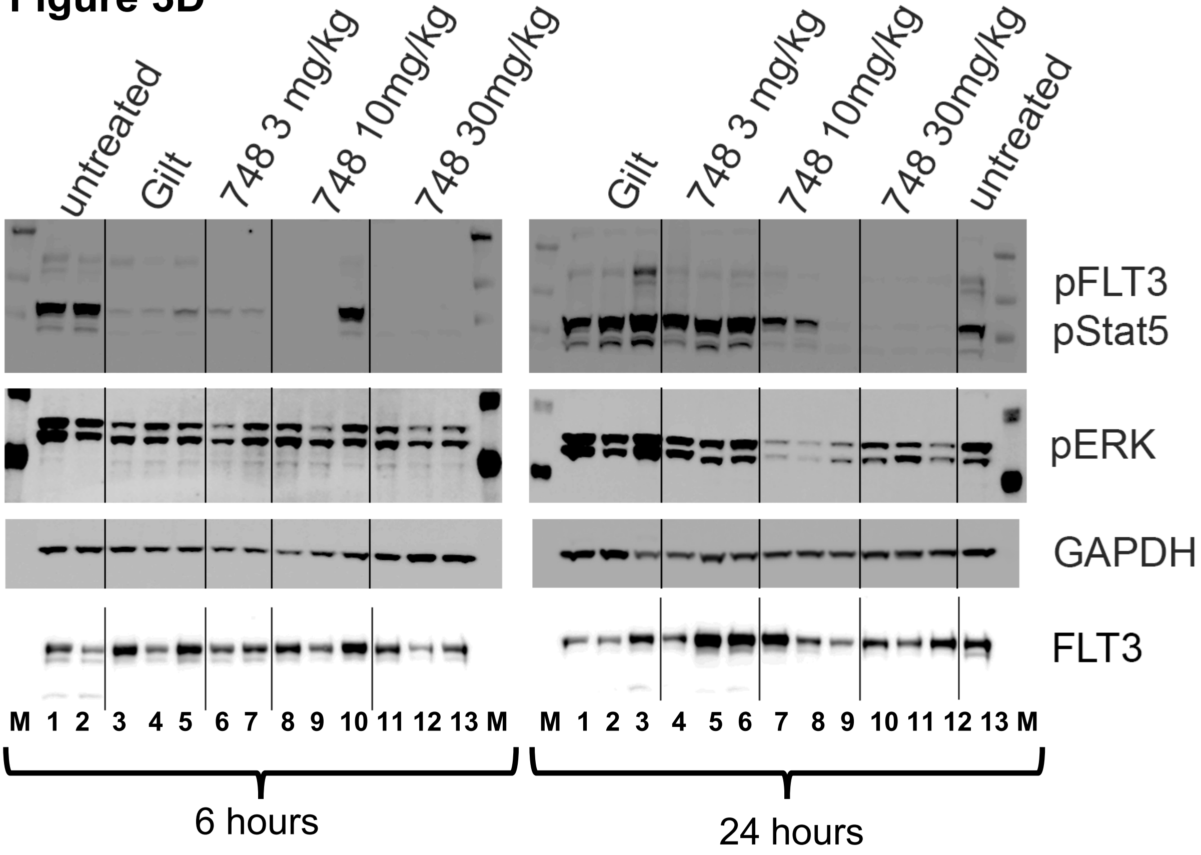

<u>Left side blot - 6 hours after drug administration</u>

M Marker
1 & 2 Lysates from Untreated control
3,4,5 Lysates from Gilteritinib treated mice
6 & 7 Lysates from 748 treated at dosage of 3mg/kg
8,9,10 Lysates from 748 treated at dosage of 10mg/kg
11,12,13 Lysates from 748 treated at dosage of 30mg/kg
M Marker

<u>Right side blot - 24 hours after drug administration</u>

M Marker
1,2,3 Lysates from Gilteritinib treated mice
4,5,6 Lysates from 748 treated at dosage of 3mg/kg
7,8,9 Lysates from 748 treated at dosage of 10mg/kg
10,11,12 Lysates from 748 treated at dosage of 30mg/kg
13 Lysates from Untreated control
